# Chemo- and Regioselective Synthesis of 3,4-Dihydropyrimidin-4-ones from 4,5-Dihydro-1,2,4-oxadiazoles and Chromium Alkoxy Alkynyl Fischer Carbene Complexes

**DOI:** 10.1021/acs.orglett.5c03642

**Published:** 2025-11-03

**Authors:** Sergio Sánchez-Alonso, M. Isabel Menéndez, Isabel Merino, Enrique Aguilar

**Affiliations:** † Centro de Innovación en Química Avanzada (ORFEO-CINQA), Instituto Universitario de Química Organometálica “Enrique Moles”, Departamento de Química Orgánica e Inorgánica, Universidad de Oviedo, C/Julián Clavería, 8, 33006 Oviedo, Spain; ‡ Departamento de Química Física y Analítica, 16763 Universidad de Oviedo, C/Julián Clavería, 8, 33006 Oviedo, Spain; § Unidad de RMN, Servicios Científico-Técnicos, Universidad de Oviedo, C/Fernando Bonguera, s/n, 33006 Oviedo, Spain

## Abstract

A completely chemo- and regioselective synthesis of pyrimidin-4­(3*H*)-ones by the reaction between 4,5-dihydro-1,2,4-oxadiazoles and chromium alkoxy alkynyl Fischer carbene complexes is reported. Overall, it is a formal (3+3) cycloaddition in which 4,5-dihydro-1,2,4-oxadiazoles participate for the first time in the intermolecular formation of six-membered heterocycles. Three points of diversity have been explored, and usually, synthetically useful isolated yields are reached, including one example at the gram scale. A reasonable mechanism, supported by DFT calculations, is proposed.

## Graphical abstract

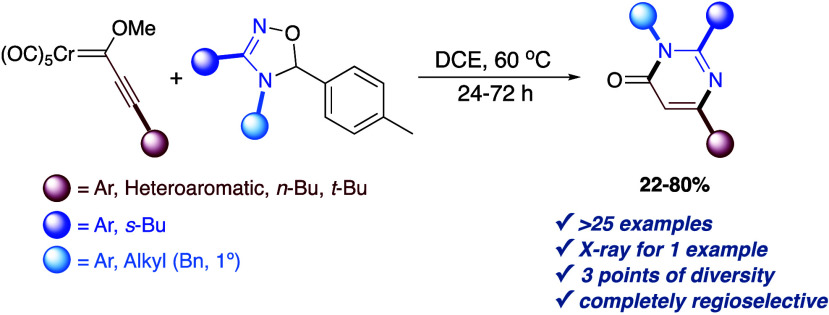

Heterocyclic compounds play a pivotal role in many aspects of our daily life, and among them, azaheterocycles (nitrogen-containing heterocycles) are ubiquitous components of both natural products and synthetic pharmaceutical drugs.^1^ Therefore, the development of new synthetic approaches toward them is of paramount interest. In this regard, we have noticed that the usefulness of 4,5-dihydro-1,2,4-oxadiazoles in heterocyclic synthesis is rather limited, and their chemistry is still underdeveloped. In fact, in the only examples reported so far, they have participated in intramolecular reactions as the source of N-centered radicals such as in a skeletal rearrangement to produce quinazolinones either by a thermal oxidation^2^ or by electrochemistry^3^ (Scheme 1A, top) or in the synthesis of spiro-azalactams by a double functionalization of arenes via a photocatalytic electron transfer (Scheme 1A, bottom).^4^ They are also able to rearrange to benzimidazoles through a nitrene intermediate, under LED conditions by energy transfer catalysis, providing that an aromatic group is linked to the sp^3^ N (Scheme 1B).^5^ In addition, they undergo a thermal (3,3)-rearrangement followed by aldehyde extrusion leading to pyrrols when alkenyl substituents are placed at positions 4 and 5 (Scheme 1C).^6^ However, most remarkable is the fact that there are only two examples of intermolecular cycloadditions involving 4,5-dihydro-1,2,4-oxadiazoles; thus, they have proven to be useful synthons as [N–C–N] surrogates (or masked 1,1-diamine synthons) for the preparation of imidazoles (Scheme 1D). In these reactions against activated alkynes (ynamides) under gold catalysis conditions,^7^ they behave as nitrene transfer reagents and gold α-iminocarbene complexes have been postulated as intermediates.^8^ In a second case, a very recent publication has shown that NH-4,5-dihydro-1,2,4-oxadiazoles behave as single-nitrogen transfer reagents in their reactions with ynones, either assisted by Sc­(OTf)_3_ or under Au­(I)/Sc­(OTf)_3_ synergistic catalysis conditions (Scheme 1E).^9^

**Scheme 1:**
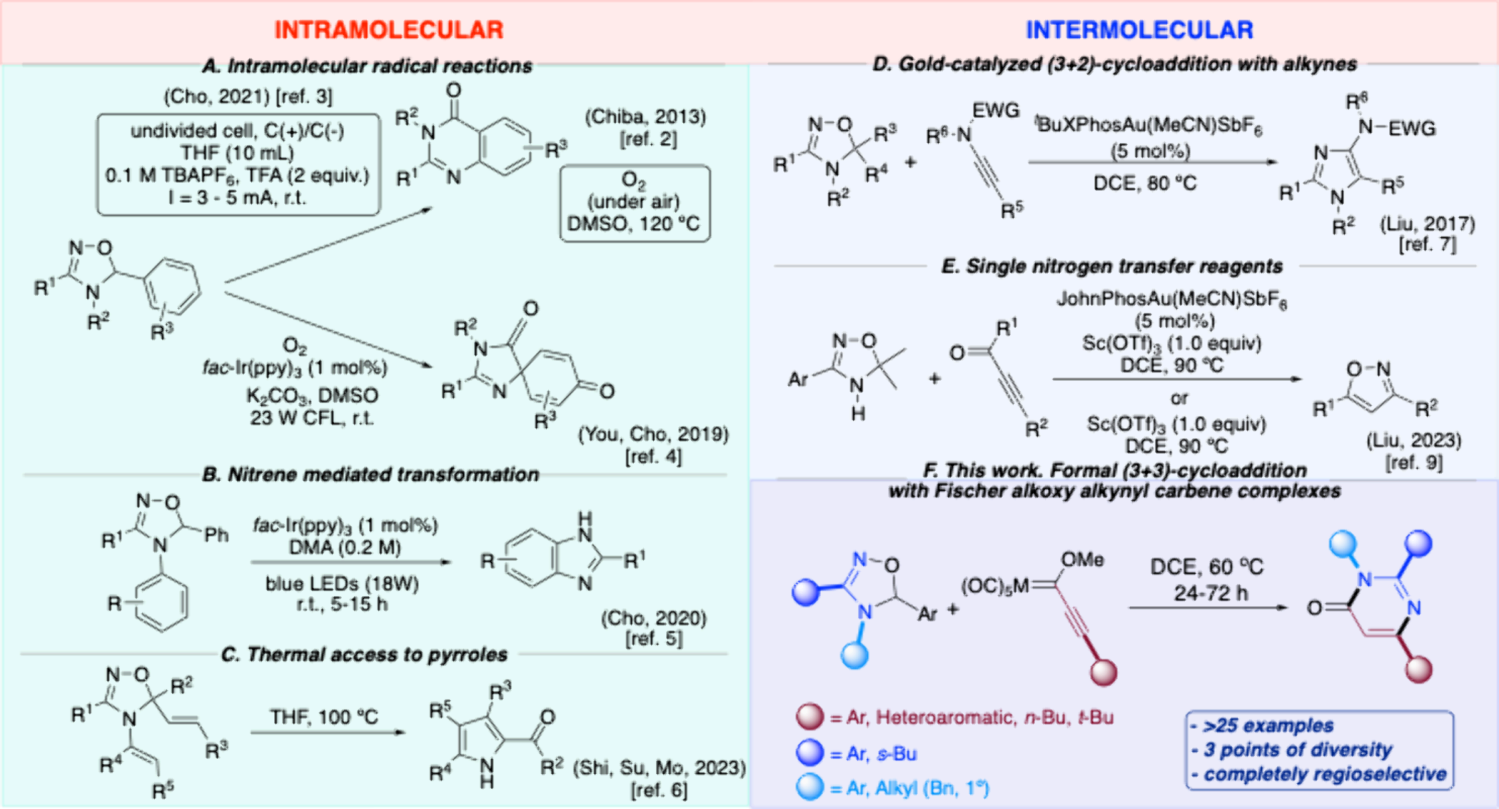
4,5-Dihydro-1,2,4-oxadiazoles in Heterocyclic Reactions

On the other hand, the paramount role of group 6 metal Fischer carbene complexes (FCCs) as highly valuable reagents in synthetic organic chemistry relies mainly on the following: (1) their relatively affordable synthesis at a multigram scale, (2) their friendly purification, storage, and manipulation, (3) their wide range of reactivity patterns (chemical multitalents)^10^ and easy removal of the metal fragment, and (4) the possibility of performing diastereo- and enantioselective transformations by employing chiral auxiliaries.^11^ For all of these reasons, group 6 metal FCCs have found widespread application in heterocyclic synthesis, becoming appropriate starting materials for the preparation of three- to eight-membered ring heterocycles.^12^

Considering the aforementioned reactivity of both FCCs and 4,5-dihydro-1,2,4-oxadiazoles, we envisioned that both substrates could combine in a new (3+3) fashion leading to 3,4-dihydropyrimidin-4-ones (Scheme 1F). Nevertheless, a (3+2) cycloaddition to produce pyrazoles was not initially discarded as an alternative possible reaction pathway.

Herein, we report the regioselective synthesis of pyrimidin-4­(3*H*)-ones by formal (3+3) cycloaddition between 4,5-dihydro-1,2,4-oxadiazoles and chromium alkoxy alkynyl Fischer carbene complexes.

We initially selected chromium carbene complex **1a** and dihydrooxadiazole **4a** as model substrates. We just mixed them in a 1:1.2 ratio and tested their reaction at 60 °C for 24 h in THF, hexane, 1,4-dioxane, toluene, DCM, and DCE (Table 1, entries 1–6, respectively); in all cases, pyrimidin-4-(3*H*)-one **5a**, coming from a formal (3+3) cycloaddition, was the only isolated product, with DCE being the solvent that provided a better yield. Structural elucidation of **5a** was accomplished by 1D and 2D NMR spectroscopy and HRMS, and the completely chemo- and regioselective nature of the process could also be established (*vide infra*). Increasing the temperature to 70 °C, employing a larger excess of **4a** (2 equiv), or a slow addition of the carbene complex did not achieve any improvement in yield (entries 7–9). A better result was achieved by allowing the reaction to reach completion at rt, although 4 days was required (entry 10). Finally, the best conditions found involved the employment of a 1.5-fold excess of dihydrooxadiazole and heating in DCE at 60 °C for 36 h (entry 11). Tungsten alkoxy alkynyl carbene complex **2a** also underwent the same type of reaction, although more time was required, and a lower yield was reached (entry 12). Remarkably, the transformation did not proceed at all with analogous methyl propiolate **3** (entry 13), thus indicating that the metal moiety is crucial for the reaction outcome.

**Table 1:**
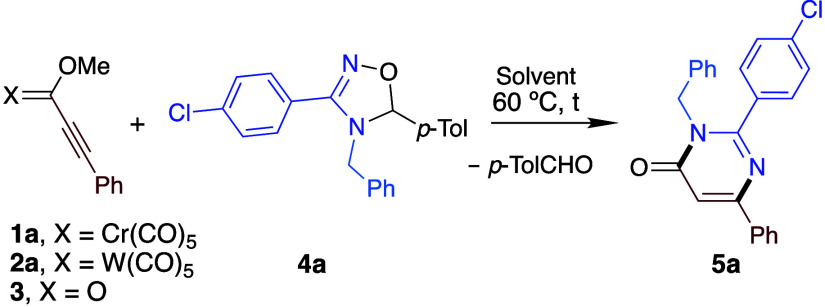
Optimization of the Reaction Conditions

entry	sm^a^	sm:**4a**	*t* (h)	solvent	yield of **5a** (%)^b^
1	**1a**	1:1.2	24	THF	(23)
2	**1a**	1:1.2	24	hexane	(14)
3	**1a**	1:1.2	24	1,4-dioxane	(36)
4	**1a**	1:1.2	24	toluene	(44)
5	**1a**	1:1.2	24	DCM	(52)^c^
6	**1a**	1:1.2	24	DCE	59
7	**1a**	1:1.2	24	DCE	17^d^
8	**1a**	1:2	24	DCE	40
9	**1a**	1:1.2	24	DCE	54^e^
10	**1a**	1:1.2	96	DCE	66^f^
11	**1a**	1:1.5	36	DCE	80 (81)
12	**2a**	1:1.5	48	DCE	37
13	**3**	1:1.5	48	DCE	–

a sm = starting material.

b Isolated yields. In parentheses are NMR estimated yields using 1,3,5-trimethoxybenzene as the internal standard.

c Reaction performed in a sealed tube.

d Reaction carried out at 70 °C.

e **1a** was added dropwise for 2 h over **4a** at 60 °C.

f Reaction carried out at rt.

Once the optimized conditions were established, we explored the reaction scope starting by analyzing the effect of the variation on the dihydrooxadiazole component (Scheme 2). Thus, on one hand, at position 3 of the dihydrooxadiazole, the reaction tolerates aromatic substituents bearing both electron-withdrawing (**5a**, **5e**, **5f**, and **5h–j**) and electron-donating (**5b**, **5d**, and **5k**) groups, besides Ph (**5c**). Interestingly, an X-ray structural determination of **5c** corroborated the proposed regiochemistry.^13^ *ortho*-substituted (**5d** and **5e**) and *meta*-substituted aromatic (**5f**) as well as secondary aliphatic groups **(5g**) are also feasible substituents at that position. On the other hand, regarding the substitution at position 3 of the dihydropyrimidone core, benzyl (**5a**–**g**), *p*-methoxyphenyl (**5h**), phenyl (**5i**), and *n*-butyl (**5j** and **5k**) groups provide the corresponding pyrimidin-4­(3*H*)-ones in satisfactory yields.

**Scheme 2:**
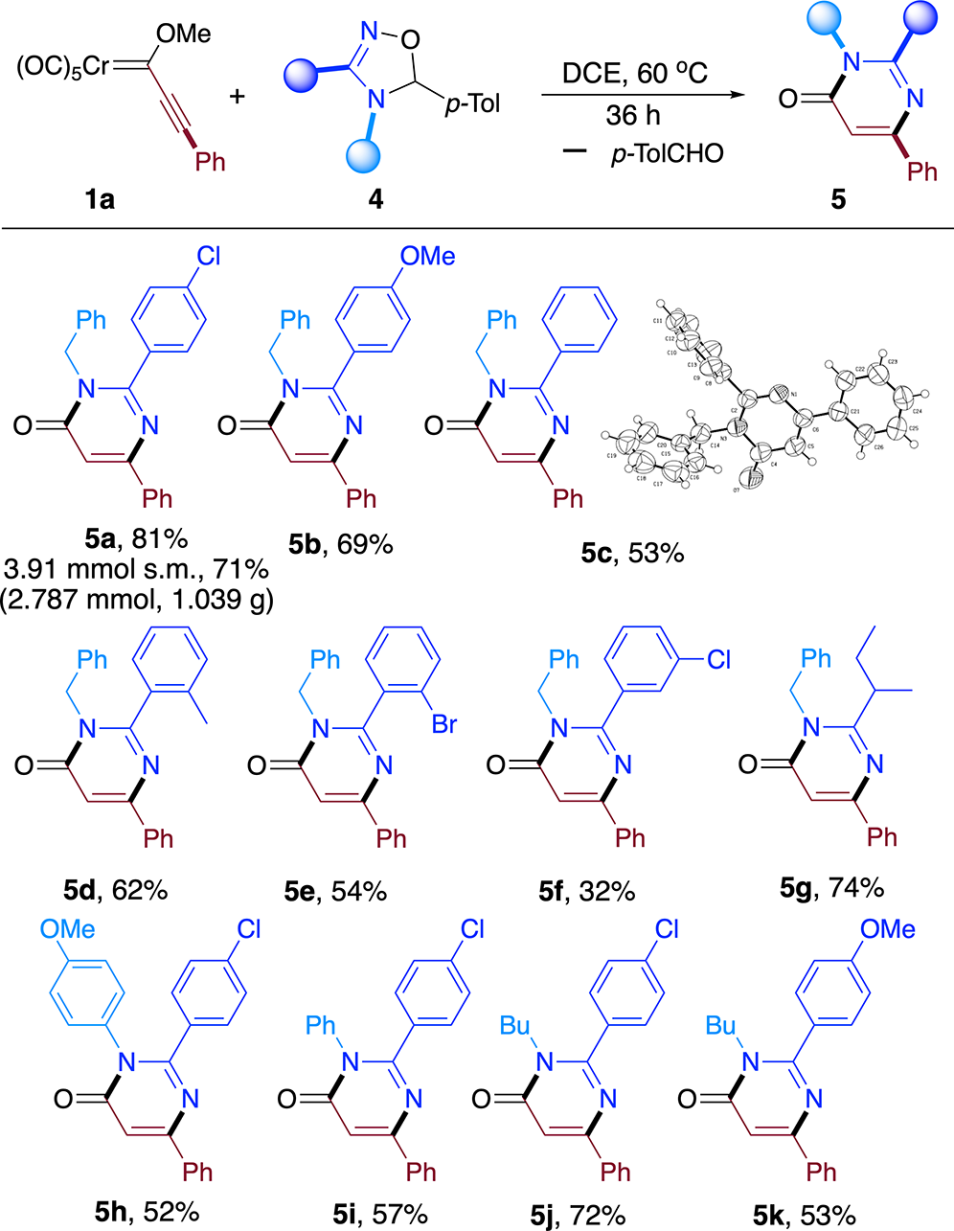
Scope of the Reaction upon Variation of the Dihydrooxadiazole

Regarding the substitution pattern of the Fischer carbene complex, the reaction is compatible with electron-donating (**5l–p**, **5u–y**, **5aa**, and **5ab**) and electron-withdrawing aromatic substituents (**5t** and **5z**). *Ortho*-substituted phenyl groups (**5n**, **5o**, **5v**, **5x**, **5aa**, and **5ab**) and heteroaromatics (**5p**) were also satisfactorily employed, as well as alkenyl (**5q**) and primary (**5s**) and tertiary (**5r**) alkyl groups. In most cases, synthetically useful yields were achieved, the 38% yield of **5r** being the lower limit (Scheme 3).

**Scheme 3:**
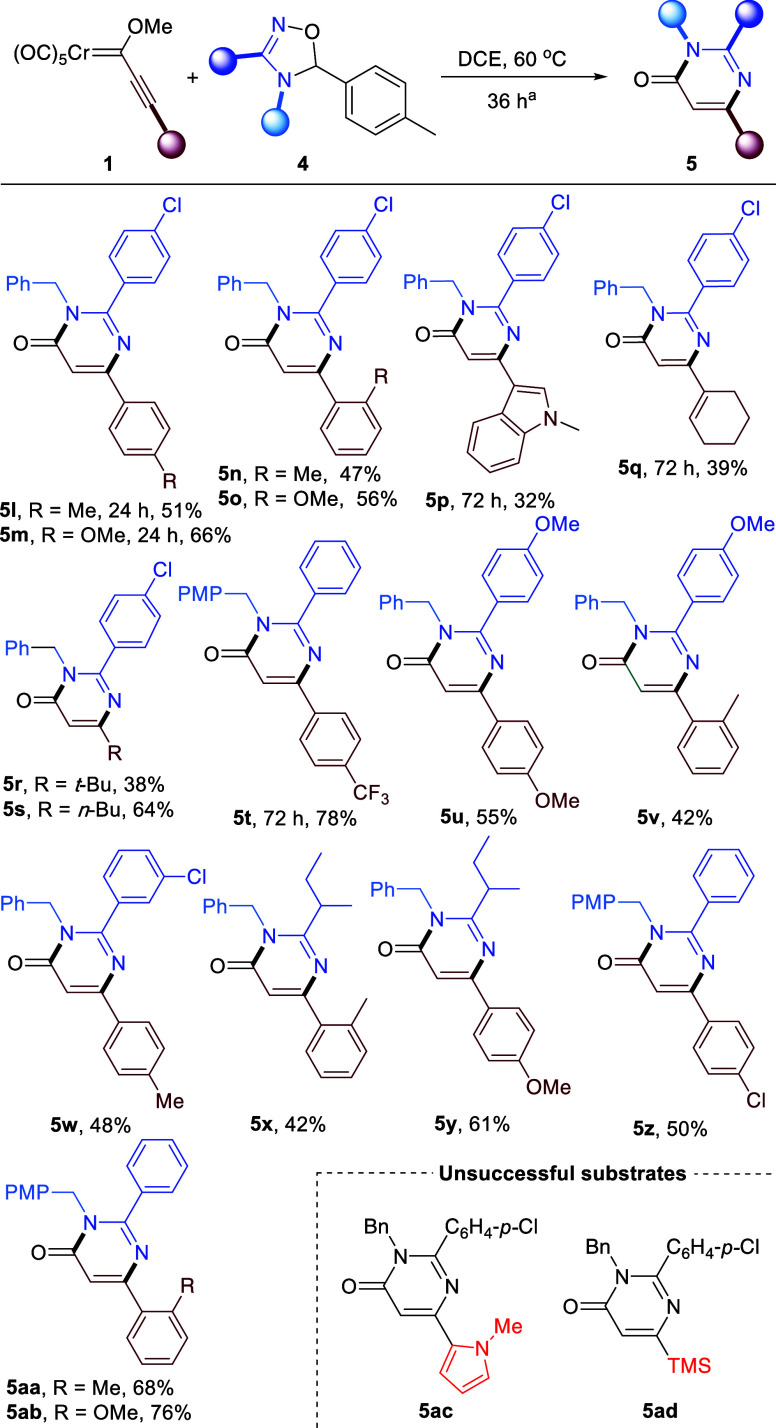
Scope of the Reaction upon Variation of the Fischer Carbene Complex

a
*t* = 36 h, unless otherwise indicated. PMP = *p*-methoxyphenyl.

In addition, the reaction seems to be rather sensitive to sterics in the FFC component as only modest yields were achieved for the bulky *t*-Bu derivative (**5r**). Also, limitations on the scope were found as the reaction failed when attempting to prepare dihydropyrimidone **5ac** or **5ad** from an (*N*-methyl)­pyrrol-2-yl- or TMS-derived FFC, respectively (Scheme 3, bottom). On the contrary, little effect on the reaction yield was observed when varying the substituent at position 5 of the dihydrooxadiazole (which ends up forming part of a released carbonyl compound).^14^

Furthermore, a gram scale reaction could be performed between **1a** and **4a** under the optimized reaction conditions; 1.039 g of **5a** was synthesized (71% yield (Scheme 2)), which highlights the synthetic usefulness of this transformation.

Some additional tests were performed in order to gain some information about the reaction mechanism. Thus, 4-isopropoxypyrimidine **7** was the very major product detected in the crude product when FCC **6** bearing a bulky isopropoxy group was employed, although it could be isolated in only 23% yield (Scheme 4a). Also, the reaction of FCC **1a** with N-unsubstituted 4,5-dihydro-1,2,4-oxadiazole **4n** led to a 58% yield of 4-methoxypyrimidine **8** (Scheme 4b). A very similar yield of **8** (60%) was reached when acetone-derived N-unsubstituted 4,5-dihydro-1,2,4-oxadiazole **9a** was used instead, thus highlighting the almost negligible effect of the nature of the released carbonyl compound (within the tested ones) on the reaction yield. In addition, deuteration experiments helped to confirm the position of the metal fragment prior to the hydrolysis leading to the final products.^14^

**Scheme 4:**
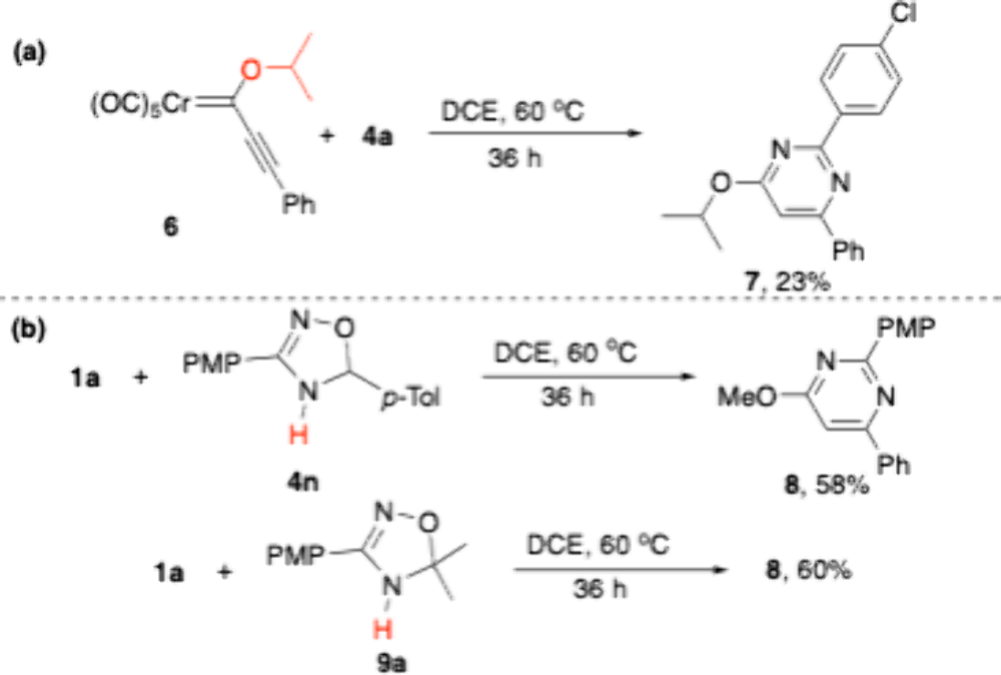
Mechanistic Probes in (a) the Reaction with FCC **6** Bearing a Bulky Alkoxy Group and (b) the Reaction with N-Unsubstituted 4,5-Dihydro-1,2,4-oxadiazoles

With all of the data found, the proposed reaction mechanism should involve an initial 1,4-addition of the more nucleophilic sp^2^ N atom of the dihydrooxadiazole moiety to the β-carbon atom of the triple bond of metal carbene complex **10** leading to zwitterionic species **I** (Scheme 5, top). The alternative nucleophilic attack on the electrophilic carbene carbon (Scheme 5, bottom, purple arrows) should lead to opposite regioisomer **11**, which was not observed. Additionally, the formation of imidazole **12** by a formal (3+2) cycloaddition (Scheme 5, bottom, blue arrows) was not detected either. Then, the evolution of allenyl metalate intermediate **I** by a cyclization step involving a [1,2]­pentacarbonylmetal migration should form (2*H*)-1,3,5-oxadiazocinium intermediate **II** (Scheme 5, top, route A, brown arrows), now metalated at position 7; although ring closure to medium-sized rings involving a [1,2]­[M­(CO)_5_] shift is well documented, this one would be the first example in which an eight-membered ring intermediate is formed.^15^ The fact that a lower reaction yield was obtained when bulky FFC **6** was employed (Scheme 4a) suggests that this cyclization step is hampered in that case. Next, a 6π-electro-cyclization reaction on resonant form **II-A** ^16^ or a nitrogen nucleophilic attack on resonant form **II-B** would lead to metalated 6-methoxy-7-oxa-1,3-diazabicyclo[4.2.0]­octa-2,4-dien-1-ium **III**, which would undergo the elimination of *p*-tolualdehyde to produce a new zwitterionic pyrimidine derivative **IV**. Alternatively, all of these sequential steps may occur in either a synchronous or asynchronous concerted pathway (Scheme 5, top, route B, red arrows). Finally, removal of the metal moiety^15b^ from metalated intermediate **IV** would allow the formation of isolated pyrimidin-4­(3*H*)-ones **5** for N-substituted 4,5-dihydro-1,2,4-oxadiazoles or 4-methoxypyrimidine **8** when N-unsubstituted 4,5-dihydro-1,2,4-oxadiazoles are employed.

**Scheme 5:**
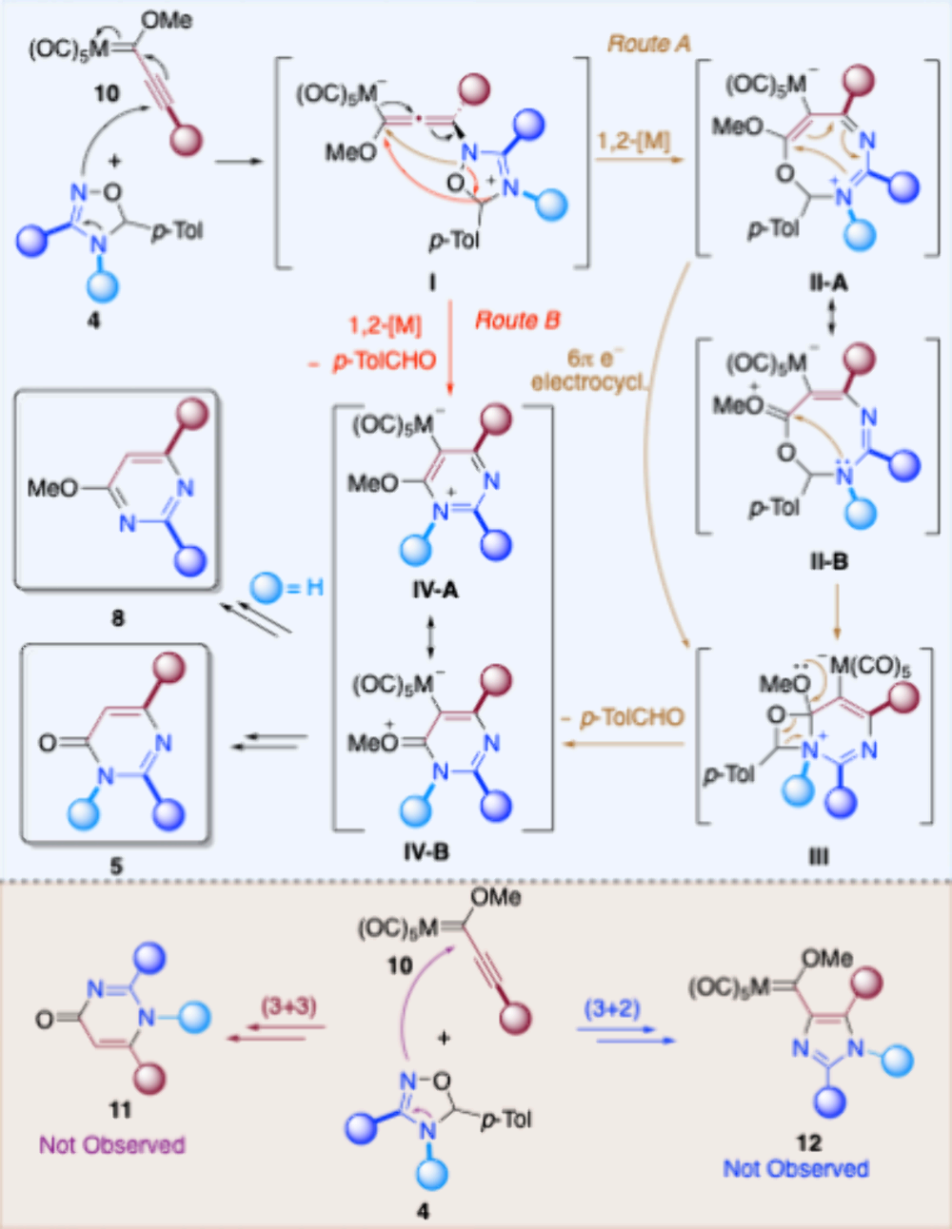
Proposed Reaction Mechanism

DFT calculations (for **1a** and **4a**) allowed the confirmation of route A of the proposed mechanism as the preferred reaction pathway.^14^ They show that the reaction is highly thermodynamically favored (Scheme S3). Only the first transition state (**ts-Ia**, 9.9 kcal/mol) is higher in energy than the reactants. The reaction proceeds through allenyl intermediate **Ia** (−1.2 kcal/mol) and then forms eight-membered ring **IIa** (−48.9 kcal/mol), which undergoes intramolecular nucleophilic attack (**ts-IVa**, −22.6 kcal/mol), releasing *p*-tolualdehyde and generating six-membered ring **IVa** (−69.4 kcal/mol). Demetalation of **IVa** requires water and occurs in two steps: 1,2-addition to the double bond and assisted deprotonation. This water-mediated process also enables isotope incorporation (e.g., D from D_2_O). Final product **5a** (−102.7 kcal/mol) is highly stable.

In conclusion, we have developed a simple methodology to prepare 3,4-dihydropyrimidin-4-ones in a chemo- and regioselective manner from the reaction of chromium alkoxy alkynyl carbene complexes and 4,5-dihydro-1,2,4-oxadiazoles, both unconventional but valuable reagents, which provide wide variability in the final products. This novel formal (3+3) process tolerates the presence of three points of diversity bearing either aromatic or aliphatic substituents, and moreover, it allows for the preparation of a wide number of pyrimidin-4­(3*H*)-ones in synthetically useful yields, including one of them at gram scale. Taken together, this strategy represents the first case in which 4,5-dihydro-1,2,4-oxadiazoles behave as [N–C–N] synthons for the formation of six-membered heterocycles through a formal (3+3) cycloaddition. The proposed mechanism was confirmed by DFT calculations.

## Supplementary Material



## Data Availability

The data underlying this study are available in the published article, in its Supporting Information, and openly available in Repositorio Institucional de la Universidad de Oviedo (RUO) at https://hdl.handle.net/10651/80685.

## Notes

Effect of the released carbonyl compound on the reaction yield, deuteration and NMR mechanistic experiments, DFT calculations, full experimental procedures, spectroscopic and physical data, and NMR spectra for all new compounds (PDF)

## Notes

This Letter is dedicated to the memory of Prof. Iván Lavandera García and graduate student Antonio Velázquez-Stavínov.
