# Supplementary material for: Chemo- and Regioselective Synthesis of 3,4-Dihydropyrimidin-4-ones from 4,5-Dihydro-1,2,4-oxadiazoles and Chromium Alkoxy Alkynyl Fischer Carbene Complexes
Source: Org Lett. 2025 Nov 3;27(45):12453–8. doi: 10.1021/acs.orglett.5c03642 (PMC12624828; doi:10.1021/acs.orglett.5c03642)

Chemo- and regioselective synthesis of 3,4-dihydropyrimidin-4-ones from 4,5-dihydro-1,2,4-oxadiazoles and chromium alkoxy alkynyl Fischer carbene complexes

Sergio Sánchez-Alonso,[a] M. Isabel Menéndez,[b] Isabel Merino[c] and Enrique Aguilar[a]*

[a] Centro de Innovación en Química Avanzada (ORFEO-CINQA). Instituto Universitario de Química Organometálica "Enrique Moles". Departamento de Química Orgánica e Inorgánica. Universidad de Oviedo. C/ Julián Clavería, 8. 33006, Oviedo, Spain.

[b] Departamento de Química Física y Analítica. Universidad de Oviedo. C/ Julián Clavería, 8. 33006, Oviedo, Spain.

[c] Unidad de RMN. Servicios Científico-Técnicos. Universidad de Oviedo. C/ Fernando Bonguera, s/n. 33006, Oviedo, Spain.

Electronic Supporting Information

TABLE OF CONTENTS

Page

1. Effect of the substituent at position 5 of the 4,5-dihydro-1,2,4-oxadiazole in the reaction yield — **SI-4**

2. Deuteration experiments — **SI-4**

3. Theoretical calculations — **SI-5**

- Cartesian coordinates, absolute electronic (E) and Gibbs (G) energies (in atomic units), and imaginary frecuencies (ν_i) of the transition states — **SI-7**

4. ^{1}H NMR mechanistic studies — **SI-19**

5. Experimental section — **SI-22**

- General Methods — **SI-22**

- Group VI metal Fischer carbene complexes 1, 2 and 6 employed — **SI-24**

- Synthesis of 4,5-dihydro-1,2,4-oxadiazoles 4. General Procedure A — **SI-24**

- Physical and spectroscopic data of 4,5-dihydro-1,2,4-oxadiazoles 4 — **SI-26**

- Synthesis of 3-(4-methoxyphenyl)-5-(*p*-tolyl)-4,5-dihydro-1,2,4-oxadiazole 4n — **SI-33**

- Optimization of the reaction conditions. General Procedure for the determination of the NMR estimated yield. — **SI-33**

- Synthesis of 3,4-dihydropyrimidin-4-ones 5. General Procedure B — **SI-34**

- Physical and spectroscopic data of 3,4-dihydropyrimidin-4-ones 5 — **SI-34**

- Synthesis of 2-(*p*-chlorophenyl)-4-isopropoxy-6-phenylpyrimidine 7 — **SI-50**

- Synthesis of 4-methoxy-2-(*p*-methoxyphenyl)-6-phenylpyrimidine 8 — **SI-51**

- Gram-scale reaction for the synthesis of 3,4-dihydropyrimidin-4-one 5a — **SI-51**

- Deuteration experiments: Synthesis of 3,4-dihydropyrimidin-4-one D-5a — **SI-52**

- Synthesis of 4,5-dihydro-1,2,4-oxadiazoles 9 — **SI-53**

- **Synthesis of 3-(*p*-chlorophenyl)-4-(*p*-methoxyphenyl)-5-phenyl-4,5-dihydro-1,2,4-oxadiazole 13** **SI-54**

6. Tables with 2D-NMR Experiments for Selected Compounds SI-55

7. X-Ray structure of 4,5-dihydro-1,2,4-oxadiazole 5c SI-59

8. References SI-61

9. Copies of ^{1}H-, ^{2}H-, ^{13}C-NMR and 2D-NMR Spectra SI-63

- **^{1}H and ^{13}C-NMR spectra for 4,5-dihydro-1,2,4-oxadiazoles 4** **SI-64**
- **^{1}H and ^{13}C-NMR spectra for 3,4-dihydropyrimidin-4-ones 5 and 2D NMR (COSY, HSQC, HMBC) experiments for selected compounds (5a,b,d,g)** **SI-77**
- **^{1}H and ^{13}C-NMR spectra for 4-isopropoxypyrimidine 7** **SI-117**
- **^{1}H and ^{13}C-NMR spectra for 4-methoxypyrimidine 8** **SI-118**
- **^{1}H, ^{13}C, and ^{2}H-NMR spectra for deuterated 3,4-dihydropyrimidin-4-ones D-5a** **SI-119**
- **^{1}H and ^{13}C-NMR spectra for 4,5-dihydro-1,2,4-oxadiazole 9b** **SI-123**
- **^{1}H and ^{13}C-NMR spectra for 4,5-dihydro-1,2,4-oxadiazole 13** **SI-124**

1. Effect of the substituent at position 5 of the 4,5-dihydro-1,2,4-oxadiazole in the reaction yield

The role of substituent at position 5, which ends up forming part of a released carbonyl compound, in the reaction yield was also analyzed. In fact, little effect was observed when the aldehyde released, from the 4,5-dihydro-1,2,4-oxadiazole partner, was benzaldehyde, instead of the more commonly employed-by-us *p*-tolualdehyde (compare **5h**, Scheme 2 *vs* Scheme S1, *top*). This finding was corroborated when acetone derived 4,5-dihydro-1,2,4-oxadiazole **9b** was tested, as 62% isolated yield of **5b** was reached (compare **5b**, Scheme 2 *vs* Scheme S1, *bottom*).

Scheme S1. Effect of the released carbonyl compound in the reaction yield

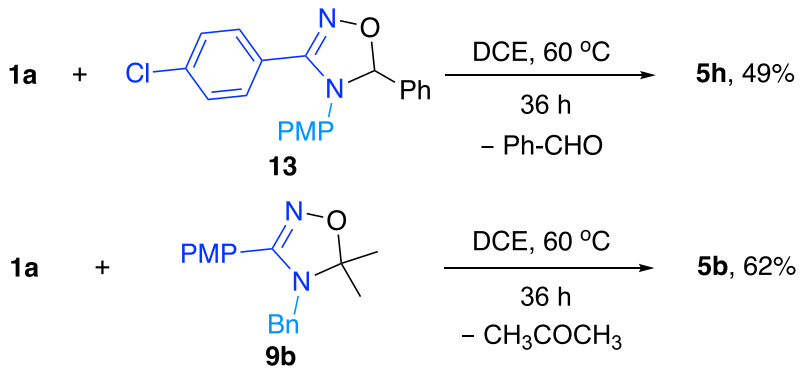

2. Deuteration Experiments

Deuteration experiments were carried out to shed some light into the reaction mechanism. Thus, the reaction was performed in the presence of D_2O both to facilitate the cleavage of the chromium-C bond and, mainly, to serve as an isotopic probe: the position of deuterium in the dihydropyrimidone ring should be that of the metal moiety immediately prior to the breaking of its bond to the heterocyclic skeleton. In those reactions, a 60% of deuterium incorporation was observed on position 5 when 1.5 equiv of D_2O were employed, which increased to a 90% if 5 equiv of D_2O were used (Scheme S2).

Scheme S2. Deuteration experiments

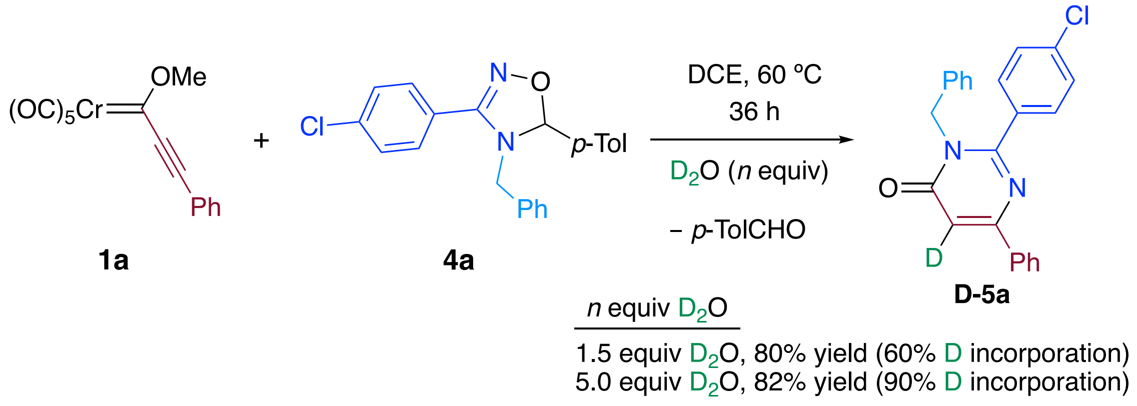

3. Theoretical Calculations

All calculations have been performed with the density functional theory, using M06 functional,[1] as implemented in the Gaussian09 suit of programs.[2] The 6-31G(d) basis set[3] was used for all atoms involved but for chromium, which has been described with the LANL2DZ pseudopotential.[4] All stationary points located in the potential energy surface of the reaction under study were fully optimized and characterized to be either minima or transition states (first-order saddle points) by the calculation of the corresponding harmonic vibrational frequencies. Thermal free energy corrections in DCE solution (G_{therm}) were calculated using the standard procedure starting from the molecular partition functions developed for computing gas-phase thermodynamics properties within the ideal gas, rigid rotor, and harmonic oscillator approximations at a pressure of 1 atm and a temperature of 298.15 K.[5] Energies discussed in the text are Gibbs free energies in DCE solution referred to the complexes indicated in each case. Energy values are indicated in kcal/mol.

DFT calculations (M06/6-31G(d), LANL2DZ for Cr) were performed for the reaction between chromium FFC **1a** and dihydrooxadiazole **4a**, chosen as a model, as shown in Scheme S3, and lead to a Gibbs energy profile where only the initial transition state, **ts-Ia** (9.9 kcal/mol) has an energy over that of the reactants. It corresponds to the nucleophilic attack of the dihydrooxazole moiety to the metal carbene complex, yielding, first, allenyl intermediate **Ia** (-1.2 kcal/mol) and then, the stable eight-membered cyclic intermediate **IIa** (-48.9 kcal/mol). **IIa** undergoes an intramolecular nucleophilic addition of the substituted N to the C-OMe one. The transition state for this step, **ts-IVa** (-22.6

kcal/mol), evolves to the release of the *p*-tolualdehyde and the formation of the six-membered ring **IVa-A** (-69.4 kcal/mol), direct precursor of the final product. Proposed bicyclic intermediate **IIIa** (Scheme 5) was not detected.

Scheme S3. M06/6-31G(d), (LANL2DZ for Cr) Gibbs energy profile (in kcal/mol) for the model reaction confirming the mechanism proposed in Scheme 5 (*Route A*).

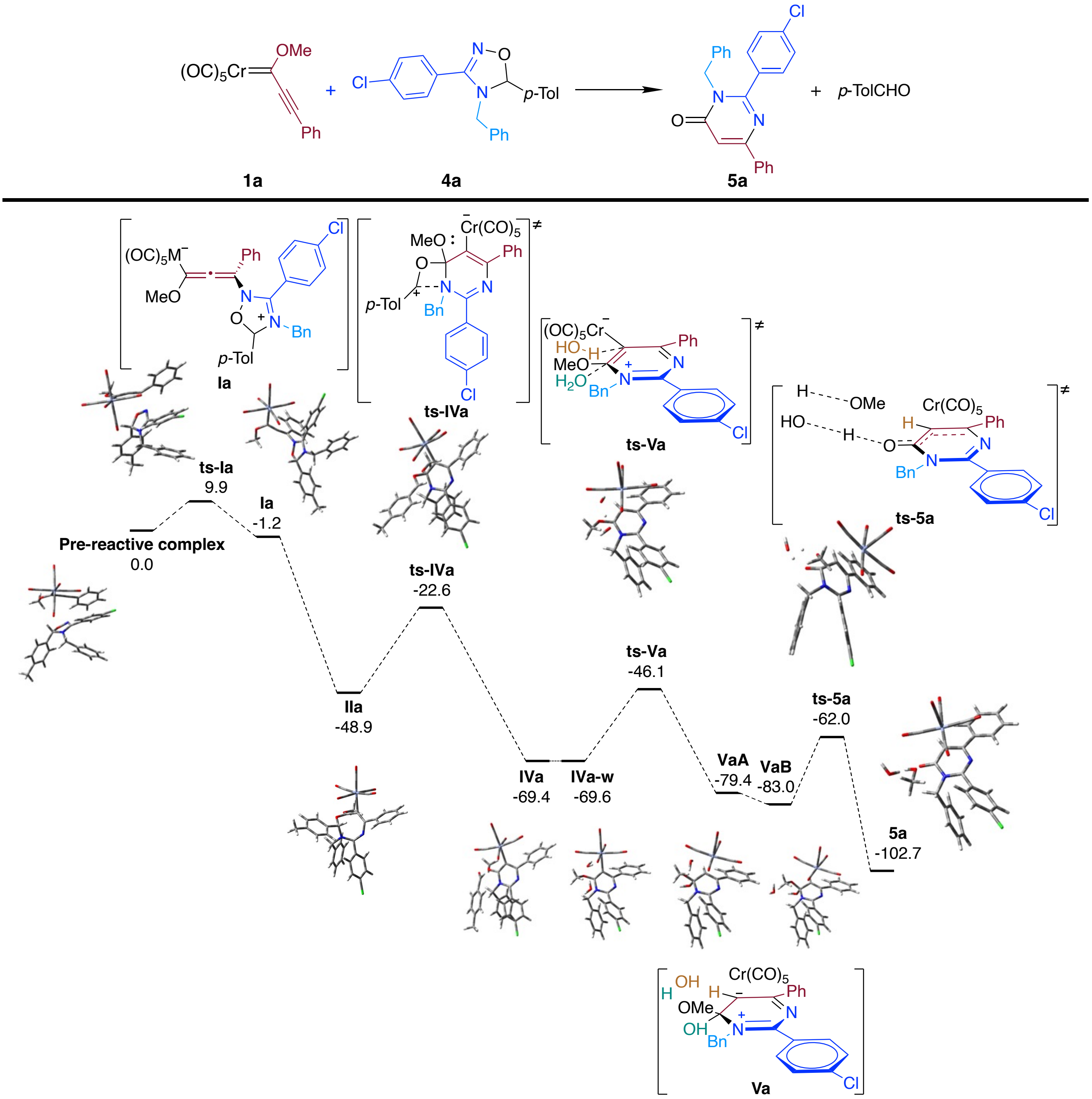

Regarding the final step, some water must be present in the environment to perform the demetalation of **IVa** (-69.6 kcal/mol). A simple model made of two water molecules

indicates that demetalation happens through a two-step route, one step for the 1,2-addition of a water molecule to the C-C double bond (present in resonant structure **IV-A**, Scheme 5; this is the step for the incorporation of D from D_2O) and the second for the assisted deprotonation of the just added OH group along with the elimination of methanol. Intermediate **Va** (**VaA/VaB** -79.4/-83.0 kcal/mol, depending on the orientation of the assisting water molecule) connects both steps. Eventually, product **5a** (-102.7 kcal/mol) forms. The large stability of this species indicates that thermodynamics strongly favors the reaction under study.

Therefore, DFT calculations (M06/6-31G(d) + LANL2DZ for Cr) confirm the mechanism proposed in Scheme 5, *Route A*.

Cartesian coordinates, absolute electronic (E) and Gibbs (G) energies (in atomic units), and imaginary frecuencies (ν_i) of the transition states

Pre-reactive complex
E = -2607.268539 G = -2606.798804

C	-0.99407800	0.57686200	0.25611500
O	-1.83082100	-0.73916500	1.75713000
N	-0.93145600	0.32974800	1.52570100
C	-0.23431000	1.67149300	-0.35317900
C	0.27093800	1.55410300	-1.65093100
C	-0.02451300	2.85245100	0.36455400
C	0.97340900	2.60066200	-2.23274100
H	0.11537500	0.63595800	-2.21519600
C	0.67403000	3.90681300	-0.20709100
H	-0.44115700	2.95065900	1.36585600
C	1.16149700	3.76792900	-1.50219100
H	1.37151900	2.51354700	-3.24084000
H	0.83116700	4.83343200	0.34028700
Cl	2.02803200	5.10136100	-2.23568100
C	-2.62682200	0.12005200	-1.59530400
H	-2.08098600	-0.05415200	-2.53512600
H	-3.48796000	-0.56195600	-1.61160100
C	-3.09056500	1.55256500	-1.51954700
C	-2.79643000	2.44993400	-2.54438400
C	-3.77546500	2.01142100	-0.39183400
C	-3.16792300	3.78786200	-2.44365900
H	-2.24989900	2.10085000	-3.42154900
C	-4.15246900	3.34530300	-0.28920000
H	-3.99799100	1.31411500	0.41797400
C	-3.84517600	4.23809400	-1.31439500
H	-2.92244300	4.48052900	-3.24697900
H	-4.68496700	3.69153400	0.59514300
H	-4.13224700	5.28495500	-1.23099000
C	-3.51863800	-1.87946700	0.47084400
C	-4.52370800	-1.32727000	1.26360200
C	-3.83068100	-2.93259600	-0.38961200
C	-5.82052200	-1.82347300	1.19197600
H	-4.27771200	-0.52280000	1.95496000
C	-5.13122600	-3.41508000	-0.46627500
H	-3.04456000	-3.36833800	-1.00960200
C	-6.14602500	-2.87194200	0.32856300
H	-6.59833400	-1.39069100	1.82186500
H	-5.36650400	-4.23348400	-1.14726300
C	-7.53955800	-3.42263900	0.27487300
H	-8.27270000	-2.69891100	0.65074700
H	-7.82407700	-3.69991400	-0.74784200
H	-7.63044200	-4.32876700	0.89030400

N	-1.81085800	-0.27608400	-0.45011400
C	-2.11948100	-1.34733000	0.50353700
H	-1.39861300	-2.18078000	0.33842100
C	1.26764900	-1.95695200	-1.66885300
C	2.81470300	-4.01132700	-1.07693200
C	3.81521400	-2.05087100	-2.59721300
C	4.63631200	-2.39224200	-0.02375700
C	3.20321900	-0.27457400	-0.72108500
O	5.62765100	-2.54768600	0.54598200
C	1.89762600	1.17606800	3.01510500
C	0.95500100	1.38188200	4.03721500
C	2.78627600	2.20723000	2.66509500
C	0.90505300	2.60164000	4.69363300
H	0.25793700	0.58242500	4.27962500
C	2.73831500	3.41905100	3.33924200
H	3.51109500	2.03831100	1.87060700
C	1.79742900	3.61755000	4.34851700
H	0.16951300	2.76483400	5.47882000
H	3.43306400	4.21380100	3.07564600
O	4.32335000	-1.99740500	-3.63637100
C	1.95011000	-0.06014100	2.32973200
H	1.75831300	4.57124100	4.87189600
O	2.71425600	-5.15576300	-1.18714200
O	0.20407700	-1.88149800	-2.11551100
O	3.37665800	0.85973600	-0.59131400
C	2.07918900	-2.23866300	0.88569000
C	2.02301900	-1.12932200	1.73841800
O	1.48549100	-3.34366500	1.29394700
C	0.83625600	-3.37517800	2.57929600
H	0.38293900	-4.36401300	2.66025900
H	1.57977600	-3.22951700	3.37056900
H	0.07182000	-2.59116400	2.63126500
Cr	2.98985400	-2.14109600	-0.91877200

ts-Ia

E = -2607.251750 G = -2606.783027 (vi = -352.49 cm^{-1})

C	-0.94851900	0.41251400	0.24312300
O	-1.34574400	-1.29328000	1.54596400
N	-0.72805100	-0.03285600	1.45705800
C	-0.41459400	1.69850300	-0.20434100
C	0.18384700	1.80846900	-1.46188000
C	-0.48534500	2.81253200	0.63628100
C	0.71217500	3.01987700	-1.88195200
H	0.24984100	0.93742300	-2.11245900
C	0.03489900	4.03078800	0.22299900
H	-0.95675600	2.72063700	1.61327400
C	0.62675300	4.11796500	-1.03284700
H	1.18578000	3.11437900	-2.85607000
H	-0.01993900	4.90722900	0.86390800
Cl	1.27648400	5.65143300	-1.56146800
C	-2.38629600	-0.15347900	-1.75177700
H	-1.71442900	-0.23662900	-2.61688200
H	-3.12853100	-0.95717900	-1.85399800
C	-3.06284300	1.19387800	-1.72536500
C	-2.76959700	2.14956200	-2.69632400
C	-3.95644800	1.51710300	-0.70189900
C	-3.33820800	3.41868600	-2.63425800
H	-2.06693400	1.90646300	-3.49430300
C	-4.52902400	2.78214000	-0.63951200
H	-4.19750300	0.77053500	0.05646600
C	-4.21485300	3.73905800	-1.60240300
H	-3.08895500	4.16073000	-3.39074100
H	-5.22134500	3.02358200	0.16521500
H	-4.65354800	4.73379200	-1.54660900
C	-3.21521300	-2.20163900	0.35022300
C	-4.12748600	-1.59363800	1.21312400
C	-3.63486400	-3.26525800	-0.44755300
C	-5.44126200	-2.04042900	1.26727200
H	-3.79717500	-0.77607200	1.85416600
C	-4.95336700	-3.70313000	-0.39378400
H	-2.92394600	-3.74959100	-1.11869200
C	-5.87599500	-3.10042800	0.46569800
H	-6.14673600	-1.56328700	1.94781100
H	-5.27383000	-4.53306600	-1.02411200
C	-7.28965200	-3.59333900	0.53925800
H	-7.93302200	-2.88880500	1.07923400

H	-7.71462600	-3.74632800	-0.46095600
H	-7.34639600	-4.55853500	1.06133500
N	-1.65576200	-0.44683100	-0.51736100
C	-1.80731200	-1.69440700	0.25706500
H	-1.13551700	-2.47049900	-0.16335100
C	1.57199200	-1.58552400	-1.94073300
C	3.30312700	-3.52286200	-1.45176300
C	4.19905600	-1.29740600	-2.58313400
C	4.75370900	-1.86868100	0.02675800
C	3.16536900	0.14901800	-0.61798100
O	5.67427100	-1.99025900	0.71400900
C	1.28699100	0.92391000	2.99858000
C	0.25901600	1.33752100	3.85645900
C	2.52331100	1.58568300	3.04367500
C	0.46492200	2.39145900	4.73598700
H	-0.70318600	0.83189500	3.81398800
C	2.72487800	2.63405900	3.93291400
H	3.31910700	1.26696000	2.37241300
C	1.69618400	3.04389200	4.77688700
H	-0.34179900	2.70561000	5.39597300
H	3.68788900	3.14082900	3.95793500
O	4.81116100	-1.05960000	-3.54146500
C	1.15936400	-0.18744900	2.08656600
H	1.85165200	3.87191600	5.46609000
O	3.36701300	-4.64747200	-1.70859400
O	0.53884200	-1.54550400	-2.46687600
O	3.16250800	1.27293800	-0.34490600
C	2.14620900	-2.13903300	0.66500100
C	1.70550800	-1.15490800	1.49718100
O	1.92534000	-3.41438100	0.98139800
C	1.20543100	-3.69536400	2.18776000
H	1.08627600	-4.77962900	2.22784400
H	1.77797300	-3.34361800	3.05491500
H	0.22863400	-3.19577000	2.16881300
Cr	3.21938300	-1.68362200	-1.05592600

Ia

E = -2607.272806   G = -2606.800701

C	-1.12714100	0.13505000	-0.31699400
O	-1.72833300	-1.98305500	0.02046900
N	-0.75334600	-0.98490500	0.26579600
C	-0.41182900	1.40125400	-0.15208300
C	0.09248400	2.05745400	-1.27700000
C	-0.27243700	1.95991500	1.12054000
C	0.74509800	3.27300000	-1.13232400
H	-0.00513600	1.60852700	-2.26325700
C	0.35472900	3.18742300	1.26774900
H	-0.66782300	1.44191200	1.99341800
C	0.85721500	3.82684300	0.13907800
H	1.15777200	3.78932400	-1.99527000
H	0.46421900	3.64194200	2.24899000
Cl	1.65524800	5.36660300	0.32365200
C	-3.03014500	0.95039200	-1.73485100
H	-2.51159900	1.28932900	-2.64180700
H	-3.93269000	0.41804800	-2.06563200
C	-3.39134900	2.11147600	-0.84649900
C	-3.09052600	3.41552700	-1.23436900
C	-4.03077100	1.89100600	0.37541400
C	-3.42098700	4.48901500	-0.41174800
H	-2.58628700	3.59115600	-2.18529700
C	-4.35673000	2.96090200	1.19998900
H	-4.27333400	0.87173100	0.68068900
C	-4.04963500	4.26291100	0.80850800
H	-3.17698500	5.50333800	-0.72206800
H	-4.85348300	2.77984600	2.15152300
H	-4.30215900	5.10091200	1.45552800
C	-3.91281700	-1.90694800	-0.97610700
C	-4.58089600	-1.81642300	0.24575600
C	-4.60920300	-2.31893900	-2.11210200
C	-5.93125500	-2.13042400	0.32125900
H	-4.03634300	-1.51461100	1.14036400
C	-5.96175200	-2.62277700	-2.02987600
H	-4.08782400	-2.39728200	-3.06669900
C	-6.64279300	-2.53526600	-0.81245600
H	-6.44777200	-2.06231600	1.27843400
H	-6.50136200	-2.93811200	-2.92283600

C	-8.09667600	-2.88777300	-0.72734000
H	-8.53684800	-2.55725200	0.22038000
H	-8.66647600	-2.43282500	-1.54737500
H	-8.24372000	-3.97400500	-0.80056400
N	-2.22326600	-0.05991000	-1.04616200
C	-2.47682700	-1.52376500	-1.10208300
H	-2.04487800	-1.91665100	-2.04143100
C	2.80200500	-0.00061700	-2.17323700
C	4.82741900	-1.70320900	-2.09188700
C	5.30045900	0.72420500	-1.23219400
C	4.94352400	-1.38298100	0.54171300
C	3.25070600	0.62314900	0.41042900
O	5.44371600	-1.95139000	1.41782900
C	0.44507200	-1.37136500	2.38635600
C	-0.72661000	-1.10645600	3.10564800
C	1.62433200	-1.63170600	3.09829200
C	-0.71860000	-1.10042400	4.49745200
H	-1.65873600	-0.91232200	2.57274400
C	1.62870500	-1.62350500	4.48547900
H	2.54154800	-1.82995500	2.54066100
C	0.45707200	-1.35842100	5.19460700
H	-1.64140300	-0.89494300	5.03785000
H	2.55624400	-1.82325200	5.01994800
O	6.11753700	1.51846400	-1.47531300
C	0.46869400	-1.38052300	0.91448000
H	0.46304700	-1.35341400	6.28288700
O	5.34017200	-2.40402500	-2.85630000
O	2.00911200	0.26879500	-2.97507400
O	2.82943500	1.34571700	1.21093400
C	2.60992100	-2.08616900	-0.43822700
C	1.47062400	-1.76949600	0.13544500
O	2.94063300	-3.35562600	-0.77572600
C	2.01092100	-4.35248400	-0.37577200
H	2.40197400	-5.31337600	-0.71929700
H	1.90400200	-4.35876700	0.71898400
H	1.02468700	-4.16428100	-0.82608500
Cr	4.01834400	-0.54089100	-0.85604700

IIa

E = -2607.354495 G = -2606.876768

C	1.53615100	-0.51342500	-0.33067200
O	-0.68553400	1.49023400	0.95321200
N	0.67225400	-1.45731700	-0.47277700
C	2.95211400	-0.90939800	-0.55996800
C	3.77921300	-0.14902200	-1.38869100
C	3.43945400	-2.09338300	-0.00321800
C	5.08471400	-0.55073500	-1.64633500
H	3.39824800	0.76159800	-1.85027800
C	4.74699800	-2.49562800	-0.23113700
H	2.78425800	-2.69151000	0.62709500
C	5.55594700	-1.71420300	-1.05054500
H	5.72942800	0.03166000	-2.30030600
H	5.13873700	-3.40645800	0.21574400
Cl	7.20826200	-2.21436500	-1.34537800
C	2.34239700	1.60516500	0.73746900
H	3.06311800	2.09975900	0.07055200
H	1.77465900	2.41096400	1.22402200
C	3.06444500	0.80791500	1.79071100
C	4.45622200	0.84666300	1.87052700
C	2.35663000	0.04556800	2.72214400
C	5.13112800	0.11834100	2.84618300
H	5.01851400	1.43849100	1.14737500
C	3.02707300	-0.68970700	3.69260300
H	1.26785300	0.03070400	2.68801000
C	4.41871900	-0.66002700	3.75360300
H	6.21827900	0.15318800	2.89107100
H	2.45950300	-1.28481900	4.40674000
H	4.94550600	-1.23733700	4.51136600
C	0.22499800	2.90849400	-0.75222700
C	-0.34855900	3.98742800	-0.08166500
C	0.93863900	3.13967900	-1.92827400
C	-0.20327600	5.27499200	-0.58513500
H	-0.91187700	3.82334400	0.83420300
C	1.07871300	4.42984900	-2.42357100
H	1.38356100	2.29963900	-2.46391600
C	0.51054000	5.51990700	-1.76041900

H	-0.65852000	6.11146800	-0.05381000
H	1.63594000	4.59600700	-3.34556200
C	0.65800300	6.91598100	-2.28619100
H	1.22988800	7.54607300	-1.59039800
H	1.17617500	6.93041400	-3.25300900
H	-0.32091200	7.39669400	-2.41931900
N	1.34659600	0.83868600	-0.04666600
C	0.08674300	1.48582600	-0.28261500
H	-0.45905300	0.89663700	-1.02978100
C	-3.76394800	0.83945100	1.99933100
C	-3.96380800	-1.78915200	1.41822000
C	-5.50716700	0.11971500	0.34693400
C	-3.97113800	-1.11788500	-1.20852900
C	-3.31355400	1.34648400	-0.57310800
O	-4.30690600	-1.57549800	-2.21228400
C	-1.31188600	-2.45058900	-1.30630500
C	-1.06504900	-2.36717900	-2.67893800
C	-2.06245600	-3.51937700	-0.81254500
C	-1.60975600	-3.30485400	-3.54796400
H	-0.45275700	-1.54991600	-3.06174900
C	-2.58879800	-4.47170600	-1.67972300
H	-2.21842800	-3.60713800	0.26223200
C	-2.37454300	-4.35866700	-3.05012200
H	-1.43125800	-3.21917700	-4.61869700
H	-3.16740100	-5.30432600	-1.28285500
O	-6.65112900	0.31656600	0.29713300
C	-0.73199300	-1.43915800	-0.38316800
H	-2.79285100	-5.09737300	-3.73181900
O	-4.11365900	-2.76354400	2.02038100
O	-3.88870200	1.49664800	2.94418200
O	-3.09541900	2.29096800	-1.20069800
C	-1.08287900	0.28633800	1.33563900
C	-1.50625800	-0.71062700	0.45175700
O	-1.04979100	0.20662500	2.64388000
C	-1.32545000	-1.04960700	3.28038000
H	-2.38745100	-1.09743600	3.53953500
H	-1.05071100	-1.88547200	2.62885000
H	-0.72033100	-1.05384100	4.18908400
Cr	-3.70605500	-0.19808400	0.43916900

tsIV-a

E = -2607.310996 G = -2606.834868 (ν_i = -402.27 cm^{-1})

C	1.05668200	-0.93032900	-0.28082700
O	-0.55534500	1.93429100	0.55843600
N	0.09643000	-1.64395000	-0.72584500
C	2.44913400	-1.26500700	-0.65057800
C	3.35229800	-0.29816100	-1.09869800
C	2.85568200	-2.60137200	-0.58753500
C	4.64873200	-0.64947200	-1.45361400
H	3.04902300	0.74450300	-1.19073600
C	4.15045100	-2.96544800	-0.92575800
H	2.14684500	-3.35275600	-0.24523400
C	5.03588200	-1.97985800	-1.35022000
H	5.35075200	0.09951600	-1.81208500
H	4.47429000	-4.00142500	-0.85951400
Cl	6.67407900	-2.42687900	-1.77285800
C	1.87215800	0.59021100	1.51087400
H	2.64419600	1.18103700	0.99960100
H	1.38918400	1.25695300	2.23300000
C	2.50534500	-0.58189600	2.21249400
C	3.88482000	-0.57561800	2.42171200
C	1.75962900	-1.67276600	2.66266100
C	4.50938500	-1.63487900	3.07096200
H	4.47820100	0.26295800	2.05559900
C	2.38290300	-2.73692400	3.30605500
H	0.68693900	-1.71557400	2.47967300
C	3.75923100	-2.72064600	3.51412700
H	5.58760700	-1.61708100	3.21902400
H	1.78899000	-3.58558900	3.64059300
H	4.24704600	-3.55539000	4.01406800
C	1.25229700	2.91433800	-0.65222300
C	1.55841500	3.81692700	0.38501400
C	1.89622300	3.03788100	-1.89835600
C	2.48274100	4.81910200	0.16680100
H	1.05681300	3.71863900	1.34575600
C	2.82490500	4.04043400	-2.09874400

H	1.65078300	2.34277800	-2.70145500
C	3.12865700	4.94747000	-1.07290800
H	2.72010500	5.52157900	0.96426500
H	3.32228900	4.13835000	-3.06223100
C	4.10720700	6.05216700	-1.30172600
H	4.67483700	6.28216100	-0.39255800
H	4.81027100	5.81114800	-2.10655400
H	3.57924300	6.97162600	-1.59208500
N	0.83268400	0.22923400	0.50913300
C	0.30152300	1.86866100	-0.46929000
H	0.00225400	1.28969300	-1.35193300
C	-3.24251200	1.91549100	1.55513800
C	-4.27167800	-0.55784300	1.41119800
C	-5.29940200	1.58018000	0.12276700
C	-4.35360900	-0.32299100	-1.25277300
C	-2.90766300	1.86153300	-1.10127500
O	-4.89541500	-0.84593000	-2.13163800
C	-2.14926700	-2.29285100	-1.15274600
C	-2.01133900	-2.55062300	-2.52017800
C	-3.09903300	-3.01490000	-0.42607000
C	-2.84583600	-3.46100400	-3.15733800
H	-1.24649300	-2.01654700	-3.08380700
C	-3.92434100	-3.93979800	-1.05778900
H	-3.18604400	-2.85295100	0.64722500
C	-3.80995200	-4.15433000	-2.42812000
H	-2.74147000	-3.63515000	-4.22737400
H	-4.65857100	-4.49565100	-0.47645900
O	-6.32617400	2.13184200	0.09821600
C	-1.25433700	-1.31110700	-0.48829600
H	-4.46055500	-4.87178600	-2.92569900
O	-4.63360500	-1.35168800	2.17401800
O	-3.07026900	2.69640700	2.39382100
O	-2.41750900	2.53994600	-1.90311800
C	-0.62982200	0.53192800	0.94003500
C	-1.69235200	-0.21873300	0.19827500
O	-0.72918800	0.55895400	2.29589800
C	-1.45627700	-0.43447000	3.01407200
H	-2.42299800	-0.02871900	3.32904700
H	-1.62151400	-1.33622400	2.41409500
H	-0.85974400	-0.68087100	3.89744100
Cr	-3.68194500	0.72259800	0.17317100

IVa

E = -2607.385419 G = -2606.909469

C	0.68496000	-0.95557100	-0.21697500
O	-0.16342300	3.08181900	0.20097700
N	-0.29394600	-1.58335400	-0.80962900
C	2.07170300	-1.22222800	-0.64807800
C	2.93765100	-0.19011300	-1.01393300
C	2.50884300	-2.54760100	-0.71635000
C	4.23647300	-0.47043700	-1.41654000
H	2.59523600	0.84401000	-1.00801400
C	3.80884800	-2.84051300	-1.10391700
H	1.82701200	-3.34919100	-0.43773400
C	4.66012700	-1.79419900	-1.44414000
H	4.90873700	0.33191900	-1.71485200
H	4.16181700	-3.86804800	-1.14071200
Cl	6.30089200	-2.15344700	-1.92975700
C	1.53360100	0.24997100	1.77195200
H	2.25309700	0.87958100	1.23964700
H	1.07838600	0.87628900	2.54316100
C	2.21000400	-0.96210600	2.34908000
C	3.60101500	-0.98932100	2.43885000
C	1.47292200	-2.06170200	2.79240500
C	4.25063600	-2.10001600	2.96769200
H	4.17867200	-0.13850200	2.07279500
C	2.12207600	-3.17645300	3.31202400
H	0.38373800	-2.06023900	2.71258600
C	3.51203100	-3.19797900	3.40007000
H	5.33732100	-2.11407100	3.02640400
H	1.53989000	-4.03324700	3.64610500
H	4.01881900	-4.07300500	3.80268600
C	1.99874800	3.32361400	-0.76165100
C	2.70316100	3.30128600	0.45027300
C	2.70240100	3.31996200	-1.96752400
C	4.08546800	3.24125400	0.44603000

H	2.14298900	3.32390100	1.38466800
C	4.09100400	3.25664800	-1.96547300
H	2.15242300	3.34114100	-2.90898300
C	4.80005900	3.20765700	-0.76240400
H	4.63512700	3.21751500	1.38753300
H	4.63807300	3.23724000	-2.90777100
C	6.29428200	3.11609500	-0.74850100
H	6.63020800	2.26203600	-0.14491600
H	6.70262900	3.00112100	-1.75900700
H	6.74198500	4.01473800	-0.30309000
N	0.43805800	-0.11781400	0.83939400
C	0.53256600	3.26061500	-0.78132900
H	0.07447000	3.35539500	-1.79239100
C	-3.15699200	2.39607700	1.20782100
C	-4.55462500	0.08067800	1.45189300
C	-5.15340300	2.14706100	-0.26203000
C	-4.58119000	-0.08826800	-1.21385900
C	-2.66795200	1.70216900	-1.33766900
O	-5.20382200	-0.63776900	-2.02169600
C	-2.55563200	-2.24147300	-1.04044300
C	-2.45504200	-2.66849000	-2.36780900
C	-3.55781100	-2.77376600	-0.22148000
C	-3.37519600	-3.57312100	-2.88249600
H	-1.65972000	-2.26923100	-2.99507100
C	-4.45653500	-3.70292100	-0.72900800
H	-3.61551200	-2.46468200	0.82151800
C	-4.37592700	-4.09278700	-2.06397300
H	-3.30794300	-3.88116600	-3.92431600
H	-5.22453700	-4.12227900	-0.08164900
O	-6.05202600	2.86336700	-0.45539500
C	-1.57516900	-1.29386900	-0.48069700
H	-5.08958000	-4.81023400	-2.46550300
O	-5.02368500	-0.54170100	2.30941100
O	-2.94154800	3.31440600	1.87839900
O	-1.98914700	2.04470200	-2.21049300
C	-0.86487400	0.24872100	1.09462700
C	-1.94590500	-0.19516800	0.32598000
O	-0.99526500	1.06346200	2.14333200
C	-1.69740800	0.55261800	3.28248600
H	-2.27509500	1.37655700	3.70862700
H	-2.36558000	-0.26572600	2.99491100
H	-0.96580500	0.19230700	4.01709800
Cr	-3.73912500	1.02535000	0.04992000

Water
E = -76.380526 G = -76.376700

IVa-w
E = -2375.551895 G = -2375.160032

C	-1.38821500	0.12145300	-0.27434100
N	-0.59253500	1.13036800	-0.49445800
C	-2.73958000	0.11350200	-0.84421000
C	-3.24857200	-1.04876600	-1.43153500
C	-3.49636100	1.28745300	-0.85687800
C	-4.51260300	-1.05105900	-2.00337200
H	-2.64450300	-1.95527500	-1.45963100
C	-4.76942200	1.29223800	-1.40693600
H	-3.08740100	2.18916800	-0.40531300
C	-5.26319900	0.11926100	-1.97032100
H	-4.91389000	-1.94755100	-2.46960600
H	-5.37724400	2.19375400	-1.40165700
Cl	-6.86751600	0.11968700	-2.66243000
C	-1.84874400	-1.68695100	1.40635900
H	-2.18910300	-2.62314500	0.94786400
H	-1.19528500	-1.92966400	2.25341300
C	-3.01672100	-0.86201600	1.87315600
C	-4.31786500	-1.32559600	1.68135900
C	-2.81259900	0.36416900	2.51322300
C	-5.40636200	-0.56872300	2.10439800
H	-4.48082700	-2.27969300	1.17878500
C	-3.90127200	1.11968300	2.93386300
H	-1.79399600	0.72093600	2.66972000
C	-5.19985900	0.65793200	2.72802300
H	-6.41734000	-0.93740700	1.93968100

H	-3.73519600	2.07668400	3.42601700
H	-6.05037700	1.25469100	3.05315900
N	-0.94873100	-0.95278000	0.45902500
C	2.20492700	-1.78037200	-1.82909400
C	3.54590000	-2.26135200	0.41496900
C	4.81712700	-1.32928800	-1.62275900
C	4.40856200	0.29494400	0.40097500
C	3.20897500	0.67573300	-1.92888800
O	5.12145000	0.93019400	1.05793900
C	1.38183600	2.35633100	-0.02790700
C	2.31093500	2.57586700	0.99439600
C	1.04472300	3.41166700	-0.88359000
C	2.91294200	3.81788800	1.14426700
H	2.52456200	1.76961800	1.69433300
C	1.65925000	4.64891700	-0.74314900
H	0.31002100	3.24356500	-1.66857600
C	2.59527900	4.85422000	0.26904700
H	3.62758600	3.97963500	1.94921200
H	1.40616100	5.45946600	-1.42392600
O	5.75722400	-1.69548400	-2.20311300
C	0.70677800	1.05455800	-0.12421700
H	3.07088100	5.82690500	0.38189500
O	3.74517200	-3.21938800	1.03127100
O	1.48211900	-2.39587200	-2.48976100
O	3.19264200	1.52470200	-2.71306400
C	0.41533500	-1.14440100	0.51964500
C	1.34569500	-0.18347900	0.12381400
O	0.82120600	-2.29868300	1.04827800
C	0.33809700	-3.54328100	0.52936700
H	1.21726300	-4.17669300	0.37614800
H	-0.33256300	-4.01732300	1.25287800
H	-0.17787700	-3.39826300	-0.42724000
Cr	3.33017100	-0.76365500	-0.71571600
O	2.83095800	-0.53446500	2.94154800
H	2.72267300	-0.49329200	1.96996100
H	3.53559300	0.09688500	3.14354900
O	0.14880700	0.01234200	3.33229400
H	-0.16881000	-0.32818500	4.17830900
H	1.11568100	-0.14486500	3.34641900

ts-Va

E = -2375.509701 G = -2,375.122607 (ν_i = -420.70 cm^{-1})

C	-1.15204800	-0.08359400	-0.15408900
N	-0.33384700	0.56437400	-0.92630700
C	-2.45738300	-0.50560200	-0.67103600
C	-2.97349500	-1.77271500	-0.38022900
C	-3.17785000	0.37120600	-1.48830000
C	-4.20646200	-2.15733200	-0.88638200
H	-2.40296700	-2.47527900	0.22674400
C	-4.42138900	0.00547200	-1.97903100
H	-2.76797000	1.35577300	-1.70505800
C	-4.92225800	-1.25560000	-1.66845500
H	-4.61208400	-3.14441500	-0.67966000
H	-5.00294000	0.68833100	-2.59330600
Cl	-6.48914900	-1.72092100	-2.27761100
C	-1.90022700	-0.34150200	2.23751900
H	-2.38201800	-1.31891900	2.34900800
H	-1.34469200	-0.11514500	3.15346900
C	-2.90111700	0.74697300	1.96851800
C	-4.26161300	0.44754100	1.90897000
C	-2.46758400	2.06589400	1.79701900
C	-5.19013200	1.45436300	1.66376700
H	-4.59485700	-0.58253700	2.04137200
C	-3.39976700	3.06577400	1.54093600
H	-1.39961200	2.29313500	1.88907600
C	-4.75875200	2.76363300	1.47058800
H	-6.25047100	1.21278200	1.61560200
H	-3.06407000	4.09256100	1.40453000
H	-5.48346600	3.55126100	1.27117100
N	-0.84424800	-0.40209300	1.16650200
C	1.47444300	-2.53607800	-0.33351800
C	3.36137500	-1.76587700	1.45913900
C	4.04742600	-2.55495700	-0.89906700
C	4.26962400	0.01929100	-0.30355500
C	2.50388900	-0.88120500	-2.12710200
O	5.13301900	0.78019000	-0.30594100

C	1.55276100	2.03222500	-1.14001300
C	2.51957400	2.80473100	-0.47890900
C	1.20352500	2.33714600	-2.46359000
C	3.14545000	3.84600000	-1.14892000
H	2.75078000	2.59287500	0.56705800
C	1.84099300	3.37393000	-3.12881400
H	0.43959000	1.74427500	-2.96153500
C	2.81588400	4.12520800	-2.47449500
H	3.89249700	4.44713700	-0.63488700
H	1.57930800	3.59752800	-4.16102300
O	4.79358300	-3.36698600	-1.26364900
C	0.87939800	0.94744900	-0.43094500
H	3.31656200	4.93739800	-2.99855500
O	3.70453400	-2.10823300	2.50409300
O	0.60767000	-3.30249600	-0.35034000
O	2.32171000	-0.69474000	-3.25136200
C	0.46783100	-0.33012500	1.53377500
C	1.40779200	0.31676700	0.70226200
O	0.87546700	-0.93296000	2.61937800
C	0.30210300	-2.17996100	3.05077300
H	1.14671300	-2.78515200	3.38776600
H	-0.38799100	-2.01509500	3.88209700
H	-0.20524800	-2.68268500	2.21922100
Cr	2.87854900	-1.28711700	-0.31884000
O	2.92663000	1.55093000	2.67882500
H	2.28777500	0.71433800	1.26608700
H	3.38211900	2.39469000	2.56839000
O	0.52000900	1.90348800	2.75211000
H	0.24219200	1.91234800	3.67733800
H	1.66495800	1.80739700	2.77946900

VaA
E = -2375.572503 G = -2375.175784

C	1.05024900	-0.05597000	0.03831800
N	0.20208800	0.38810500	0.92062500
C	2.34382200	-0.56422100	0.54624500
C	2.85009800	-1.80237300	0.14450700
C	3.05370500	0.20662400	1.46949400
C	4.06533100	-2.25858600	0.63921900
H	2.28645900	-2.42750700	-0.54733000
C	4.27787000	-0.22782500	1.95641000
H	2.64938700	1.16881500	1.77814500
C	4.77089200	-1.45609600	1.52871400
H	4.46183400	-3.22555700	0.33922500
H	4.84761800	0.37720300	2.65740100
Cl	6.31635600	-2.01055300	2.13225500
C	2.00623800	0.02249500	-2.22991200
H	2.49951700	-0.95400100	-2.30869000
H	1.59914000	0.23227300	-3.22572200
C	3.00562000	1.07820500	-1.83281100
C	4.36676900	0.77577000	-1.84528600
C	2.59264700	2.35303200	-1.43742300
C	5.30641500	1.72803100	-1.46171000
H	4.69085600	-0.22654200	-2.12964000
C	3.53121500	3.30202200	-1.04588500
H	1.52895600	2.59394600	-1.43034500
C	4.88948400	2.99208000	-1.05501600
H	6.36539600	1.47543800	-1.46590000
H	3.19953200	4.28981200	-0.72990800
H	5.62137900	3.73442500	-0.74130100
N	0.84675500	-0.06894000	-1.31617000
C	-1.28203400	-2.46776400	0.19008900
C	-3.17466600	-1.59895200	-1.64938900
C	-3.85012900	-2.68438500	0.56428500
C	-4.21649500	-0.08880700	0.27319100
C	-2.46910700	-1.08461700	2.03158500
O	-5.13431100	0.60420600	0.34424300
C	-1.69703900	1.80875000	1.34729900
C	-2.63084200	2.71605100	0.82773500
C	-1.33661300	1.90464100	2.69760000
C	-3.21198900	3.67208900	1.65075900
H	-2.89306200	2.69066000	-0.23035100
C	-1.92212700	2.85973600	3.51769900
H	-0.59195600	1.21684900	3.09141300
C	-2.86473300	3.74354000	2.99803100
H	-3.93238000	4.37417800	1.23513900

H	-1.63913200	2.91659400	4.56715100
O	-4.55114900	-3.57949200	0.80146500
C	-1.02045600	0.83099400	0.47082100
H	-3.32088200	4.49495700	3.64004000
O	-3.53199200	-1.83100600	-2.71998200
O	-0.41690000	-3.23094700	0.27664000
O	-2.34613700	-1.04432600	3.17906000
C	-0.45140700	0.29606900	-1.87454200
C	-1.48443800	0.47419300	-0.79002700
O	-0.82418800	-0.54809500	-2.92780500
C	-0.32246700	-1.86972300	-3.02454700
H	-1.06243300	-2.44007900	-3.59356300
H	0.63295000	-1.89383500	-3.56683100
H	-0.18569700	-2.33997400	-2.04547500
Cr	-2.74402600	-1.28586300	0.17668400
O	-3.32344200	2.01034700	-2.79117100
H	-2.35178300	0.99526100	-1.18336600
H	-3.68507000	2.90610400	-2.81936900
O	-0.38382400	1.57610100	-2.48837500
H	-0.18310300	1.42920700	-3.42932400
H	-2.36020900	2.13339600	-2.84667600

VaB

E = -2375.578874 G = -2375.181443

C	0.91846800	-0.11417000	-0.05637300
N	0.05628600	-1.05564300	0.20218700
C	2.16822800	-0.52748200	-0.73441100
C	2.66948900	0.18181800	-1.82786500
C	2.83669600	-1.66998800	-0.28794400
C	3.83894100	-0.22547300	-2.45641300
H	2.13106600	1.05028300	-2.20343900
C	4.01567400	-2.07871300	-0.89436000
H	2.43308300	-2.22307200	0.55774100
C	4.50436400	-1.34497000	-1.97038200
H	4.22994600	0.31536800	-3.31473100
H	4.55343300	-2.95485200	-0.53999400
Cl	5.99182600	-1.85130600	-2.74156400
C	1.97743800	2.01264000	0.57121700
H	2.46193500	2.33523500	-0.35890100
H	1.62485500	2.92451200	1.06611900
C	2.96794300	1.29345500	1.45045100
C	4.32414900	1.31284300	1.12607700
C	2.54905900	0.59494000	2.58555400
C	5.25013500	0.63357300	1.91213700
H	4.65608100	1.85161000	0.23764700
C	3.47331400	-0.08803500	3.36879900
H	1.49039800	0.58978000	2.84523700
C	4.82532000	-0.07488800	3.03221000
H	6.30449100	0.64841100	1.64086200
H	3.13623800	-0.63572100	4.24755500
H	5.54585900	-0.61696200	3.64231700
N	0.77017100	1.20849800	0.26189400
C	-1.33883400	0.58310500	-2.21982800
C	-3.30357600	1.99891300	-0.95305200
C	-3.89984900	0.17067100	-2.62109800
C	-4.30082500	-0.31964700	-0.06168200
C	-2.48886400	-1.65162700	-1.50723600
O	-5.22488800	-0.61797000	0.56072900
C	-1.82213500	-1.80516500	1.51086300
C	-2.76747100	-1.54950500	2.51249000
C	-1.48012500	-3.13547400	1.23437700
C	-3.37774900	-2.59408900	3.19401800
H	-3.02064300	-0.52522400	2.78066000
C	-2.09235800	-4.17933900	1.91586300
H	-0.73126600	-3.33741700	0.47260700
C	-3.04693900	-3.91348000	2.89391600
H	-4.10829800	-2.37637800	3.97077100
H	-1.82133500	-5.20742200	1.68229300
O	-4.58171400	0.17999100	-3.56172400
C	-1.13153800	-0.70383500	0.80219900
H	-3.52610100	-4.73182100	3.42834500
O	-3.69269200	3.08337000	-0.91597500
O	-0.44114700	0.78559000	-2.91996800
O	-2.32848600	-2.75334800	-1.81401100
C	-0.49237400	1.70250100	0.82550300
C	-1.55198100	0.62047400	0.82055300

O	-0.94841400	2.86567000	0.17008800
C	-0.24495600	3.47293700	-0.89506000
H	-0.98883800	4.02450900	-1.48052500
H	0.50893200	4.18355200	-0.52645100
H	0.23929200	2.74032800	-1.55016300
Cr	-2.82521700	0.16798500	-1.14687000
O	0.08867700	4.74727000	2.11513500
H	-2.37486700	0.90155700	1.47516400
H	0.00394100	5.25140000	2.93706500
O	-0.31910300	2.03094600	2.16703200
H	-0.18431100	3.00757200	2.23896900
H	-0.70066300	4.97124200	1.60017600

ts-5a

E = -2375.540781 G = -2375.147973 (ν_i = -1112.85 cm^{-1})

C	-0.93850500	0.07473300	-0.05928000
N	-0.07820700	1.04342300	0.07644300
C	-2.19508500	0.40040000	-0.77156900
C	-2.69164100	-0.41377200	-1.79157500
C	-2.87939700	1.56863000	-0.42740300
C	-3.87119400	-0.08257000	-2.44629600
H	-2.14178800	-1.30405400	-2.09437200
C	-4.06798700	1.90238300	-1.05977800
H	-2.48281500	2.20328500	0.36266600
C	-4.55221700	1.06654400	-2.06044200
H	-4.25824800	-0.70624500	-3.24846600
H	-4.61741000	2.79844000	-0.78126600
Cl	-6.05098300	1.47754200	-2.86444900
C	-1.96810500	-1.99140000	0.81280200
H	-2.42129200	-2.44419300	-0.07766800
H	-1.58154600	-2.81203600	1.42806800
C	-2.99463700	-1.21330600	1.59187600
C	-4.34077300	-1.27976000	1.23472300
C	-2.61216300	-0.41672200	2.67450000
C	-5.29677900	-0.55737200	1.94285000
H	-4.63865200	-1.88182900	0.37453100
C	-3.56607700	0.31240500	3.37594600
H	-1.55868400	-0.37853400	2.95544700
C	-4.90963700	0.24577000	3.01154500
H	-6.34390200	-0.61101500	1.64939800
H	-3.26023000	0.93530500	4.21521500
H	-5.65376400	0.82034600	3.56066200
N	-0.78152800	-1.19464600	0.42612000
C	1.33389200	-0.72907300	-2.16676800
C	3.28457400	-2.06421200	-0.74182600
C	3.90428800	-0.42059500	-2.57713400
C	4.29660100	0.31123500	-0.07340100
C	2.51978000	1.52442200	-1.64502500
O	5.21898200	0.66343300	0.52226600
C	1.78273900	1.97421500	1.28707300
C	2.74117600	1.86378500	2.30339700
C	1.39340500	3.25432600	0.87120100
C	3.31643900	2.99817100	2.86092400
H	3.03373100	0.88587200	2.68191000
C	1.96755800	4.38782400	1.43113900
H	0.63405100	3.34685900	0.09916100
C	2.93541000	4.26478700	2.42446700
H	4.05810900	2.89092900	3.65026600
H	1.65497000	5.37335800	1.09073300
O	4.58994600	-0.53231600	-3.50779100
C	1.11812300	0.78309800	0.70895100
H	3.38430700	5.15306000	2.86537200
O	3.66280000	-3.14256500	-0.58594000
O	0.43141900	-0.95261500	-2.85440300
O	2.38358000	2.59598800	-2.05180000
C	0.45338100	-1.54713900	1.13227400
C	1.54425200	-0.52842700	0.90332000
O	0.93649700	-2.92739200	0.47788000
C	0.33193000	-3.51965200	-0.66812100
H	1.12165700	-4.01213200	-1.24575300
H	-0.41950000	-4.26206600	-0.36606200
H	-0.14437300	-2.75663100	-1.28739600
Cr	2.82172800	-0.26273000	-1.11729400
O	0.65331200	-4.16115900	2.47522900
H	2.36927400	-0.71571300	1.58844000
H	1.45857300	-4.44051700	2.94153000

O	0.29441400	-1.79214600	2.40117500
H	0.44379700	-3.12591900	2.73184700
H	0.92340100	-3.73288900	1.41531600

5a

E = -2375.610258 G = -2,375.212807

C	-0.85253700	0.45255700	0.06011300
N	0.04505500	1.39659100	-0.02367600
C	-2.11346900	0.65629800	-0.68615700
C	-2.58416800	-0.28675300	-1.60210300
C	-2.81947200	1.84483000	-0.48647900
C	-3.76274500	-0.05728600	-2.29962700
H	-2.01138100	-1.19289600	-1.79448800
C	-4.00924700	2.07558900	-1.16185400
H	-2.43905500	2.58029300	0.22012000
C	-4.46767200	1.11700100	-2.05915300
H	-4.13127800	-0.77985300	-3.02374300
H	-4.57620800	2.98845900	-0.99709800
Cl	-5.96430200	1.40235300	-2.91582400
C	-1.86243000	-1.54088100	1.15837600
H	-2.22760100	-2.03892300	0.25606000
H	-1.45229100	-2.32342300	1.80419200
C	-2.95369900	-0.77215500	1.85075200
C	-4.28098400	-0.92851200	1.45404500
C	-2.65287200	0.10714700	2.89322600
C	-5.29424500	-0.20809700	2.07988300
H	-4.51864900	-1.60844400	0.63491300
C	-3.66270500	0.83458400	3.51344300
H	-1.61821900	0.22904600	3.21866000
C	-4.98576300	0.68164200	3.10468800
H	-6.32584100	-0.33251700	1.75508800
H	-3.41640400	1.52219800	4.32068500
H	-5.77581600	1.25382900	3.58778500
N	-0.70840000	-0.68186200	0.81793700
C	1.24496800	-1.15854600	-1.71359300
C	2.85521200	-2.32657900	0.16367100
C	3.83107100	-1.42426200	-2.02737700
C	4.36147500	-0.07531300	0.14759900
C	2.80934000	0.92690200	-1.77515600
O	5.33803200	0.27055200	0.65483900
C	2.03785400	2.47657200	0.78932300
C	3.09411900	2.55479200	1.70872500
C	1.72240800	3.60807200	0.02527400
C	3.82199600	3.72745800	1.84835300
H	3.35328100	1.70017700	2.33106000
C	2.45403300	4.77971200	0.16543500
H	0.89869900	3.55407300	-0.68206700
C	3.50582600	4.84308500	1.07567700
H	4.63581100	3.77345900	2.56912900
H	2.19995800	5.64871500	-0.43826200
O	4.49154400	-1.92031900	-2.84273600
C	1.22796900	1.25294500	0.64856900
H	4.07695800	5.76281800	1.18722000
O	2.93884700	-3.36209800	0.66204700
O	0.35109700	-1.38884900	-2.40367700
O	2.85671600	1.84419300	-2.47381400
C	0.50619500	-0.92817400	1.49045300
C	1.55818600	0.03408400	1.24301900
O	-0.00606500	-3.47393300	-0.42321500
C	-1.01389900	-4.23144900	-1.03566500
H	-0.65586100	-5.21274100	-1.38970100
H	-1.87445200	-4.40882500	-0.36661400
H	-1.37484300	-3.67568300	-1.91136900
Cr	2.79548400	-0.65525800	-0.74161600
O	0.35457300	-4.61717900	2.07106600
H	2.38859100	-0.05297900	1.93729600
H	1.23339400	-5.01451500	2.14497300
O	0.62031000	-1.86026800	2.27902100
H	0.47230100	-3.70674100	2.40335000
H	0.23497300	-3.93082300	0.41175300

4. ^{1}H NMR mechanistic studies

In order to gather evidence supporting any of the possible mechanisms, a reaction was conducted in a Schlenk flask using the model substrates **1a** and **4a** and monitored by NMR. The reaction took place at room temperature in 1,2-dichloroethane, following the standard procedure described below. After one day, a sample was transferred to an NMR tube and a capillary tube of D_2O was introduced to record a ^{1}H NMR spectrum with solvent suppression.

Two approaches were taken to monitor the reaction. Firstly, the initial NMR sample was left under the reaction conditions and monitored daily. Secondly, a fresh sample was taken from the reaction flask every day and a new ^{1}H NMR spectrum was recorded using a new NMR tube and a capillary tube of D_2O. The second approach provided cleaner and more significant spectra than the first one. Therefore, these spectra will be used for further analysis discussion (Figure S1).

The piled spectra were used for monitoring over a 3-day period to track the formation of the final product. The red mark indicates the signals that correspond to 4,5-dihydro-1,2,4-oxadiazole **4a**. The green mark represents the extruded *p*-tolualdehyde, while the purple mark indicates the final product **5a**. The blue mark represents the signals of only possible intermediate detected (Scheme S4). There are two regions of the spectra that provide significant information in this study. The first region corresponds to the aromatic methyl groups between 2.0-2.4 ppm (*p*-Tol region), and the second region (benzyl region) corresponds to benzyl protons around 4.0-4.5 ppm.

Scheme S4. Compounds detected in the ^{1}H NMR monitorization

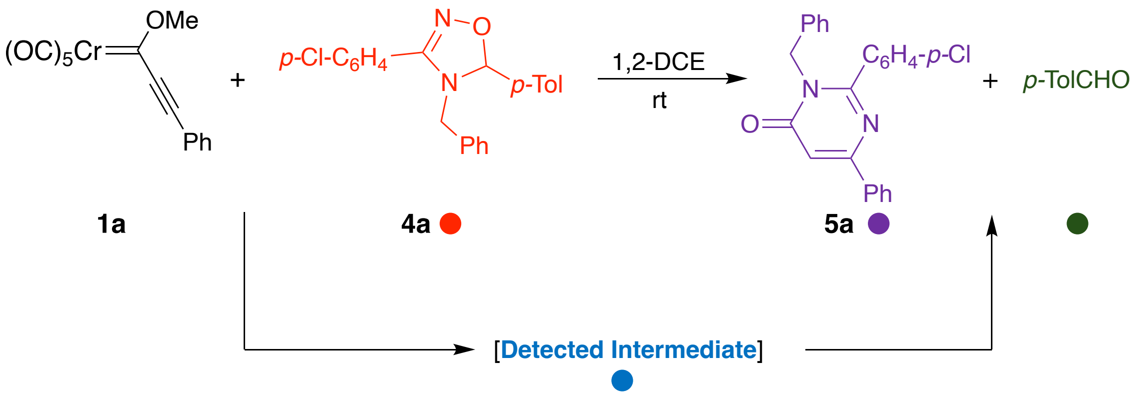

Figure S1: ^{1}H NMR spectra (600 MHz) with solvent suppression piled at 1 to 3 days and the final product **5a**.

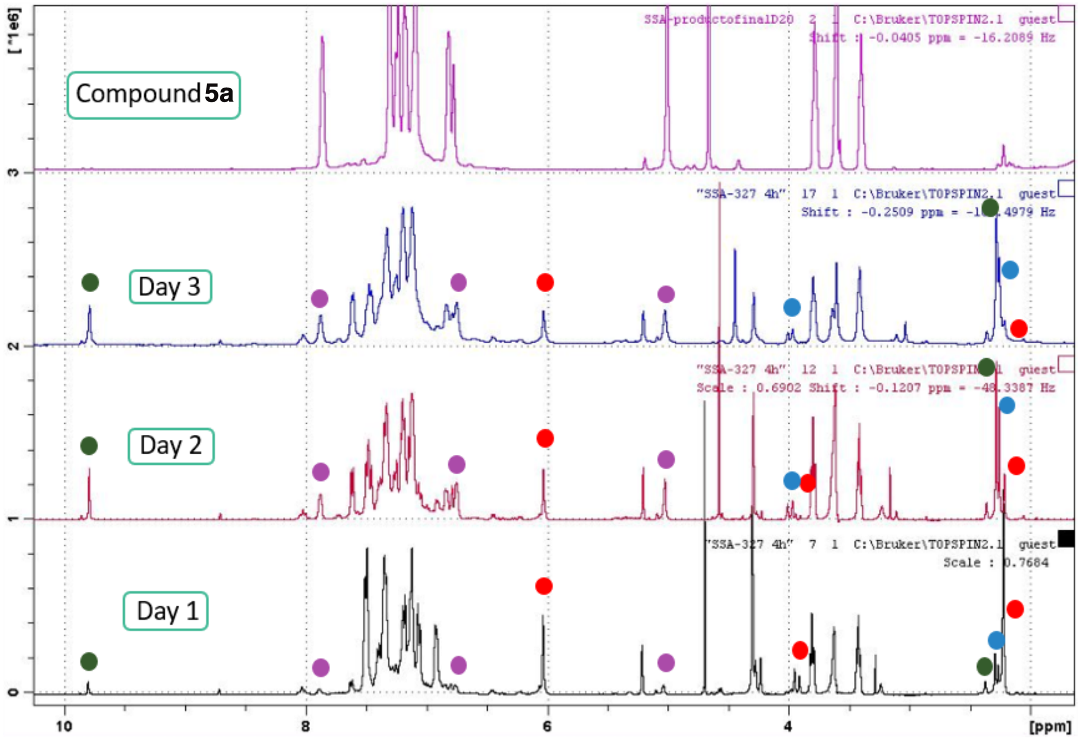

On the first day, three signals on the *p*-Tol region of the that correspond to the 4,5-dihydro-1,2,4-oxadiazole **4a**, to the possible intermediate and to the extruded *p*-tolualdehyde are observed. Moreover, one signal around 4 ppm that corresponds to the benzylic protons and one signal around 6 ppm that is assigned to the CH proton, both belonging to the 4,5-dihydro-1,2,4-oxadiazole **4a**, are observed. In addition, different signals that correspond to 3,4-dihydropyrimidin-4-one **5a** also appear. Finally, a singlet around 10 ppm that correspond to the proton of the extruded *p*-tolualdehyde is detected.

On the second day, the signals for *p*-tolualdehyde, the intermediate, and the final product increase, while the signals for the starting material decrease, indicating the expected progression of the reaction.

By day 3, the signal for the intermediate do not increase at the same rate as before. In fact, the intensity of the intermediate signal is much smaller than that of *p*-tolualdehyde, unlike on day 2, where they are similar. This suggests that the intermediate is evolving into the final product.

Figure S2: Expansions of selected signals (Days 1, 3, 7) in the ^{1}H NMR spectra (600 MHz).

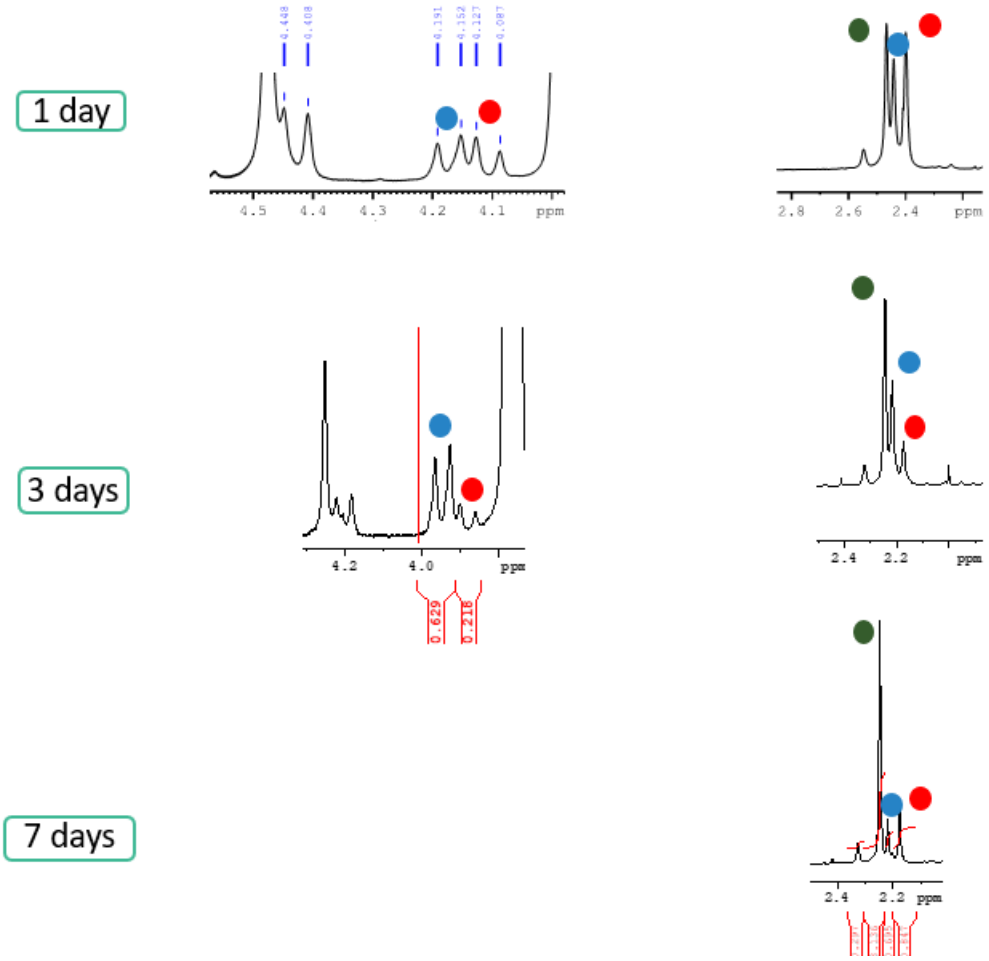

Going in detail to the regions mentioned before, in the 4,5-dihydro-1,2,4-oxadiazole and the release *p*-tolualdehyde, there is an aromatic methyl group, which is absent in the final product. Hence, in the *p*-Tol region (Figure S2, right) in addition to the signals corresponding to these two species (red and green marks, Figure S2), a new signal (blue mark) that appears initially (day 1), grows (days 1-3) and then decreases gradually (day 3-7) is observed.

Regarding the benzyl region (Figure S2, left), those H atoms appear as an AB quartet in the starting material due to their diastereotopicity because of the presence of a stereocenter. However, in the final product, they appear as a singlet because there is no stereocenter. It is important to note that a new signal (blue mark), which also appears as an AB quartet, is observed in that region. This signal can be assigned to the detected reaction intermediate, as it follows the same trend as the previously described methyl group.

Based on these findings, we conclude that the observed reaction intermediate should contain diastereotopic benzyl protons and an aromatic methyl group. Therefore, allenyl chromate **Ia** or eight-membered heterocyclic chromate **IIa**, drawn in blue in Scheme S5, appear as tentative options. Initially, we considered allenyl chromate **Ia** as the most probable structure for the proposed observed intermediate. However, it could not be confirmed as no representative signal for the allenyl carbon could be observed in the ^{13}C NMR spectra.

Scheme S5. Tentative structures (**Ia** and **IIa**) for the intermediate detected in the ^{1}H NMR monitorization

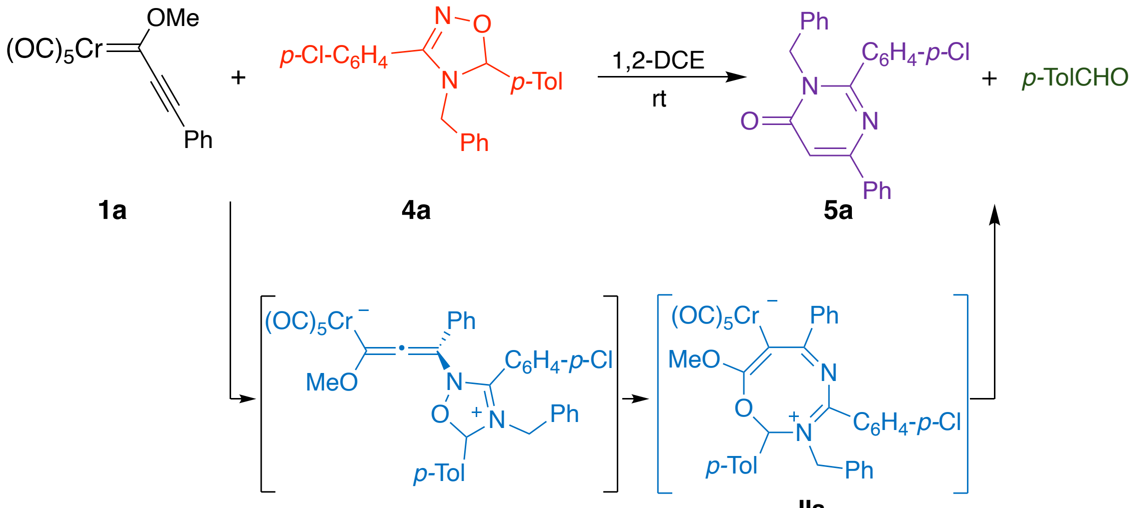

In addition, after getting the results from the DFT calculations and considering the high stability of intermediate **IIa** (See Scheme S3), eight-membered heterocyclic chromate **IIa** appeared as the most probable option. However, no confirmation could be reached by ^{13}C NMR. Also, no other intermediate was observed in the NMR experiments.

In conclusion from these NMR experiments, a reaction intermediate displaying diastereotopic benzyl H atoms was detected and, although attempts were made to elucidate its structure, all data collected were inconclusive, and its identity could not be unequivocally established.

5. Experimental Section

General Methods. All reactions involving air sensitive compounds were carried out under inert atmosphere (Ar or N_2, ≥ 99.99%). All glassware was oven-dried (120 °C),

evacuated and purged with nitrogen or argon. Reactions that require heating were carried out in oil baths placed over magnetic stirring plates equipped with temperature controllers. All common reagents and solvents were obtained from commercial suppliers and used without any further purification unless otherwise indicated. Fischer alkoxy alkynyl metal carbene complexes **1**, [6] **2**[6] and **6**,[6] (*Z*)-*N'*-hydroxy-4-methoxybenzimidamide[7] and 4,5-dihydro-1,2,4-oxadiazoles **5i**[8] and **9a**[9] have have been previously synthesized and were prepared as described. Methyl phenylpropiolate (methyl 3-phenylpropynoate) **3** was obtained from commercial suppies and used without any further purification. Solvents were dried by standard methods.[10] Hexane (HxH), ethyl acetate and triethylamine were purchased as extra pure grade reagents and used as received. TLC was performed on aluminium-backed plates coated with silica gel 60 with F_{254} indicator; the chromatograms were visualized under ultraviolet light and by staining with $KMnO_4$ reagent and subsequent heating. R_f values are reported on silica gel. Column chromatography was carried out on silica gel 60, 230-240 mesh. Routine NMR measurements were recorded on Bruker AV-400 or DPX-300 spectrometers. The values of δ are expressed in ppm and referred to the residual signal of the solvent ($CDCl_3$). The coupling constants values *J* are expressed in Hz. ^{1}H NMR: splitting pattern abbreviations are: s, singlet; d, doublet; t, triplet; q, quartet; p, pentet; sext, sextet; at, apparent triplet; dd, double doublet; ddd, double doublet of doublet; adt, apparent double triplet; m, multiplet. ^{13}C NMR: multiplicities were determined by DEPT, abbreviations are: q, CH_3; t, CH_2; d, CH; s quaternary carbons, except for compound **5t**, which bears fluorine atoms. For compound **5t**, the abbreviation (q) regarding the carbon multiplicity refers to the F-C coupling and there will be no abbreviation if there is no F-C coupling; the number of hydrogen atoms linked to a determined carbon atom is indicated as C, CH, CH_2 or CH_3. In the cases where a mixture of two diastereomers was observed, the abbreviation "min" refers to the signals assigned to the minor diastereomer and the abbreviation "maj" to the signals belonging to the major one; in the cases where nothing is specified, either it hasn't been possible to assign the signal to any of the diastereomers or it belongs to both of them. ^{19}F NMR: Trichlorofluoromethane ($CFCl_3$) was employed as reference standard (δ = 0). ^{2}D NMR: The signal of the solvent was employed as reference ($CDCl_3$, δ = 7.26 ppm). Structural assignments were made with additional information from gCOSY, gHSQC, and gHMBC experiments, carried out on Bruker DPX-300, Bruker AV-400 or Bruker AV-600 spectrometers. Standard pulse sequences were employed for the DEPT

experiments. Mass spectra were determined at Universidad de Oviedo on a Bruker model Impact II mass spectrometer equipped with a TOF analyzer for high resolution mass spectra (HRMS) or on an Agilent 6560 LC-IM-Q-TOF mass spectrometer with samples introduced by flow injection analysis (FIA) through a UHPLC system using an acetonitrile/water (0.1% formic acid) solvent mixture, both of them operated in positive electrospray ionization (ESI) mode; low resolution mass spectra were obtained on a gas chromatograph mass spectrometer Shimadzu QP2010 Plus with auto injector AOC-20i; electrospray ionization (ESI) was employed.

Group VI metal Fischer carbene complexes 1, 2 and 6 employed.

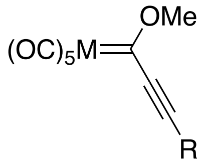

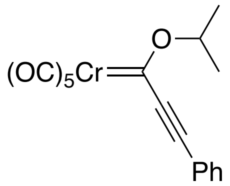

6

M = Cr (**1**)	M = W (**2**)	R
**1a**	**2a**	Ph
**1b**		*p*-Tol
**1c**		*p*-MeO-C_6H_4-
**1d**		*p*-F_3C-C_6H_4-
**1e**		*o*-Tol
**1f**		*o*-MeO-C_6H_4-
**1g**		Cyclohexen-1-yl
**1h**		*n*-Bu
**1i**		*t*-Bu
**1j**		*N*-Methylindol-3-yl
**1k**		*p*-Cl-C_6H_4-

Synthesis of 4,5-dihydro-1,2,4-oxadiazoles 4. General procedure A. 4,5-Dihydro-1,2,4-oxadiazoles **4**, were prepared by 1,3-dipolar cycloaddition of nitrile oxides (generated from *C*-chlorooximes **S3** with Et3N) with aldimines **S6**, by a variation of a previously reported procedure.[8,11] Hydroxymoyl chlorides (*C*-chlorooximes) **S3** were formed by NCS-chlorination of oximes **S2**, which were readily generated by standard condensation between aldehyde **S1** and hydroxylamine hydrochloride.[12] Aldimines **S6** were synthesized by condensation of aromatic aldehydes **S4** with amines **S5**.[13] All experimental procedures are detailed below.

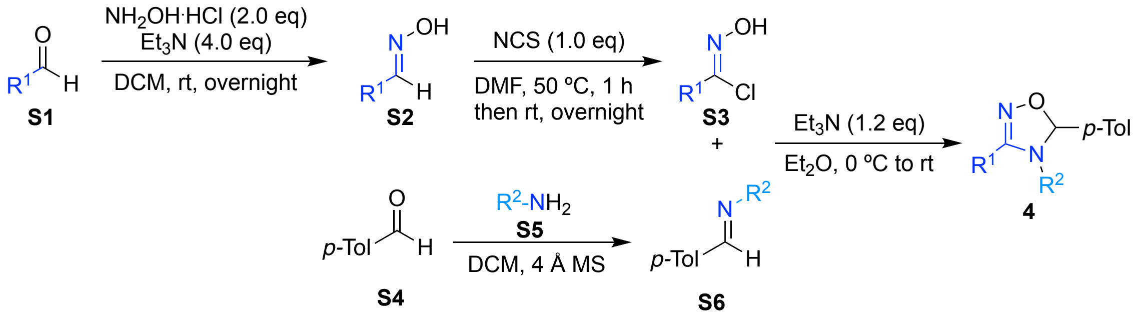

Hydroxylamine hydrochloride (1.39 g, 20 mmol, 2.0 equiv) and triethylamine (5.58 mL, 40 mmol, 4.0 equiv) were added to a solution of aldehyde **S1** (10.0 mmol, 1.0 equiv) in anhydrous DCM (15 mL) in a dry Schlenk flask under N_2 atmosphere. The resulting mixture was stirred at room temperature until consumption of starting materials (usually overnight). Then, saturated $NaHCO_3$ was added to the reaction mixture at 0 °C and the resulting mixture was extracted with DCM (3 x 20 mL), dried over Na_2SO_4, filtered and concentrated under reduced pressure. The residue was purified by silica gel chromatography (HxH/AcOEt 5:1) to give the oxime **S2** in pure form

A solution of *N*-chlorosuccinimide (1.34 g, 10.0 mmol, 1.0 equiv) in DMF (80 mL) was added dropwise to a solution of oxime **S2** (10.0 mmol, 1.0 equiv) in DMF (80 mL) in a round-bottomed flask over 30 minutes, at 50 °C. The resulting mixture was stirred for 1 hour, and then allowed to stir at room temperature overnight. A mixture of ice-water was added to the reaction mixture and extracted with Et_2O (3 x 20 mL). The combined organic extracts were washed with ice-water and brine, dried over Na_2SO_4, filtered and concentrated under reduced pressure to give hydroxymoyl chlorides **S3** in pure form.

Amine **S5** (10.0 mmol, 1.0 equiv) was added dropwise via syringe to a solution of *p*-tolualdehyde **S4** (10.0 mmol, 1.0 equiv) in anhydrous DCM (100 mL) in a dry Schlenk flask under N_2 atmosphere in the presence of activated 4 Å MS. The reaction mixture was stirred at room temperature and, upon consumption of starting materials (usually overnight), filtered through a pad of Celite and concentrated under reduced pressure to give imine **S6** in pure form.

A solution of triethylamine (0.50 – 3.35 mL, 3.60 – 24.0 mmol, 1.2 equiv) in anhydrous Et_2O (22.5 – 150 mL) was added dropwise during 15 min at 0 °C to a solution of hydroxymoyl chloride **S3** (3.30 – 22.0 mmol, 1.1 equiv) and imine **S6** (3.00 – 20.0 mmol, 1.0 equiv) in anhydrous Et_2O (75 mL) in a dry Schlenk under N_2 atmosphere. The reaction was then allowed to stir at room temperature overnight. Then the reaction mixture was

filtered through Celite and concentrated under reduced pressure. The residue was purified by silica gel chromatography (HxH/AcOEt 20:1) to give 4,5-dihydro-1,2,4-oxadiazole **4** in pure form.

Table S1. Synthesis of 4,5-dihydro-1,2,4-oxadiazoles **4.**

Entry	4	R^1	R^2	Yield[a]
1	**4a**	*p*-Cl-C_6H_4-	Bn	88
2	**4b**	*p*-MeO-C_6H_4-	Bn	83
3	**4c**	Ph	Bn	32
4	**4d**	*o*-Tol-	Bn	34
5	**4e**	*o*-Br-C_6H_4-	Bn	47
6	**4f**	*m*-Cl-C_6H_4-	Bn	10
7	**4g**	*s*-Bu	Bn	15
8	**4h**	*p*-Cl-C_6H_4-	*p*-MeO-C_6H_4-	61
9	**4i**	*p*-Cl-C_6H_4-	Ph	32
10	**4j**	*p*-Cl-C_6H_4-	*n*-Bu	32
11	**4k**	*p*-MeO-C_6H_4-	*n*-Bu	49
12	**4l**	*p*-Cl-C_6H_4-	*p*-MeO-C_6H_4-CH_2-	54
14	**4m**	Ph	*p*-MeO-C_6H_4-CH_2-	88

[a] Isolated yield for the [3+2]-cycloaddition step.

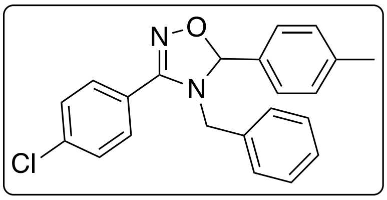

4a

4-Benzyl-3-(*p*-chlorophenyl)-5-(*p*-tolyl)-4,5-dihydro-1,2,4-oxadiazole (4a). Prepared according to <u>General Procedure A</u> in the yield indicated in Table S1 (88% for the cycloaddition step, 5329,6 mg, 14.69 mmol) as a brown oil. Rf = 0.20 (HexH/AcOEt 8:2); ^{1}H NMR (300 MHz, $CDCl_3$) δ 7.72 – 7.62 (m, 2H), 7.53 – 7.43 (m, 2H), 7.42 – 7.35 (m, 2H), 7.35 – 7.28 (m, 3H), 7.27 – 7.20 (m, 2H), 7.14 – 7.04 (m, 2H), 6.27 (s, 1H), 4.47 (d, *J* = 15.9 Hz, 1H), 4.08 (d, *J* = 15.9 Hz, 1H), 2.41 (s, 3H); ^{13}C NMR (75 MHz, $CDCl_3$) δ 157.3 (s), 139.7 (s), 136.8 (s), 135.5 (s), 134.9 (s), 129.6 (d, 2CH), 129.4 (d, 4CH), 128.8

(d, 2CH), 127.9 (d), 127.8 (d, 2CH), 127.3 (d, 2CH), 123.9 (s), 96.9 (d), 49.8 (t), 21.4 (q); HRMS (ESI): (*m/z*) calculated for $C_{22}H_{20}ClN_2O$ [M+H]$^+$: 363.1259, found: 363.1259.

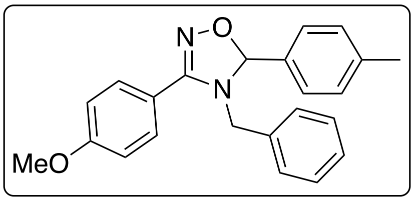

4b

4-Benzyl-3-(*p*-methoxyphenyl)-5-(*p*-tolyl)-4,5-dihydro-1,2,4-oxadiazole (4b). Prepared according to General Procedure A in the yield indicated in Table S1 (83% for the cycloaddition step, 897.3 mg, 2.5 mmol) as a brown-yellow oil. Rf = 0.30 (HexH/AcOEt 8:2); ^{1}H NMR (300 MHz, $CDCl_3$) δ 7.76 – 7.61 (m, 2H), 7.41 – 7.34 (m, 2H), 7.34 – 7.26 (m, 3H), 7.25 – 7.17 (m, 2H), 7.14 – 7.06 (m, 2H), 7.05 – 6.97 (m, 2H), 6.22 (s, 1H), 4.51 (d, *J* = 15.9 Hz, 1H), 4.07 (d, *J* = 15.9 Hz, 1H), 3.86 (s, 3H), 2.39 (s, 3H); ^{13}C NMR (75 MHz, $CDCl_3$) δ 161.5 (s), 157.9 (s), 139.6 (s), 135.9 (s), 135.4 (s), 129.9 (d, 2CH), 129.4 (d, 2CH), 128.8 (d, 2CH), 127.9 (d, 2CH), 127.8 (d), 127.4 (d, 2CH), 117.4 (s), 114.5 (d, 2CH), 96.5 (d), 55.5 (q), 49.9 (t), 21.4 (q); HRMS (ESI): (*m/z*) calculated for $C_{23}H_{23}N_2O_2$ [M+H]$^+$: 359.1754, found: 359.1750.

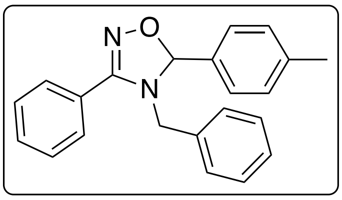

4c

4-Benzyl-3-phenyl-5-(*p*-tolyl)-4,5-dihydro-1,2,4-oxadiazole (4c). Prepared according to General Procedure A in the yield indicated in Table S1 (32% for the cycloaddition step, 844.9 mg, 2.60 mmol) as a brown oil. Rf = 0.40 (HexH/AcOEt 8:2); ^{1}H NMR (300 MHz, $CDCl_3$) δ 7.89 – 7.67 (m, 2H), 7.56 – 7.46 (m, 3H), 7.44 – 7.37 (m, 2H), 7.36 – 7.27 (m, 3H), 7.24 (d, *J* = 7.8 Hz, 2H), 7.15 – 7.06 (m, 2H), 6.27 (s, 1H), 4.53 (d, *J* = 15.8 Hz, 1H), 4.09 (d, *J* = 15.8 Hz, 1H), 2.41 (s, 3H); ^{13}C NMR (75 MHz, $CDCl_3$) δ 158.1 (s), 139.6 (s), 135.7 (s), 135.1 (s), 130.8 (d), 129.3 (d, 2CH), 129.1 (d, 2CH), 128.7 (d, 2CH), 128.4 (d, 2CH), 127.9 (d, 2CH), 127.8 (d), 127.4 (d, 2CH), 125.4 (s), 96.6 (d), 49.7 (t), 21.4 (q); HRMS (ESI): (*m/z*) calculated for $C_{22}H_{21}N_2O$ [M+H]$^+$: 329.1648, found: 329.1649.

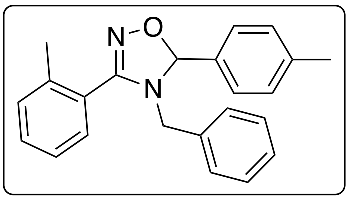

4d

4-Benzyl-3-(*o*-tolyl)-5-(*p*-tolyl)-4,5-dihydro-1,2,4-oxadiazole (4d). Prepared according to General Procedure A in the yield indicated in Table S1 (34% for the cycloaddition step, 756.2 mg, 2.21 mmol) as a brown oil. Rf = 0.40 (HexH/AcOEt 8:2); ^{1}H NMR (300 MHz, $CDCl_3$) δ 7.53 (dd, J = 7.9, 1.6 Hz, 1H), 7.48 – 7.37 (m, 3H), 7.37 – 7.18 (m, 7H), 7.06 – 6.94 (m, 2H), 6.26 (s, 1H), 4.19 (d, J = 15.5 Hz, 1H), 3.85 (d, J = 15.5 Hz, 1H), 2.52 (s, 3H), 2.42 (s, 3H); ^{13}C NMR (75 MHz, $CDCl_3$) δ 156.9 (s), 139.8 (s), 138.0 (s), 135.2 (s), 134.7 (s), 130.9 (d), 130.5 (d), 130.1 (d), 129.5 (d, 2 CH), 128.7 (d, 2CH), 128.1 (d, 2CH), 127.8 (d), 127.6 (d, 2CH), 126.2 (d), 124.6 (s), 95.6 (d), 48.0 (t), 21.4 (q), 19.9 (q); HRMS (ESI): (*m/z*) calculated for $C_{23}H_{23}N_2O$ $[M+H]^+$: 343.1805, found: 343.1804.

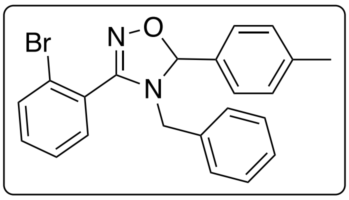

4e

4-Benzyl-3-(*o*-bromophenyl)-5-(*p*-tolyl)-4,5-dihydro-1,2,4-oxadiazole (4e). Prepared according to General Procedure A in the yield indicated in Table S1 (47% for the cycloaddition step, 3835.7 mg, 9.42 mmol) as a yellow solid. Mp. = 107.5 – 110.5 ºC; Rf = 0.30 (HexH/AcOEt 8:2); ^{1}H NMR (300 MHz, $CDCl_3$) δ 7.75 – 7.68 (m, 1H), 7.61 – 7.55 (m, 1H), 7.52 – 7.31 (m, 4H), 7.30 – 7.23 (m, 5H), 7.02 (dt, J = 6.7, 2.2 Hz, 2H), 6.30 (s, 1H), 4.19 (d, J = 15.6 Hz, 1H), 3.89 (d, J = 15.6 Hz, 1H), 2.41 (s, 3H); ^{13}C NMR (75 MHz, $CDCl_3$) δ 156.4 (s), 140.0 (s), 135.2 (s), 134.3 (s), 133.6 (d), 132.4 (d), 132.1 (d), 129.5 (d, 2CH), 128.7 (d, 2CH), 128.12 (d, 2CH), 128.08 (d, 2CH), 127.9 (d), 127.8 (d), 126.9 (s), 123.6 (s), 96.4 (d), 47.9 (t), 21.5 (q); HRMS (ESI): (*m/z*) calculated for $C_{22}H_{20}BrN_2O$ $[M+H]^+$: 407.0754, found: 407.0752.

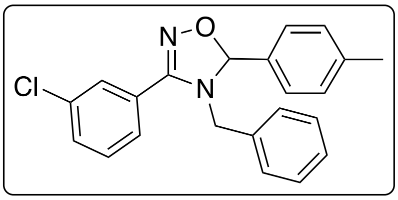

4f

4-Benzyl-3-(m-chlorophenyl)-5-(*p*-tolyl)-4,5-dihydro-1,2,4-oxadiazole (4f). Prepared according to General Procedure A in the yield indicated in Table S1 (10% for the cycloaddition step, 414.0 mg, 1.14 mmol) as a brown oil. Rf = 0.50 (HexH/AcOEt 8:2); ^{1}H NMR (300 MHz, $CDCl_3$) δ 7.74 (t, *J* = 1.9 Hz, 1H), 7.62 (dt, *J* = 7.4, 1.5 Hz, 1H), 7.50 (ddd, *J* = 8.1, 2.1, 1.4 Hz, 1H), 7.47 – 7.42 (m, 1H), 7.42 – 7.35 (m, 2H), 7.34 – 7.28 (m, 3H), 7.27 – 7.21 (m, 2H), 7.10 (dd, *J* = 7.3, 2.2 Hz, 2H), 6.27 (s, 1H), 4.49 (d, *J* = 15.8 Hz, 1H), 4.09 (d, *J* = 15.8 Hz, 1H), 2.41 (s, 3H); ^{13}C NMR (75 MHz, $CDCl_3$) δ 157.0 (s), 139.7 (s), 135.4 (s), 135.0 (s), 134.9 (s), 130.9 (d), 130.4 (d), 129.4 (d, 2CH), 128.8 (d, 2CH), 128.3 (d), 128.0 (d), 127.9 (d, 2CH), 127.30 (d, 2CH), 127.26 (s), 126.4 (d), 97.0 (d), 49.9 (t), 21.4 (q); HRMS (ESI): (*m/z*) calculated for $C_{22}H_{20}ClN_2O$ $[M+H]^+$: 363.1259, found: 363.1259.

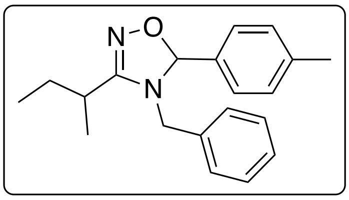

4g

4-Benzyl-3-(*sec*-butyl)-5-(*p*-tolyl)-4,5-dihydro-1,2,4-oxadiazole (4g). Prepared according to General Procedure A, as a 4:1 mixture of diastereomers in the yield indicated in Table S1 (15% for the cycloaddition step, 320.2 mg, 1.04 mmol) as a brown oil. Rf = 0.40 (HexH/AcOEt 8:2); ^{1}H NMR (300 MHz, $CDCl_3$) δ 7.38 – 7.25 (m, 5H, maj + 5H, min), 7.20 – 7.11 (m, 4H, maj + 4H, min), 6.07 (s, 1H, min), 6.04 (s, 1H, maj), 4.41 (d, *J* = 16.4 Hz, 1H, maj + 1H, min), 3.93 (d, *J* = 16.4, 1H, maj + 1H, min), 2.59 – 2.44 (m, 2H, maj + 2 H, min), 2.38 (s, 3H, maj + 3H, min), 1.97 – 1.88 (m, 1H, min), 1.87 – 1.74 (m, 1H, maj), 1.71 – 1.52 (m, 2H, maj + 2H, min), 1.34 (d, *J* = 6.9 Hz, 3H, maj), 1.31 (d, *J* = 6.9 Hz, 3H, min), 1.11 – 0.99 (m, 3H, maj + 3H, min); ^{13}C NMR (75 MHz, $CDCl_3$) δ 160.3 (s), 139.7 (s), 135.9 (s), 135.0 (s), 129.4 (d, 2CH), 128.90 (d, 2CH, maj), 128.88 (d, 2CH, min), 127.82 (d), 127.79 (d, 2CH, min), 127.7 (d, 2CH, maj), 127.4 (d, 2CH, maj), 127.3 (d, 2CH, min), 95.7 (d, maj), 95.5 (d, min), 47.0 (t, maj), 46.7 (t, min), 31.6 (d,

min), 30.9 (d, maj), 28.1 (t, maj), 26.7 (t, min), 21.4 (t), 18.9 (t, min), 16.9 (t, maj), 12.2 (q, min), 11.3 (q, maj); HRMS (ESI): (*m/z*) calculated for $C_{20}H_{25}N_2O$ $[M+H]^+$: 309.1961, found: 309.1956.

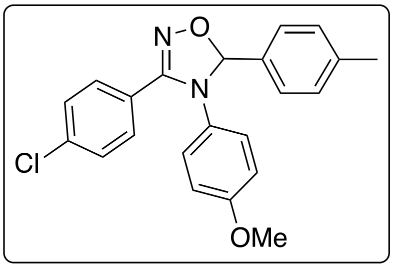

4h

3-(*p*-Chlorophenyl)-4-(*p*-methoxyphenyl)-5-(*p*-tolyl)-4,5-dihydro-1,2,4-oxadiazole (4h). Prepared according to General Procedure A in the yield indicated in Table S1 (61% for the cycloaddition step, 2262.1 mg, 5.97 mmol) as a brown solid. Mp = 106.3 – 118.9 ºC; Rf = 0.50 (HexH/AcOEt 8:2); ^{1}H NMR (300 MHz, $CDCl_3$) δ 7.51 (d, *J* = 8.6 Hz, 2H), 7.47 (d, *J* = 8.2 Hz, 2H), 7.30 (d, *J* = 8.5 Hz, 2H), 7.26 (d, *J* = 7.6 Hz, 2H), 6.78 (d, *J* = 9.1 Hz, 2H), 6.72 (d, *J* = 9.1 Hz, 2H), 6.39 (s, 1H), 3.74 (s, 3H), 2.41 (s, 3H); ^{13}C NMR (75 MHz, $CDCl_3$) δ 158.0 (s), 155.2 (s), 140.0 (s), 136.4 (s), 135.7 (s), 133.5 (s), 129.6 (d, 2CH), 129.4 (d, 2CH), 129.0 (d, 2CH), 127.6 (d, 2CH), 127.2 (d, 2CH), 124.0 (s), 114.7 (d, 2CH), 101.4 (d), 55.6 (q), 21.5 (q); HRMS (ESI): (*m/z*) calculated for $C_{22}H_{20}ClN_2O_2$ $[M+H]^+$: 379.1208, found: 379.1216.

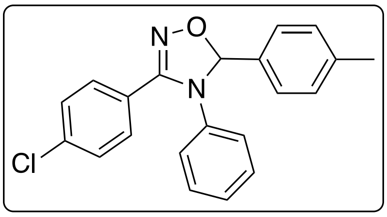

4i

3-(*p*-Chlorophenyl)-4-phenyl-5-(*p*-tolyl)-4,5-dihydro-1,2,4-oxadiazole (4i). [14] Prepared according to General Procedure A in the yield indicated in Table S1 (32% for the cycloaddition step, 80.0 mg, 0.23 mmol) as a brown oil. The spectroscopic data obtained are in agreement with the ones reported in the literature.

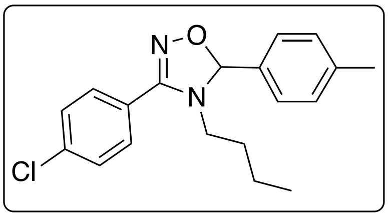

4j

4-(*n*-Butyl)-3-(*p*-chlorophenyl)-5-(*p*-tolyl)-4,5-dihydro-1,2,4-oxadiazole (4j). Prepared according to General Procedure A in the yield indicated in Table S1 (32% for the cycloaddition step, 317.7 mg, 0.97 mmol) as a brown oil. Rf = 0.50 (HexH/AcOEt 8:2); ^{1}H NMR (300 MHz, $CDCl_3$) δ 7.65 – 7.54 (m, 2H), 7.50 – 7.41 (m, 4H), 7.29 – 7.19 (m, 2H), 6.28 (s, 1H), 3.20 – 2.88 (m, 2H), 2.40 (s, 3H), 1.58 – 1.31 (m, 1H), 1.34 – 1.06 (m, 3H), 0.76 (t, *J* = 7.3 Hz, 3H); ^{13}C NMR (75 MHz, $CDCl_3$) δ 157.9 (s), 139.8 (s), 136.7 (s), 136.0 (s), 129.6 (d, 2CH), 129.5 (d, 2CH), 129.3 (d, 2CH), 127.3 (d, 2CH), 124.3 (s), 98.5 (d), 47.3 (t), 30.3 (t), 21.5 (q), 19.9 (t), 13.7 (q); HRMS (ESI): (*m/z*) calculated for $C_{19}H_{22}ClN_2O$ $[M+H]^+$: 329.1415, found: 329.1408.

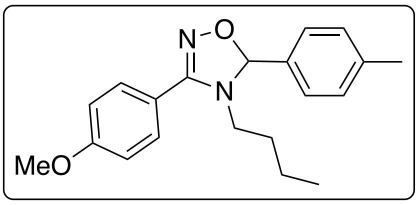

4k

4-(*n*-Butyl)-3-(*p*-methoxyphenyl)-5-(*p*-tolyl)-4,5-dihydro-1,2,4-oxadiazole (4k). Prepared according to General Procedure A in the yield indicated in Table S1 (49% for the cycloaddition step, 476.9 mg, 1.47 mmol) as a brown oil. Rf = 0.20 (HexH/AcOEt 8:2); ^{1}H NMR (300 MHz, $CDCl_3$) δ 7.57 (d, *J* = 8.8 Hz, 2H), 7.46 (d, *J* = 8.1 Hz, 2H), 7.23 (d, *J* = 7.6 Hz, 2H), 6.97 (d, *J* = 8.7 Hz, 2H), 6.25 (s, 1H), 3.83 (s, 3H), 3.10 – 3.01 (m, 2H), 2.38 (s, 3H), 1.48 – 1.37 (m, 1H), 1.32 – 1.04 (m, 3H), 0.82 – 0.66 (m, 3H); ^{13}C NMR (75 MHz, CDCl3) δ 161.4 (s), 158.6 (s), 139.6 (s), 136.4 (s), 129.7 (d, 2CH), 129.4 (d, 2CH), 127.3 (d, 2CH), 117.8 (s), 114.4 (d, 2CH), 98.1 (d), 55.5 (q), 47.3 (t), 30.4 (t), 21.4 (q), 19.9 (t), 13.7 (q); HRMS (ESI): (*m/z*) calculated for $C_{20}H_{25}N_2O_2$ $[M+H]^+$: 325.1911, found: 325.1906.

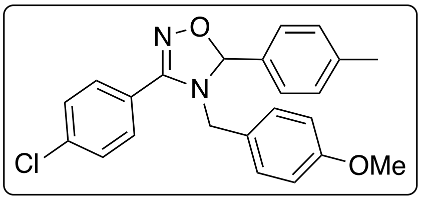

4l

3-(4-Chlorophenyl)-4-(*p*-methoxybenzyl)-5-(*p*-tolyl)-4,5-dihydro-1,2,4-oxadiazole (4l). Prepared according to General Procedure A in the yield indicated in Table S1 (54% for the cycloaddition step, 1968.2 mg, 5.01 mmol) as a brown oil. Rf = 0.4 (HexH/AcOEt 8:2). ^{1}H NMR (300 MHz, $CDCl_3$) δ 7.67 (d, *J* = 8.4 Hz, 2H), 7.47 (d, *J* = 8.4 Hz, 2H), 7.38 (d, *J* = 8.0 Hz, 2H), 7.23 (d, *J* = 7.8 Hz, 2H), 6.97 (d, *J* = 8.6 Hz, 2H), 6.84 (d, *J* = 8.6 Hz, 2H), 6.23 (s, 1H), 4.42 (d, *J* = 15.6 Hz, 1H), 3.98 (d, *J* = 15.6 Hz, 1H), 3.79 (s, 3H), 2.40 (s, 3H); ^{13}C NMR (75 MHz, $CDCl_3$) δ 159.1 (s), 157.2 (s), 139.6 (s), 136.7 (s), 134.9 (s), 129.6 (d, 2CH),129.3 (d, 4CH), 129.2 (d, 2CH), 127.3 (d, 2CH), 127.1 (s), 123.9 (s), 114.0 (d, 2CH), 96.5 (d), 55.2 (t), 49.1 (q), 21.3 (q); HRMS (ESI): (*m/z*) calculated for $C_{23}H_{22}ClN_2O_2$ [M+H]$^+$: 393.1364, found: 393.1375.

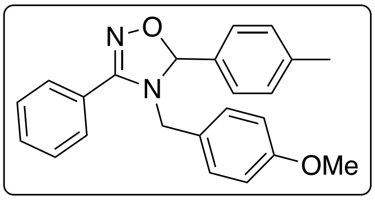

4m

4-(*p*-Methoxybenzyl)-3-phenyl-5-(*p*-tolyl)-4,5-dihydro-1,2,4-oxadiazole (4m). Prepared according to General Procedure A in the yield indicated in Table S1 (88% for the cycloaddition step, 527.3 mg, 1.31 mmol) as a brown oil. Rf = 0.30 (HexH/AcOEt 8:2); ^{1}H NMR (300 MHz, $CDCl_3$) δ 7.63 (d, *J* = 8.8 Hz, 2H), 7.34 (d, *J* = 8.1 Hz, 2H), 7.30 – 7.24 (m, 3H), 7.19 (d, *J* = 7.3 Hz, 2H), 7.08 (d, *J* = 7.8 Hz, 2H), 6.98 (d, *J* = 8.9 Hz, 2H), 6.20 (s, 1H), 4.47 (d, *J* = 15.8 Hz, 1H), 4.05 (d, *J* = 15.8 Hz, 1H), 3.85 (s, 3H), 2.37 (s, 3H); ^{13}C NMR (75 MHz, $CDCl_3$) δ 161.6 (s), 158.0 (s), 139.6 (s), 136.0 (s), 135.4 (s), 129.9 (s), 129.4 (d, 2CH), 128.8 (d, 2CH), 127.9 (d, 2CH), 127.8 (d, 2CH), 127.4 (d), 117.5 (s), 114.6 (d, 2CH), 96.6 (d), 55.5 (q), 49.9 (q), 21.4 (q); HRMS (ESI): (*m/z*) calculated for $C_{23}H_{23}N_2O_2$ [M+H]$^+$: 359.1754, found: 359.1750.

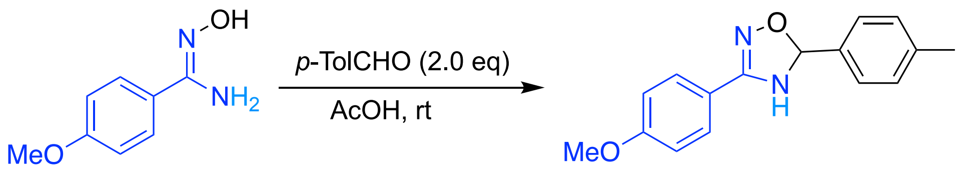

4n

Synthesis of 3-(4-methoxyphenyl)-5-(*p*-tolyl)-4,5-dihydro-1,2,4-oxadiazole (4n). This compound was prepared according to the procedure described by Liu and coworkers.[8] *p*-Tolualdehyde (6.008 g, 5.92 mL, 50 mmol) was added to a solution of (*Z*)-*N*'-hydroxy-4-methoxybenzimidamide[7] (830.9 mg, 5 mmol) in AcOH (5 mL) under argon atmosphere. After stirring for 48 h at room temperature, solvent was evaporated under reduced pressure and the residue was quenched with saturated sodium bicarbonate at 0 ºC. Then the reaction mixture was extracted with ethyl acetate, washed with brine and dried over anhydrous Na_2SO_4. The solvent was evaporated under reduced pressure and the residue was purified by column chromatography on silica gel (Hexane/EtOAc: 6:1 to 3:1) to afford **4n** (161.3 mg, 0.60 mmol, 12%) as a colorless oil. Rf = 0.20 (HexH/AcOEt 8:2); ^{1}H NMR (300 MHz, $CDCl_3$) δ 7.64 (d, *J* = 8.9 Hz, 2H), 7.41 (d, *J* = 8.1 Hz, 2H), 7.21 (d, *J* = 7.8 Hz, 2H), 6.90 (d, *J* = 8.9 Hz, 2H), 6.45 (d, *J* = 3.5 Hz, 1H), 5.15 (br d, *J* = 3.7 Hz, 1H), 3.84 (s, 3H), 2.38 (s, 3H); ^{13}C NMR (75 MHz, $CDCl_3$) δ 161.7 (s), 155.7 (s), 139.6 (s), 136.3 (s), 129.5 (d, 2CH), 128.2 (d, 2CH), 126.7 (d, 2CH), 117.7 (s), 114.2 (d, 2CH), 93.4 (d), 55.4 (q), 21.4 (q); HRMS (ESI): (*m/z*) calculated for $C_{16}H_{17}N_2O_2$ $[M+H]^+$: 269.1290, found: 269.1286.

Optimization of the reaction conditions. General procedure for the determination of the NMR estimated yield. 4,5-Dihydro-1,2,4-oxadiazole **4a** (0.12-0.15 mmol, 1.2-1.5 equiv) was added to a solution of chromium alkoxy alkynyl Fischer carbene complex **1a** (33.6 mg, 0.1 mmol, 1 equiv, entries 1-11), tungsten alkoxy alkynyl Fischer carbene complex **2a** (46.8 mg, 0.1 mmol, 1 equiv, entry 12), or alkynyl ester **3** (16.0 mg, 0.1 mmol, 1 equiv, entry 13) in the solvent indicated in Table 1 (1.5 mL) in a Schlenk flask, at room temperature under argon atmosphere. The mixture was stirred at the temperatue indicated in Table 1 until complete disappearance of the Fischer carbene complex was monitored by TLC (24-96 h). A measured amount of the reference compound (1,3,5-trimethoxybenzene, 16.8 mg, 0.1 mmol, 1 equiv) was then added to the crude mixture and stirred at room temperature until it is completely dissolved. The reaction solvent is then evaporated under reduced pressure.

The preparation of the NMR sample is performed by: 1) transferring a small amount of the crude residue containing the NMR reference compound to a vial; 2) adding the deuterated solvent of choice (typically $CDCl_3$) to the vial and dissolving the crude residue; 3) filtering the sample through a pipette containing a plug of celite to the NMR tube. Under these conditions no effect of the metal on the H signals was observed. The monitored signals were those corresponding to the reference compound (δ = 6.08, 3.75 ppm) and to the benzylic H atoms of **5a** (δ = 5.25 ppm).

For the determination of the isolated yield (entries 6-12), silica gel was added to the reaction crude prior to solvent evaporation; then, solvent was evaporated under reduced pressure, and the crude residue was purified by column chromatography on silica gel (Hexane/EtOAc: 20:1 to 4:1) to afford 3,4-dihydropyrimidin-4-one **5a**.

Synthesis of 3,4-dihydropyrimidin-4-ones (5). General Procedure B. The corresponding 4,5-dihydro-1,2,4-oxadiazole **4** (0.3-1.5 mmol, 1.5 equiv) was added to a solution of the corresponding chromium alkoxy alkynyl Fischer carbene complex **1** (0.2-1 mmol, 1 equiv) in DCE (3-15 mL) in a Schlenk flask, at room temperature under argon atmosphere. The mixture was stirred at 60 ºC until complete disappearance of the Fischer carbene complex was monitored by TLC (24-72 h). Then, silica gel was added, solvent was evaporated under reduced pressure, and the crude residue was purified by column chromatography on silica gel (Hexane/EtOAc: 20:1 to 4:1) to afford 3,4-dihydropyrimidin-4-ones **5**.

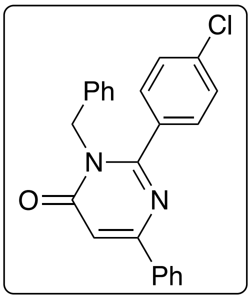

5a

3-Benzyl-2-(*p*-chlorophenyl)-3,4-dihydro-6-phenylpyrimidin-4-one (5a). Prepared according to General Procedure B from **1a** (100.0 mg, 0.30 mmol) and **4a** (161.88 mg, 0.45 mmol) in DCE (4.5 mL) in 81% yield (89 mg, 0.24 mmol) as a brown oil. Rf = 0.20 (HexH/AcOEt 8:2); ^{1}H NMR (300 MHz, $CDCl_3$) δ 8.12 – 7.88 (m, 2H), 7.50 – 7.46 (m, 3H), 7.40 (d, J = 8.4 Hz, 2H), 7.33 (d, J = 8.4 Hz, 2H), 7.30 – 7.22 (m, 3H), 7.00 (s+d, J

= 6.9 Hz, 3H), 5.25 (s, 2H). ^{13}C NMR (75 MHz, $CDCl_3$) δ 163.1 (s), 159.9 (s), 159.6 (s), 136.5 (s), 136.1 (s), 135.9 (s), 133.4 (s), 130.8 (d), 129.6 (d, 2CH), 128.8 (d, 6CH), 127.7 (d), 127.1 (d, 2CH), 126.8 (d, 2CH), 108.1 (d), 48.8 (t). HRMS (ESI): (*m/z*) calculated for $C_{23}H_{18}ClN_2O$ $[M+H]^+$: 373.1102, found: 373.1096.

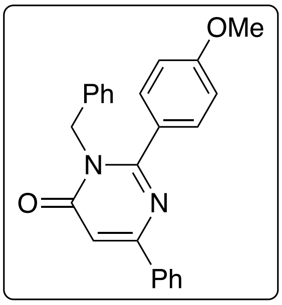

5b

3-Benzyl-3,4-dihydro-2-(*p*-methoxyphenyl)-6-phenylpyrimidin-4-one (5b). Prepared according to General Procedure B from **1a** (100.0 mg, 0.30 mmol) and **4b** (160.0, 0.45 mmol) in DCE (4.5 mL) in 69% yield (76 mg, 0.21 mmol); prepared according to General Procedure B from **1a** (134.4 mg, 0.40 mmol) and **7b** (177.8 mg, 0.60 mmol) in DCE (12.0 mL) in 62% yield (94.3 mg, 0.256 mmol) as a brown solid. Mp = 128.7 – 131.0 ºC; Rf = 0.18 (HexH/AcOEt 8:2); ^{1}H NMR (300 MHz, $CDCl_3$) δ 8.08 – 7.99 (m, 2H), 7.51 – 7.44 (m, 3H), 7.38 (d, *J* = 8.4 Hz, 2H), 7.33 – 7.20 (m, 3H), 7.04 (d, *J* = 6.1 Hz, 2H), 6.97 (s, 1H), 6.93 (d, *J* = 8.3 Hz, 2H), 5.31 (s, 2H), 3.87 (s, 3H). ^{13}C NMR (75 MHz, $CDCl_3$) δ 163.6 (s), 161.2 (s), 160.6 (s), 160.0 (s), 136.6 (s), 136.3 (s), 130.7 (d), 130.0 (d, 2CH), 128.86 (d, 2CH), 128.76 (d, 2CH), 127.6 (d), 127.5 (s), 127.3 (d, 2CH), 126.9 (d, 2CH), 114.0 (d, 2CH), 107.7 (d), 55.6 (q), 49.1 (t). HRMS (ESI): (*m/z*) calculated for $C_{24}H_{21}N_2O_2$ $[M+H]^+$: 369.1598, found: 369.1591.

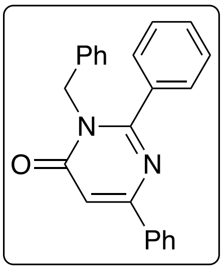

5c

3-Benzyl-3,4-dihydro-2,6-diphenylpyrimidin-4-one (5c). Prepared according to General Procedure B from **1a** (100.0 mg, 0.30 mmol) and **4c** (146.2 mg, 0.45 mmol) in DCE (4.5 mL) in 53% yield (53.2 mg, 0.16 mmol) as a brown solid. Mp = 135.7 – 138.6

ºC; Rf = 0.10 (HexH/AcOEt 8:2); ^{1}H NMR (300 MHz, $CDCl_3$) δ 8.08 – 7.99 (m, 2H), 7.60 – 7.46 (m, 8H), 7.31 – 7.15 (m, 3H), 7.03-6.94 (s+m, 3H), 5.28 (s, 2H). ^{13}C NMR (75 MHz, $CDCl_3$) δ 163.2 (s), 160.6 (s), 159.9 (s), 136.3 (s), 136.1 (s), 134.9 (s), 130.7 (d), 130.2 (d), 128.8 (d, 2 CH), 128.6 (d, 2CH), 128.5 (d, 2CH), 128.1 (d, 2CH), 127.5 (d), 127.1 (d, 2CH), 126.9 (d, 2CH), 108.0 (d), 48.7 (t). HRMS (ESI) (*m/z*) calculated for $C_{23}H_{19}N_2O$ $[M+H]^+$: 339.1492, found: 339.1483.

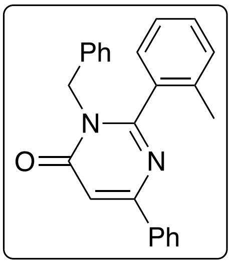

5d

3-Benzyl-3,4-dihydro-6-phenyl-2-(*o*-tolyl)pyrimidin-4-one (5d). Prepared according to <u>General Procedure B</u> from **1a** (100.0 mg, 0.30 mmol) and **4d** (152.8 mg, 0.45 mmol) in DCE (4.5 mL) in 62% yield (64.5 mg, 0.18 mmol) as a brown oil. Rf = 0.33 (HexH/AcOEt 8:2); ^{1}H NMR (300 MHz, $CDCl_3$) δ 8.07 – 7.98 (m, 2H), 7.51 – 7.45 (m, 3H), 7.44 – 7.36 (m, 1H), 7.29 – 7.08 (m, 6H), 7.02 (s, 1H), 6.95 – 6.80 (m, 2H), 5.17 (s, 2H), 1.98 (s, 3H). ^{13}C NMR (75 MHz, $CDCl_3$) δ 163.3 (s), 160.2 (s), 160.0 (s), 136.2 (s), 136.0 (s), 135.9 (s), 134.5 (s), 130.6 (d, 2CH), 129.9 (d), 128.7 (d, 2CH), 128.4 (d, 2CH), 127.8 (d, 2CH), 127.7 (d, 2CH), 127.2 (d, 2CH), 125.8 (d), 108.2 (d), 47.9 (t), 19.0 (q). HRMS (ESI): (*m/z*) calculated for $C_{24}H_{21}N_2O$ $[M+H]^+$: 353.1648, found: 353.1636.

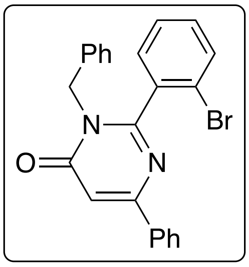

5e

3-Benzyl-2-(*o*-bromophenyl)-3,4-dihydro-6-phenylpyrimidin-4-one (5e). Prepared according to <u>General Procedure B</u> from **1a** (100.0 mg, 0.30 mmol) and **4e** (181.7 mg, 0.45 mmol) in DCE (4.5 mL) in 54% yield (67.2 mg, 0.16 mmol) as a brown oil. Rf = 0.20 (HexH/AcOEt 8:2); ^{1}H NMR (300 MHz, $CDCl_3$) δ 8.06 – 7.94 (m, 2H), 7.69 (dd, J = 8.0, 1.2 Hz, 1H), 7.52 – 7.42 (m, 3H), 7.40 – 7.33 (m, 1H), 7.27 (td, J = 7.6, 1.2 Hz 1H), 7.24

– 7.15 (m, 3H), 7.08 – 7.01 (s+m, 2H), 6.95 – 6.86 (m, 2H) 5.72 (d, J = 15.0 Hz, 1H), 4.61 (d, J = 15.0 Hz, 1H). ^{13}C NMR (75 MHz, $CDCl_3$) δ 163.0 (s), 160.3 (s), 158.8 (s), 136.2 (s, 2C), 136.0 (s), 132.9 (d), 131.3 (d), 130.8 (d), 130.4 (d), 128.9 (d, 2CH), 128.6 (d, 2CH), 127.8 (d), 127.6 (d, 2CH), 127.5 (d), 127.3 (d, 2CH), 121.6 (s), 108.9 (d), 48.0 (t). HRMS (ESI): (*m/z*) calculated for $C_{23}H_{18}BrN_2O$ $[M+H]^+$: 417.0597, found: 417.0592.

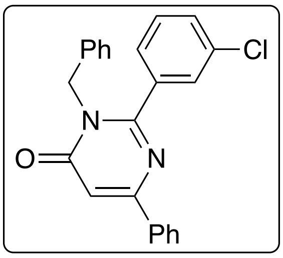

5f

3-Benzyl-2-(*m*-chlorophenyl)-3,4-dihydro-6-phenylpyrimidin-4-one (5f). Prepared according to General Procedure B from **1a** (290.2 mg, 0.86 mmol) and **4f** (469.8 mg, 1.29 mmol) in DCE (12.9 mL) in 32% yield (103.5 mg, 0.28 mmol) as a yellow oil. Rf = 0.23 (HexH/AcOEt 8:2); ^{1}H NMR (300 MHz, $CDCl_3$) δ 8.06 – 7.99 (m, 2H), 7.62 – 7.41 (m, 4H), 7.41 – 7.16 (m, 6H), 7.10 – 6.84 (m, 3H), 5.24 (s, 2H); ^{13}C NMR (75 MHz, $CDCl_3$) δ 163.2 (s), 160.0 (s), 159.3 (s), 136.6 (s), 136.2 (s), 136.0 (s), 134.7 (s), 130.9 (d), 130.5 (d), 129.9 (d), 128.94 (d, 2CH), 128.88 (d, 2CH), 128.6 (d), 127.9 (d), 127.3 (d, 2CH), 127.1 (d, 2CH), 126.2 (d), 108.5 (d), 48.8 (t); HRMS (ESI): (*m/z*) calculated for $C_{23}H_{18}ClN_2O$ $[M+H]^+$: 373.1102, found: 373.1101.

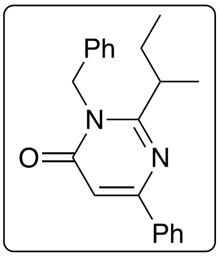

5g

3-Benzyl-2-(*sec*-butyl)-3,4-dihydro-6-phenylpyrimidin-4-one (5g). Prepared according to General Procedure B from **1a** (100.0 mg, 0.30 mmol) and **4g** (137.6 mg, 0.45 mmol) in DCE (4.5 mL) in 74% yield (70 mg, 0.22 mmol) as a brown solid. Mp = 77.6 – 81.6 ºC; Rf = 0.35 (HexH/AcOEt 8:2); ^{1}H NMR (300 MHz, $CDCl_3$) δ 8.15 – 8.02 (m, 2H), 7.56 – 7.41 (m, 3H), 7.41 – 7.23 (m, 3H), 7.21 (d, J = 6.6 Hz, 2H), 6.90 (s, 1H), 5.52 (d, J = 15.9 Hz, 1H), 5.36 (d, J = 15.9 Hz, 1H), 3.02 – 2.75 (m, 1H), 2.10 – 1.80 (m,

1H), 1.71 – 1.45 (m, 1H), 1.23 (d, J = 6.6 Hz, 3H), 0.76 (t, J = 7.4 Hz, 3H); ^{13}C NMR (75 MHz, $CDCl_3$) δ 166.2 (s), 163.7 (s), 159.6 (s), 136.5 (s), 136.3 (s), 130.6 (d), 128.9 (d, 2CH), 128.7 (d, 2CH), 127.7 (d), 127.0 (d, 2CH), 126.5 (d, 2CH), 106.4 (d), 45.7 (t), 39.4 (d), 28.9 (t), 19.5 (q), 11.9 (q). HRMS (ESI): (*m/z*) calculated for $C_{21}H_{23}N_2O$ $[M+H]^+$: 319.1805, found: 319.1796.

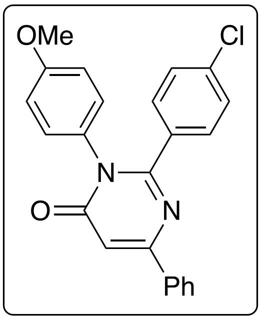

5h

2-(*p*-Chlorophenyl)-3,4-dihydro-3-(*p*-methoxyphenyl)-6-phenylpyrimidin-4-one (5h). Prepared according to General Procedure B from **1a** (89.3 mg, 0.27 mmol) and **4h** (145.3 mg, 0.45 mmol) in DCE (4.0 mL) in 52% yield (54.6 mg, 0.14 mmol); prepared according to General Procedure B from **1a** (100.9 mg, 0.30 mmol) and **4n** (164.2 mg, 0.45 mmol) in DCE (4.0 mL) in 49% yield (57.2 mg, 0.147 mmol) as a brown solid. Mp = 165.0 – 167.2 ºC; Rf = 0.40 (HexH/AcOEt 8:2); ^{1}H NMR (300 MHz, $CDCl_3$) δ 8.08 (dd, J = 6.6, 2.9 Hz, 2H), 7.55 – 7.47 (m, 3H), 7.35 (d, J = 8.2 Hz, 2H), 7.26 (at, J = 8.0 Hz, 2H), 7.09 (d, J = 8.4 Hz, 2H), 7.02 (s, 1H), 6.89 (d, J = 8.3 Hz, 2H), 3.82 (s, 3H). ^{13}C NMR (75 MHz, $CDCl_3$) δ 163.3 (s), 160.1 (s), 159.6 (s), 158.3 (s), 136.1 (s), 136.0 (s), 133.7 (s), 130.9 (d), 130.8 (d, 2CH), 129.7 (d, 2CH), 129.6 (s), 128.9 (d, 2CH), 128.4 (d, 2CH), 127.2 (d, 2CH), 114.6 (d, 2CH), 108.2 (d), 55.5 (q); HRMS (ESI): (*m/z*) calculated for $C_{23}H_{18}ClN_2O_2$ $[M+H]^+$: 389.1051, found: 389.1050.

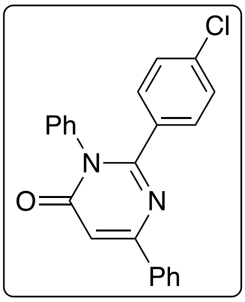

5i

2-(*p*-Chlorophenyl)-3,4-dihydro-3,6-diphenylpyrimidin-4-one (5i). Prepared according to General Procedure B from **1a** (100.0 mg, 0.30 mmol) and **4i** (155.76 mg, 0.45 mmol) in DCE (4.5 mL) in 57% yield (61.2 mg, 0.17 mmol) as a red solid. Mp = 127.0 – 129.8 ºC; Rf = 0.1 (HexH/AcOEt 8:2); ^{1}H NMR (300 MHz, $CDCl_3$) δ 7.98 (d, *J* = 8.5 Hz, 2H), 7.57 – 7.48 (m, 1H), 7.47 – 7.36 (m, 6H), 7.31 – 7.22 (m, 3H), 7.12 – 6.79 (m, 3H); ^{13}C NMR (75 MHz, $CDCl_3$) δ 163.1 (s), 160.2 (s), 158.1 (s), 137.2 (s), 136.16 (s), 136.12 (s), 133.6 (s), 131.0 (d), 130.9 (d, 2CH), 129.4 (d, 2CH), 129.0 (d), 129.0 (d, 2CH), 128.8 (d, 2CH), 128.4 (d, 2CH), 127.3 (d, 2CH), 108.3 (d); HRMS (ESI): (*m/z*) calculated for $C_{22}H_{16}ClN_2O$ $[M+H]^+$: 359.0946, found: 359,0939.

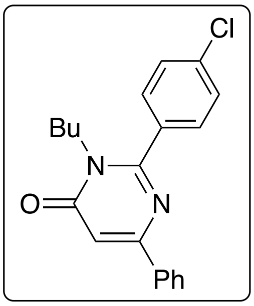

5j

3-(*n*-Butyl)-2-(*p*-chlorophenyl)-3,4-dihydro-6-phenylpyrimidin-4-one (5j). Prepared according to General Procedure B from **1a** (16.5 mg, 0.05 mmol) and **4j** (24.2 mg, 0.07 mmol) in DCE (4.0 mL) in 72% yield (12.2 mg, 0.036 mmol) as a yellowish solid. Mp = 132.0 – 135.1 ºC; Rf = 0.25 (HexH/AcOEt 8:2); ^{1}H NMR (300 MHz, $CDCl_3$) δ 8.11 – 7.87 (m, 2H), 7.56 – 7.50 (m, 4H), 7.49 – 7.42 (m, 3H), 6.90 (s, 1H), 4.04 – 3.91 (m, 2H), 1.69 – 1.56 (m, 2H), 1.30 – 1.14 (m, 2H), 0.82 (t, *J* = 7.3 Hz, 3H). ^{13}C NMR (75 MHz, $CDCl_3$) δ 163.0 (s), 159.7 (s), 159.3 (s), 136.4 (s), 136.2 (s), 133.8 (s), 130.7 (d), 129.6 (d, 2CH), 129.1 (d, 2CH), 128.9 (d, 2CH), 127.2 (d, 2CH), 108.3 (d), 45.9 (t), 30.9 (t), 20.0 (t), 13.6 (q); HRMS (ESI): (*m/z*) calculated for $C_{20}H_{20}ClN_2O$ $[M+H]^+$: 339.1259, found: 339.1257.

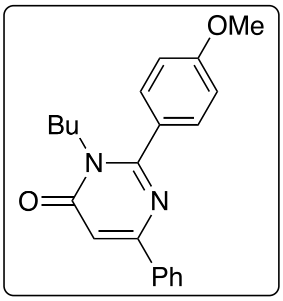

5k

3-(n-Butyl)-3,4-dihydro-2-(p-methoxyphenyl)-6-phenylpyrimidin-4-one (5k). Prepared according to General Procedure B from **1a** (100.0 mg, 0.30 mmol) and **4k** (144.7 mg, 0.45 mmol) in DCE (11.8 mL) in 53% yield (140.0 mg, 0.42 mmol) as a brown oil. Rf = 0.15 (HexH/AcOEt 8:2); ^{1}H NMR (300 MHz, $CDCl_3$) δ 8.09 – 7.90 (m, 2H), 7.53 (d, J = 8.7 Hz, 2H), 7.49 – 7.41 (m, 3H), 7.04 (d, J = 8.8 Hz, 2H), 6.88 (s, 1H), 4.11 – 3.96 (m, 2H), 3.91 (s, 3H), 1.72 – 1.54 (m, 2H), 1.23 (heptet, J = 7.4 Hz, 2H), 0.82 (t, J = 7.3 Hz, 3H); ^{13}C NMR (75 MHz, $CDCl_3$) δ 163.4 (s), 160.9 (s), 160.2 (s), 159.6 (s), 136.4 (s), 130.5 (d), 129.7 (d, 2CH), 128.7 (d, 2CH), 127.8 (s), 127.1 (d, 2CH), 114.1 (d, 2CH), 107.8 (d), 55.5 (q), 45.9 (t), 30.8 (t), 20.0 (t), 13.6 (q); HRMS (ESI): (m/z) calculated for $C_{21}H_{23}N_2O_2$ $[M+H]^+$: 335.1754, found: 335.1747.

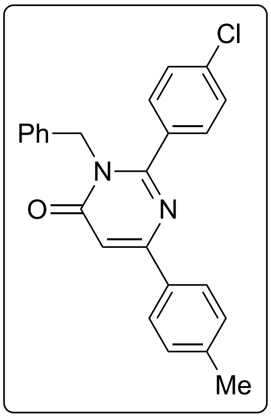

5l

3-Benzyl-2-(p-chlorophenyl)-3,4-dihydro-6-(p-tolyl)pyrimidin-4-one (5l). Prepared according to General Procedure B from **1b** (171.2 mg, 0.53 mmol) and **4a** (291.0 mg, 0.80 mmol) in DCE (8.0 mL) in 51% yield (101.2 mg, 0.27 mmol) as a brown oil. Rf = 0.10 (HexH/AcOEt 8:2); ^{1}H NMR (300 MHz, CDC13) δ 7.53 – 7.46 (m, 2H), 7.40 – 7.35 (m, 2H), 7.33-7.25 (m, 7H), 7.07-6.96 (s+m, 3H), 5.26 (s, 2H), 2.49 (s, 3H). ^{13}C NMR (75 MHz, $CDCl_3$) δ 163.3 (s), 160.0 (s), 159.6 (s), 141.3 (s), 136.5 (s), 136.3 (s), 133.6 (s), 133.2 (s), 129.71 (d, 2CH), 129.65 (d, 2CH), 128.9 (d, 4CH), 127.8 (d), 127.2 (d, 2CH), 126.9 (d, 2CH), 107.5 (d), 48.8 (t), 21.6 (q); HRMS (ESI): (m/z) calculated for $C_{24}H_{20}ClN_2O$ $[M+H]^+$: 387.1259, found: 387.1251.

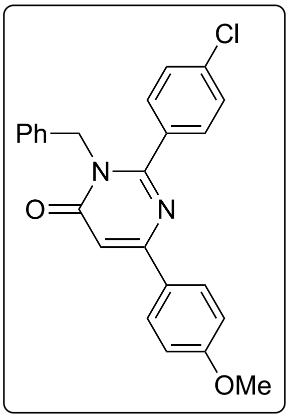

5m

3-Benzyl-2-(*p*-chlorophenyl)-3,4-dihydro-6-(*p*-methoxyphenyl)pyrimidin-4-one (5m). Prepared according to General Procedure B from **1c** (205.4 mg, 0.56 mmol) and **4a** (305.2 mg, 0.84 mmol) in DCE (8.4 mL) in 66% yield (148.1 mg, 0.37 mmol) as a yellow solid. Mp = 118.2 – 119.6 ºC; Rf = 0.13 (HexH/AcOEt 8:2); ^{1}H NMR (300 MHz, CDCl3) δ 7.99 (d, *J* = 8.9 Hz, 2H), 7.39 (d, *J* = 8.7 Hz, 2H), 7.35 – 7.21 (m, 6H), 7.01 – 6.96 (m, 3H), 6.92 (s, 1H), 5.23 (s, 2H), 3.88 (s, 3H); ^{13}C NMR (75 MHz, $CDCl_3$) δ 163.3 (s), 162.0 (s), 159.6 (s), 159.5 (s), 136.5 (s), 136.4 (s), 133.6 (s), 129.7 (d, 2CH), 128.9 (d, 6CH), 128.4 (s), 127.7 (d), 126.9 (d, 2CH), 114.3 (d, 2CH), 106.6 (d), 55.6 (q), 48.8 (t); HRMS (ESI): (*m/z*) calculated for $C_{24}H_{20}ClN_2O_2$ $[M+H]^+$: 403.1208, found: 403.1202.

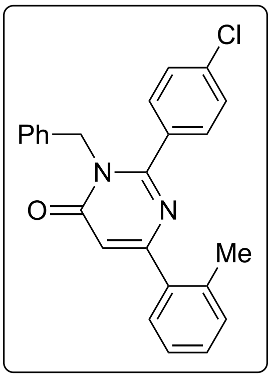

5n

3-Benzyl-2-(*p*-chlorophenyl)-3,4-dihydro-6-(*o*-tolyl)pyrimidin-4-one (5n). Prepared according to General Procedure B from **1e** (113.3 mg, 0.32 mmol) and **4a** (176.1 mg, 0.49 mmol) in 47% yield (58.8 mg, 0.15 mmol) as a yellowish oil. Rf = 0.25 (HexH/AcOEt 8:2); ^{1}H NMR (300 MHz, $CDCl_3$) δ 7.50 (dd, *J* = 7.7, 1.8 Hz, 1H), 7.43 – 7.19 (m, 10H), 7.14 – 6.96 (m, 2H), 6.68 (s, 1H), 5.26 (s, 2H), 2.49 (s, 3H). ^{13}C NMR (75 MHz, $CDCl_3$) δ 163.3 (s) , 162.8 (s), 159.4 (s), 137.3 (s), 136.6 (s), 136.2 (s), 136.1 (s),

133.3 (s), 131.2 (d), 129.6 (d, 3CH), 129.3 (d), 128.9 (d, 4CH), 127.8 (d), 127.0 (d, 2CH), 126.2 (d), 113.0 (d), 49.0 (t), 20.8 (q); HRMS (ESI): (*m/z*) calculated for $C_{24}H_{20}ClN_2O$ $[M+H]^+$: 387.1259, found: 387.1254.

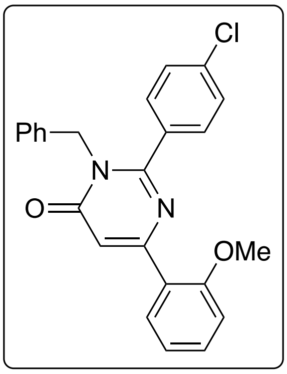

5o

3-Benzyl-2-(*p*-chlorophenyl)-3,4-dihydro-6-(*o*-methoxyphenyl)pyrimidin-4-one (5o). Prepared according to General Procedure B from **1f** (151.0 mg, 0.40 mmol) and **4a** (217.7 mg, 0.60 mmol) in DCE (6.0 mL) in 56% yield (90.2 mg, 0.22 mmol) as a yellow oil. Rf = 0.1 (HexH/AcOEt 8:2); ^{1}H NMR (300 MHz, $CDCl_3$) δ 8.06 (dd, *J* = 7.7, 1.9 Hz, 1H), 7.54 – 7.21 (m, 9H), 7.10 – 6.97 (m, 4H), 5.24 (s, 2H), 3.96 (s, 3H); ^{13}C NMR (75 MHz, $CDCl_3$) δ 163.4 (s), 158.8 (s), 158.4 (s), 157.4 (s), 136.44 (s), 136.41 (s), 133.7 (s), 131.6 (d), 131.1 (d), 129.7 (d, 2CH), 128.9 (d, 4CH), 127.7 (d), 127.0 (d, 2CH), 125.1 (s), 120.9 (d), 113.5 (d), 111.5 (d), 55.7 (q), 48.80 (t); HRMS (ESI): (*m/z*) calculated for $C_{24}H_{20}ClN_2O_2$ $[M+H]^+$: 403.1208, found: 403.1205.

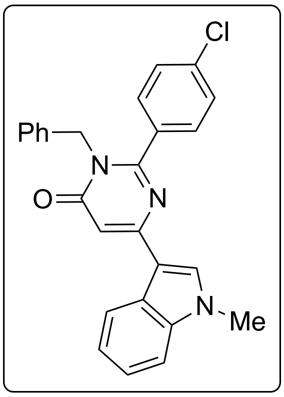

5p

3-Benzyl-2-(*p*-chlorophenyl)-3,4-dihydro-6-(*N*-methylindol-3-yl)pyrimidin-4-one (5p). Prepared according to General Procedure B from **1j** (234.8 mg, 0.60 mmol) and **4a** (326.6 mg, 0.90 mmol) in DCE (9.0 mL) in 32% yield (82.0 mg, 0.19 mmol) as a brown

solid. Mp = 145.3 – 148.7 ºC; Rf = 0.08 (HexH/AcOEt 8:2); ^{1}H NMR (300 MHz, $CDCl_3$) δ 8.16 (dd, J = 6.8, 1.6 Hz, 1H), 7.88 (s, 1H), 7.45 – 7.36 (m, 3H), 7.35 – 7.30 (m, 2H), 7.30 – 7.23 (m, 5H), 7.01 (dd, J = 7.3, 2.2 Hz, 2H), 6.95 (s, 1H), 5.24 (s, 2H), 3.86 (s, 3H). ^{13}C NMR (75 MHz, $CDCl_3$) δ 163.3 (s), 159.1 (s), 157.3 (s), 138.2 (s), 136.7 (s), 136.3 (s), 133.9 (s) , 132.6 (d), 129.7 (d, 2CH), 128.8 (d, 4CH), 127.6 (d), 126.9 (d, 2CH), 125.7 (s), 122.8 (d), 121.6 (d), 121.1 (d), 113.0 (s), 110.1 (d), 105.0 (d), 48.5 (t), 33.4 (q). HRMS (ESI): (*m/z*) calculated for $C_{26}H_{21}ClN_3O$ [M+H]$^+$: 426.1368, found: 426.1373.

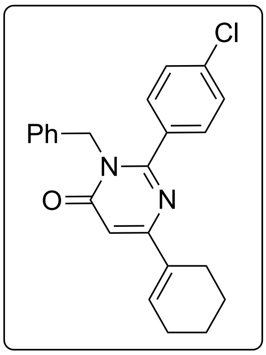

5q

3-Benzyl-2-(*p*-chlorophenyl)-6-(cyclohex-1-en-1-yl)-3,4-dihydropyrimidin-4-one (5q). Prepared according to General Procedure B from **1g** (99.2 mg, 0.29 mmol) and **4a** (158.7 mg, 0.44 mmol) in DCE (4.4 mL) in 39% yield (43.2 mg, 0.11 mmol) as a yellowish oil. Rf = 0.18 (HexH/AcOEt 8:2); ^{1}H NMR (300 MHz, $CDCl_3$) δ ^{1}H NMR (300 MHz, $CDCl_3$) δ 7.36 (d, J = 8.6 Hz, 2H), 7.32 – 7.20 (m, 5H), 7.17 (bs, 1H), 7.05 – 6.87 (m, 2H), 6.47 (s, 1H), 5.18 (s, 2H), 2.39 – 2.31 (m, 2H), 2.30 – 2.24 (m, 2H), 1.82 – 1.76 (m, 2H), 1.73 – 1.65 (m, 2H); ^{13}C NMR (75 MHz, $CDCl_3$) δ 163.7 (s), 160.2 (s), 158.2 (s), 136.5 (s), 136.4 (s), 134.5 (s), 133.4 (d), 133.7 (s), 129.6 (d, 2CH), 128.8 (d, 4CH), 127.7 (d), 126.8 (d, 2CH), 106.2 (d), 48.6 (t), 26.2 (t), 25.0 (t), 22.6 (t), 21.9 (t). HRMS (ESI): (*m/z*) calculated for $C_{23}H_{22}ClN_2O$ [M+H]$^+$: 377.1413, found: 377.1415.

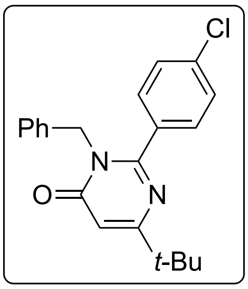

5r

3-Benzyl-6-(*t*-butyl)-2-(*p*-chlorophenyl)-3,4-dihydropyrimidin-4-one (5r). Prepared according to General Procedure B from **1i** (234.8 mg, 0.60 mmol) and **4a** (326.6 mg, 0.90 mmol). in DCE (9.0 mL) in 38% yield (80.7 mg, 0.23 mmol) as a brown oil. Rf = 0.35 (HexH/AcOEt 8:2); ^{1}H NMR (300 MHz, $CDCl_3$) δ 7.35 (d, *J* = 8.4 Hz, 2H), 7.32 – 7.21 (m, 5H), 6.97 (dd, *J* = 7.1, 2.3 Hz, 2H), 6.52 (s, 1H), 5.17 (s, 2H), 1.30 (s, 9H). ^{13}C NMR (75 MHz, $CDCl_3$) δ 173.8 (s), 163.5 (s), 158.5 (s), 136.4 (s), 136.3 (s), 133.6 (s), 129.8 (d, 2CH), 128.8 (d, 2CH), 128.7 (d, 2CH), 127.6 (d), 126.8 (d, 2CH), 107.8 (d), 48.7 (t), 37.1 (s), 28.7 (q, $3CH_3$). HRMS (ESI): (*m/z*) calculated for $C_{21}H_{21}ClN_2NaO$ $[M+Na]^+$: 375.1235, found: 375.1244.

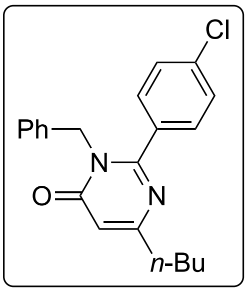

5s

3-Benzyl-6-*n*-butyl-2-(*p*-chlorophenyl)-3,4-dihydropyrimidin-4-one (5s). Prepared according to General Procedure B from **1h** (102.3 mg, 0.32 mmol) and **4a** (176.1 mg, 0.49 mmol) in DCE (4.8 mL) in 64% yield (72.6 mg, 0.21 mmol) as a yellowish oil. Rf = 0.30 (HexH/AcOEt 8:2); ^{1}H NMR (300 MHz, CDCl3) δ 7.42 – 7.33 (m, 2H), 7.32 – 7.18 (m, 5H), 7.07 – 6.85 (m, 2H), 6.41 (s, 1H), 5.17 (s, 2H), 2.69 – 2.49 (m, 2H), 1.77 – 1.62 (m, 2H), 1.43 (h, *J* = 7.3 Hz, 2H), 0.97 (t, *J* = 7.3 Hz, 3H). ^{13}C NMR (75 MHz, CDCl3) δ 166.9 (s), 162.7 (s), 159.5 (s), 136.5 (s), 136.2 (s), 133.2 (s), 129.5 (d, 2CH), 129.0 (d, 2CH), 128.8 (d, 2CH), 127.7 (d), 126.8 (d, 2CH), 111.0 (d), 48.7 (t) , 37.2 (t), 30.1 (t), 22.5 (t), 14.0 (q); HRMS (ESI): (*m/z*) calculated for $C_{21}H_{22}ClN_2O$ $[M+H]^+$: 353.1415, found: 353.1409.

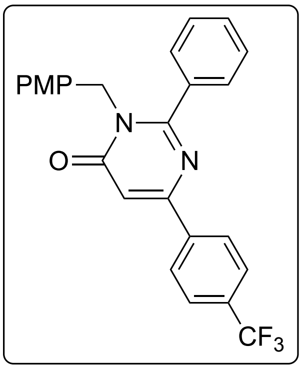

5t

3,4-Dihydro-3-(*p*-methoxybenzyl)-2-phenyl-6-[*p*-(trifluoromethyl)phenyl]pyrimidin-4-one (5t). Prepared according to General Procedure B from **1d** (185.6 mg, 0.46 mmol) and **4m** (247.3 mg, 0.69 mmol) in 78% yield (156.0 mg, 0.36 mmol) as a yellowish oil. Rf = 0.50 (HexH/AcOEt 8:2); ^{1}H NMR (300 MHz, $CDCl_3$) δ 8.15 (d, J = 8.1 Hz, 2H), 7.73 (d, J = 8.1 Hz, 2H), 7.39 (d, J = 8.4 Hz, 2H), 7.36 – 7.17 (m, 3H), 7.09 – 6.88 (m, 4H), 5.32 (s, 2H), 3.88 (s, 3H); ^{13}C NMR (75 MHz, $CDCl_3$) δ 163.3 (C), 161.3 (C), 161.1 (C), 158.4 (C), 139.7 (C), 136.6 (C), 132.2 (q, J_{CF} = 32.7 Hz, C), 130.0 (2CH), 129.7 (C), 128.8 (2CH), 127.7 (CH), 127.6 (2CH), 126.9 (2CH), 125.7 (q, J_{CF} = 4.0 Hz, 2CH), 120.3 (q, J_{CF} = 287.6 Hz, CF_3), 114.0 (2CH), 108.8 (CH), 55.5 (CH_3), 49.2 (CH_2); ^{19}F NMR (282 MHz, $CDCl_3$) δ –62.8; HRMS (ESI): (*m/z*) calculated for $C_{25}H_{20}F_3N_2O_2$ $[M+H]^+$: 437.1471, found: 437.1473.

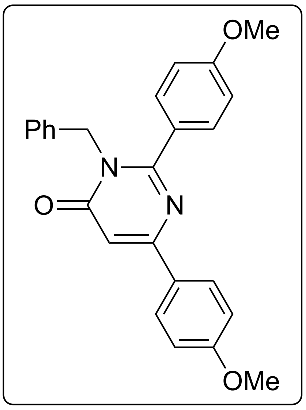

5u

3-Benzyl-3,4-dihydro-2,6-bis(*p*-methoxyphenyl)pyrimidin-4-one (5u). Prepared according to General Procedure B from **1c** (157.7 mg, 0.40 mmol) and **4b** (215.0 mg, 0.60 mmol) in DCE (6 mL) in 51% yield (87.7 mg, 0.22 mmol) as a yellow solid. Mp = 129.2 – 133.2 ºC; Rf = 0.10 (HexH/AcOEt 8:2); ^{1}H NMR (300 MHz, $CDCl_3$) δ 8.01 (d,

J = 8.9 Hz, 2H), 7.37 (d, J = 8.8 Hz, 2H), 7.29 – 7.25 (m, 3H), 7.05 – 7.01 (m, 2H), 7.00 – 6.85 (m, 5H), 5.29 (s, 2H), 3.87 (s, 3H), 3.87 (s, 3H); ^{13}C NMR (75 MHz, $CDCl_3$) δ 163.7 (s), 161.8 (s), 161.1 (s), 160.4 (s), 159.6 (s), 136.7 (s, 2C), 130.0 (d, 2CH), 128.9 (d, 2CH), 128.8 (d, 2CH), 127.7 (s), 127.5 (d), 126.9 (d, 2CH), 114.2 (d, 2CH), 113.9 (d, 2CH), 106.1 (d), 55.6 (q), 55.5 (q), 49.0 (t); HRMS (ESI): (*m/z*) calculated for $C_{25}H_{23}N_2O_3$ $[M+H]^+$: 399.1703, found: 399.1699.

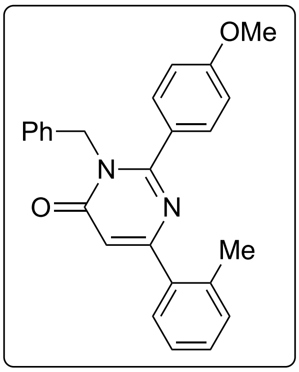

5v

3-Benzyl-3,4-dihydro-2-(4-methoxyphenyl)-6-(*o*-tolyl)pyrimidin-4-one (5v). Prepared according to General Procedure B from **1e** (128,1 mg, 0.40 mmol) and **4b** (215.1 mg, 0.60 mmol) in DCE (6.0 mL) in 42% yield (64.3 mg, 0.17 mmol) as a yellow solid. Mp = 121.7 – 123.5 ºC; Rf = 0.10 (HexH/AcOEt 8:2); ^{1}H NMR (300 MHz, $CDCl_3$) δ 7.51 (dd, J = 7.8, 1.8 Hz, 1H), 7.45 – 7.17 (m, 8H), 7.06 (dd, J = 7.6, 1.9 Hz, 2H), 6.90 (d, J = 8.8 Hz, 2H), 6.64 (s, 1H), 5.31 (s, 2H), 3.85 (s, 3H), 2.51 (s, 3H); ^{13}C NMR (75 MHz, $CDCl_3$) δ 163.3 (s), 163.2 (s), 161.2 (s), 160.4 (s), 137.7 (s), 136.6 (s), 136.1 (s), 131.1 (d), 123.0 (d, 2CH), 129.4 (d), 129.3 (d), 128.8 (d, 2CH), 127.6 (d), 127.4 (s), 127.0 (d, 2CH), 126.1 (d), 114.0 (d, 2CH), 112.4 (d), 55.5 (q), 49.2 (t), 20.8 (q); HRMS (ESI): (*m/z*) calculated for $C_{25}H_{23}N_2O_2$ $[M+H]^+$: 383.1754, found: 383.1754.

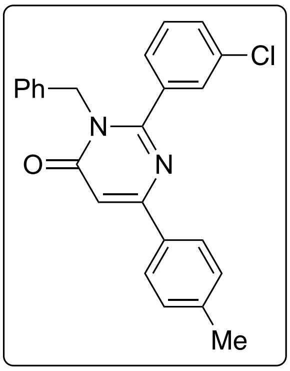

5w

3-Benzyl-2-(*m*-chlorophenyl)-3,4-dihydro-6-(*p*-tolyl)pyrimidin-4-one (5w). Prepared according to General Procedure B from **1b** (152.8 mg, 0.40 mmol) and **4f** (217.7 mg, 0.60 mmol) in DCE (6.0 mL) in 48% yield (74.3 mg, 0.19 mmol) as a yellow oil. Rf = 0.5 (HexH/AcOEt 8:2); ^1H NMR (300 MHz, $CDCl_3$) δ 8.28 (d, *J* = 8.1 Hz, 1H), 7.56 (s, 1H), 7.39 (d, *J* = 8.3 Hz, 3H), 7.32 – 7.21 (m, 7H), 6.95 (bs, 2H), 5.27 (s, 2H), 2.54 (s, 3H). ^{13}C NMR (75 MHz, $CDCl_3$) δ 162.4 (s), 155.5 (s), 147.4 (s), 145.9 (s), 136.6 (s), 136.2 (s), 133.9 (s), 129.7 (d), 129.6 (d, 2CH), 129.1 (d), 128.9 (d, 2 CH), 128.8 (d, 2CH), 127.7 (d), 127.5 (d), 127.1 (d), 126.90 (s), 126.88 (d, 2CH), 118.5 (d), 48.8 (t), 22.1 (q). HRMS (ESI): (*m/z*) calculated for $C_{24}H_{20}ClN_2O$ $[M+H]^+$: 387.1259, found: 387.1258.

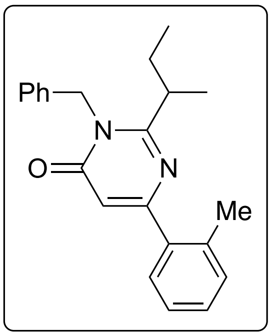

5x

3-Benzyl-2-(*s*-butyl)-3,4-dihydro-6-(*o*-tolyl)pyrimidin-4-one (5x). Prepared according to General Procedure B from **1e** (136.6 mg, 0.39 mmol) and **4g** (182.0 mg, 0.59 mmol) in DCE (5.8 mL) in 42% yield (54.5 mg, 0.16 mmol) as a yellow oil. Rf = 0.30 (HexH/AcOEt 8:2); ^1H NMR (300 MHz, $CDCl_3$) δ 7.50 (dd, *J* = 7.8, 1.8 Hz, 1H), 7.44 – 7.01 (m, 8H), 6.57 (s, 1H), 5.55 (d, *J* = 15.7 Hz, 1H), 5.35 (d, *J* = 15.7 Hz, 1H), 2.91 (sextet, *J* = 6.8 Hz, 1H), 2.50 (s, 3H), 1.96 – 1.74 (m, 1H), 1.64 – 1.42 (m, 1H), 1.19 (d, *J* = 6.7 Hz, 3H), 0.73 (t, *J* = 7.4 Hz, 3H); ^{13}C NMR (75 MHz, $CDCl_3$) δ 165.9 (s), 163.7 (s), 163.4 (s), 137.9 (s), 136.5 (s), 136.3 (s), 131.2 (d), 129.4 (d), 129.3 (d), 129.0 (d, 2CH), 127.8 (d), 126.7 (d, 2CH), 126.1 (d), 111.2 (d), 45.9 (t), 39.5 (d), 28.8 (t), 21.0 (q), 19.5 (q), 12.1 (q); HRMS (ESI): (*m/z*) calculated for $C_{22}H_{25}N_2O$ $[M+H]^+$: 333.1961, found: 333.1960.

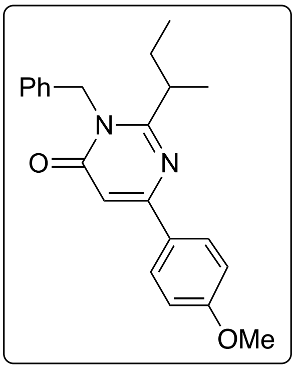

5y

3-Benzyl-2-(*s*-butyl)-3,4-dihydro-6-(*p*-methoxyphenyl)pyrimidin-4-one (5y). Prepared according to General Procedure B from **1c** (117.2 mg, 0.32 mmol) and **4g** (148.0 mg, 0.48 mmol) in DCE (4.8 mL) in 61% yield (68.0 mg, 0.20 mmol) as a yellow oil. Rf = 0.10 (HexH/AcOEt 8:2); ^{1}H NMR (300 MHz, $CDCl_3$) δ 8.03 (d, *J* = 8.9 Hz, 2H), 7.46 – 7.23 (m, 3H), 7.23 – 7.13 (m, 2H), 7.05 – 6.94 (m, 2H), 6.81 (s, 1H), 5.50 (d, *J* = 15.8 Hz, 1H), 5.34 (d, *J* = 15.8 Hz, 1H), 3.88 (s, 3H), 2.86 (sextet, *J* = 6.5 Hz, 1H), 1.99 – 1.83 (m, 1H), 1.67 – 1.47 (m, 1H), 1.21 (d, *J* = 6.6 Hz, 3H), 0.75 (t, *J* = 7.4 Hz, 3H); ^{13}C NMR (75 MHz, $CDCl_3$) δ 165.9 (s), 163.8 (s), 161.8 (s), 159.4 (s), 136.5 (s), 129.03 (s), 128.96 (d, 2CH), 128.7 (d, 2CH), 127.7 (d), 126.5 (d, 2CH), 114.1 (d, 2CH), 104.8 (d), 55.5 (q), 45.6 (t), 39.4 (d), 28.9 (t), 19.6 (q), 12.0 (q); HRMS (ESI): (*m/z*) calculated for $C_{22}H_{25}N_2O_2$ $[M+H]^+$: 349.1911, found: 349.1909.

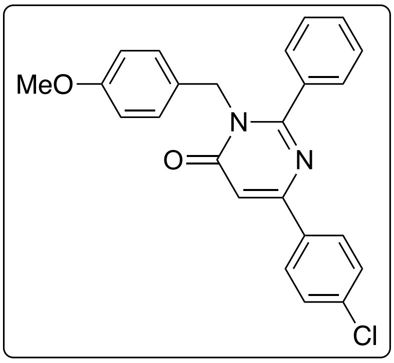

5z

6-(*p*-Chlorophenyl)-3,4-dihydro-3-(*p*-methoxybenzyl)-2-phenylpyrimidin-4-one (5z). Prepared according to General Procedure B from **1k** (152.8 mg, 0.40 mmol) and **4m** (215.1 mg, 0.60 mmol) in DCE (6.0 mL) in 50% yield (87.5 mg, 0.20 mmol) as a yellow oil. Rf = 0.10 (HexH/AcOEt 8:2); ^{1}H NMR (300 MHz, $CDCl_3$) δ 7.98 (d, *J* = 8.7 Hz, 2H), 7.44 (d, *J* = 8.6 Hz, 2H), 7.37 (d, *J* = 8.7 Hz, 2H), 7.33 – 7.20 (m, 3H), 7.12 –

6.82 (m, 5H), 5.30 (s, 2H), 3.88 (s, 3H); ^{13}C NMR (75 MHz, $CDCl_3$) δ 163.4 (s), 161.2 (s), 160.8 (s), 158.7 (s), 136.9 (s), 136.5 (s), 134.8 (s), 130.0 (d, 2CH), 129.0 (d, 2CH), 128.8 (d, 2CH), 128.5 (d, 2CH), 127.6 (d), 127.4 (s), 126.9 (d, 2CH), 114.0 (d, 2CH), 107.7 (d), 55.6 (q), 49.1 (t); HRMS (ESI): (*m/z*) calculated for $C_{24}H_{20}ClN_2O_2$ $[M+H]^+$: 403.1208, found: 403.1216.

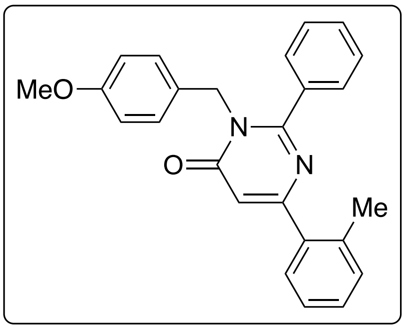

5aa

3,4-Dihydro-3-(*p*-methoxybenzyl)-6-(*o*-methylphenyl)-2-phenylpyrimidin-4-one (5aa). Prepared according to General Procedure B from **1e** (151.0 mg, 0.40 mmol) and **4m** (215.1 mg, 0.60 mmol) in DCE (6.0 mL) in 68% yield (113.4 mg, 0.27 mmol) as a yellow solid. Mp = 131.5 – 134.0 ºC; Rf = 0.10 (HexH/AcOEt 8:2); ^{1}H NMR (300 MHz, $CDCl_3$) δ 7.52 (dd, J = 7.7, 1.8 Hz, 1H), 7.40 – 7.21 (m, 8H), 7.07 (dd, J = 7.4, 2.0 Hz, 2H), 6.90 (d, J = 8.8 Hz, 2H), 6.65 (s, 1H), 5.32 (s, 2H), 3.85 (s, 3H), 2.51 (s, 3H); ^{13}C NMR (75 MHz, $CDCl_3$) δ 163.3 (s), 163.2 (s), 161.2 (s), 160.4 (s), 137.7 (s), 136.6 (s), 136.1 (s), 131.1 (d), 123.0 (d, 2CH), 129.4 (d), 129.3 (d), 128.8 (d, 2CH), 127.6 (d), 127.4 (s), 127.0 (d, 2CH), 126.1 (d), 114.0 (d, 2CH), 112.4 (d), 55.6 (q), 49.2 (t), 20.8 (q). HRMS (ESI): (*m/z*) calculated for $C_{25}H_{23}N_2O_2$ $[M+H]^+$: 383.1753, found: 383.1747.

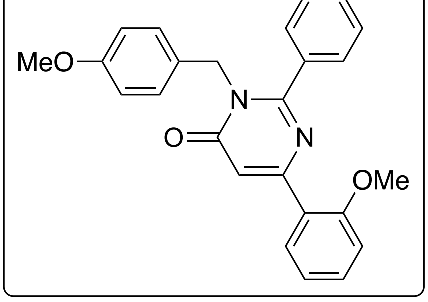

5ab

2-(*p*-Chlorophenyl)-3,4-dihydro-3-(*p*-methoxybenzyl)-6-(*o*-methoxyphenyl)pyrimidin-4-one (5ab). Prepared according to General Procedure B

from **1f** (146.4 mg, 0.40 mmol) and **4m** (215.1 mg, 0.60 mmol) in DCE (6.0 mL) in 76% yield (131.6 mg, 0.30 mmol) as a yellow oil. Rf = 0.10 (HexH/AcOEt 8:2); ^{1}H NMR (300 MHz, $CDCl_3$) δ 8.08 (dd, *J* = 7.8, 1.8 Hz, 1H), 7.55 – 7.12 (m, 7H), 7.03 (dd, *J* = 12.2, 7.6 Hz, 4H), 6.90 (d, *J* = 8.7 Hz, 2H), 5.28 (s, 2H), 3.95 (s, 3H), 3.86 (s, 3H); ^{13}C NMR (75 MHz, $CDCl_3$) δ 163.7 (s), 161.0 (s), 159.8 (s), 158.3 (s), 157.4 (s), 136.8 (s), 131.4 (d), 131.1 (d), 130.0 (d, 2CH), 128.7 (d, 2CH), 127.8 (s), 127.5 (d), 127.1 (d, 2CH), 125.4 (s), 120.8 (d), 113.9 (d, 2CH), 113.0 (d), 111.5 (d), 55.7 (q), 55.6 (q), 49.0 (t); HRMS (ESI): (*m/z*) calculated for $C_{25}H_{23}N_2O_3$ $[M+H]^+$: 399.1703, found: 399.1705.

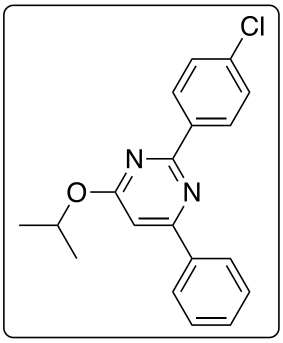

7

Synthesis of 2-(*p*-chlorophenyl)-4-isopropoxy-6-phenylpyrimidine (7). Prepared according to General Procedure B from **6** (109 mg, 0.30 mmol) and **4a** (163 mg, 0.45 mmol) in DCE (6.0 mL) in 23% yield (22 mg, 0.068 mmol) as a colourless oil. Rf = 0.10 (HexH/AcOEt 8:2); ^{1}H NMR (300 MHz, $CDCl_3$) δ 8.51 (d, *J* = 8.6 Hz, 2H), 8.23 – 8.08 (m, 2H), 7.66 – 7.39 (m, 6H), 6.98 (s, 1H), 5.60 (hept, *J* = 6.2 Hz, 1H), 1.46 (d, *J* = 6.2 Hz, 6H). ^{13}C NMR (75 MHz, $CDCl_3$) δ 170.2 (s), 165.0 (s), 163.1 (s), 137.4 (s), 136.8 (s), 136.7 (s), 130.6 (d), 129.8 (d, 2CH), 128.9 (d, 2CH), 128.8 (d, 2CH), 127.2 (d, 2CH), 102.1 (d), 69.5 (d), 22.1 (q, $2CH_3$); HRMS (ESI): (*m/z*) calculated for $C_{19}H_{18}ClN_2O$ $[M+H]^+$: 325.1102, found: 325.1106.

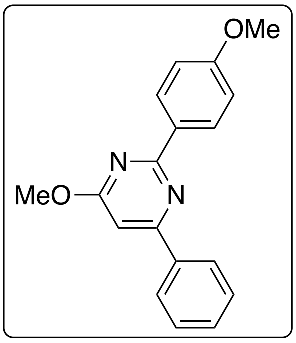

8

Synthesis of 4-methoxy-2-(*p*-methoxyphenyl)-6-phenylpyrimidine (8). Prepared according to General Procedure B from **1a** (67.2 mg, 0.20 mmol) and **4n** (80.5 mg, 0.30 mmol) in DCE (6.0 mL) in 58% yield (33.9 mg, 0.116 mmol) as a colourless oil. Prepared according to General Procedure B from **1a** (33.6 mg, 0.10 mmol) and **9a** (30.9 mg, 0.15 mmol) in DCE (3.0 mL) in 60% yield (17.3 mg, 0.060 mmol) as a colourless oil. Rf = 0.50 (HexH/AcOEt 8:2); ^{1}H NMR (300 MHz, $CDCl_3$) δ 8.56 (d, J = 8.9 Hz, 2H), 8.23 – 8.11 (m, 2H), 7.56 – 7.46 (m, 3H), 7.02 (d, J = 8.9 Hz, 2H), 6.97 (s, 1H), 4.12 (s, 3H), 3.90 (s, 3H); ^{13}C NMR (75 MHz, $CDCl_3$) δ 170.8 (s), 164.8 (s), 163.9 (s), 161.9 (s), 137.6 (s), 130.7 (s), 130.5 (d), 130.1 (d, 2CH), 128.9 (d, 2CH), 127.2 (d, 2CH), 113.8 (d, 2CH), 100.5 (d), 55.5 (q), 53.7 (q); HRMS (ESI): (*m/z*) calculated for $C_{18}H_{17}N_2O_2$ $[M+H]^+$: 293.1290, found: 293.1291.

Gram-scale reaction for the synthesis of 3,4-dihydropyrimidin-4-one (5a). 4-Benzyl-3-(4-chlorophenyl)-5-(*p*-tolyl)-4,5-dihydro-1,2,4-oxadiazole **4a** (2.127 g, 5.86 mmol, 1.5 equiv) was added to a solution of pentacarbonyl(1-methoxy-3-phenylprop-2-ynylidene) chromium(0) **1a** (1.314 g, 3.91 mmol, 1 equiv) in DCE (58 mL) in a Schlenk flask, at room temperature under argon atmosphere. The mixture was stirred at 60 ºC until complete disappearance of the Fischer carbene complex was monitored by TLC (48 h). Then, silica gel was added, solvent was evaporated under reduced pressure, and the crude residue was purified by column chromatography on silica gel (Hexane/EtOAc: 20:1 to 4:1) to afford 3,4-dihydropyrimidin-4-one **5a** in 71% yield (1.039 g, 2.787 mmol).

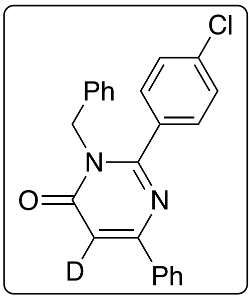

D-5a

Deuteration experiments: Synthesis of 3,4-dihydropyrimidin-4-one D-5a. Experiment 1 (with 1.5 equiv of D_2O). 4,5-Dihydro-1,2,4-oxadiazole **4a** (217.7 mg, 0.6 mmol, 1.5 equiv) and D_2O (11 ml, 0.6 mmol, 1.5 equiv) were sequentially added to a solution of chromium methoxy alkynyl Fischer carbene complex **1a** (134.5 mmol, 0.4 mmol, 1 equiv) in DCE (6 mL) in a Schlenk flask, at room temperature under argon atmosphere. The mixture was stirred at 60 ºC until complete disappearance of the Fischer carbene complex was monitored by TLC (36 h). Then, silica gel was added, solvent was evaporated under reduced pressure, and the crude residue was purified by column chromatography on silica gel (Hexane/EtOAc: 20:1 to 4:1) to obtain 119.6 mg (80%) of deuteraded 3,4-dihydropyrimidin-4-one **D-5a**, bearing 60% of deuterium incorporation at position 5. The deuterium percentaje was determined by ^{1}H NMR, by comparing the integral values of the signal at 7.06-6.96 ppm with the signal at 8.12-7.88 ppm. ^{1}H NMR (300 MHz, $CDCl_3$) δ 8.12 – 7.88 (m, 2H), 7.58 – 7.44 (m, 3H), 7.41 (d, J = 8.5 Hz, 3H), 7.36 – 7.21 (m, 5H), 7.06 – 6.96 (m, 2.40 H), 5.26 (s, 2H); ^{13}C NMR (75 MHz, $CDCl_3$) δ 163.2 (s), 159.9 (s), 159.7 (s), 136.6 (s), 136.2 (s), 136.0 (s), 133.5 (s), 130.9 (d), 129.7 (d, 2CH), 128.9 (d, 6CH), 127.8 (d), 127.2 (d, 2CH), 126.89 (d, 2CH), 108.2 (d, 0.4CH), 107,9 (t, 0.6CD, $^2J_{CD}$ = 23.5 Hz), 48.8 (t). ^{2}H NMR (61 MHz, $CDCl_3$) δ 7.02 (bs).

Experiment 2 (with 5 equiv of D_2O). 4,5-Dihydro-1,2,4-oxadiazole **4a** (217.7 mg, 0.6 mmol, 1.5 equiv) and D_2O (36 ml, 2.0 mmol, 5 equiv) were sequentially added to a solution of chromium methoxy alkynyl Fischer carbene complex **1a** (134.5 mmol, 0.4 mmol, 1 equiv) in DCE (6 mL) in a Schlenk flask, at room temperature under argon atmosphere. The mixture was stirred at 60 ºC until complete disappearance of the Fischer carbene complex was monitored by TLC (36 h). Then, silica gel was added, solvent was evaporated under reduced pressure, and the crude residue was purified by column chromatography on silica gel (Hexane/EtOAc: 20:1 to 4:1) to obtain 122.6 mg (82%) of deuteraded 3,4-dihydropyrimidin-4-one **D-5a**, bearing 90% of deuterium incorporation

at position 5, as a brown oil. The deuterium percentaje was determined by ^{1}H NMR, by comparing the integral values of the signal at 7.05-6.93 ppm with the signal at 8.12-7.89 ppm. Rf = 0.20 (HexH/AcOEt 8:2); ^{1}H NMR (401 MHz, $CDCl_3$) δ 8.12 – 7.89 (m, 2H), 7.50 – 7.46 (m, 3H), 7.43 – 7.35 (m, 2H), 7.33 – 7.29 (m, 2H), 7.28 – 7.23 (m, 3H), 7.05 – 6.93 (m, 2.1H), 5.24 (s, 2H); ^{13}C NMR (101 MHz, $CDCl_3$) δ 163.2 (s), 160.0 (s), 159.7 (s), 136.6 (s), 136.3 (s), 136.1 (s), 133.6 (s), 130.9 (d), 129.7 (d, 2CH), 128.9 (d, 6CH), 127.8 (d), 127.3 (d, 2CH), 126.9 (d, 2CH), 108.3 (d, 0.1CH), 108,0 (t, 0.9CD, $^2J_{CD}$ = 27.5 Hz), 48.9 (t); ^{2}H NMR (61 MHz, $CDCl_3$) δ 7.02 (bs). HRMS (ESI): (*m/z*) calculated for $C_{23}H_{17}ClDN_2O$ $[M+H]^+$: 373.1165, found: 374.1151.

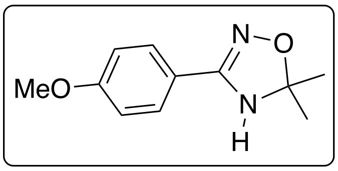

9a

3-(*p*-Methoxyphenyl)-5,5-dimethyl-4,5-dihydro-1,2,4-oxadiazole (9a). Prepared according to a reported procedure[9] in 7.5% yield (78.2 mg, 0.23 mmol) as a white solid. The recovered starting material was subjected again to the same conditions, but the reaction was allowed to proceed for 60 h; an additional 70.6% yield (516.1 mg, 2.50 mmol) was obtained (78.1% combined yield). The spectroscopic data obtained are in agreement with the ones reported in the literature.

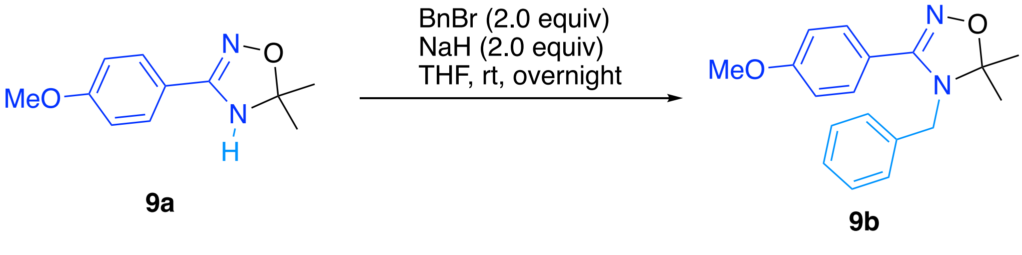

4-Benzyl-3-(*p*-methoxyphenyl)-5,5-dimethyl-4,5-dihydro-1,2,4-oxadiazole (9b). Under an argon atmosphere, sodium hydride (2.0 eq, 99% purity, 183.7 mg, 7.58 mmol) is added to a dry Schlenk flask containing 4,5-dihydro-1,2,4-oxadiazole **9a** (1.0 eq, 0.7796 g, 3.79 mmol) in THF (38 mL) at 0 °C. The mixture is allowed to react for 45 minutes at 0 °C. Then, benzyl bromide (2.0 eq, 0.90 mL, 7.58 mmol) is added and the mixture is allowed to stir at room temperature overnight. Next morning, the reaction is

quenched by adding brine and extracted with ethyl acetate (3 x 25 mL). The combined organic extracts are dried with Na_2SO_4, filtered and the organic solvent is evaporated. The product is further purified by flash column chromatography (Hex/AcOEt; 10:1 to 4:1) to obtain 1.0761 g (96% yield) of a yellowish oil. Rf = 0.20 (HexH/AcOEt 8:2); ^{1}H NMR (300 MHz, $CDCl_3$) δ 7.74 (d, J = 8.4 Hz, 2H), 7.48 – 7.18 (m, 5H), 6.98 (d, J = 8.3 Hz, 2H), 4.45 (s, 2H), 3.84 (s, 3H), 1.42 (s, 6H); ^{13}C NMR (75 MHz, $CDCl_3$) δ 161.9 (s), 161.3 (s), 136.0 (s), 130.1 (d, 2CH), 129.0 (d, 2CH), 128.2 (d, 2CH), 127.7 (d), 120.6 (s), 114.7 (d, 2CH), 106.7 (s), 59.0 (q), 55.3 (t), 28.7 (q, $2CH_3$); HRMS (ESI): (*m/z*) calculated for $C_{18}H_{21}N_2O_2$ $[M+H]^+$: 297.1603, found: 297.1602.

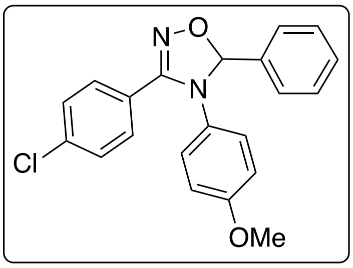

13

3-(*p*-Chlorophenyl)-4-(*p*-methoxyphenyl)-5-phenyl-4,5-dihydro-1,2,4-oxadiazole (13) Prepared according to General Procedure A, but employing benzaldehyde instead of *p*-tolualdehyde, in 63% yield for the cycloaddition step (3007.3 mg, 8.24 mmol) as a brown oil. Rf = 0.30 (HexH/AcOEt 8:2); ^{1}H NMR (300 MHz, $CDCl_3$) δ 7.61 – 7.56 (m, 2H), 7.55 – 7.49 (m, 2H), 7.49 – 7.42 (m, 2H), 7.34 – 7.26 (m, 2H), 6.83 – 6.76 (m, 2H), 6.76 – 6.69 (m, 2H), 6.42 (s, 1H), 3.74 (s, 3H); ^{13}C NMR (75 MHz, $CDCl_3$) δ 158.2 (s), 155.2 (s), 138.7 (s), 136.5 (s), 133.6 (s), 130.0 (d), 129.4 (d, 2CH), 129.0 (d, 2CH), 128.9 (d, 2CH), 127.6 (d, 2CH), 127.2 (d, 2CH), 124.0 (s), 114.8 (d, 2CH), 101.4 (d), 55.5 (q); HRMS (ESI): (*m/z*) calculated for $C_{21}H_{18}ClN_2O_2$ $[M+H]^+$: 365.1051, found: 365.1053.

6. Tables with the 2D-NMR Experiments for Selected Compounds

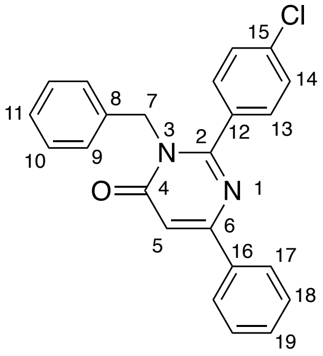

5a [300 MHz ($CDCl_3$)]

	^{13}C NMR	DEPT	^{1}H NMR	COSY	HMBC	NOESY
**1**						
**2**	159.6	C				
**3**						
**4**	163.1	C				
**5**	108.1	CH	7.00 (s, 1H)		4,6,16	17
**6**	159.9	C				
**7**	48.8	CH_2	5.25 (s, 2H)		2,4,8,9	9,13
**8**	136.1	C				
**9**	127.1	CH	8.12 – 7.88 (m, 2H)	10,11	7,11	7,10
**10**	128.8	CH	7.50 – 7.46 (m, 2H)	9,11	9,11	9,11
**11**	130.8	CH	7.50 – 7.46 (m, 1H)	9,10	10	10
**12**	133.4	C				
**13**	129.6	CH	7.33 (d, J = 8.4 Hz, 2H)	14	2,12,14	7,9
**14**	128.8	CH	7.40 (d, J = 8.4 Hz, 2H)	13	2,12,13,15	13
**15**	136.5	C				
**16**	135.9	C				
**17**	126.8	CH	7.00 (d, J = 6.9 Hz, 2H)	18,19	6,16,18,19	5,18
**18**	128.8	CH	7.30 – 7.22 (m, 2H)	17,19	16,17	17,19
**19**	127.7	CH	7.30 – 7.22 (m, 1H)	17,18	16,17	18

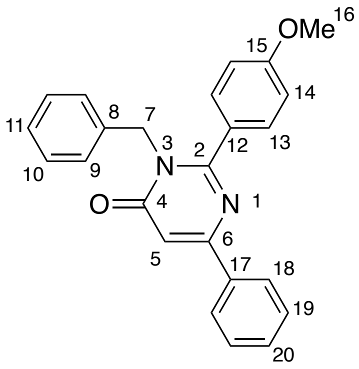

5b [300 MHz ($CDCl_3$)]

	^{13}C NMR	DEPT	^{1}H NMR	COSY	HMBC
**1**					
**2**	160,5	C			
**3**					
**4**	163.5	C			
**5**	107.6	CH	6.97 (s, 1H)		4,6,17
**6**	159,9	C			
**7**	49.0	CH_2	5.31 (s, 2H)		2,4,8,9
**8**	136.5	C			
**9**	126.8	CH	7.04 (d, *J* = 6.1 Hz, 2H)	10,11	7,11
**10**	128.6	CH	7.33 – 7.20 (m, 2H)	9,11	8,9
**11**	127.5	CH	7.33 – 7.20 (m, 1H)	9,10	8,9
**12**	127.4	C			
**13**	129.9	CH	7.38 (d, *J* = 8.4 Hz, 2H)	14	2,14,15
**14**	113.9	CH	6.93 (d, *J* = 8.3 Hz, 2H)	13	12,15
**15**	161.1	C			
**16**	55.5	CH_3	3.87 (s, 3H)		15
**17**	136.2	C			
**18**	127.1	CH	8.08 – 7.99 (m, 2H)	19,20	6,20
**19**	128.7	CH	7.51 – 7.44 (m, 2H)	18,20	17,18
**20**	130.6	CH	7.51 – 7.44 (m, 1H)	18,19	17,18

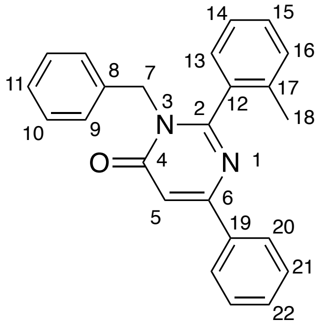

5d [300 MHz ($CDCl_3$)]

	^{13}C NMR	DEPT	^{1}H NMR	COSY	HMBC
**1**					
**2**	160.2	C			
**3**					
**4**	163.3	C			
**5**	108.2	CH	7.02 (s, 1H)		4,6,7,19
**6**	160.0	C			
**7**	47.9	CH_2	5.17 (s, 2H)		2,4,8,9
**8**	136.1	C			
**9**	127.8	CH	6.95 – 6.80 (m, 2H)	10	7,8
**10**	128.4	CH	7.29 – 7.08 (m, 2H)	9,11	-[a]
**11**	125,9	CH	7.29 – 7.08 (m, 1H)	10	-[a]
**12**	134.5/135.9	C			
**13**	129,9	CH	7.44 – 7.36 (m, 1H)	14	12,14,17
**14**	127,7	CH	7.29 – 7.08 (m, 1H)<br>Aprox 7.25	13	-[a]
**15**	127.7	CH	7.29 – 7.08 (m, 1H)<br>Aprox 7.16		-[a]
**16**	130.6	CH	7.29 – 7.08 (m, 1H)<br>Aprox 7.26		-[a]
**17**	134.5/135.9	C			
**18**	19.0	CH_3	1.98 (s, 2H)		2,12,16,17
**19**	136.2	C			
**20**	127.2	CH	8.07 – 7.98 (m, 2H)	21	6,19,22
**21**	128.7	CH	7.51 – 7.45 (m, 2H)	20, 22	19
**22**	130.6	CH	7.51 – 7.45 (m, 1H)	21	19

[a] HMBC signals are not clear for these protons because of overlapping.

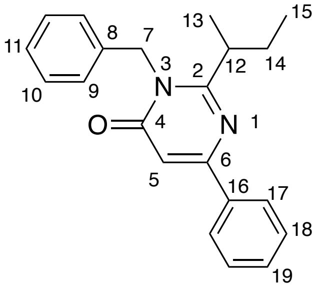

5g [300 MHz ($CDCl_3$)]

	^{13}C NMR	DEPT	^{1}H NMR	COSY	HMBC
**1**					
**2**	166.1	C			
**3**					
**4**	163.6	C			
**5**	106.3	CH	6.90 (s, 1H)		4,6,7,16
**6**	159.6	C			
**7**	45.6	CH_2	5.52 (d, *J* = 15.9 Hz, 1H)<br>5.36 (d, *J* = 15.9 Hz, 1H)	7	2,4, 8, 9
**8**	136.3	C			
**9**	126.5	CH	7.21 (d, *J* = 6.6 Hz, 2H)	10,11	7, 11
**10**	128.9	CH	7.41 – 7.23 (m, 2H)	9,11	8
**11**	127.6	CH	7.41 – 7.23 (m, 1H)	9,10	9
**12**	39.4	CH	3.02 – 2.75 (m, 1H)	13,14	2,13,14,15
**13**	19.5	CH_3	1.23 (d, *J* = 6.6 Hz, 3H)	12	2,12,14
**14**	28.8	CH_2	2.10 – 1.80 (m, 1H)<br>1.71 – 1.45 (m, 1H)	12,14,15	2,12,13,15
**15**	11.9	CH_3	0.76 (t, *J* = 7.4 Hz, 3H)	14	12,14
**16**	136.5	C			
**17**	127.0	CH	8.15 – 8.02 (m, 2H)	18,19	6,16,19
**18**	128.7	CH	7.56 – 7.41 (m, 2H)	17,19	6,16,17
**19**	130.5	CH	7.56 – 7.41 (m, 1H)	17,18	17,18

7. X-Ray structure of 4,5-dihydro-1,2,4-oxadiazole 5c

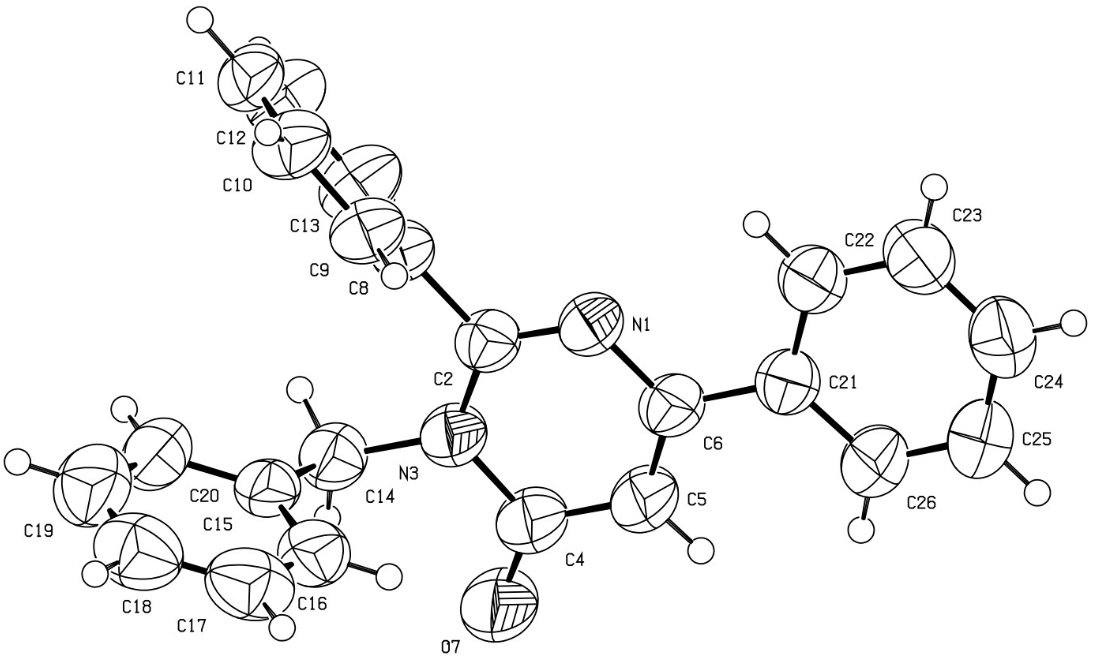

5c (ellipsoid contour 50% probability levels)

Description of the sample preparation. Suitable crystals were obtained by the vapor diffusion technique, which allowed cristal growth by dissolving the compound in a minimum amount of CH_2Cl_2 in close proximity to a hexane reservoir.

Instrumentation used for the crystal measurement. A full data set was collected from a suitable single brown plate-shaped crystal (*0.05 mm · 0.10 mm·0.28 mm*) at *297 K* using the oscillation method (*1.20°* frame width and variable exposure time) on a Rigaku (Oxford Diffraction) Xcalibur Nova diffractometer equipped with microfocus Cu K_{α} radiation *(λ=1.54184 Å*) source and Onyx CCD detector. Data was processed using CrysAlisPro software and an empirical (ABSPACK) absorption correction was applied.

**Empirical formula:** $C_{23}H_{18}N_2O$		**Formula weight:** 338.14
**Temperature:** 297.0 K		**Wavelength:** 1.54184 Å.0 K
**Crystal system, space group:**		Monoclinic, P2(1)/c
**Unit cell dimensions:**	a = 12.2026 (3) Å	α = 90.0 deg.
	b = 5.6291(2) Å	β = 92.225(2) deg.
	c = 25.8032 (7) Å	γ = 90.0 deg.

**Volume:**	1771.08(9) $Å^3$	**Z, Calculated density:**	2, 1.087 Mg/m^3
**F(000):**	476	**Absorption coefficient:**	1.299 mm^{-1}
**Crystal size:**			0.05x0.1x0.28 mm
**Theta range for data collection:**			-43.0 to 94.50 deg.
**Limiting indices:**			-14<=h<=14, -6<=k<=6, -26<=l<=26
**Reflections collected / unique: :**			11255 / 4953 [R(int) = 0.0247]
**Completeness to theta = 97.27**			99.4 %
**Absorption correction:**			Semi-empirical from equivalents
**Max. and min. Transmission:**			1.00000 and 0.6974
**Refinement method:**			Full-matrix least-squares on F^2
**Data / restraints / parameters:**			3154 / 0 / 235
**Goodness-of-fit on F^2:**			1.039
**Final R indices [I>4σ(I)]:**			R_1 = 0.0460, wR_2 = 0.1444
**R indices (all data):**			R_1 = 0.0570, wR_2 = 0.1444
**Largest diff. peak and hole:**			0.147 and -0.149 $e.A^{-3}$

8. References

[1] Zhao, Y.; Thrular, D. G. The M06 suite of density functionals for main group thermochemistry, thermochemical kinetics, noncovalent interactions, excited states, and transition elements: Two new functionals and systematic testing of four M06-class functionals and 12 other functionals *Theor. Chem. Acc.*, **2008**, *120*, 215−241.

[2] Frisch, M. J.; Trucks, G. W.; Schlegel, H. B.; Scuseria, G. E.; Robb, M. A.; Cheeseman, J. R.; Scalmani, G.; Barone, V.; Mennucci, B.; Petersson, G. A.; Nakatsuji, H.; Caricato, M.; Li, X.; Hratchian, H. P.; Izmaylov, A.; Ishida, M.; Nakajima, T.; Honda, Y.; Kitao, O.; Nakai, H.; Vreven, T.; Montgomery Jr., J. A.; Peralta, J. E.; Ogliaro, F.; Bearpark, M.; Heyd, J. J.; Brothers, E.; Kudin, K. N.; Staroverov, V. N.; Kobayashi, R.; Normand, J.; Raghavachari, K.; Rendell, A.; Burant, J. C.; Iyengar, S. S.; Tomasi, J.; Cossi, M.; Rega, N.; Millam, J. M.; Klene, M.; Knox, J. E.; Cross, J. B.; Bakken, V.; Adamo, C.; Jaramillo, J.; Gomperts, R.; Stratmann, R. E.; Yazyev, O.; Austin, A. J.; Cammi, R.; Pomelli, C.; Ochterski, J. W.; Martin, R. L.; Morokuma, K.; Zakrzewski, V. G.; Voth, G. A.; Salvador, P.; Dannenberg, J. J.; Dapprich, S.; Daniels, A. D.; Farkas, O.; Foresman, J. B.; Ortiz, J. V.; Cioslowski, J.; Fox, D. J. *Gaussian 09, Revision A.1,* Gaussian, Inc., Wallingford CT, **2009**.

[3] Hehre W. J. Ab Initio Molecular Orbital Theory. New York: John Wiley & Sons; 1986.

[4] Hay, P. J.; Wadt, W. R. Ab initio effective core potentials for molecular calculations. Potentials for the transition metal atoms Sc to Hg *J. Chem. Phys.* **1985**, *82*, 270−283.

[5] McQuarrie D. A. Statistical Mechanics. New York: Harper & Row; 1975.

[6] (a) García-García, P.; Novillo, C.; Fernández-Rodríguez, M. A.; Aguilar, E. Competitive pathways in the reaction of lithium oxy-*ortho*-quinodimethanes and Fischer alkoxy alkynyl carbene complexes: Synthesis of highly functionalised seven-membered benzocarbocycles *Chem. Eur. J.* **2011***, 17*, 564 – 571. (b) Fernández-Rodríguez, M. A.; Andina, F.; García-García, P.; Rocaboy, C.; Aguilar, E. Multicomponent cCascade reactions triggered by cycloaddition of Fischer alkoxy alkynyl carbene complexes with strained bicyclic olefins *Organometallics* **2009**, 28, 361 – 369.

[7] Chiou, S.; Shine, H. J. A simplified procedure for preparing 3,5-disubstituted-1,2,4-oxadiazoles by reaction of amidoximes with acyl chlorides in pyridine solution *J. Heterocycl. Chem.* **1989**, *26*, 125 – 128.

[8] Xu, W.; Wang, G.; Sun, N.; Liu, Y. Gold-catalyzed formal [3 + 2] cycloaddition of ynamides with 4,5-dihydro-1,2,4-oxadiazoles: synthesis of functionalized 4-aminoimidazoles *Org. Lett.* **2017**, 1*9*, 3307 – 3310.

[9] Wang, A.; Lv, P.; Liu, Y. 4,5-Dihydro-1,2,4-oxadiazole as a single nitrogen transfer reagent: Synthesis of functionalized isoxazoles assisted by $Sc(OTf)_3$ or Au(I)/$Sc(OTf)_3$ synergistic catalysis *Org. Lett.* **2023**, *25*, 4377 − 4382.

[10] D. D. Perrin, W. L. F. Armarego, D. R. Perrin, *Purification of Laboratory Chemicals*, Pergamon Press, 3rd Ed., 1998.

[11] Alcaide, B.; Mardomingo, C. L.; Plumet, J.; Cativiela, C.; Mayoral, J. A. Orbital control in the 1,3-dipolar cycloaddition of benzonitrile oxide to benzylideneanilines *Can. J. Chem*. **1987**, *65*, 2050 – 2056.

[12] Zheng, H.; McDonald, R.; Hall, D. G. Boronic acid catalysis for mild and selective [3+2] dipolar cycloadditions to unsaturated carboxylic acids *Chem. Eur. J.* **2010**, *16*, 5454 – 5460.

[13] Schaufelberger, F.; Hu, L.; Ramström, O. *trans*-Symmetric dynamic covalent systems: Connected transamination and transimination reactions *Chem. Eur. J.* **2015**, *21*, 9776 – 9783.

[14] Miralinaghi, P.; Salimi, M.; Amirhamzeh, A.; Norouzi, M.; Kandelousi, H. M.; Shafiee, A.; Amini, M. Synthesis, molecular docking study, and anticancer activity of triaryl-1,2,4-oxadiazole *Med. Chem. Res.* **2013**, *22*, 4253 – 4262.

9. Copies of ^{1}H-, ^{2}H-, ^{13}C-NMR and 2D-NMR Spectra

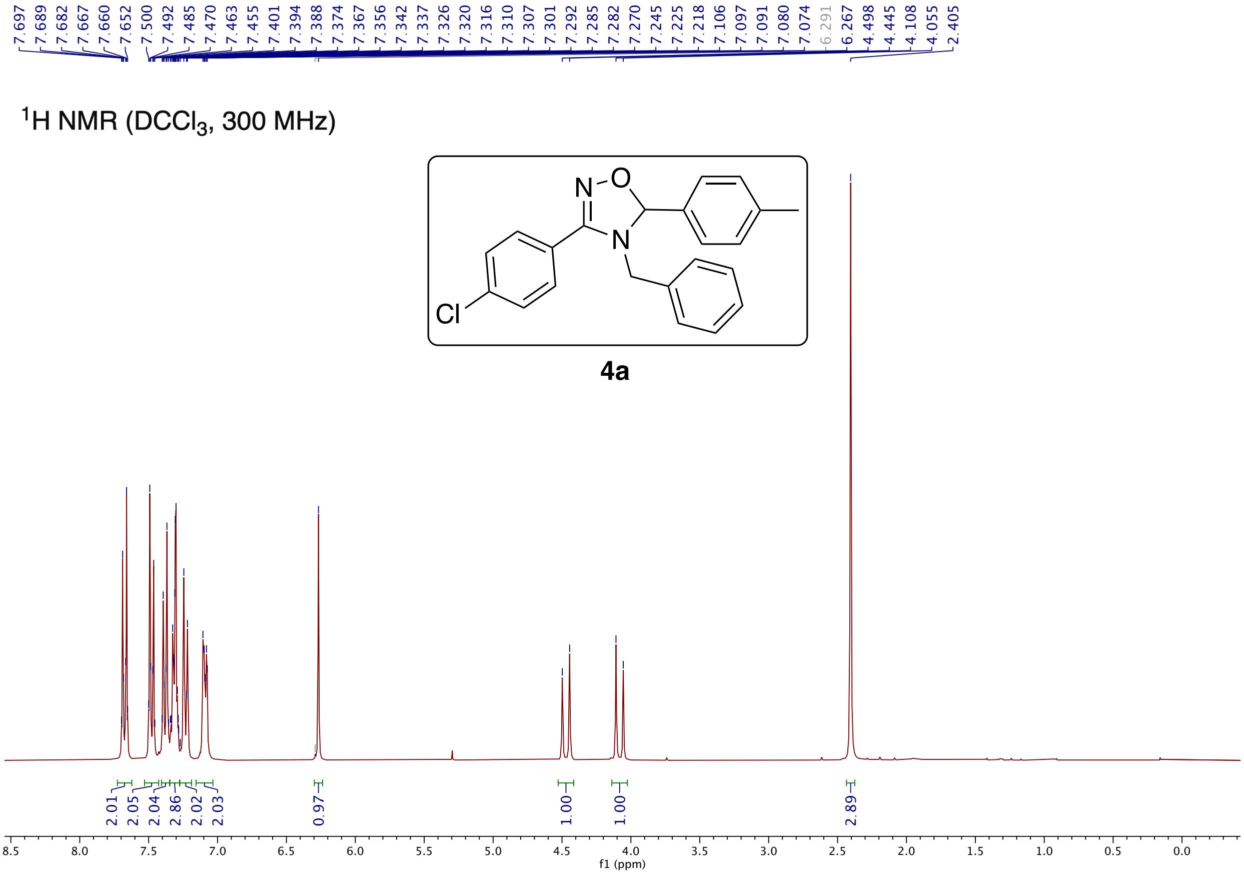
7.697
7.689
7.682
7.667
7.660
7.652
7.500
7.492
7.485
7.470
7.463
7.455
7.401
7.394
7.388
7.374
7.367
7.356
7.342
7.337
7.326
7.320
7.316
7.310
7.307
7.301
7.292
7.285
7.282
7.270
7.245
7.225
7.218
7.106
7.097
7.091
7.080
7.074
6.291
6.267
4.498
4.445
4.108
4.055
2.405
1H NMR (DCCl3, 300 MHz)
N
O
N
Cl
4a
2.01
2.05
2.04
2.86
2.02
2.03
0.97
1.00
1.00
2.89
f1 (ppm)

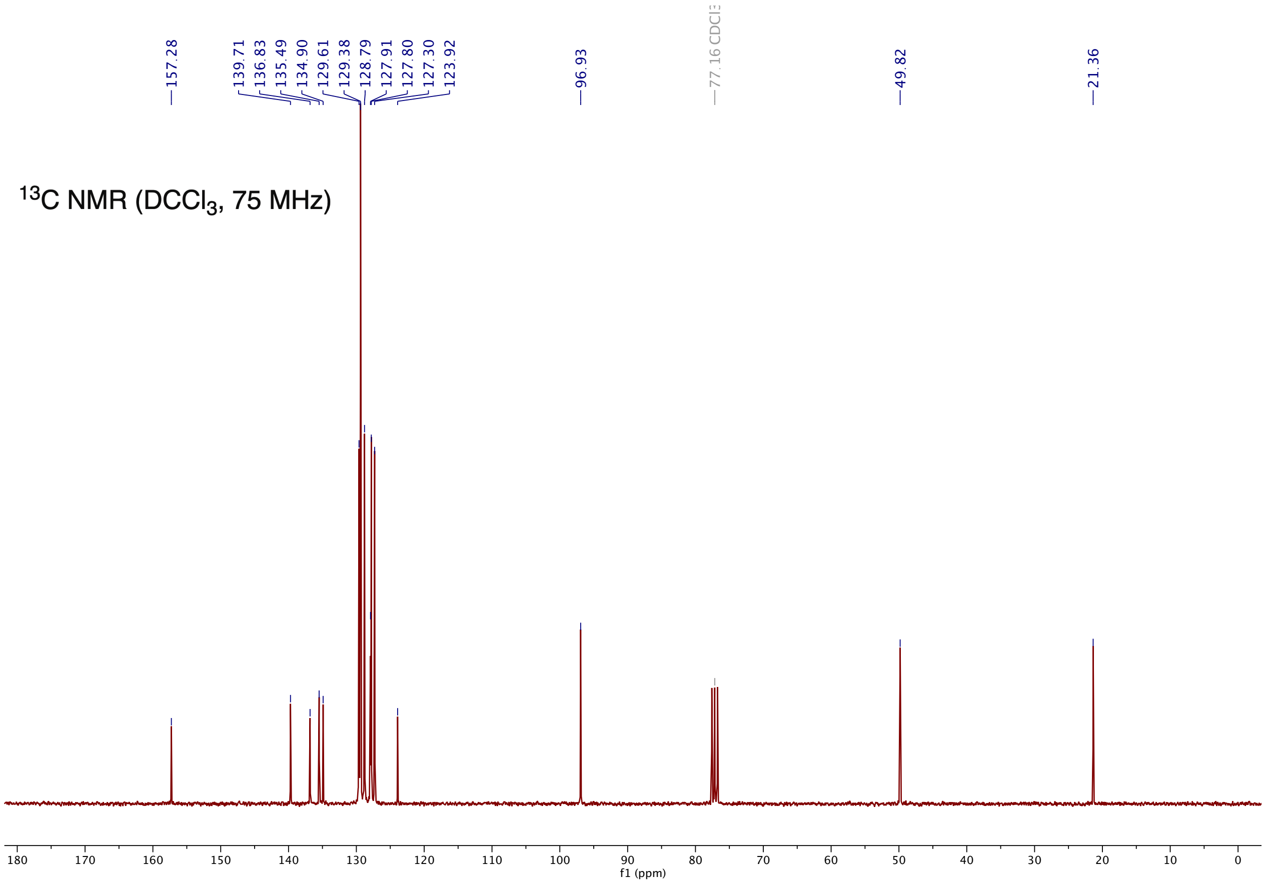
157.28
139.71
136.83
135.49
134.90
129.61
129.38
128.79
127.91
127.80
127.30
123.92
96.93
77.16 CDCl3
49.82
21.36
13C NMR (DCCl3, 75 MHz)
f1 (ppm)

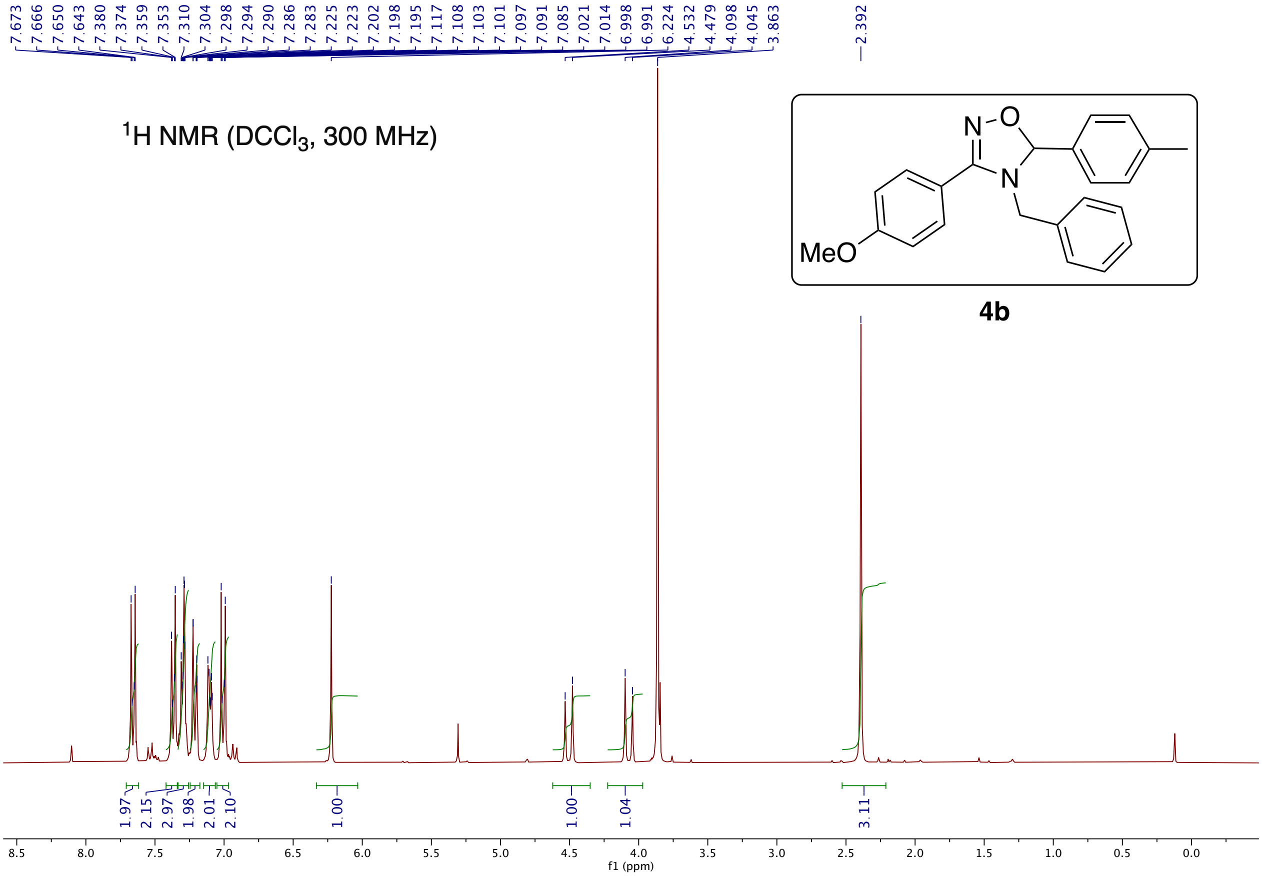
7.673
7.666
7.650
7.643
7.380
7.374
7.359
7.353
7.310
7.304
7.298
7.294
7.290
7.286
7.283
7.225
7.223
7.202
7.198
7.195
7.117
7.108
7.103
7.101
7.097
7.091
7.085
7.021
7.014
6.998
6.991
6.224
4.532
4.479
4.098
4.045
3.863
2.392
1H NMR (DCCl3, 300 MHz)
N
O
N
MeO
4b
1.97
2.15
2.97
1.98
2.01
2.10
1.00
1.00
1.04
3.11
f1 (ppm)

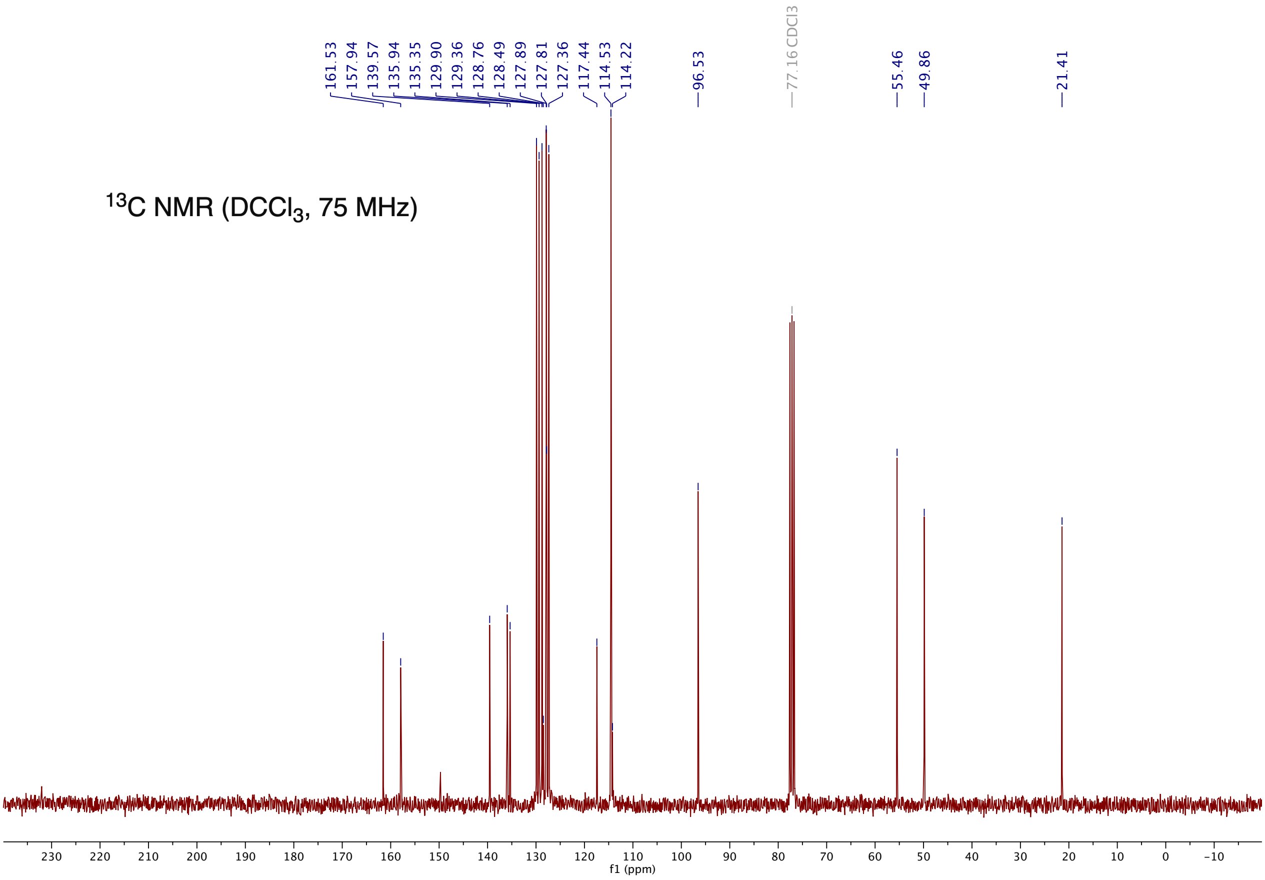
161.53
157.94
139.57
135.94
135.35
129.90
129.36
128.76
128.49
127.89
127.81
127.36
117.44
114.53
114.22
96.53
77.16 CDCl3
55.46
49.86
21.41
13C NMR (DCCl3, 75 MHz)
f1 (ppm)

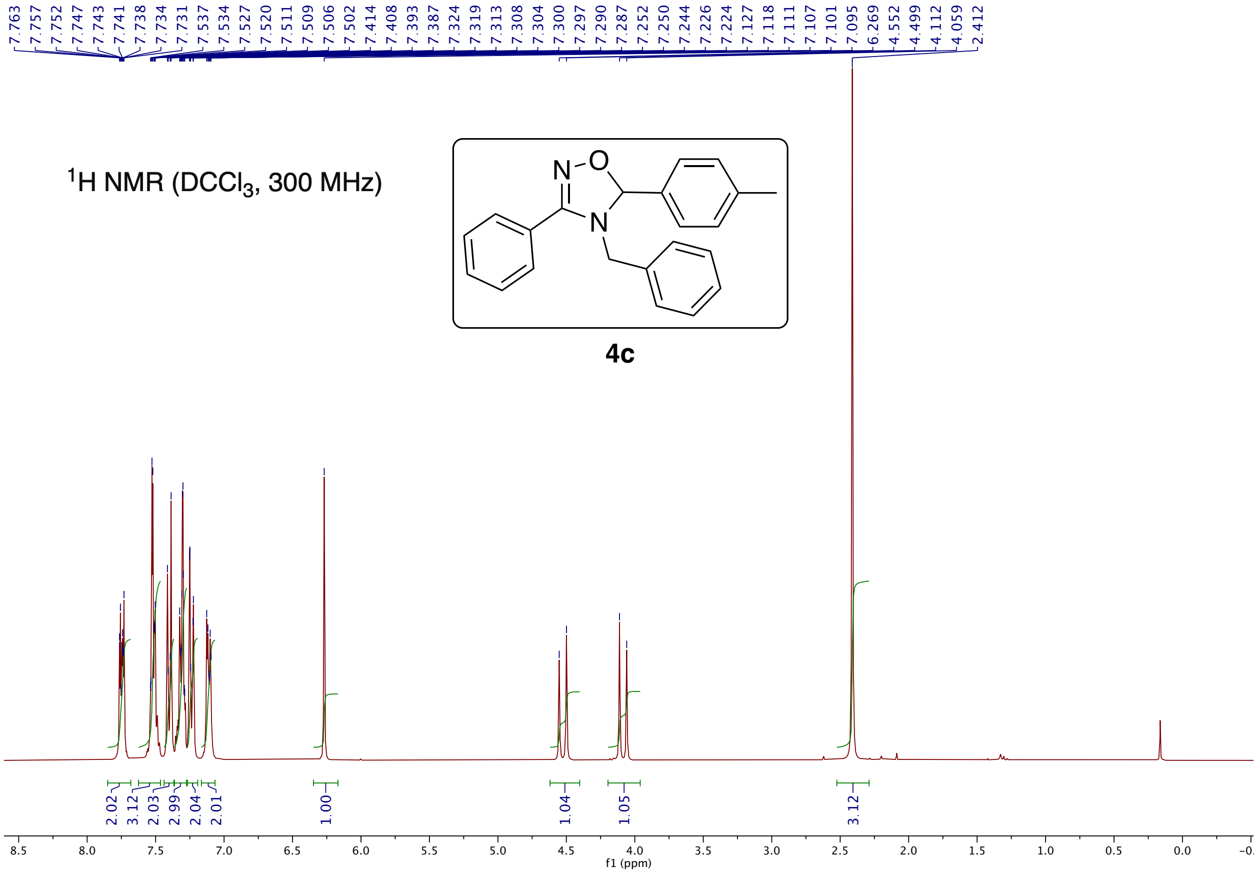
7.763
7.757
7.752
7.747
7.743
7.741
7.738
7.734
7.731
7.537
7.534
7.527
7.520
7.511
7.509
7.506
7.502
7.414
7.408
7.393
7.387
7.324
7.319
7.313
7.308
7.304
7.300
7.297
7.290
7.287
7.252
7.250
7.244
7.226
7.224
7.127
7.118
7.111
7.107
7.101
7.095
6.269
4.552
4.499
4.112
4.059
2.412
^{1}H NMR (DCCl$_3$, 300 MHz)
4c
2.02
3.12
2.03
2.99
2.04
2.01
1.00
1.04
1.05
3.12
f1 (ppm)

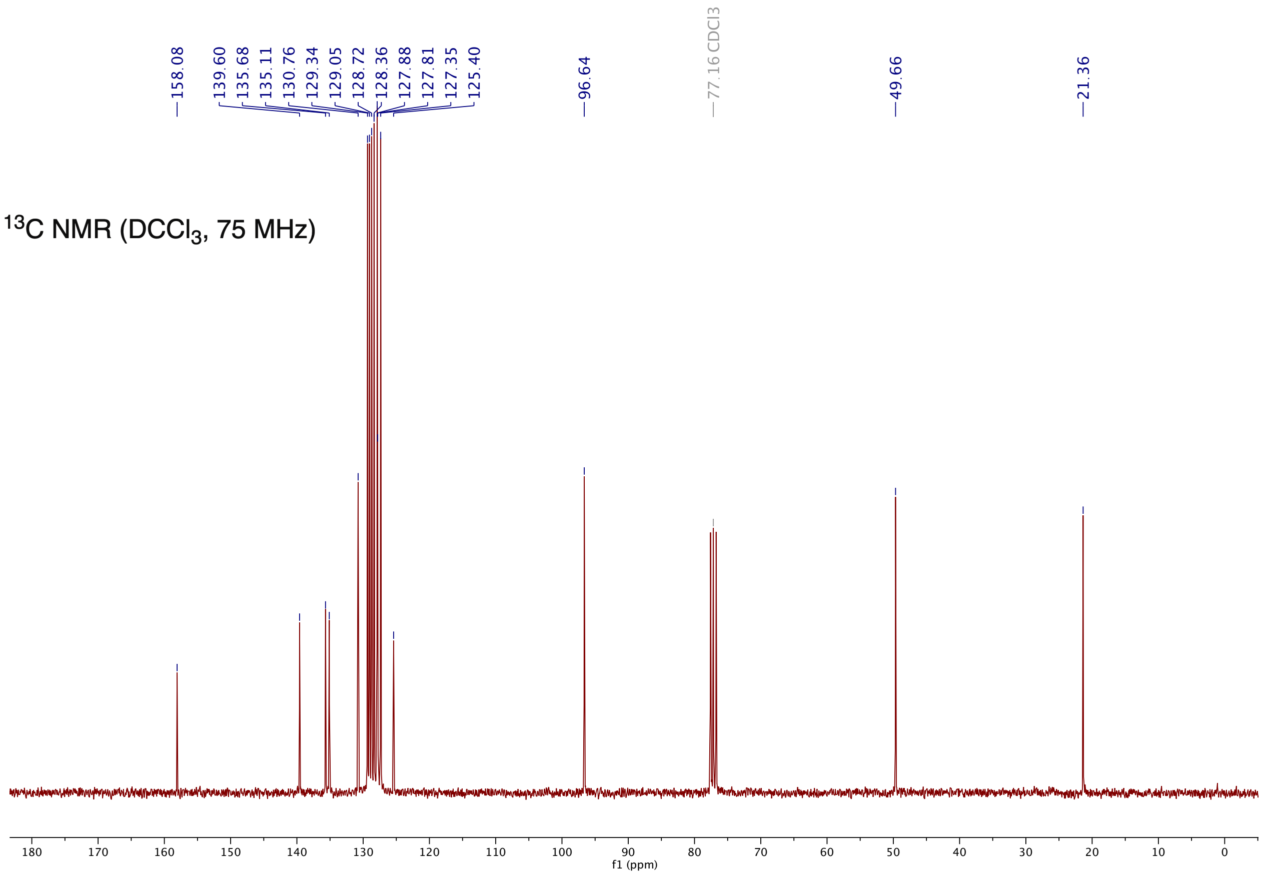
158.08
139.60
135.68
135.11
130.76
129.34
129.05
128.72
128.36
127.88
127.81
127.35
125.40
96.64
77.16 CDCl3
49.66
21.36
^{13}C NMR (DCCl$_3$, 75 MHz)
f1 (ppm)

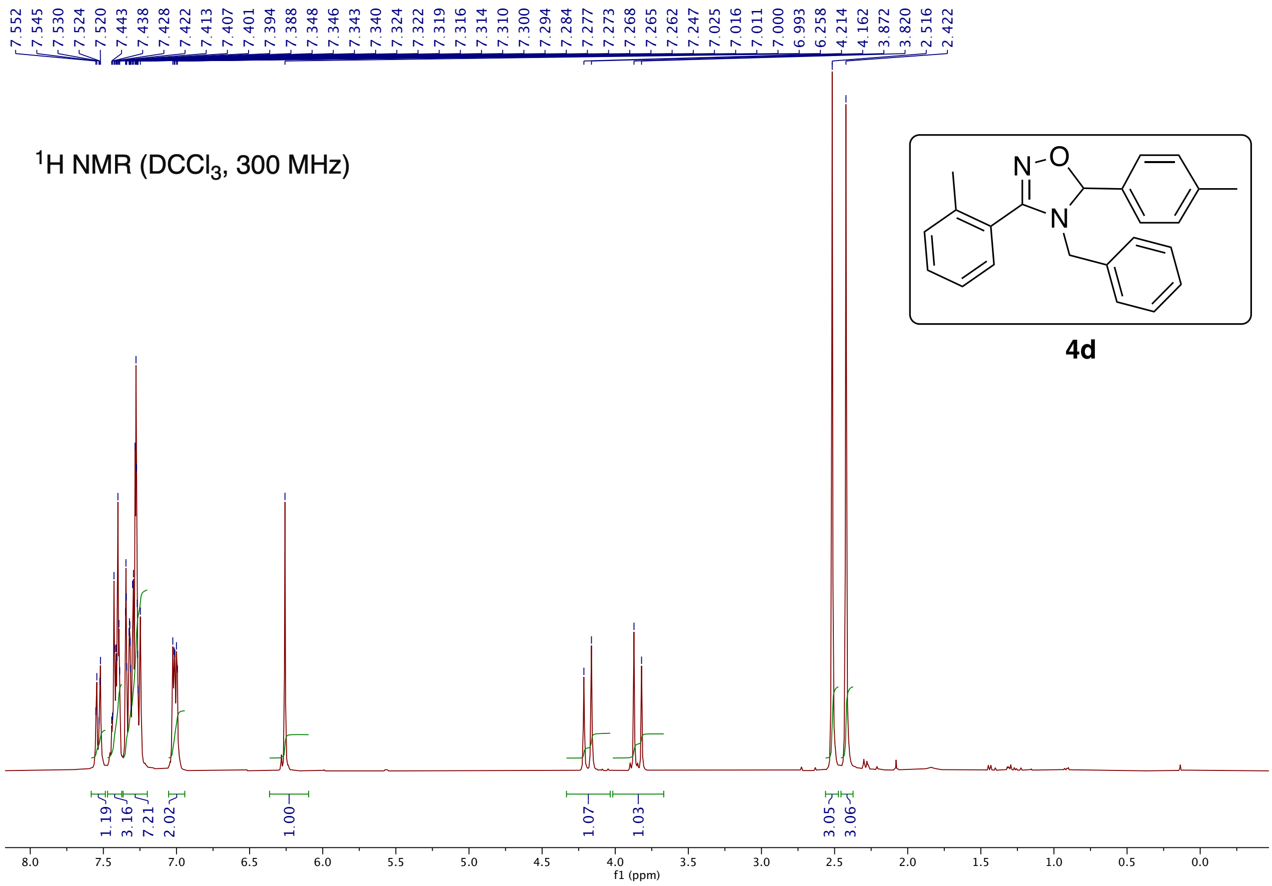
7.552 7.545 7.530 7.524 7.520 7.443 7.438 7.428 7.422 7.413 7.407 7.401 7.394 7.388 7.348 7.346 7.343 7.340 7.324 7.322 7.319 7.316 7.314 7.310 7.300 7.294 7.284 7.277 7.273 7.268 7.265 7.262 7.247 7.025 7.016 7.011 7.000 6.993 6.258 4.214 4.162 3.872 3.820 2.516 2.422
^{1}H NMR (DCCl$_3$, 300 MHz)
4d
1.19 3.16 7.21 2.02 1.00 1.07 1.03 3.05 3.06
f1 (ppm)

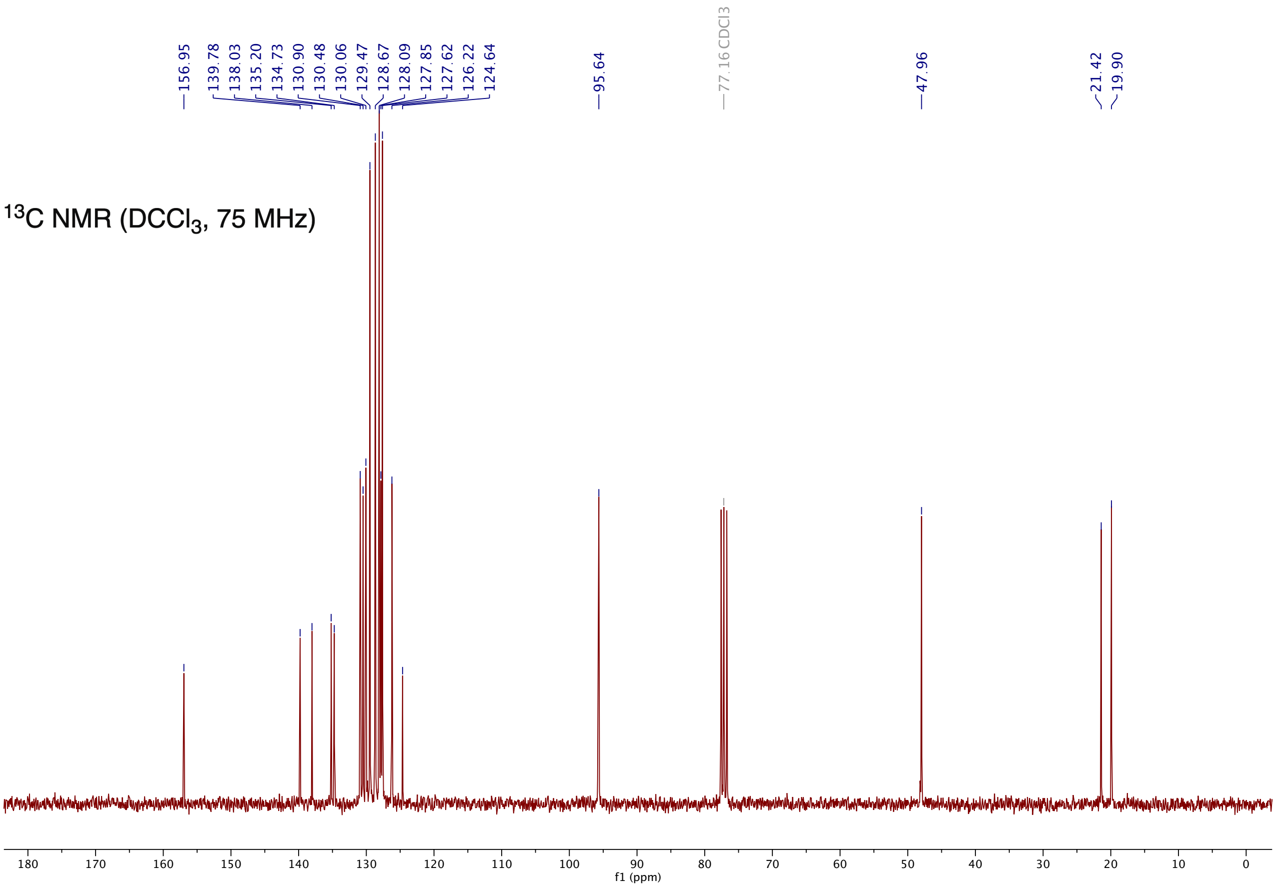
156.95 139.78 138.03 135.20 134.73 130.90 130.48 130.06 129.47 128.67 128.09 127.85 127.62 126.22 124.64 95.64 77.16 CDCl3 47.96 21.42 19.90
^{13}C NMR (DCCl$_3$, 75 MHz)
f1 (ppm)

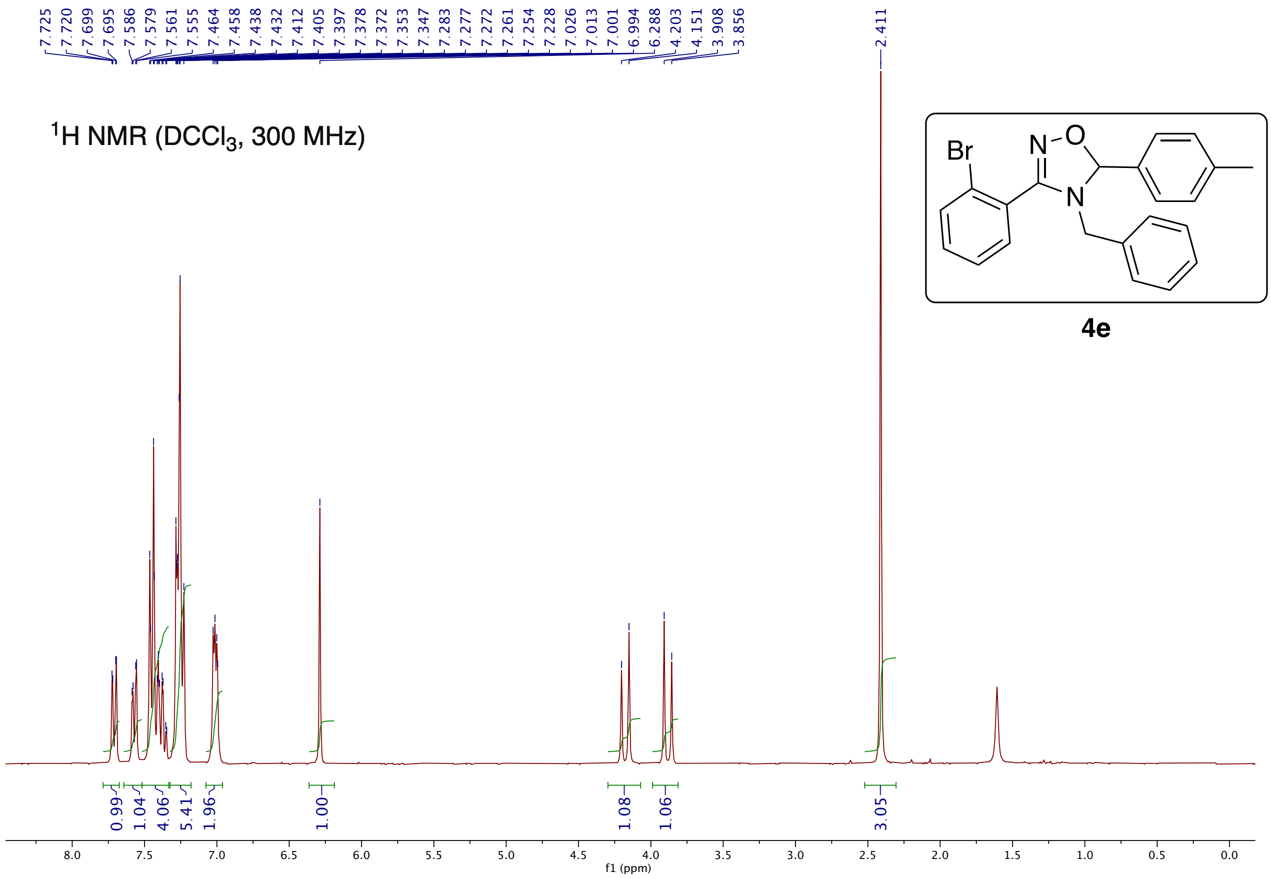

7.725
7.720
7.699
7.695
7.586
7.579
7.561
7.555
7.464
7.458
7.438
7.432
7.412
7.405
7.397
7.378
7.372
7.353
7.347
7.283
7.277
7.272
7.261
7.254
7.228
7.026
7.013
7.001
6.994
6.288
4.203
4.151
3.908
3.856
2.411
1H NMR (DCCl3, 300 MHz)
0.99
1.04
4.06
5.41
1.96
1.00
1.08
1.06
3.05
8.0 7.5 7.0 6.5 6.0 5.5 5.0 4.5 4.0 3.5 3.0 2.5 2.0 1.5 1.0 0.5 0.0
f1 (ppm)
Br
N
O
N

4e

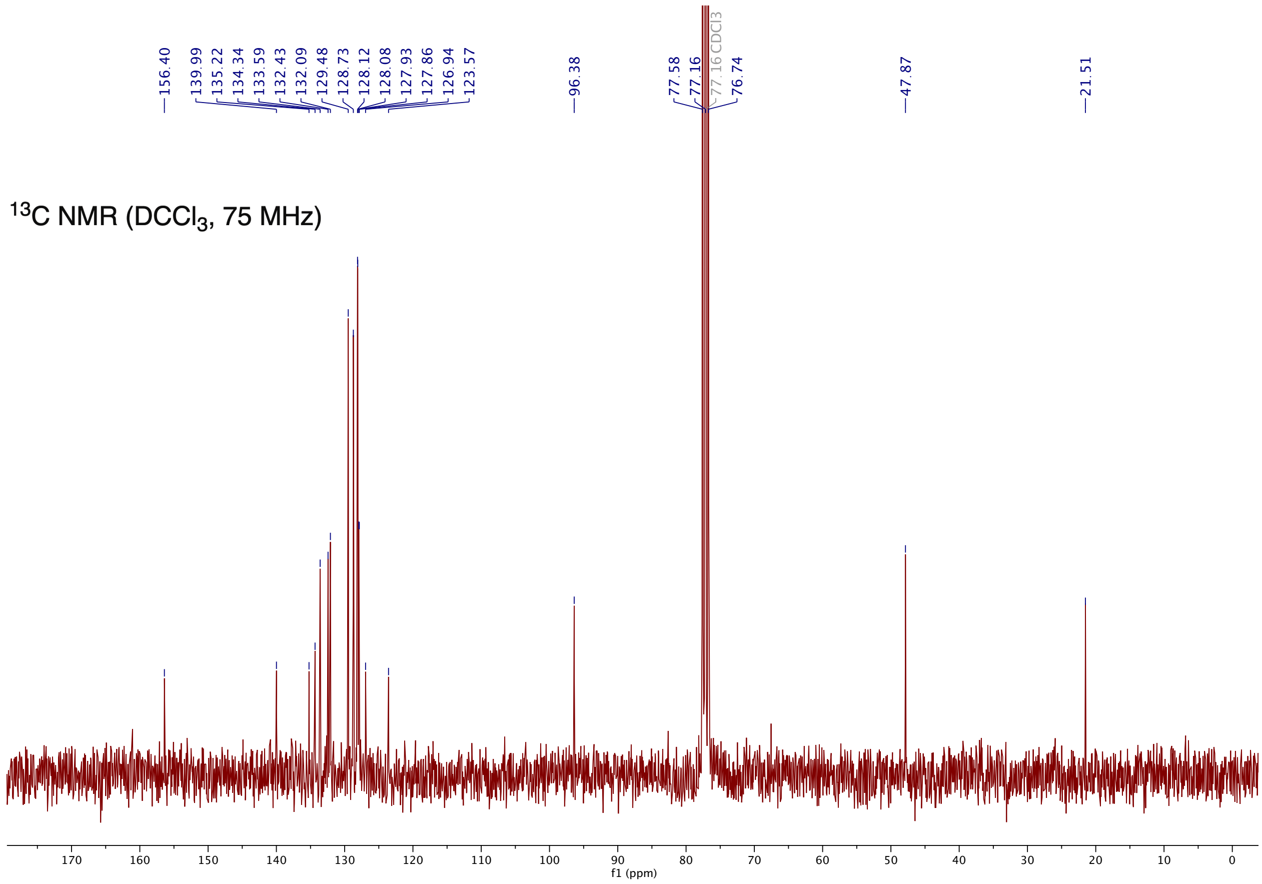

156.40
139.99
135.22
134.34
133.59
132.43
132.09
129.48
128.73
128.12
128.08
127.93
127.86
126.94
123.57
96.38
77.58
77.16
77.16 CDCl3
76.74
47.87
21.51
13C NMR (DCCl3, 75 MHz)
170 160 150 140 130 120 110 100 90 80 70 60 50 40 30 20 10 0
f1 (ppm)

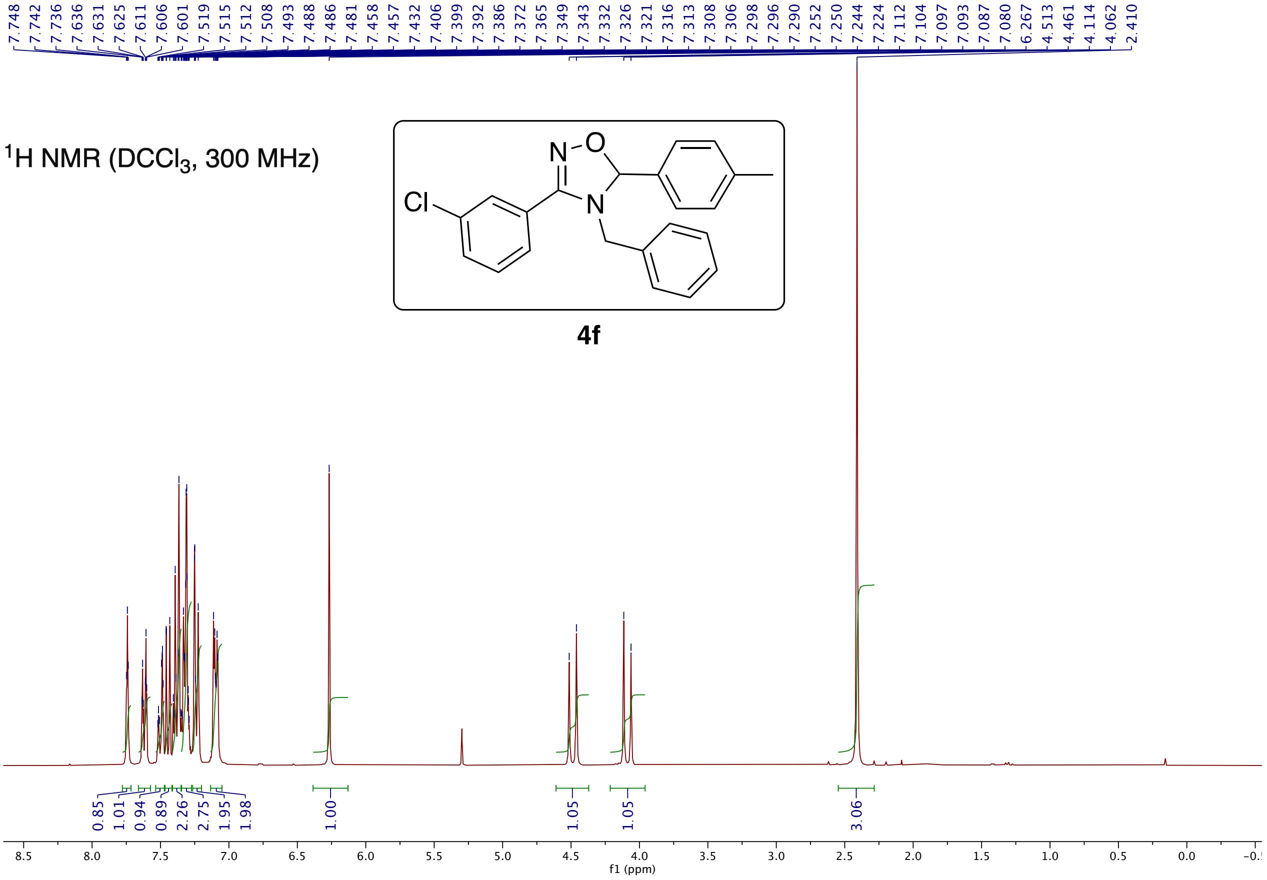
7.748 7.742 7.736 7.636 7.631 7.625 7.611 7.606 7.601 7.519 7.515 7.512 7.508 7.493 7.488 7.486 7.481 7.458 7.457 7.432 7.406 7.399 7.392 7.386 7.372 7.365 7.349 7.343 7.332 7.326 7.321 7.316 7.313 7.308 7.306 7.298 7.296 7.290 7.252 7.250 7.244 7.224 7.112 7.104 7.097 7.093 7.087 7.080 6.267 4.513 4.461 4.114 4.062 2.410
¹H NMR (DCCl3, 300 MHz)
Cl
N
O
N
4f
0.85 1.01 0.94 0.89 2.26 2.75 1.95 1.98 1.00 1.05 1.05 3.06
f1 (ppm)

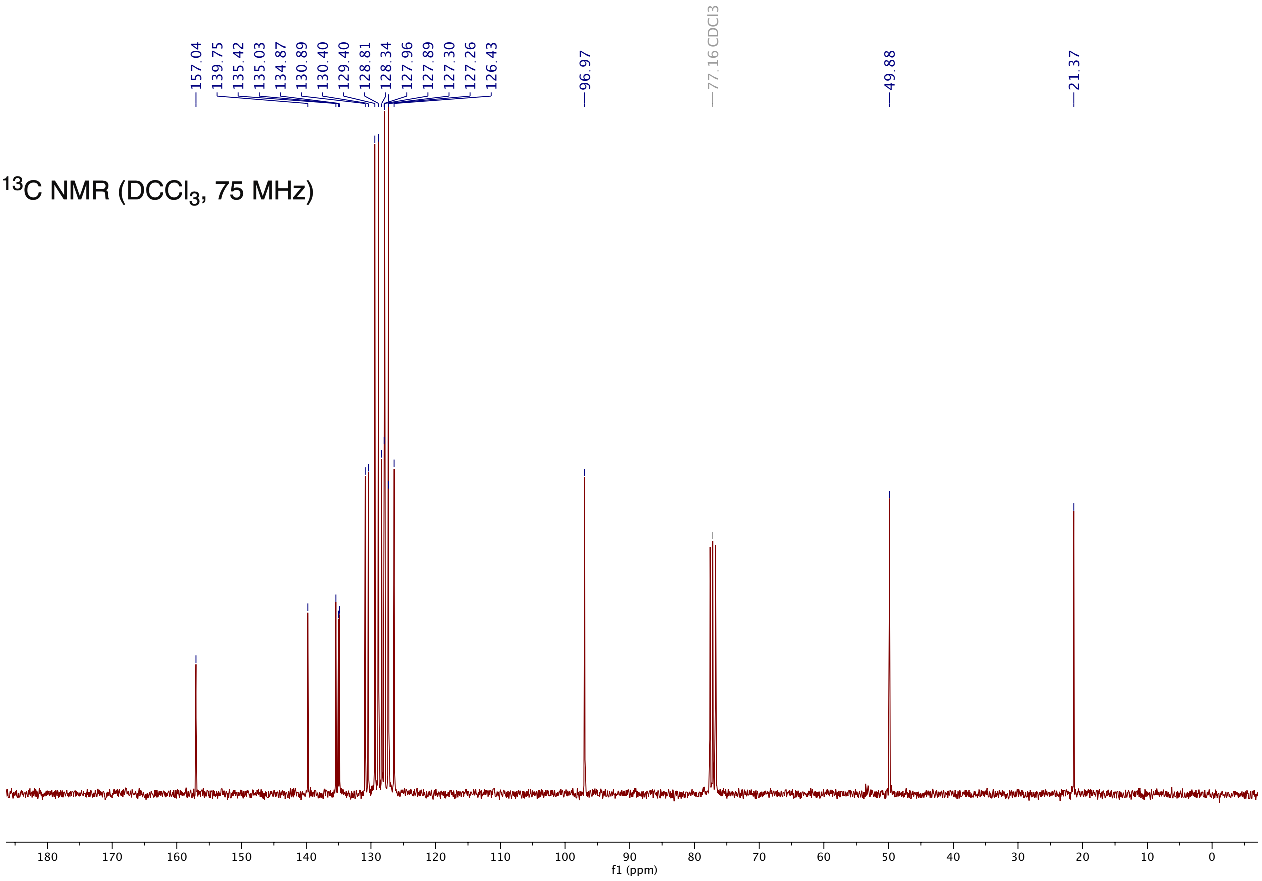
157.04 139.75 135.42 135.03 134.87 130.89 130.40 129.40 128.81 128.34 127.96 127.89 127.30 127.26 126.43 96.97 77.16 CDCl3 49.88 21.37
¹³C NMR (DCCl3, 75 MHz)
f1 (ppm)

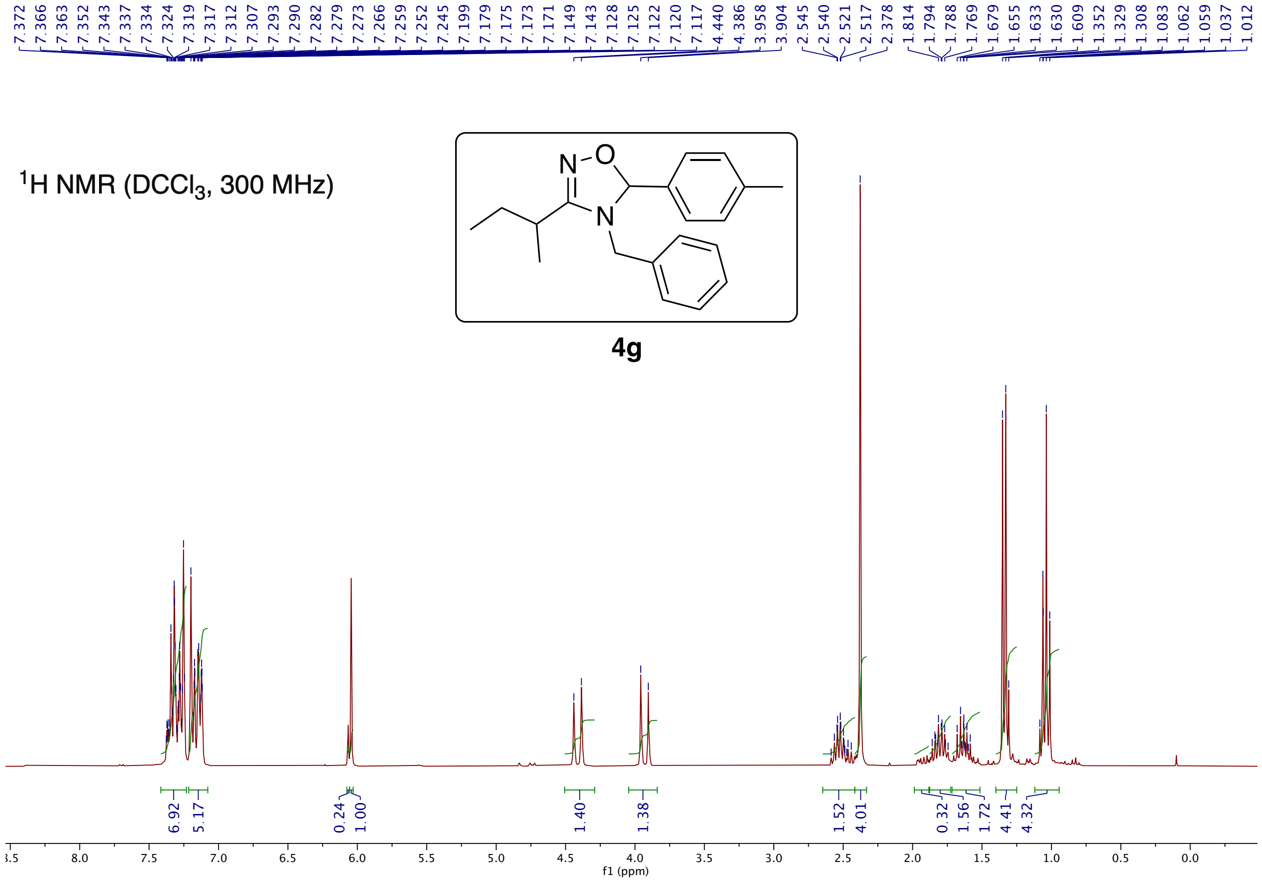
7.372 7.366 7.363 7.352 7.343 7.337 7.334 7.324 7.319 7.317 7.312 7.307 7.293 7.290 7.282 7.279 7.273 7.266 7.259 7.252 7.245 7.199 7.179 7.175 7.173 7.171 7.149 7.143 7.128 7.125 7.122 7.120 7.117 4.440 4.386 3.958 3.904 2.545 2.540 2.521 2.517 2.378 1.814 1.794 1.788 1.769 1.679 1.655 1.633 1.630 1.609 1.352 1.329 1.308 1.083 1.062 1.059 1.037 1.012
^{1}H NMR (DCCl$_3$, 300 MHz)
N
O
N
4g
6.92 5.17 0.24 1.00 1.40 1.38 1.52 4.01 0.32 1.56 1.72 4.41 4.32
f1 (ppm)

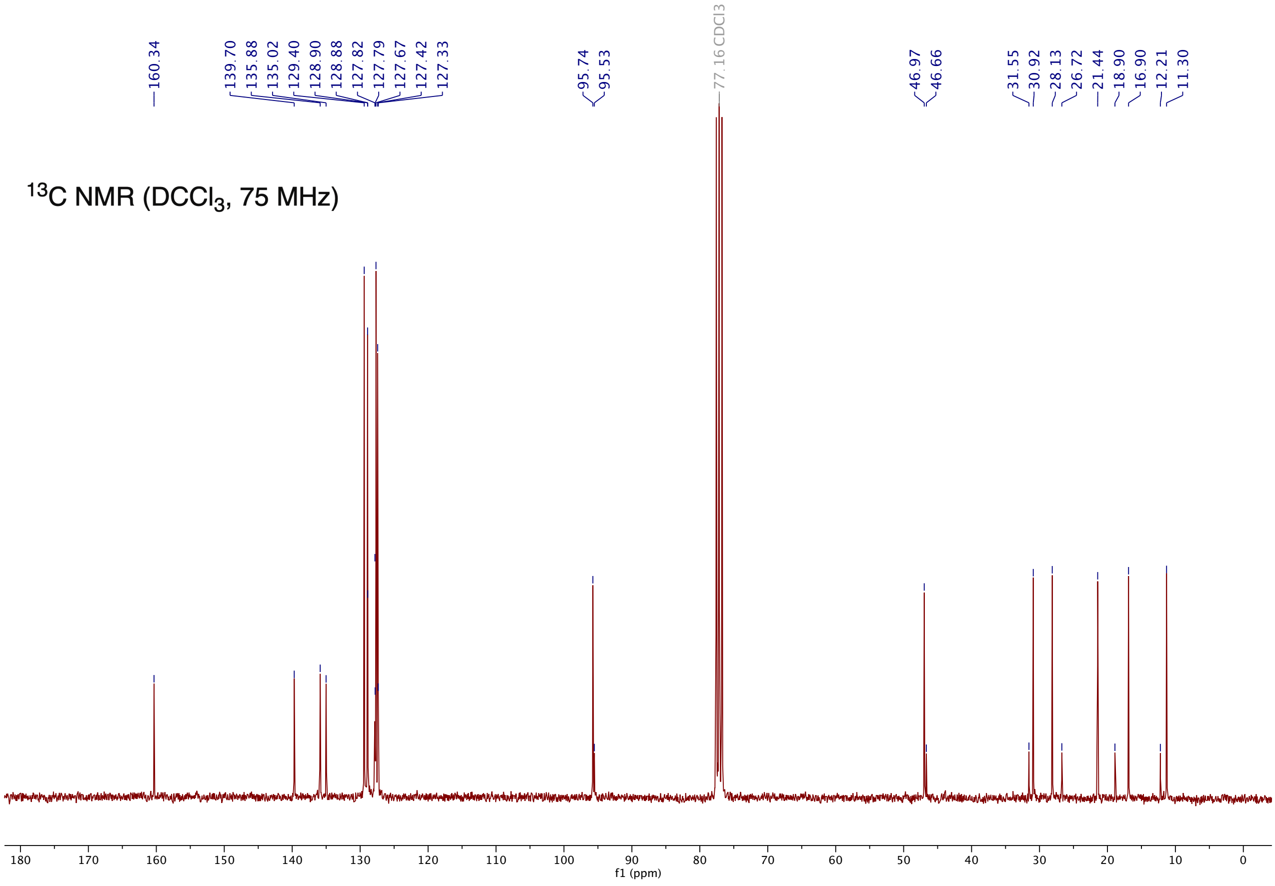
160.34 139.70 135.88 135.02 129.40 128.90 128.88 127.82 127.79 127.67 127.42 127.33 95.74 95.53 77.16 CDCl3 46.97 46.66 31.55 30.92 28.13 26.72 21.44 18.90 16.90 12.21 11.30
^{13}C NMR (DCCl$_3$, 75 MHz)
f1 (ppm)

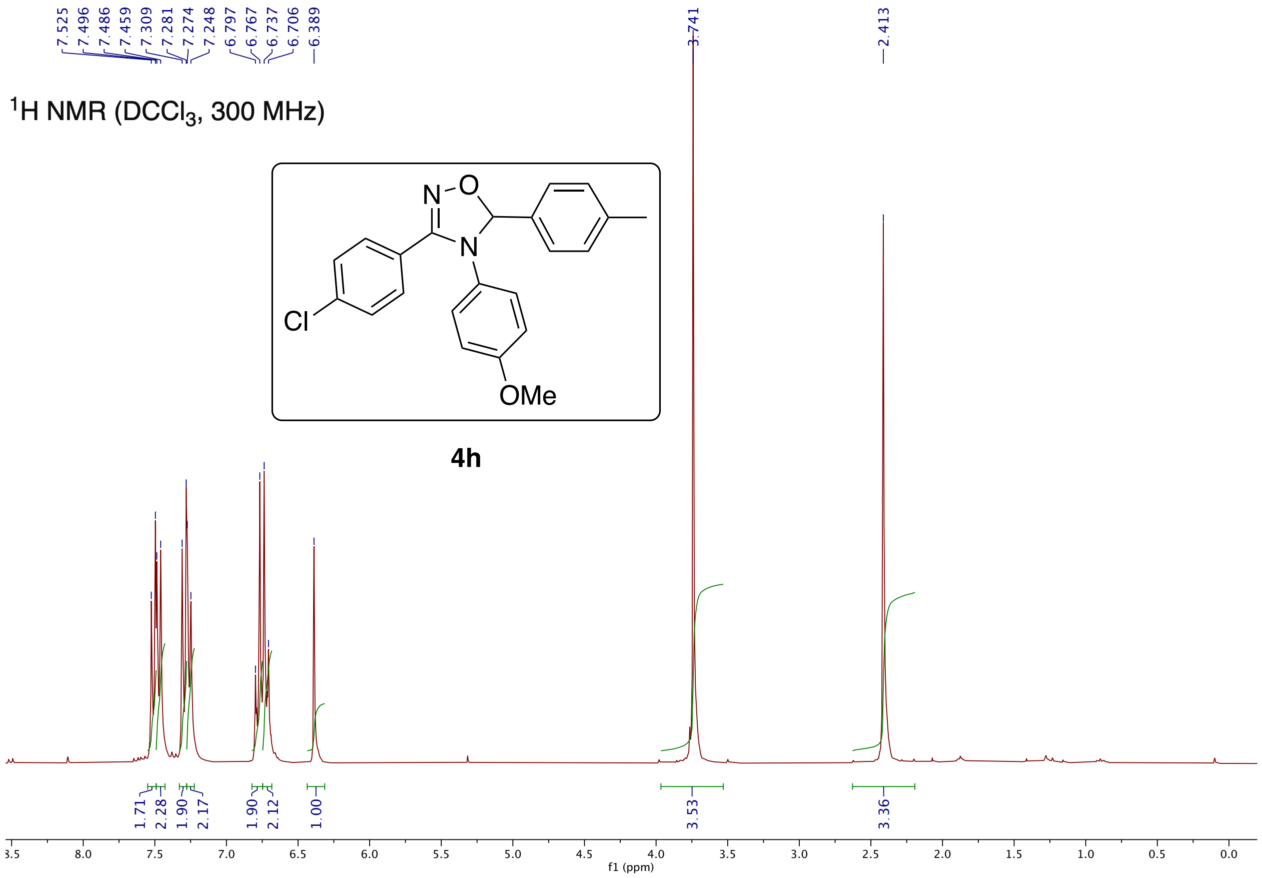
1H NMR (DCCl3, 300 MHz)
4h
N
O
N
Cl
OMe
7.525
7.496
7.486
7.459
7.309
7.281
7.274
7.248
6.797
6.767
6.737
6.706
6.389
3.741
2.413
1.71
2.28
1.90
2.17
1.90
2.12
1.00
3.53
3.36
f1 (ppm)

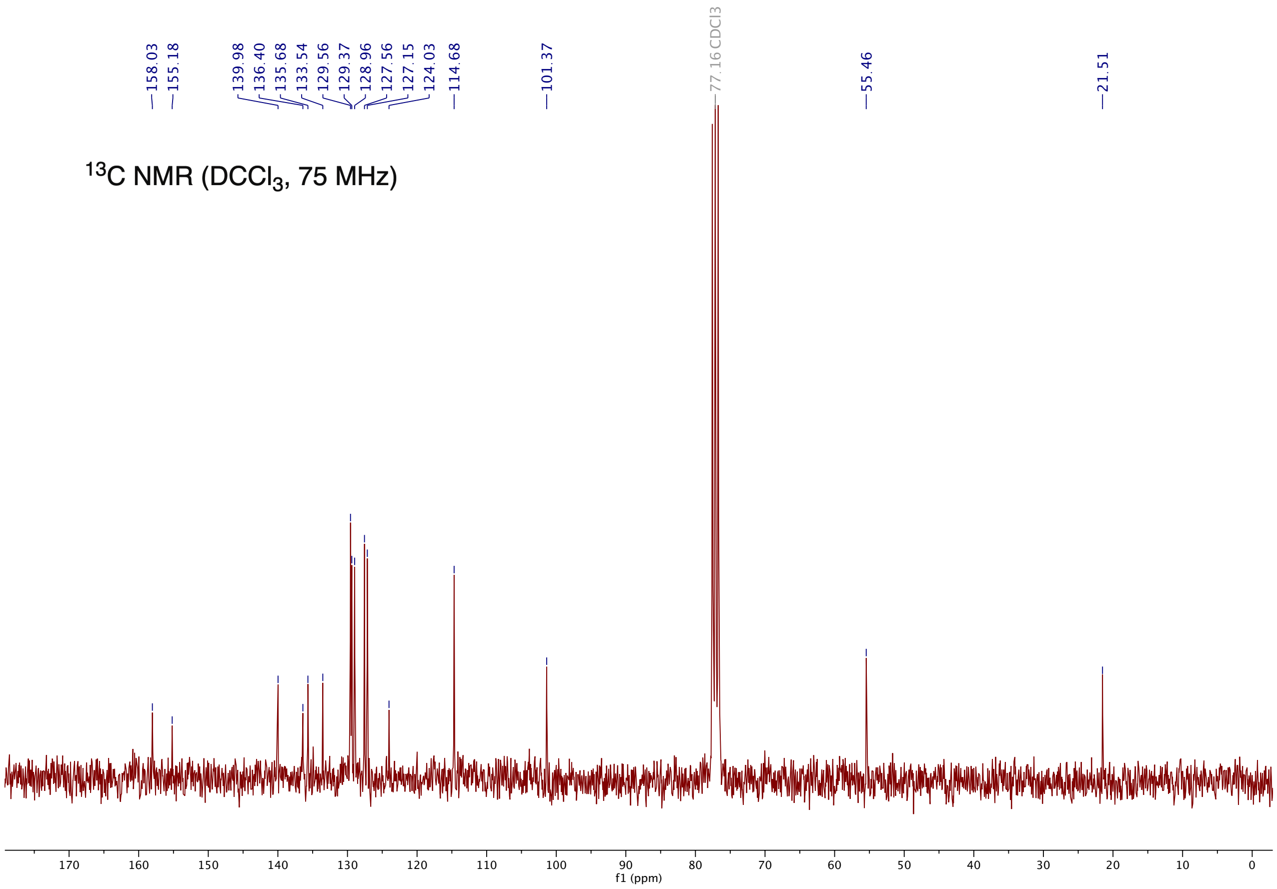
13C NMR (DCCl3, 75 MHz)
158.03
155.18
139.98
136.40
135.68
133.54
129.56
129.37
128.96
127.56
127.15
124.03
114.68
101.37
77.16 CDCl3
55.46
21.51
f1 (ppm)

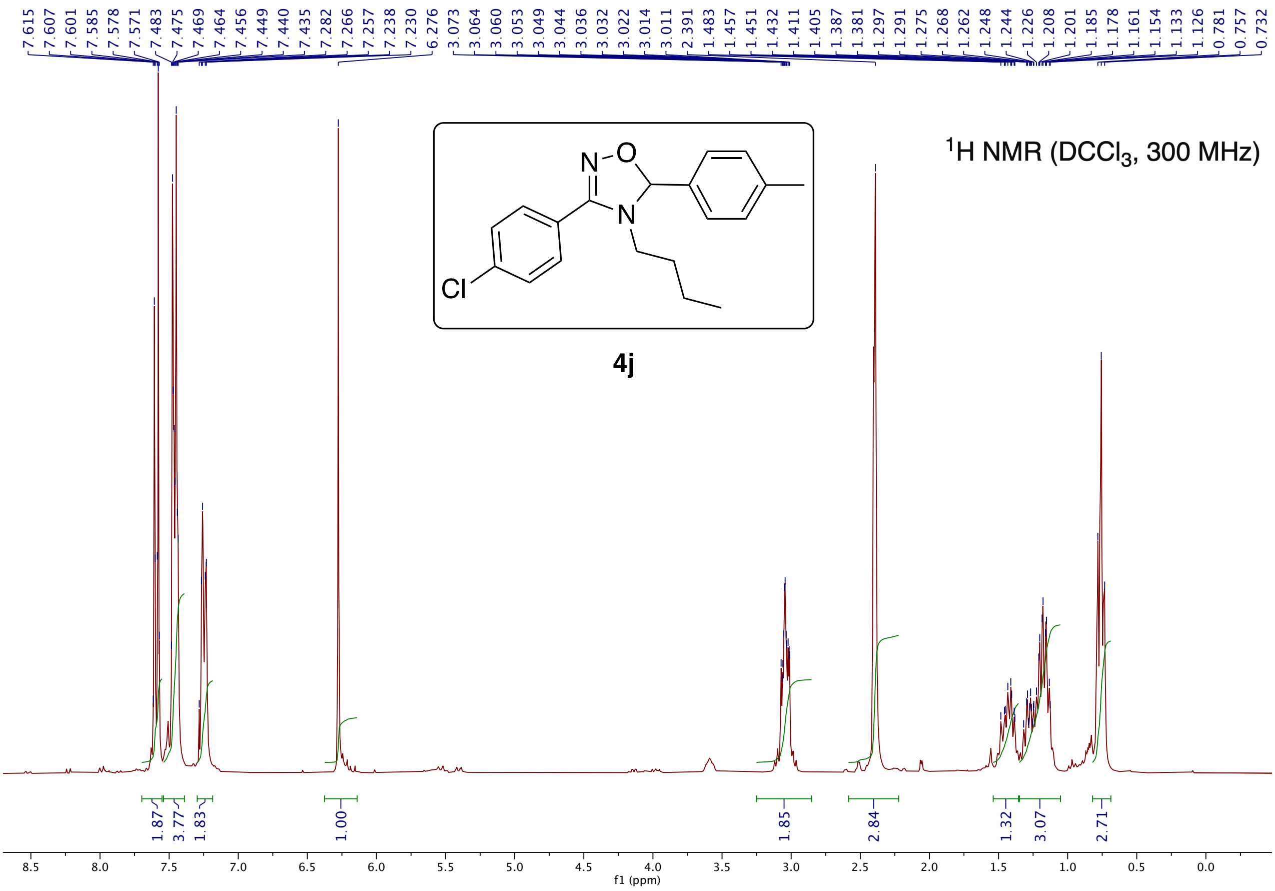
7.615 7.607 7.601 7.585 7.578 7.571 7.483 7.475 7.469 7.464 7.456 7.449 7.440 7.435 7.282 7.266 7.257 7.238 7.230 6.276 3.073 3.064 3.060 3.053 3.049 3.044 3.036 3.032 3.022 3.014 3.011 2.391 1.483 1.457 1.451 1.432 1.411 1.405 1.387 1.381 1.297 1.291 1.275 1.268 1.262 1.248 1.244 1.226 1.208 1.201 1.185 1.178 1.161 1.154 1.133 1.126 0.781 0.757 0.732
^{1}H NMR (DCCl$_3$, 300 MHz)
N
O
N
Cl
4j
1.87 3.77 1.83 1.00 1.85 2.84 1.32 3.07 2.71
f1 (ppm)

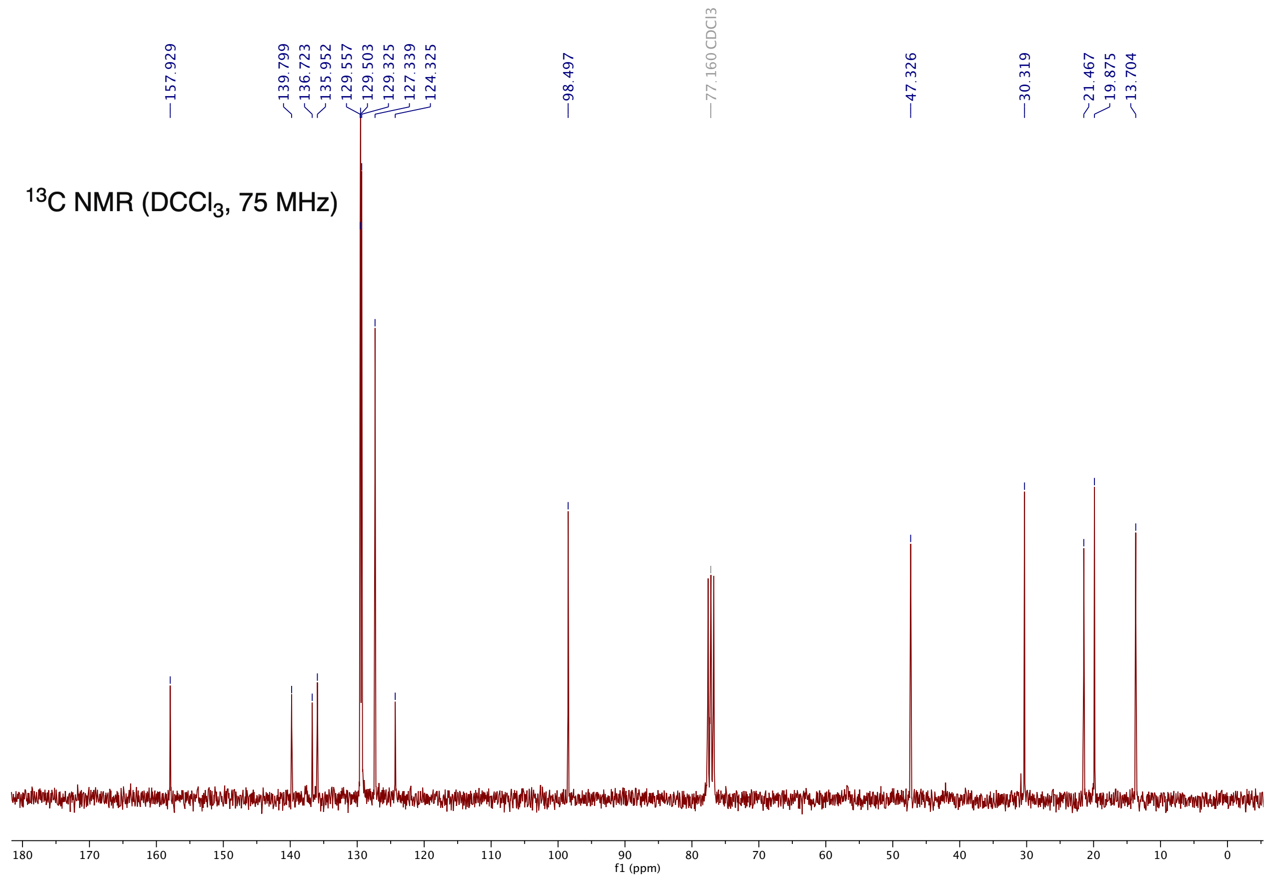
157.929 139.799 136.723 135.952 129.557 129.503 129.325 127.339 124.325 98.497 77.160 CDCl3 47.326 30.319 21.467 19.875 13.704
^{13}C NMR (DCCl$_3$, 75 MHz)
f1 (ppm)

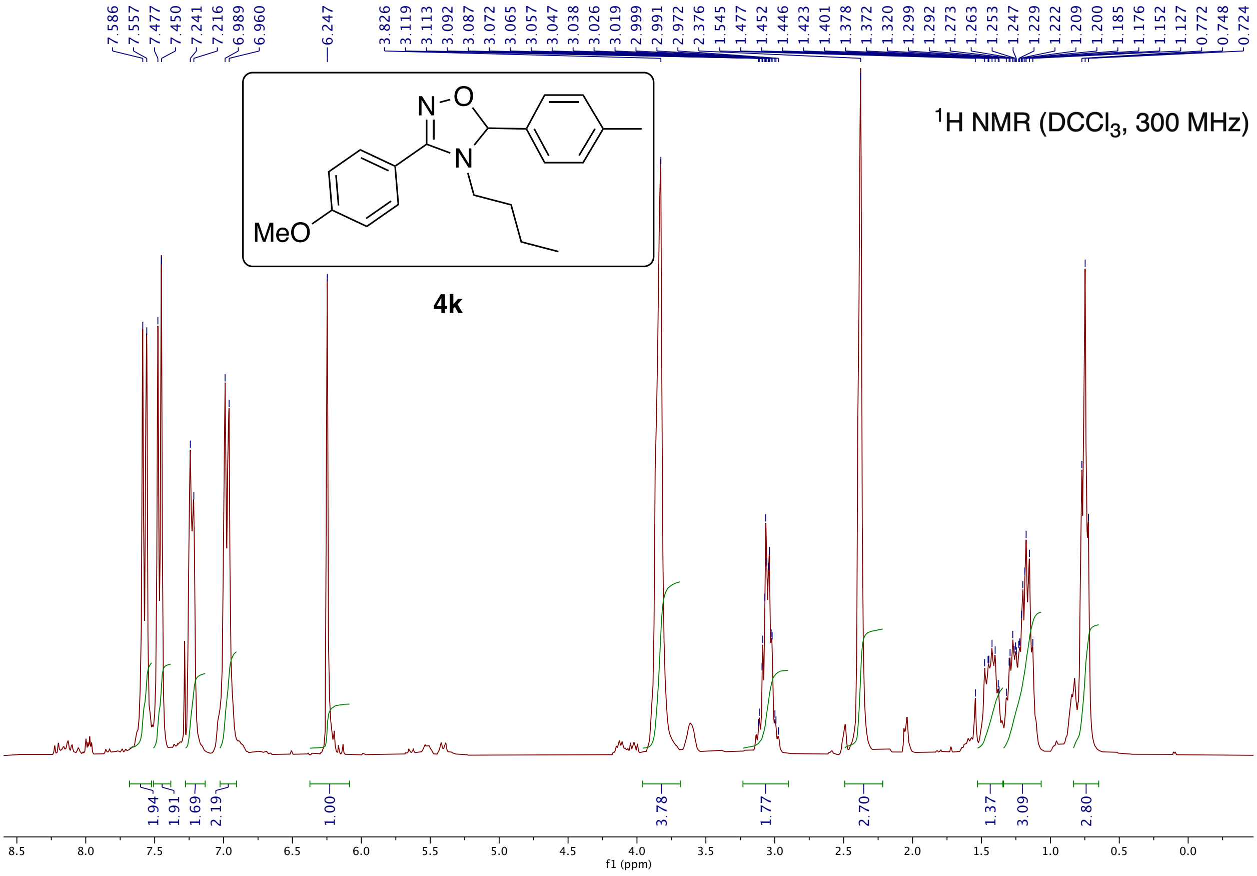
7.586
7.557
7.477
7.450
7.241
7.216
6.989
6.960
6.247
3.826
3.119
3.113
3.092
3.087
3.072
3.065
3.057
3.047
3.038
3.026
3.019
2.999
2.991
2.972
2.376
1.545
1.477
1.452
1.446
1.423
1.401
1.378
1.372
1.320
1.299
1.292
1.273
1.263
1.253
1.247
1.229
1.222
1.209
1.200
1.185
1.176
1.152
1.127
0.772
0.748
0.724
^{1}H NMR ($DCCl_3$, 300 MHz)
N
O
N
MeO
4k
1.94
1.91
1.69
2.19
1.00
3.78
1.77
2.70
1.37
3.09
2.80
f1 (ppm)

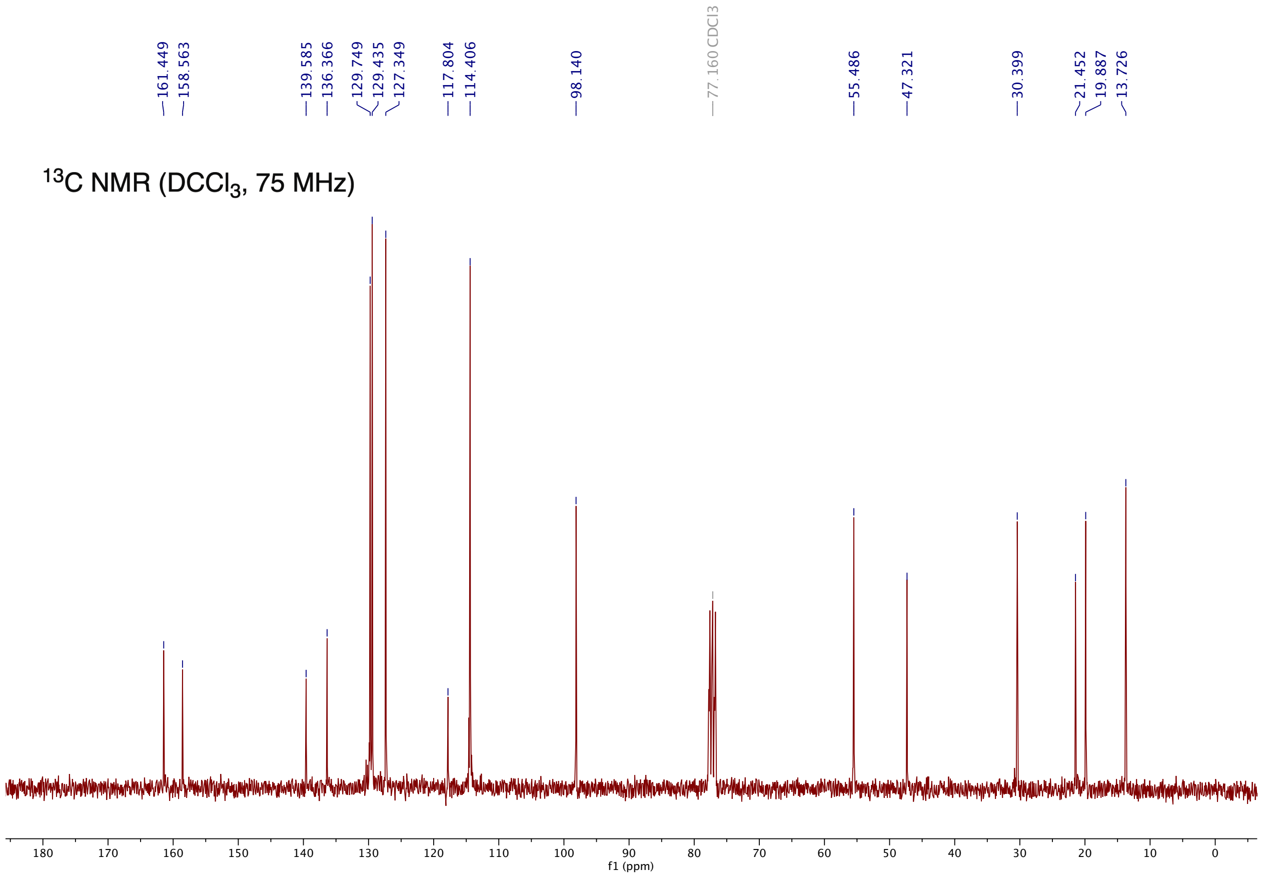
161.449
158.563
139.585
136.366
129.749
129.435
127.349
117.804
114.406
98.140
77.160 CDCl3
55.486
47.321
30.399
21.452
19.887
13.726
^{13}C NMR ($DCCl_3$, 75 MHz)
f1 (ppm)

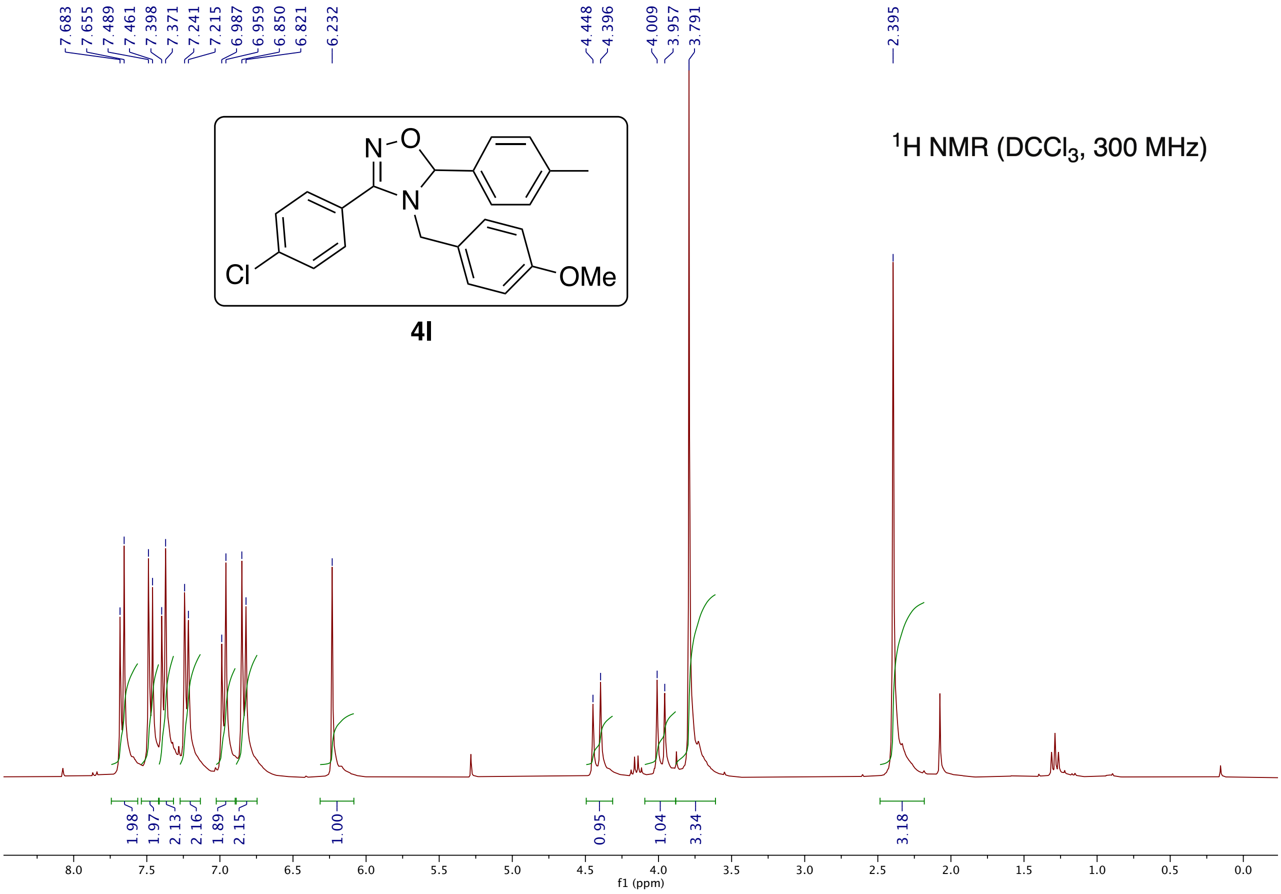
7.683
7.655
7.489
7.461
7.398
7.371
7.241
7.215
6.987
6.959
6.850
6.821
6.232
4.448
4.396
4.009
3.957
3.791
2.395
N
O
N
Cl
OMe
4l
^{1}H NMR (DCCl$_3$, 300 MHz)
1.98
1.97
2.13
2.16
1.89
2.15
1.00
0.95
1.04
3.34
3.18
f1 (ppm)

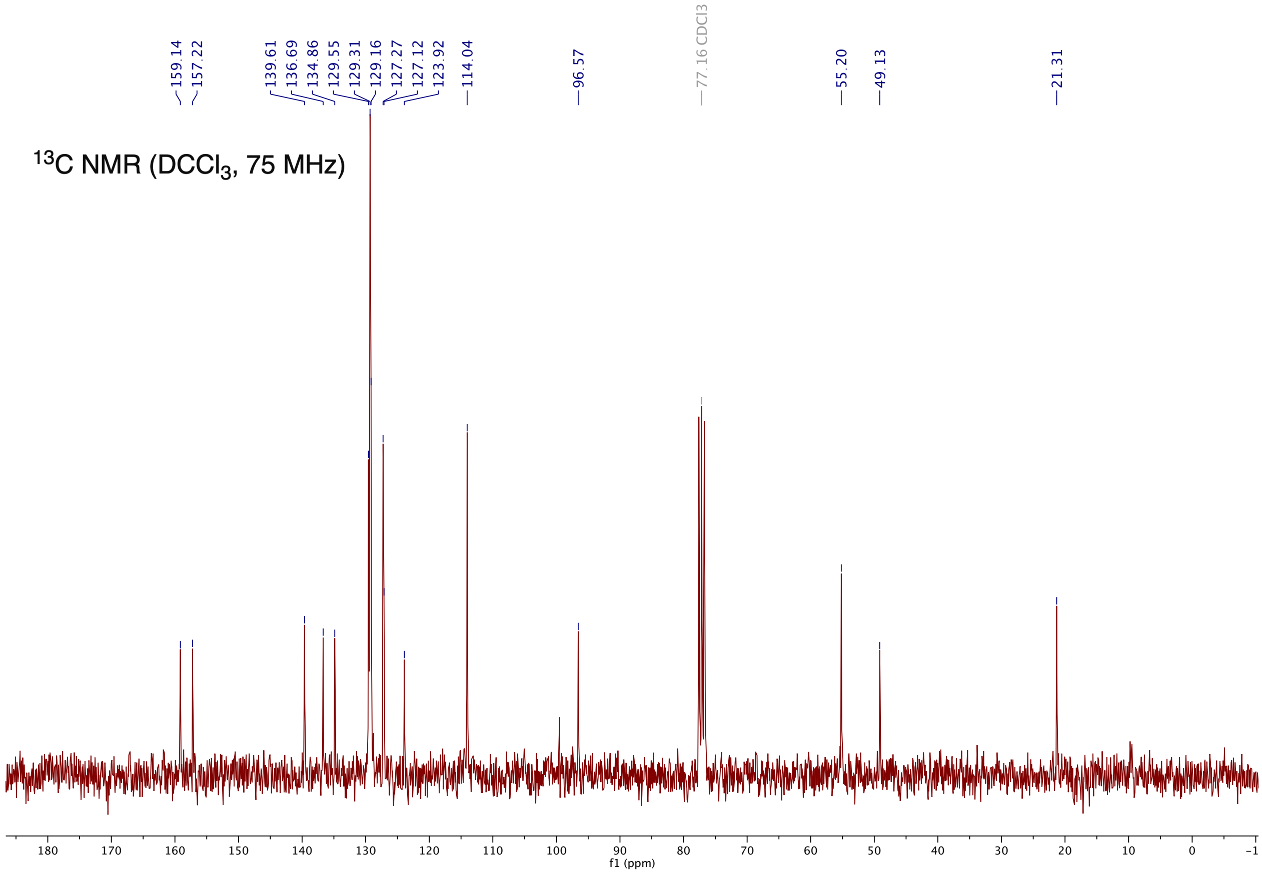
159.14
157.22
139.61
136.69
134.86
129.55
129.31
129.16
127.27
127.12
123.92
114.04
96.57
77.16 CDCl3
55.20
49.13
21.31
^{13}C NMR (DCCl$_3$, 75 MHz)
f1 (ppm)

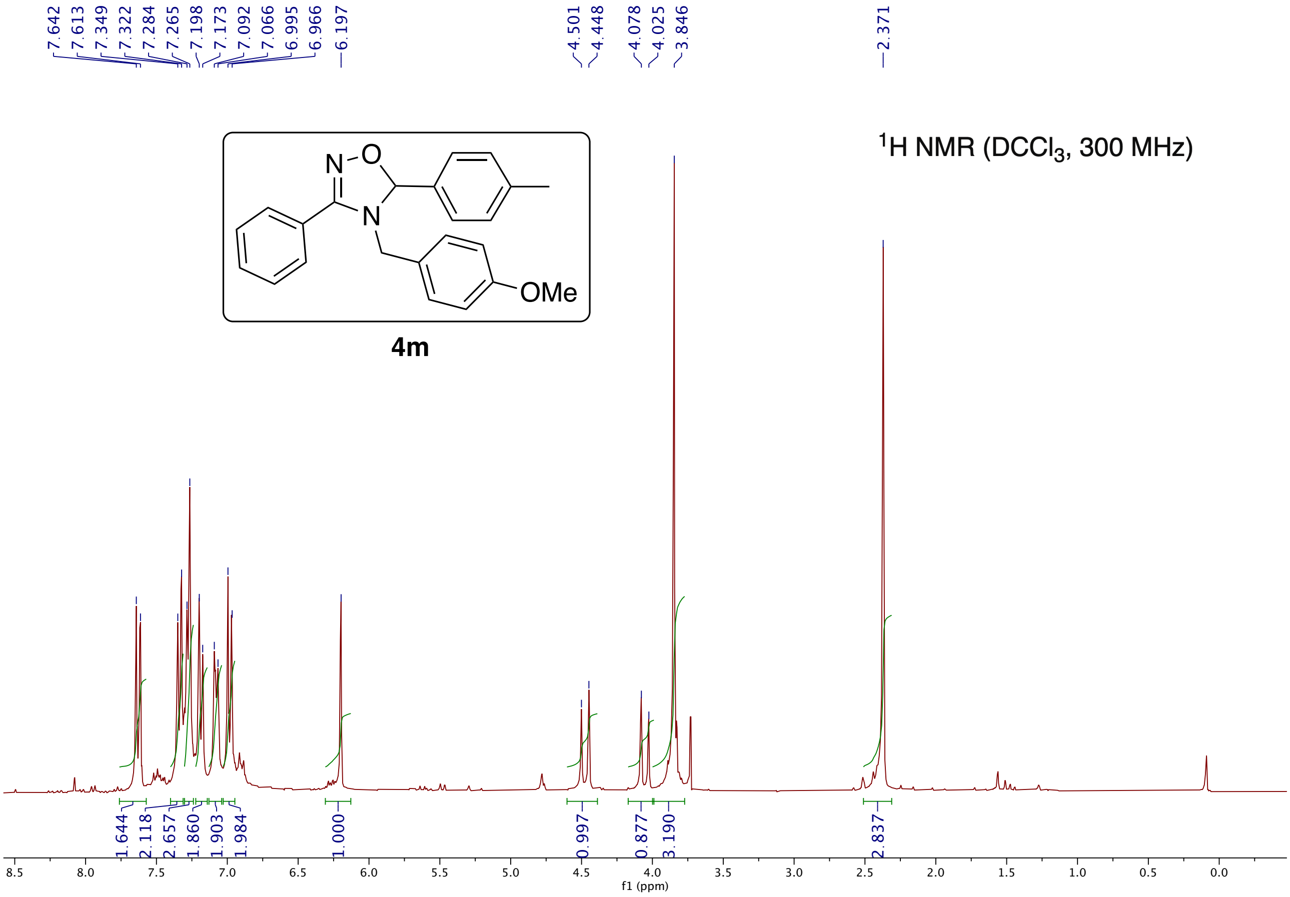
7.642
7.613
7.349
7.322
7.284
7.265
7.198
7.173
7.092
7.066
6.995
6.966
6.197
4.501
4.448
4.078
4.025
3.846
2.371
^{1}H NMR ($DCCl_3$, 300 MHz)
1.644
2.118
2.657
1.860
1.903
1.984
1.000
0.997
0.877
3.190
2.837
N
O
N
OMe
f1 (ppm)

4m

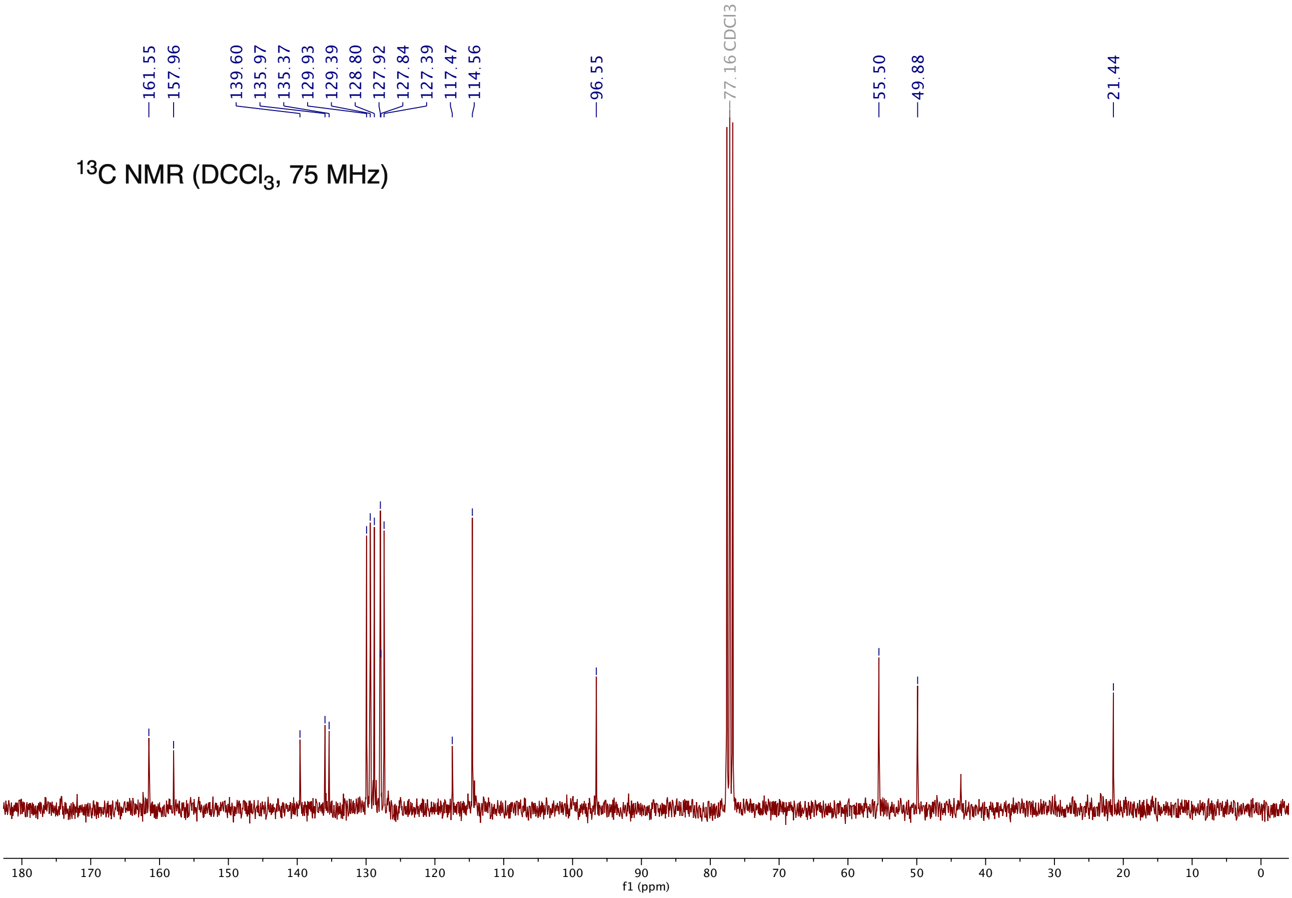
161.55
157.96
139.60
135.97
135.37
129.93
129.39
128.80
127.92
127.84
127.39
117.47
114.56
96.55
77.16 CDCl3
55.50
49.88
21.44
^{13}C NMR ($DCCl_3$, 75 MHz)
f1 (ppm)

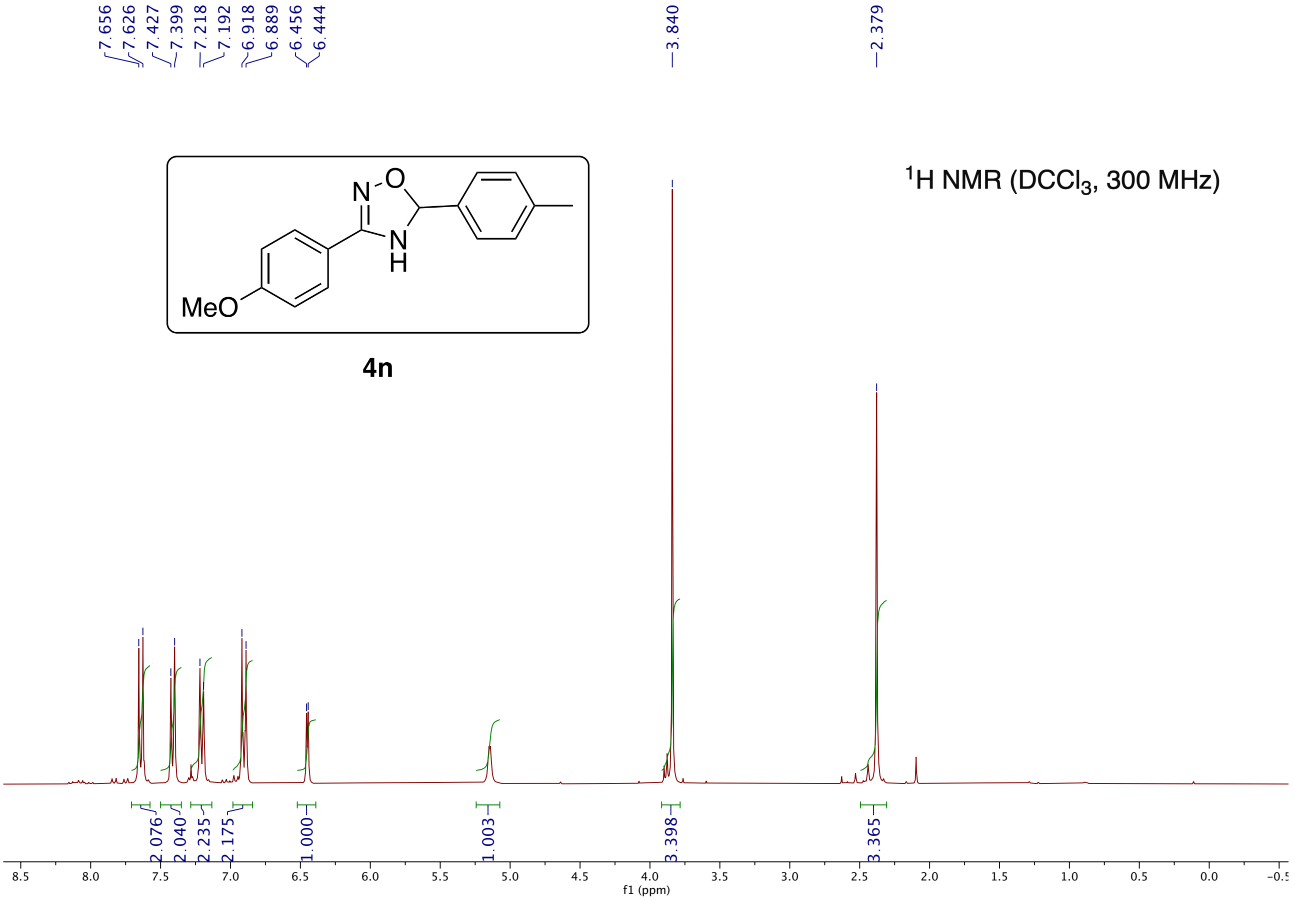
7.656
7.626
7.427
7.399
7.218
7.192
6.918
6.889
6.456
6.444
3.840
2.379
^{1}H NMR (DCCl$_3$, 300 MHz)
N
O
N
H
MeO
4n
2.076
2.040
2.235
2.175
1.000
1.003
3.398
3.365
f1 (ppm)

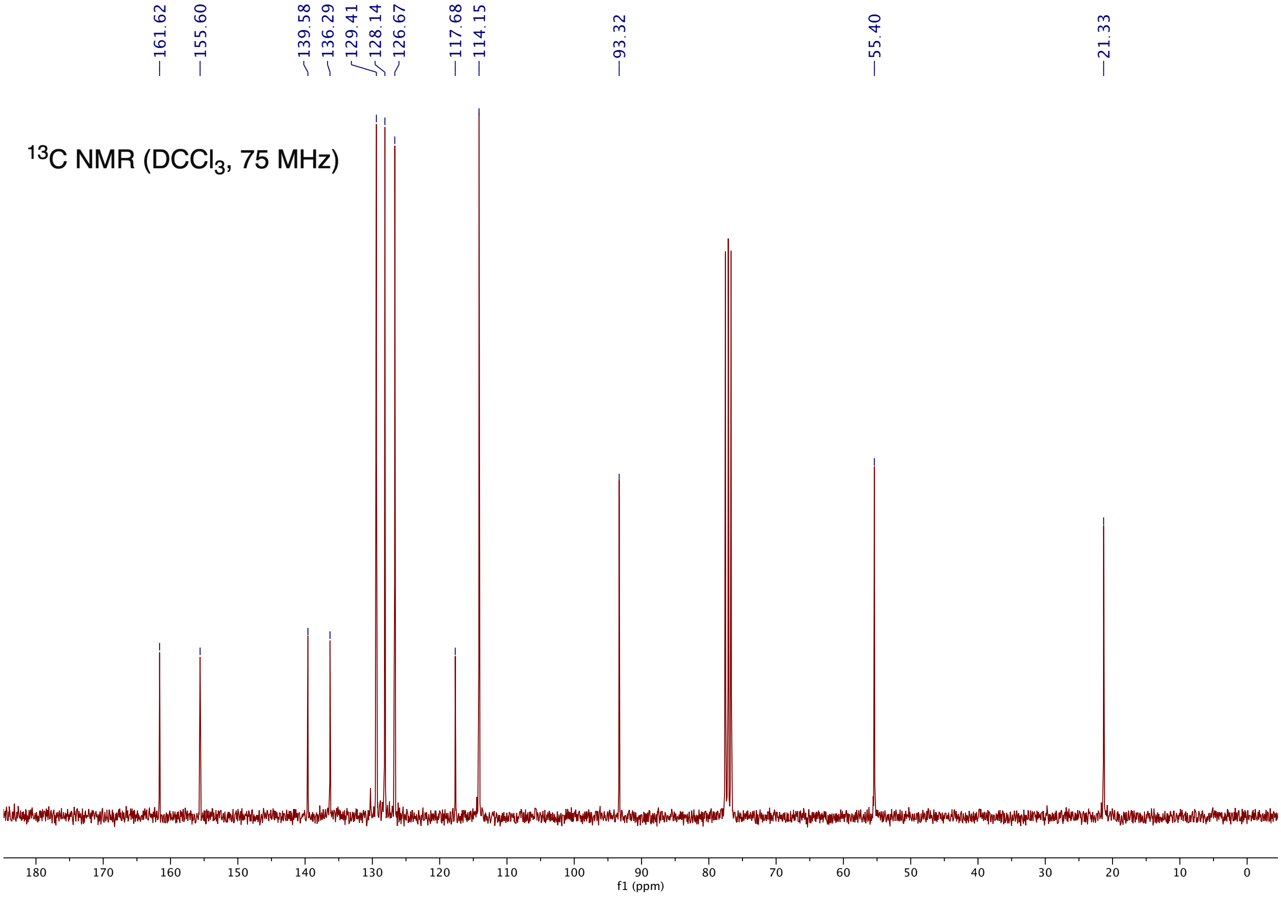
161.62
155.60
139.58
136.29
129.41
128.14
126.67
117.68
114.15
93.32
55.40
21.33
^{13}C NMR (DCCl$_3$, 75 MHz)
f1 (ppm)

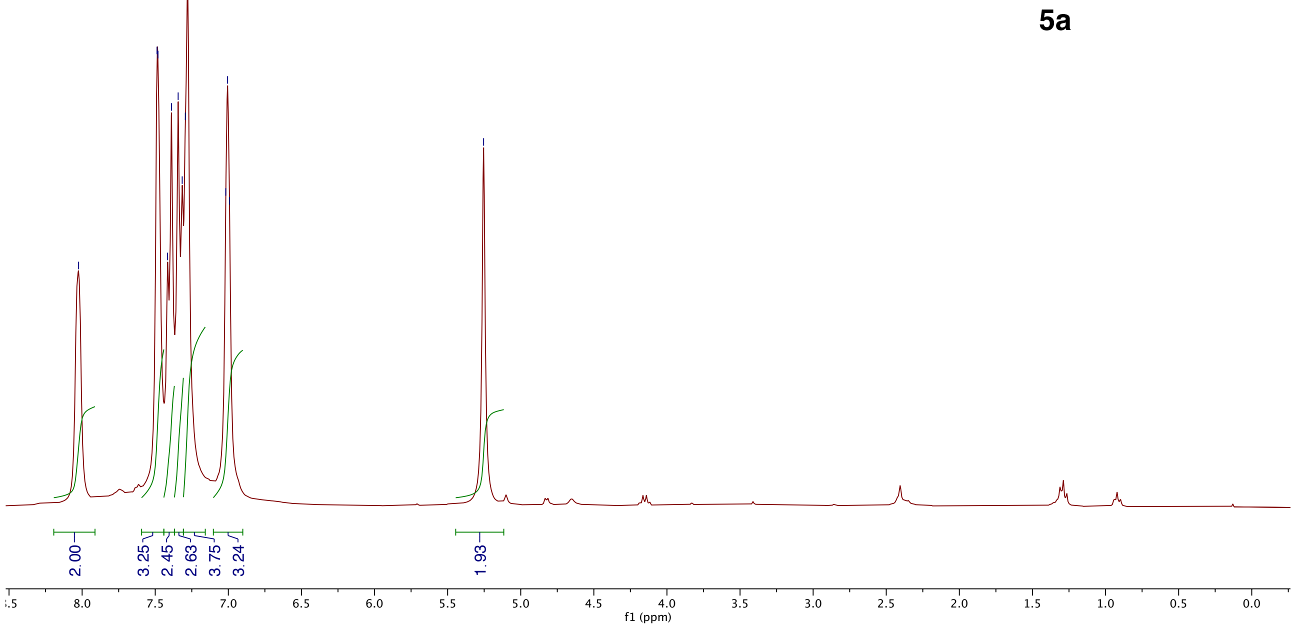
8.024
7.488
7.481
7.416
7.389
7.343
7.316
7.294
7.278
7.018
7.005
6.992
5.254
¹H NMR (DCCl₃, 300 MHz)
2.00
3.25
2.45
2.63
3.75
3.24
1.93
f1 (ppm)

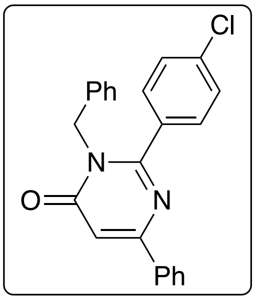
Cl
Ph
N
O
N
Ph

5a

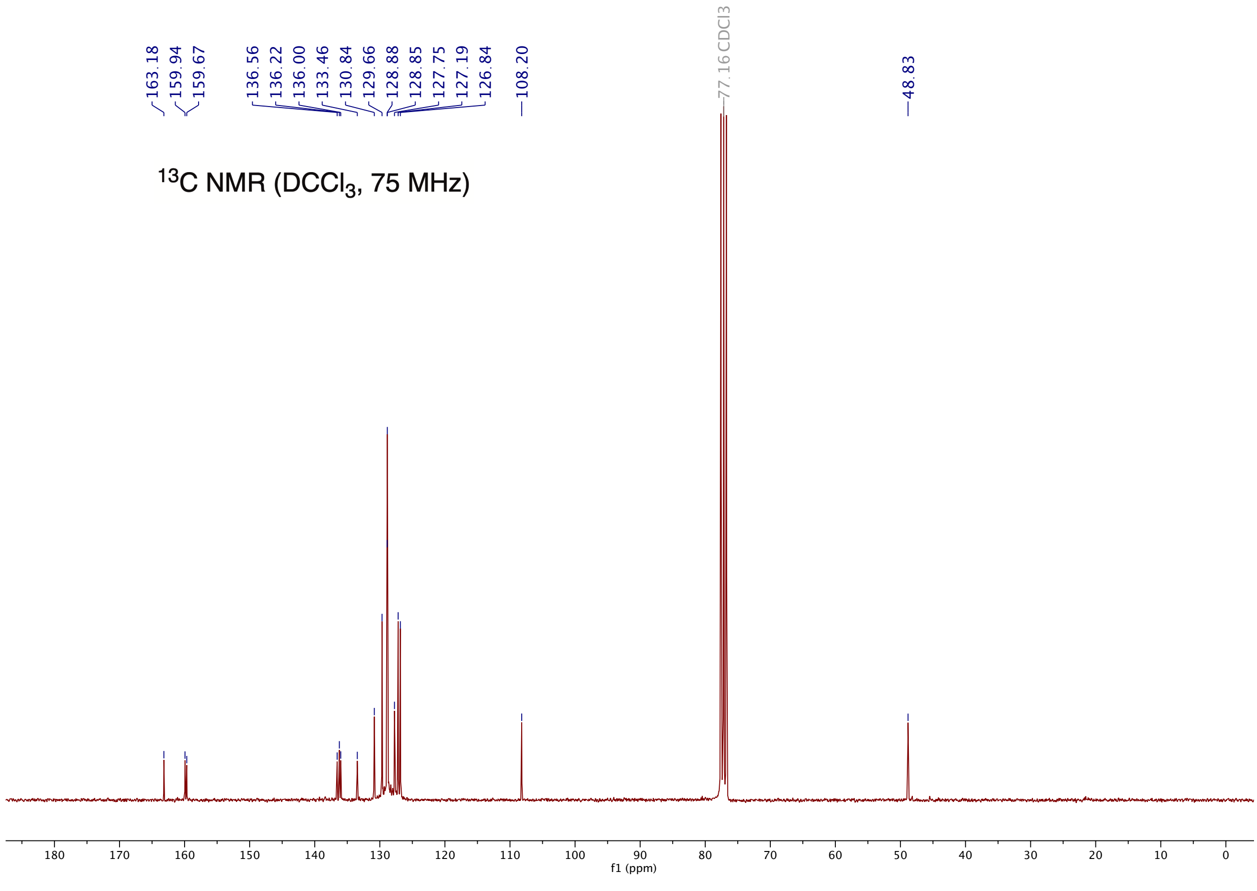
163.18
159.94
159.67
136.56
136.22
136.00
133.46
130.84
129.66
128.88
128.85
127.75
127.19
126.84
108.20
77.16 CDCl3
48.83
¹³C NMR (DCCl₃, 75 MHz)
f1 (ppm)

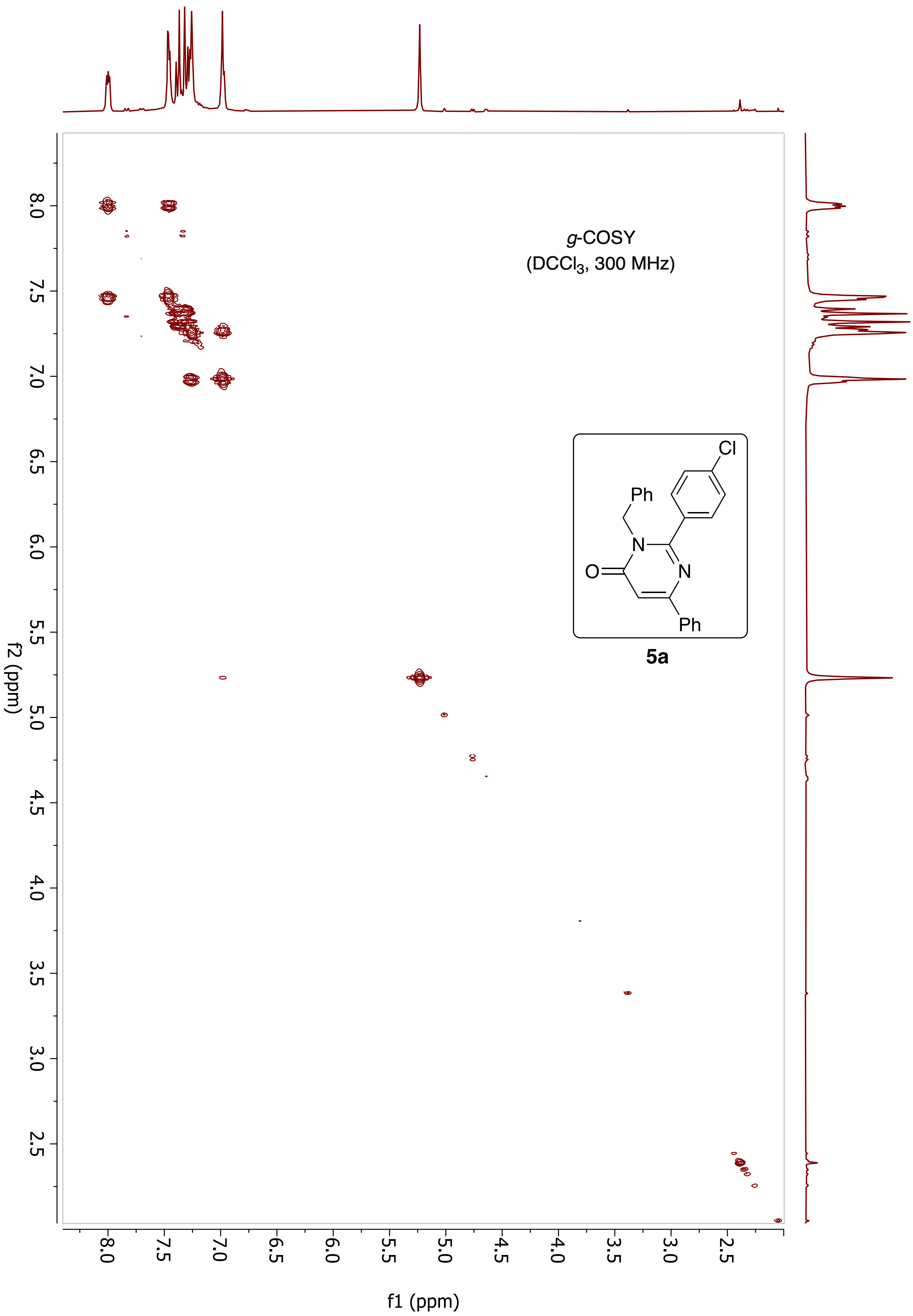
g-COSY
(DCCl3, 300 MHz)
Cl
Ph
N
O
N
Ph
5a
f2 (ppm)
f1 (ppm)

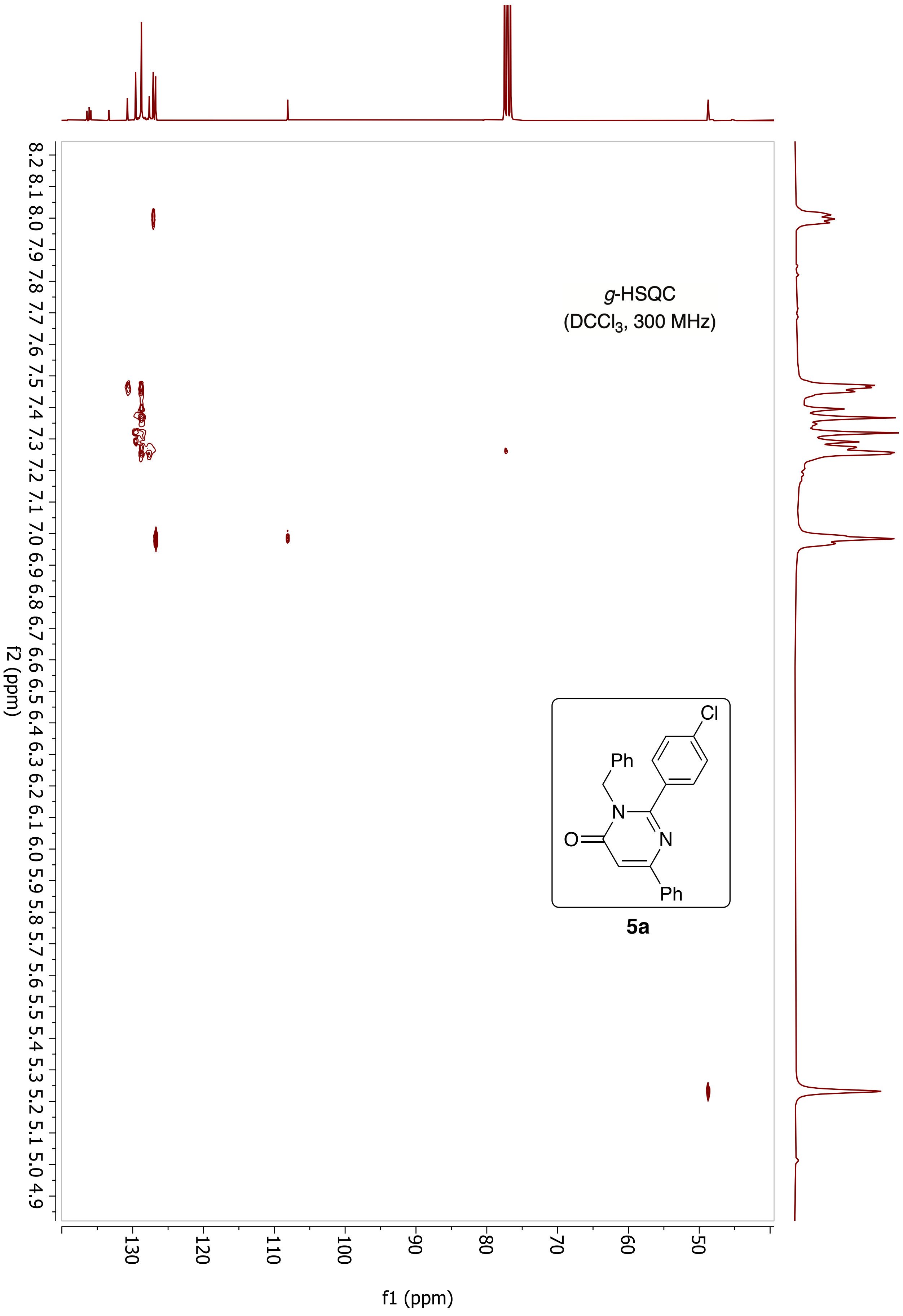

5a

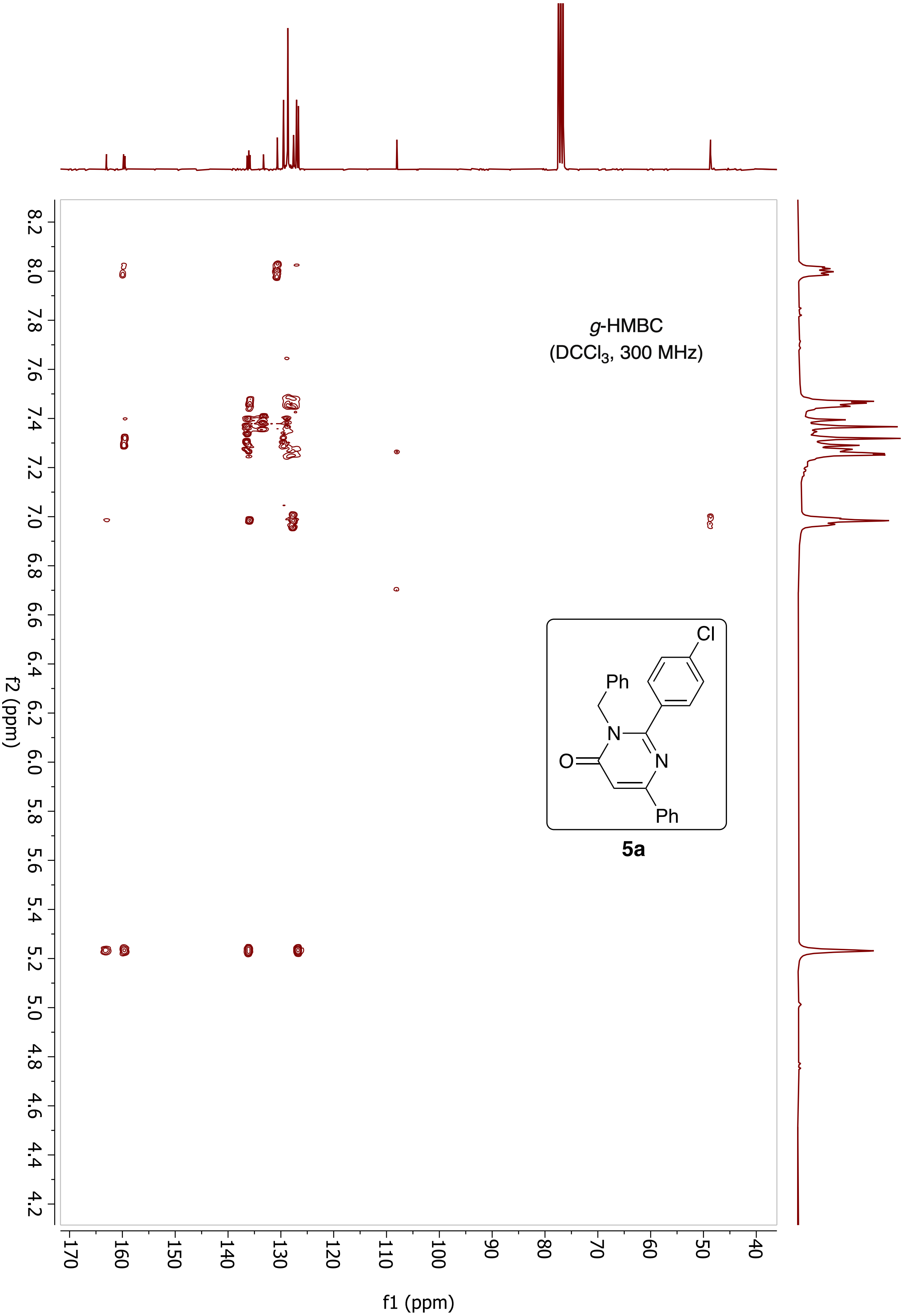
g-HMBC
(DCCl3, 300 MHz)
Cl
Ph
N
O
N
Ph
5a
f2 (ppm)
f1 (ppm)

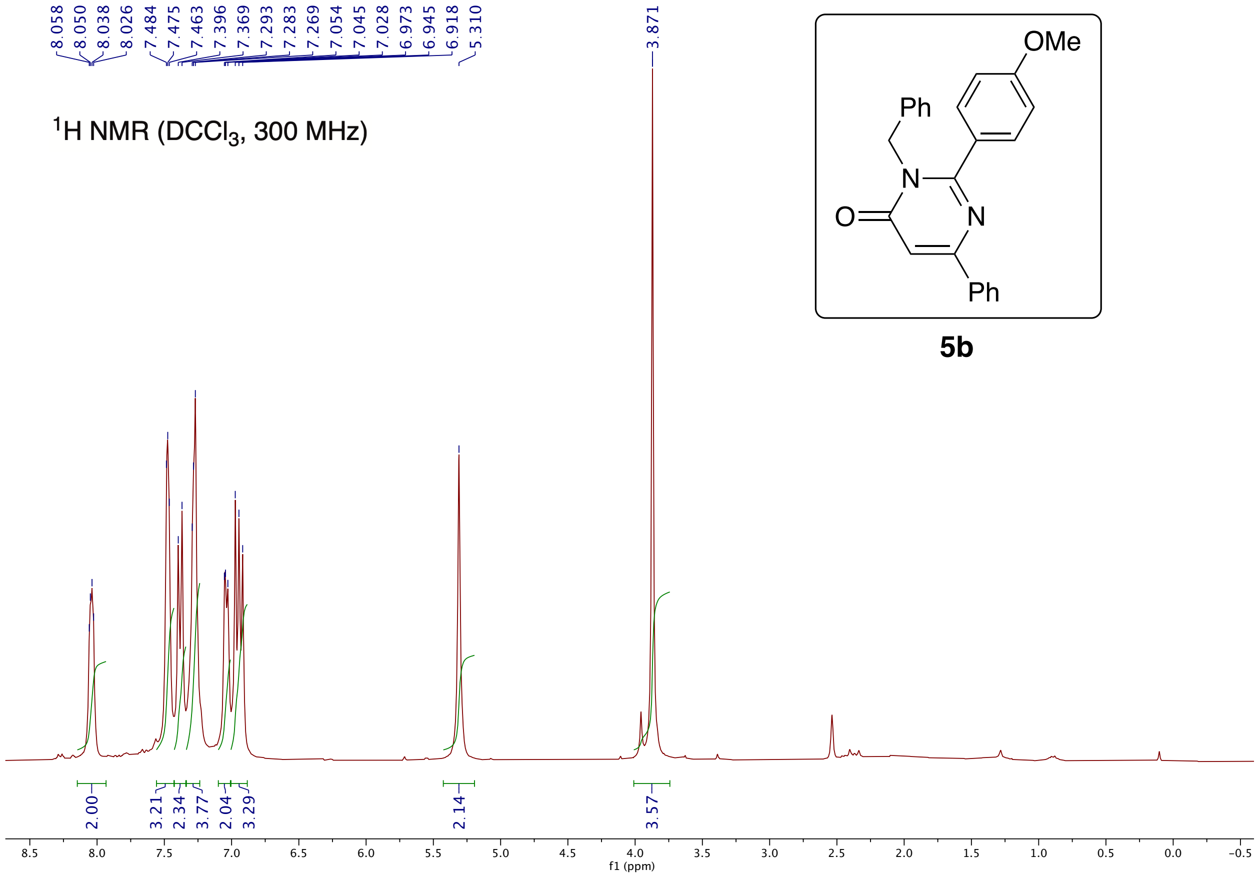
8.058
8.050
8.038
8.026
7.484
7.475
7.463
7.396
7.369
7.293
7.283
7.269
7.054
7.045
7.028
6.973
6.945
6.918
5.310
3.871
^{1}H NMR (DCCl$_3$, 300 MHz)
OMe
Ph
N
N
O
Ph
2.00
3.21
2.34
3.77
2.04
3.29
2.14
3.57
f1 (ppm)

5b

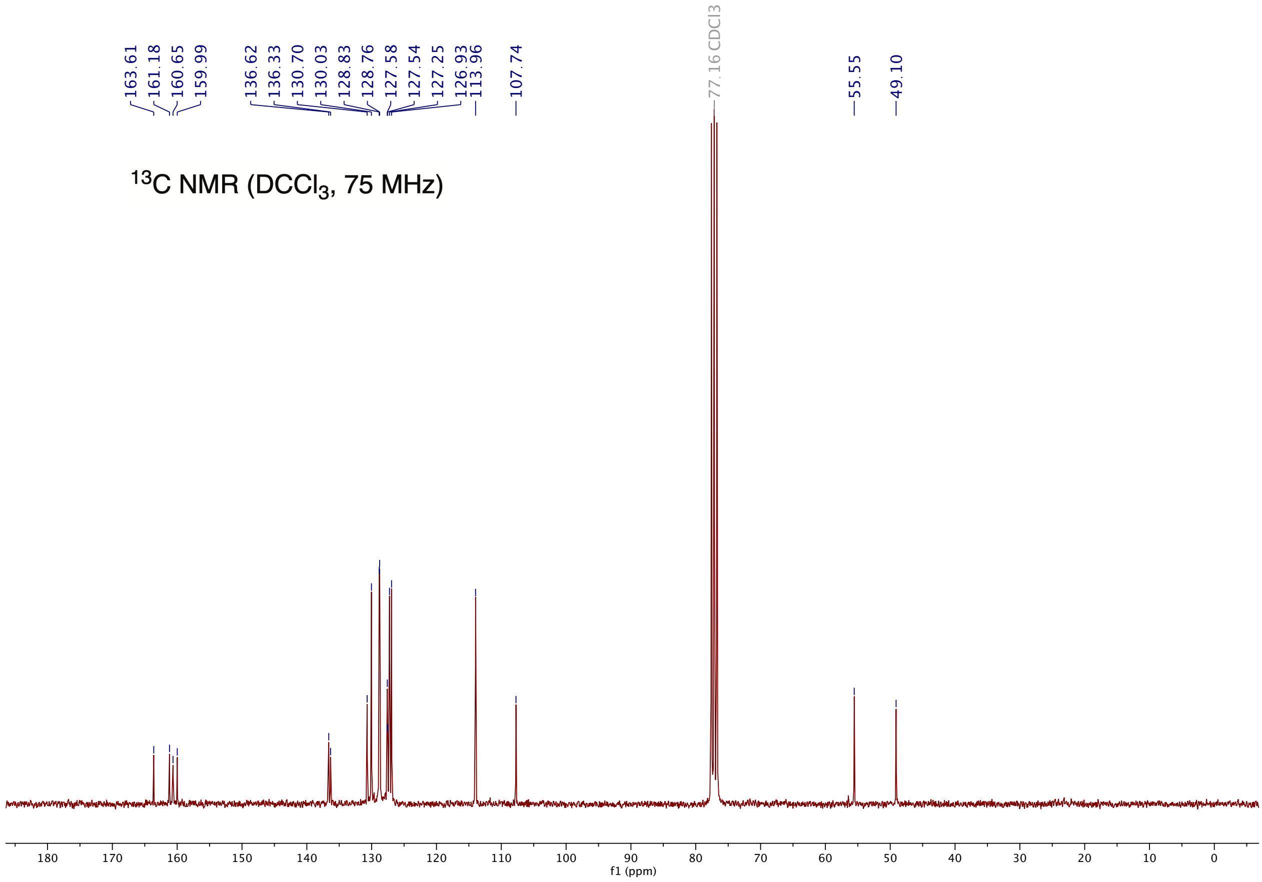
163.61
161.18
160.65
159.99
136.62
136.33
130.70
130.03
128.83
128.76
127.58
127.54
127.25
126.93
113.96
107.74
77.16 CDCl3
55.55
49.10
^{13}C NMR (DCCl$_3$, 75 MHz)
f1 (ppm)

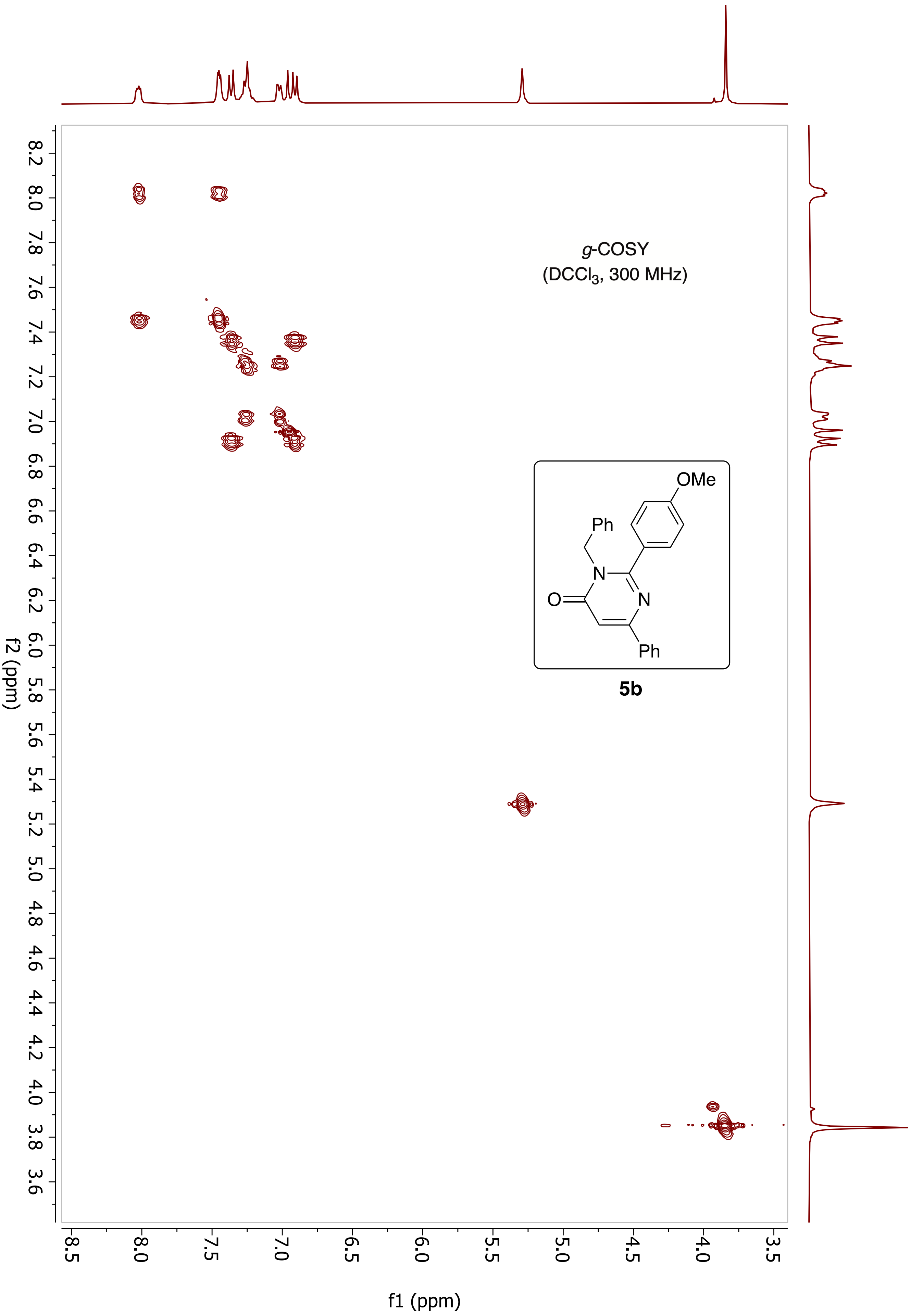

g-COSY
(DCCl3, 300 MHz)
OMe
Ph
N
N
O
Ph
5b
f1 (ppm)
f2 (ppm)

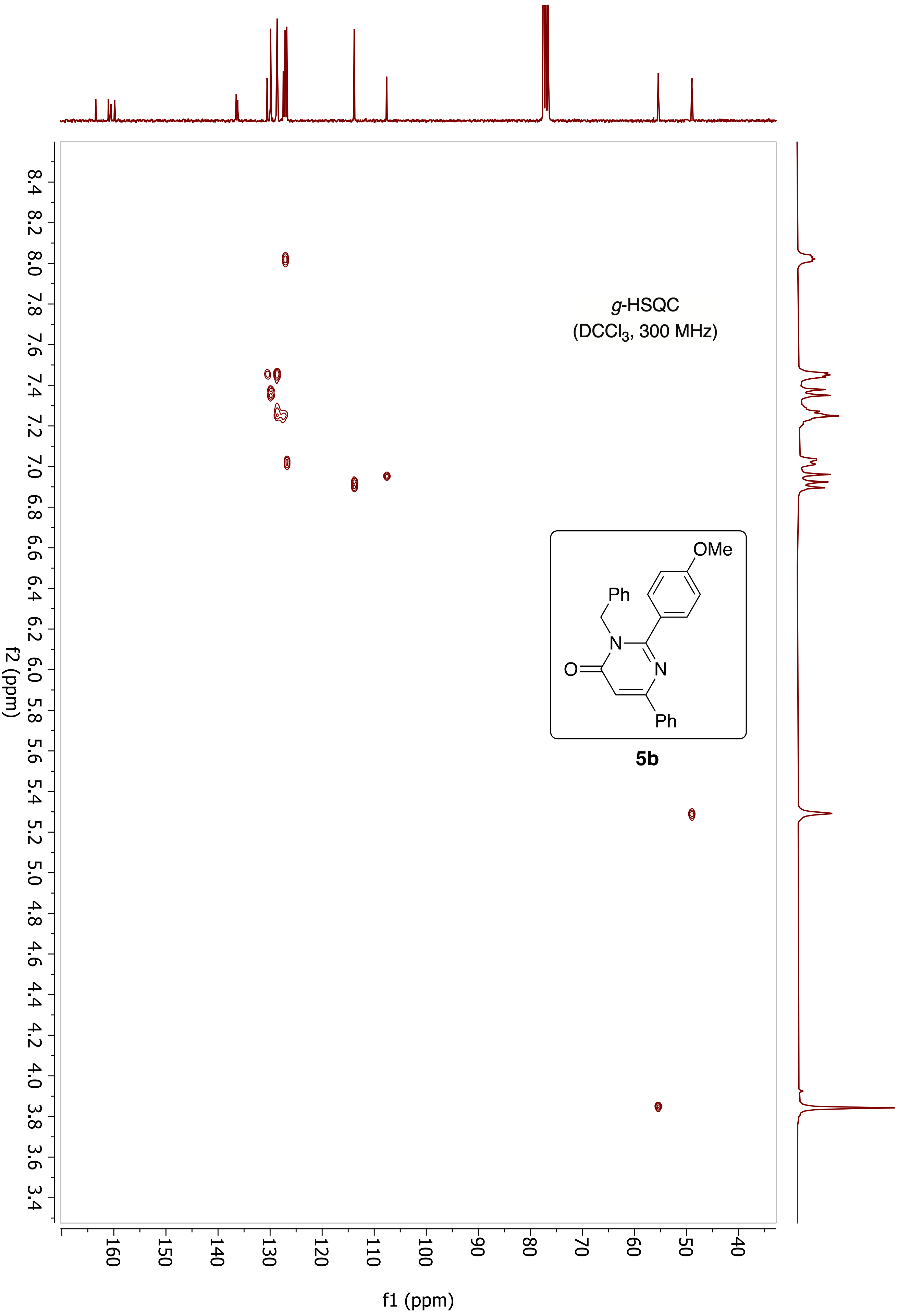
g-HSQC
(DCCl3, 300 MHz)
OMe
Ph
N
O
N
Ph
5b
f2 (ppm)
f1 (ppm)

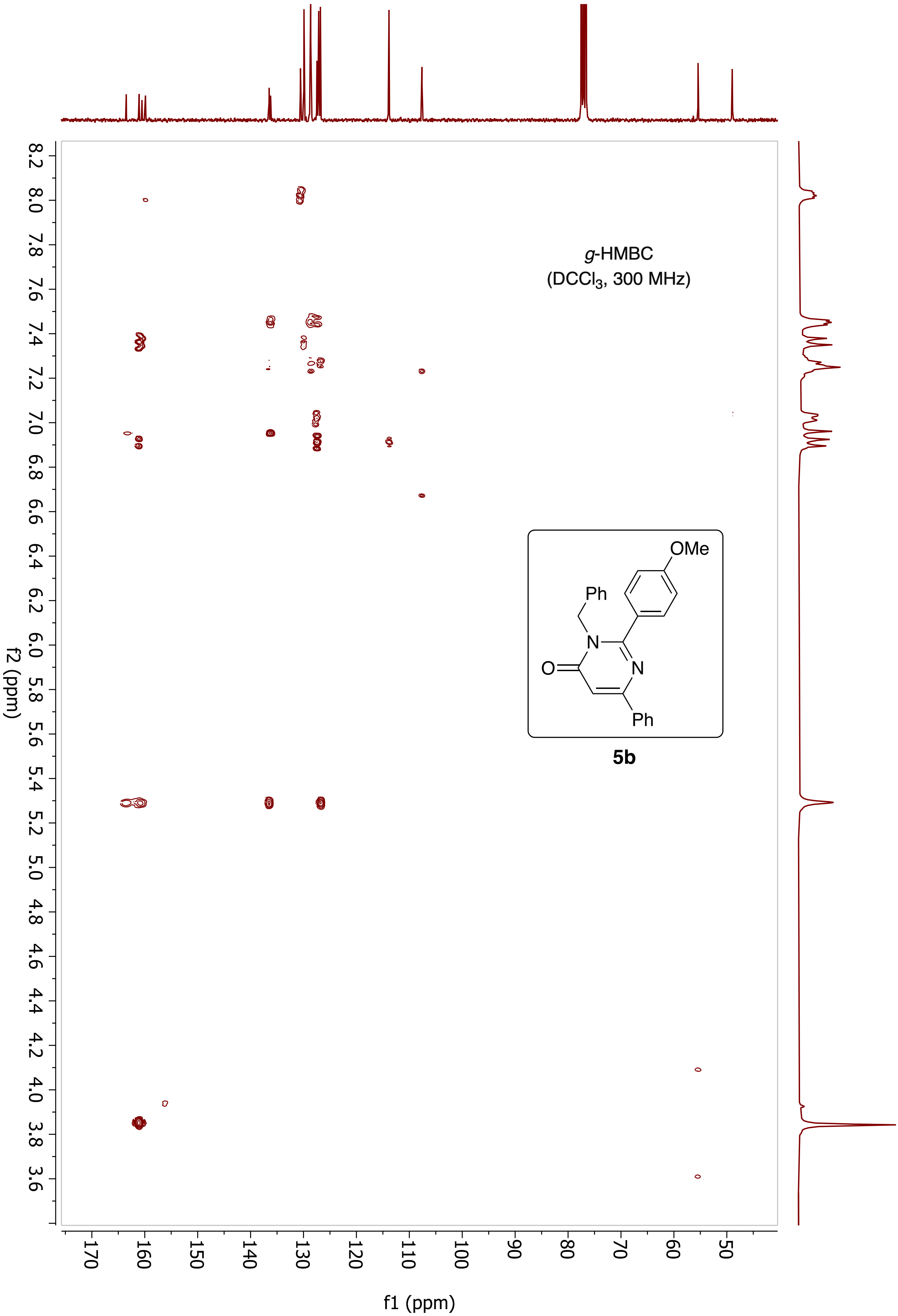
g-HMBC
(DCCl3, 300 MHz)
OMe
Ph
N
O
N
Ph
5b
f2 (ppm)
f1 (ppm)

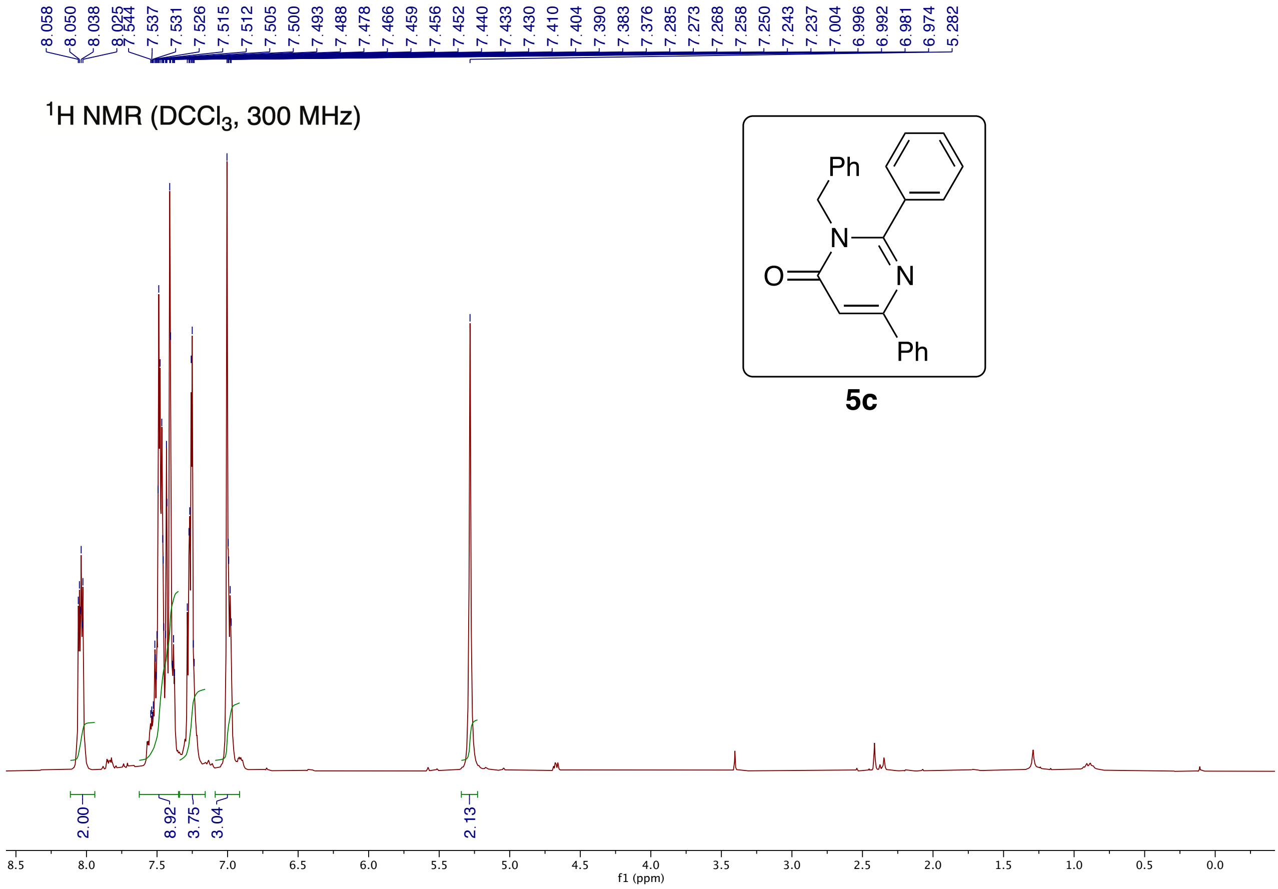
8.058
8.050
8.038
8.025
7.544
7.537
7.531
7.526
7.515
7.512
7.505
7.500
7.493
7.488
7.478
7.466
7.459
7.456
7.452
7.440
7.433
7.430
7.410
7.404
7.390
7.383
7.376
7.285
7.273
7.268
7.258
7.250
7.243
7.237
7.004
6.996
6.992
6.981
6.974
5.282
^{1}H NMR (DCCl$_3$, 300 MHz)
Ph
N
O
N
Ph
5c
2.00
8.92
3.75
3.04
2.13
8.5
8.0
7.5
7.0
6.5
6.0
5.5
5.0
4.5
4.0
3.5
3.0
2.5
2.0
1.5
1.0
0.5
0.0
f1 (ppm)

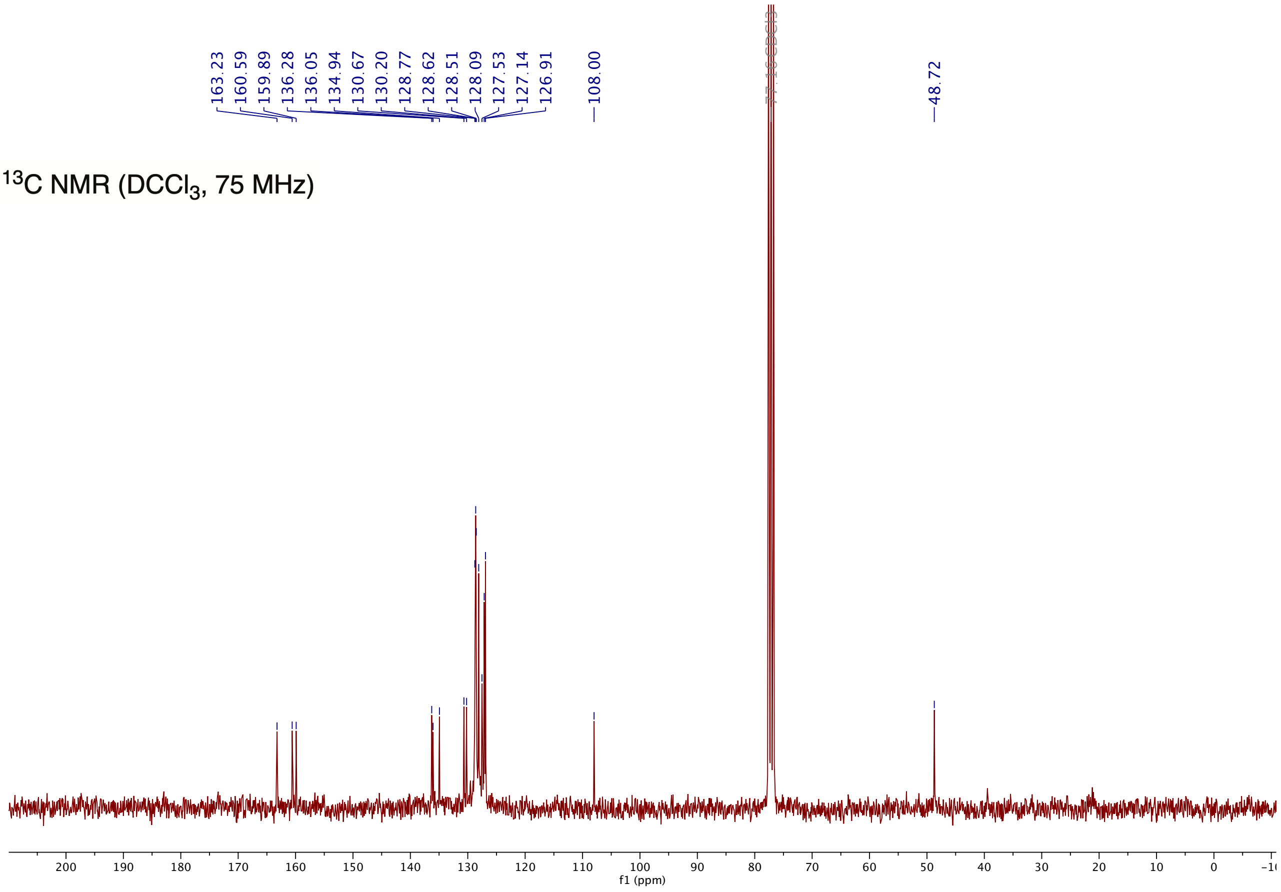
163.23
160.59
159.89
136.28
136.05
134.94
130.67
130.20
128.77
128.62
128.51
128.09
127.53
127.14
126.91
108.00
48.72
^{13}C NMR (DCCl$_3$, 75 MHz)
200
190
180
170
160
150
140
130
120
110
100
90
80
70
60
50
40
30
20
10
0
f1 (ppm)

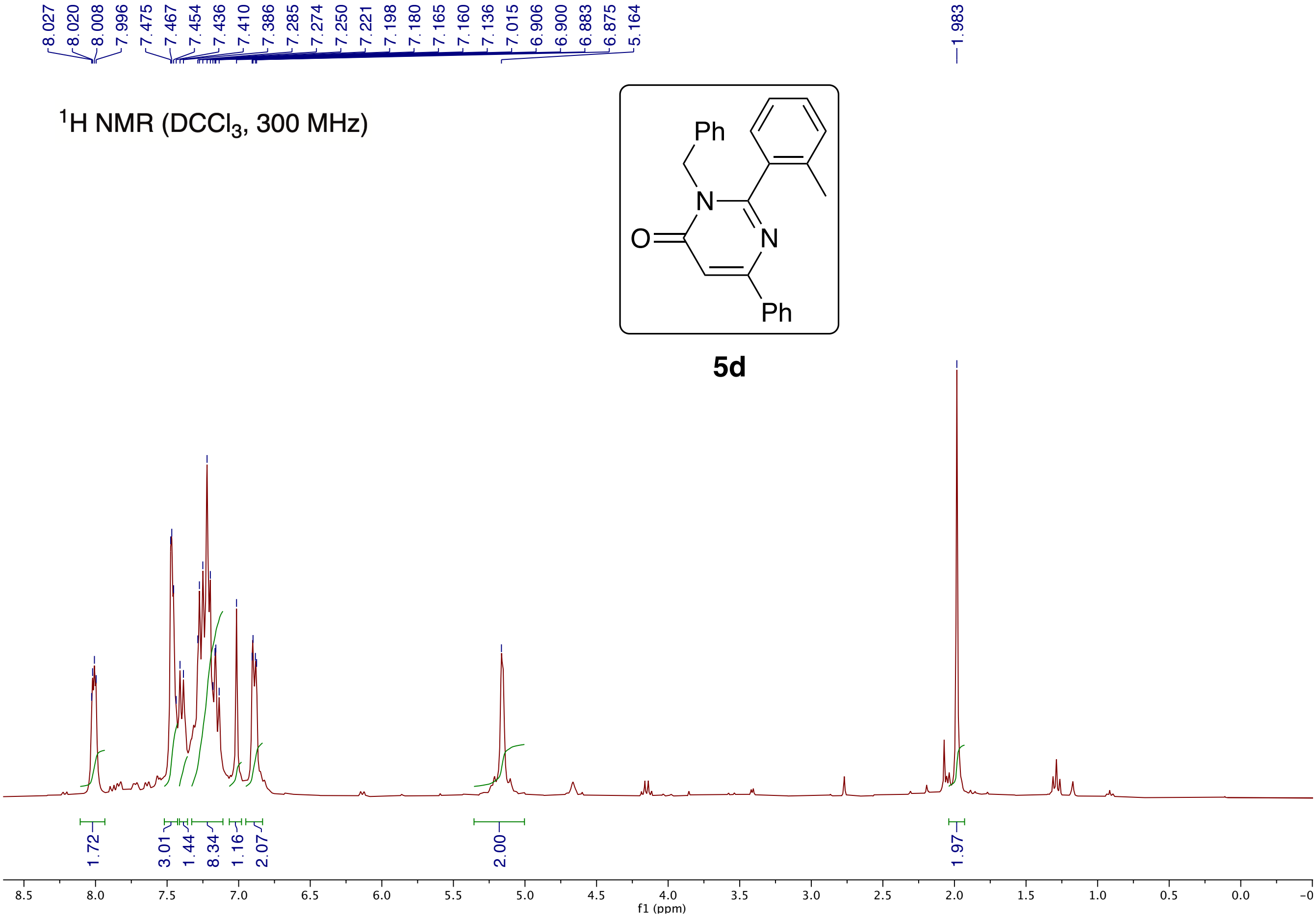

^{1}H NMR ($DCCl_3$, 300 MHz)
5d

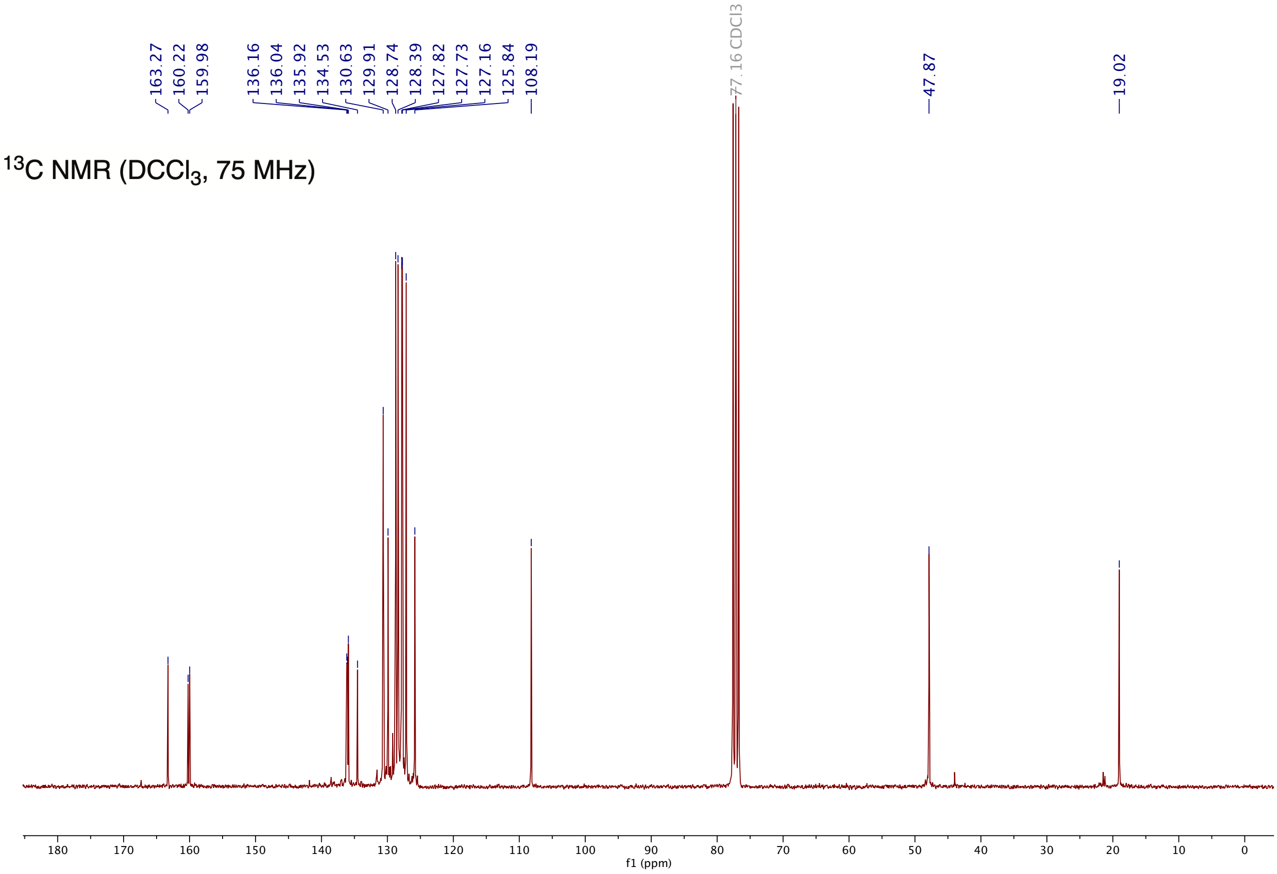

^{13}C NMR ($DCCl_3$, 75 MHz)

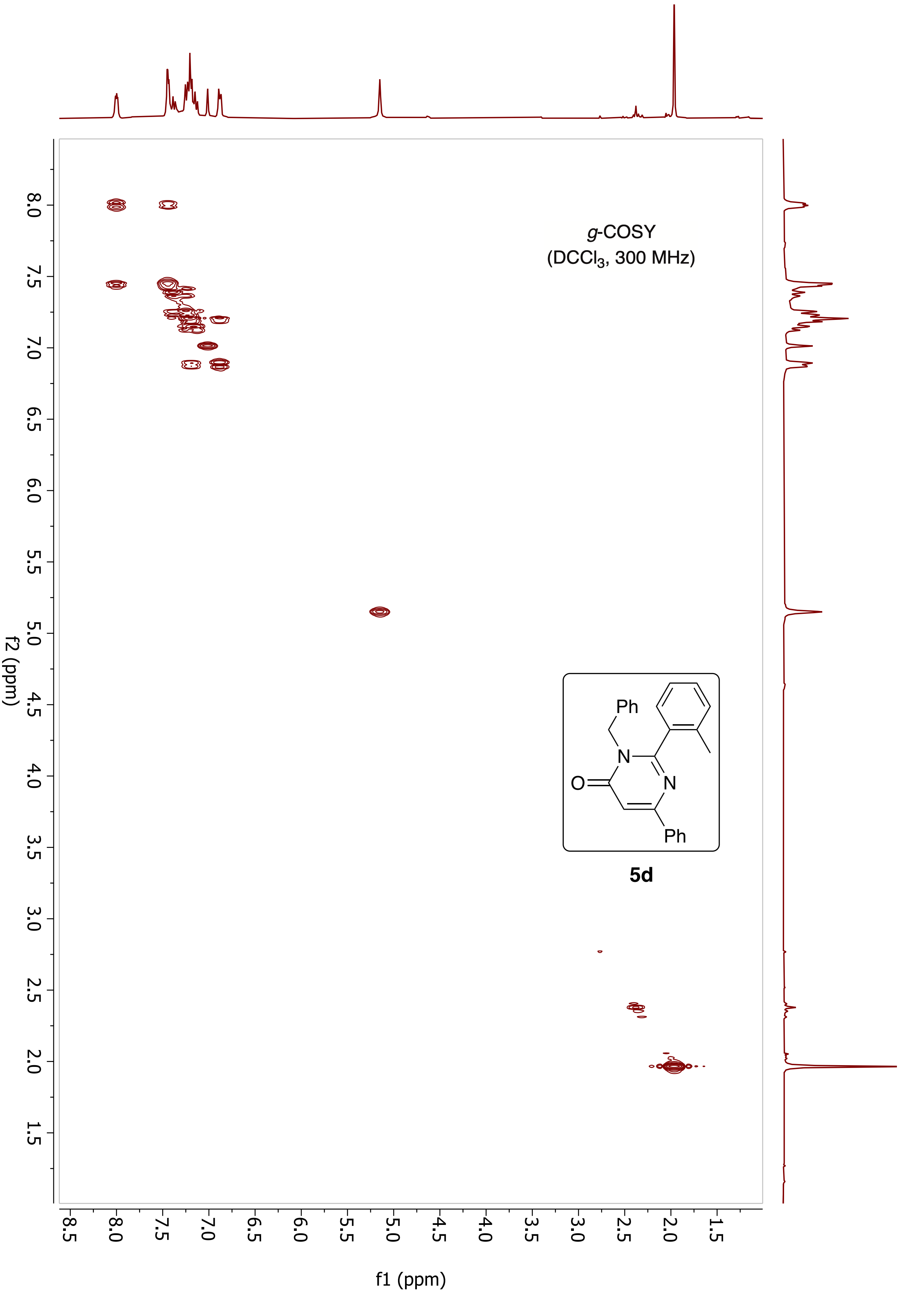
g-COSY
(DCCl3, 300 MHz)
5d
f1 (ppm)
f2 (ppm)

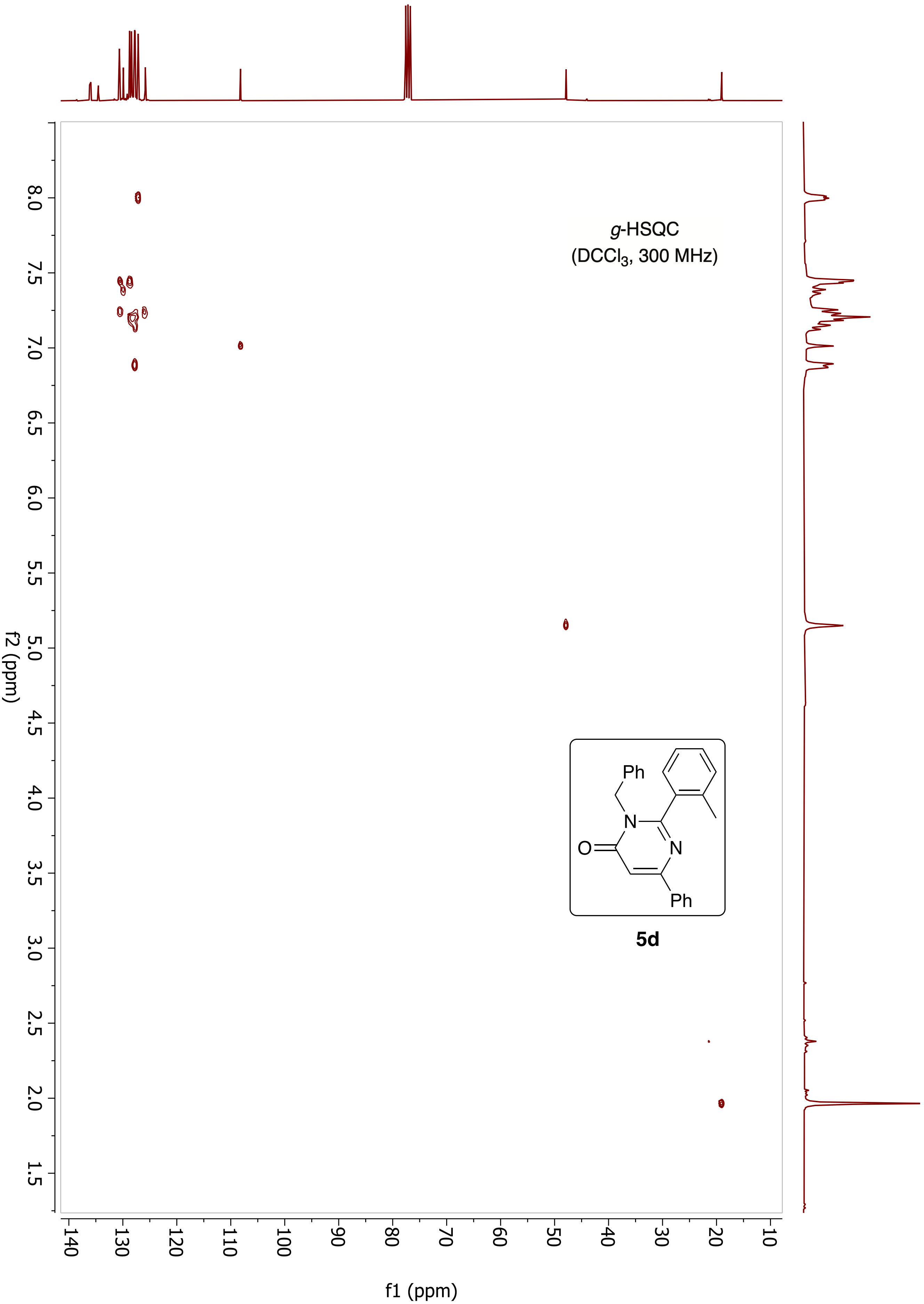
g-HSQC
(DCCl3, 300 MHz)
Ph
N
O
N
Ph
5d
f1 (ppm)
f2 (ppm)

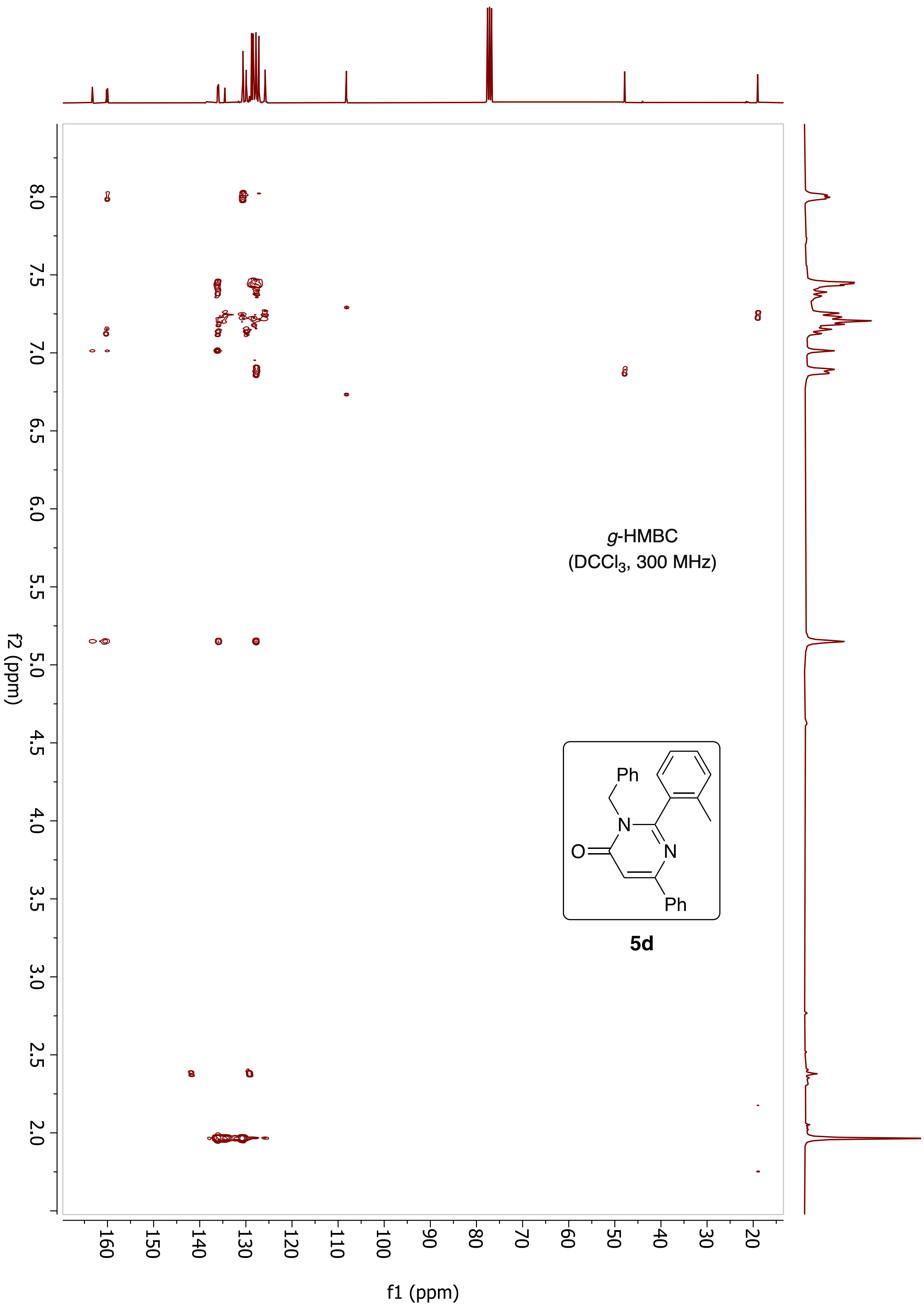

g-HMBC
(DCCl3, 300 MHz)
Ph
N
O
N
Ph
5d
f1 (ppm)
f2 (ppm)

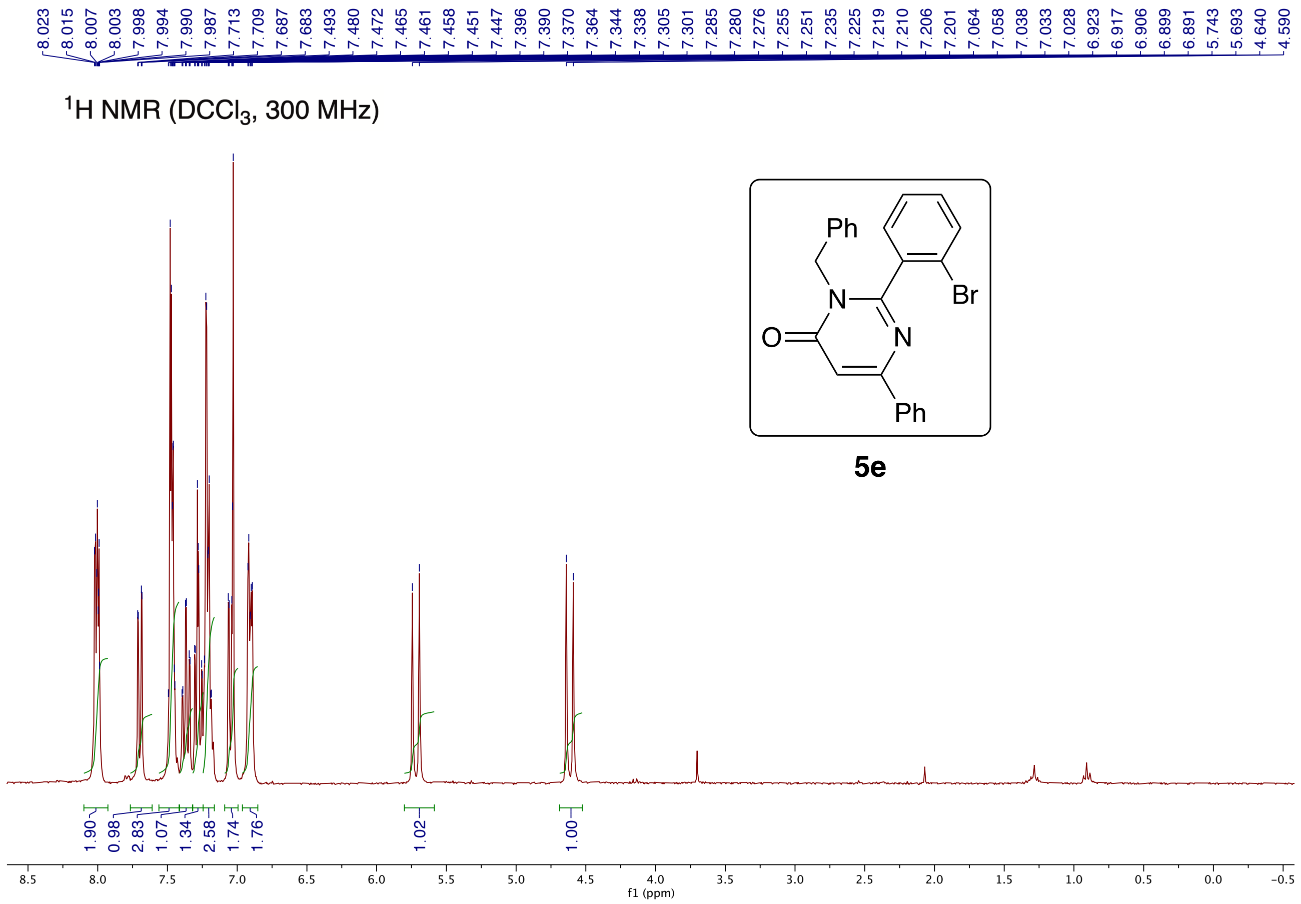
8.023 8.015 8.007 8.003 7.998 7.994 7.990 7.987 7.713 7.709 7.687 7.683 7.493 7.480 7.472 7.465 7.461 7.458 7.451 7.447 7.396 7.390 7.370 7.364 7.344 7.338 7.305 7.301 7.285 7.280 7.276 7.255 7.251 7.235 7.225 7.219 7.210 7.206 7.201 7.064 7.058 7.038 7.033 7.028 6.923 6.917 6.906 6.899 6.891 5.743 5.693 4.640 4.590
^{1}H NMR (DCCl$_3$, 300 MHz)
Ph
Br
N
O
N
Ph
5e
1.90 0.98 2.83 1.07 1.34 2.58 1.74 1.76 1.02 1.00
f1 (ppm)

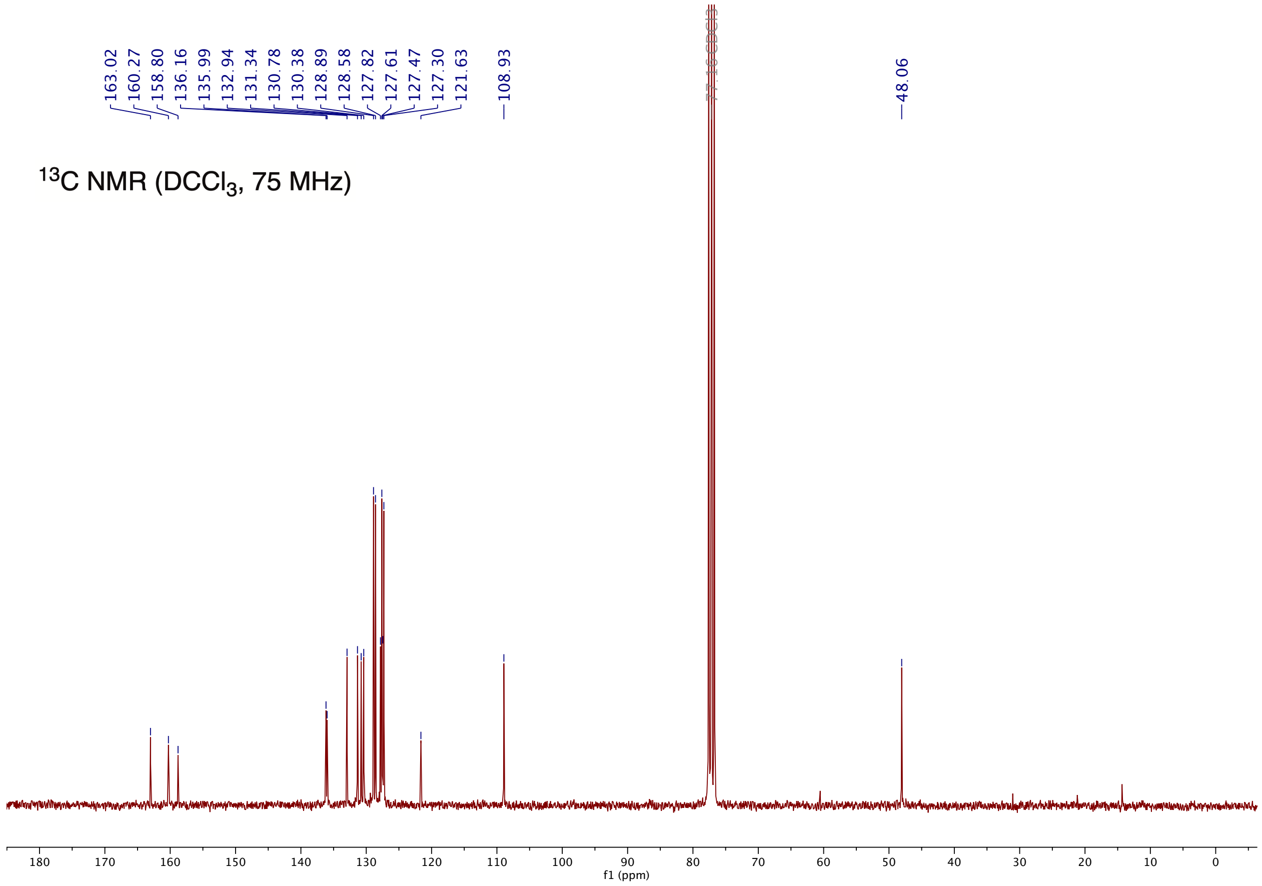
163.02 160.27 158.80 136.16 135.99 132.94 131.34 130.78 130.38 128.89 128.58 127.82 127.61 127.47 127.30 121.63 108.93 48.06
^{13}C NMR (DCCl$_3$, 75 MHz)
f1 (ppm)

^{1}H NMR ($DCCl_3$, 300 MHz)

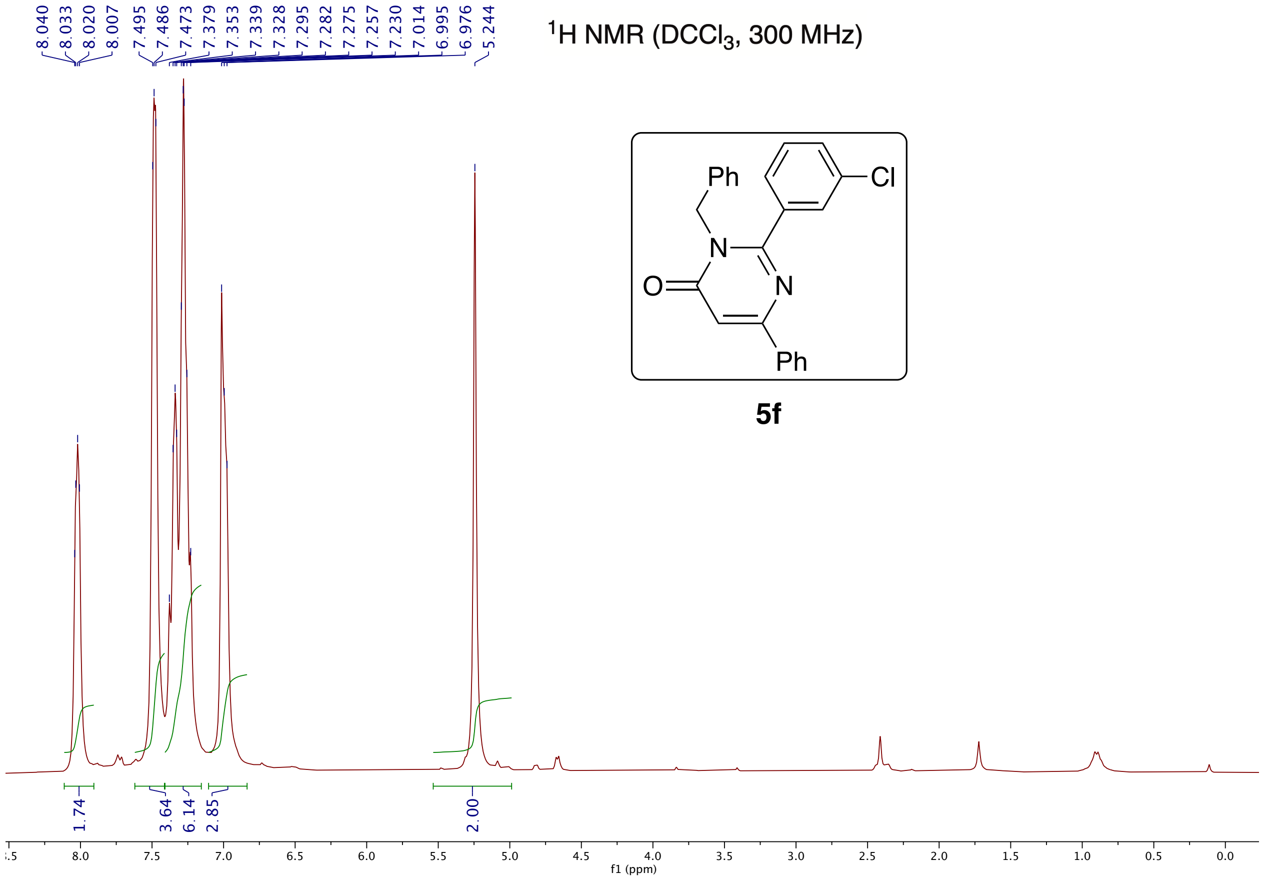

5f

^{13}C NMR ($DCCl_3$, 75 MHz)

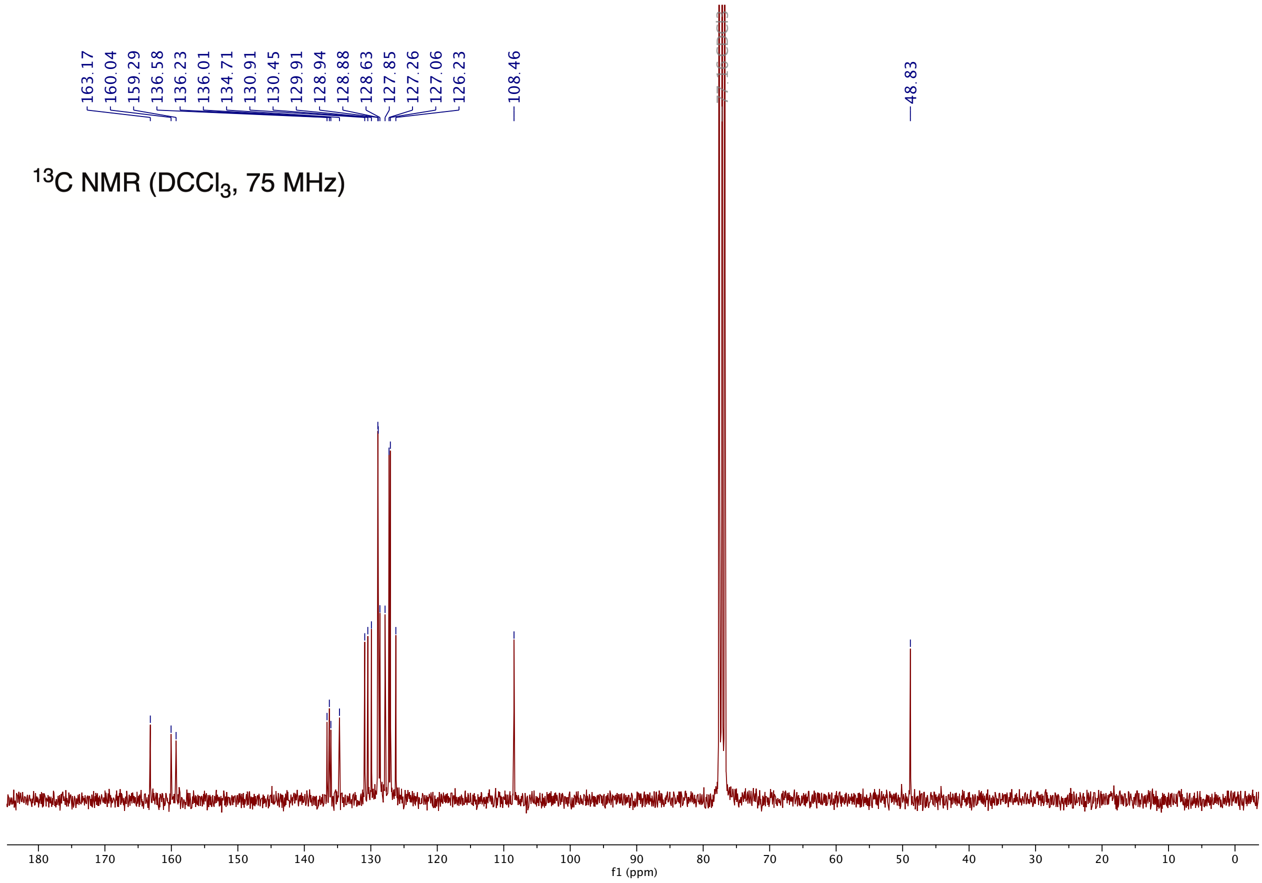

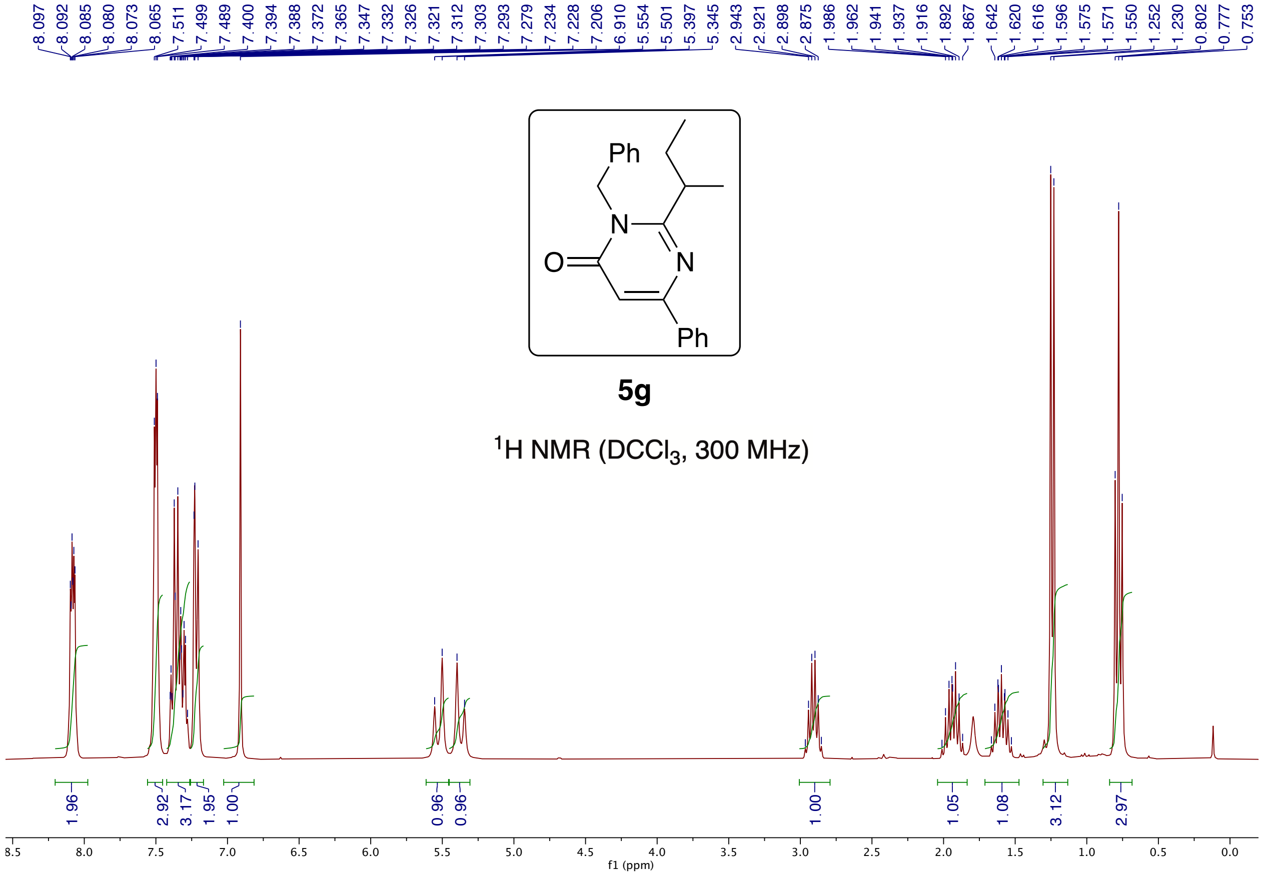
8.097 8.092 8.085 8.080 8.073 8.065 7.511 7.499 7.489 7.400 7.394 7.388 7.372 7.365 7.347 7.332 7.326 7.321 7.312 7.303 7.293 7.279 7.234 7.228 7.206 6.910 5.554 5.501 5.397 5.345 2.943 2.921 2.898 2.875 1.986 1.962 1.941 1.937 1.916 1.892 1.867 1.642 1.620 1.616 1.596 1.575 1.571 1.550 1.252 1.230 0.802 0.777 0.753
Ph
N
N
O
Ph
5g
^{1}H NMR ($DCCl_3$, 300 MHz)
1.96 2.92 3.17 1.95 1.00 0.96 0.96 1.00 1.05 1.08 3.12 2.97
f1 (ppm)

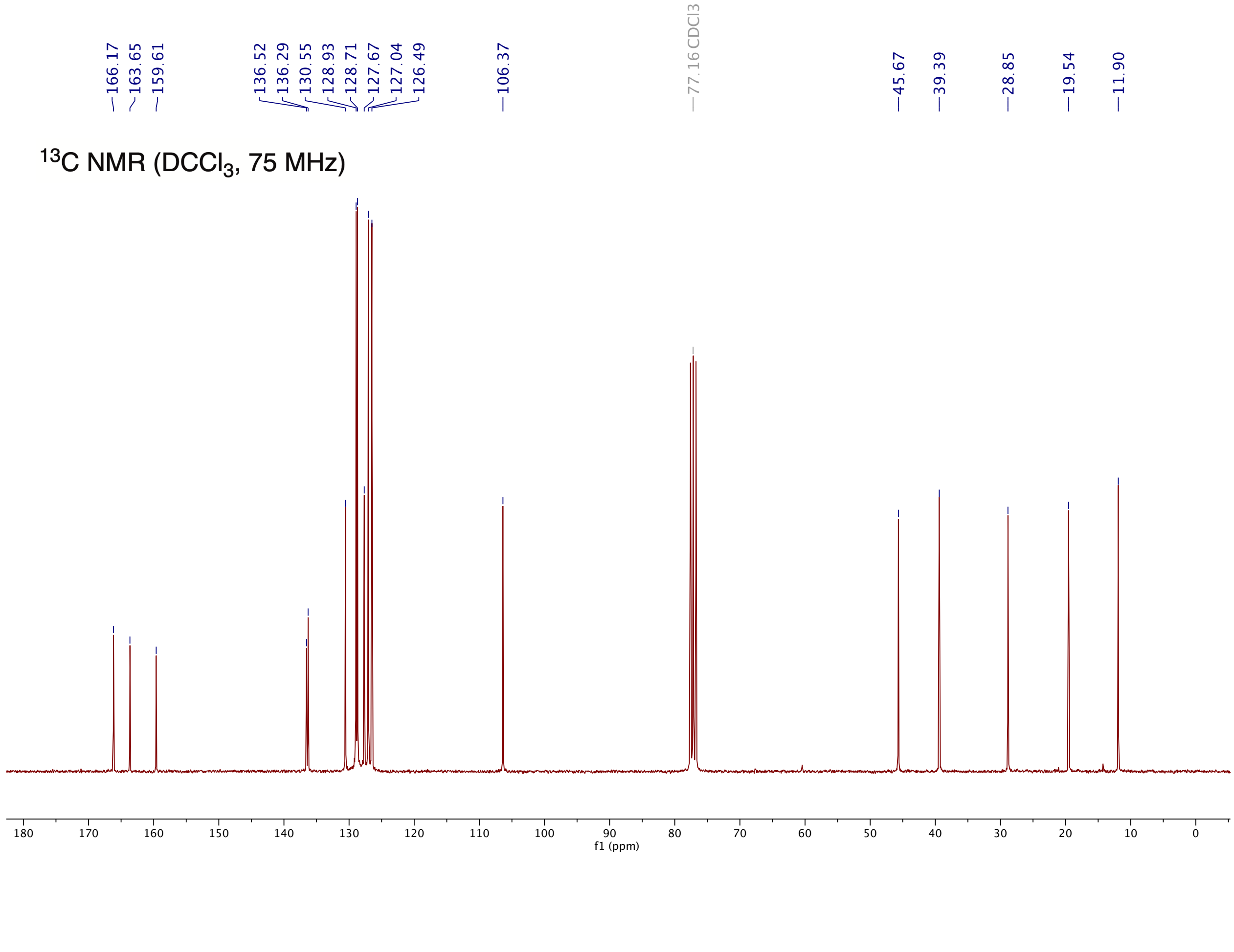
166.17 163.65 159.61 136.52 136.29 130.55 128.93 128.71 127.67 127.04 126.49 106.37 77.16 CDCl3 45.67 39.39 28.85 19.54 11.90
^{13}C NMR ($DCCl_3$, 75 MHz)
f1 (ppm)

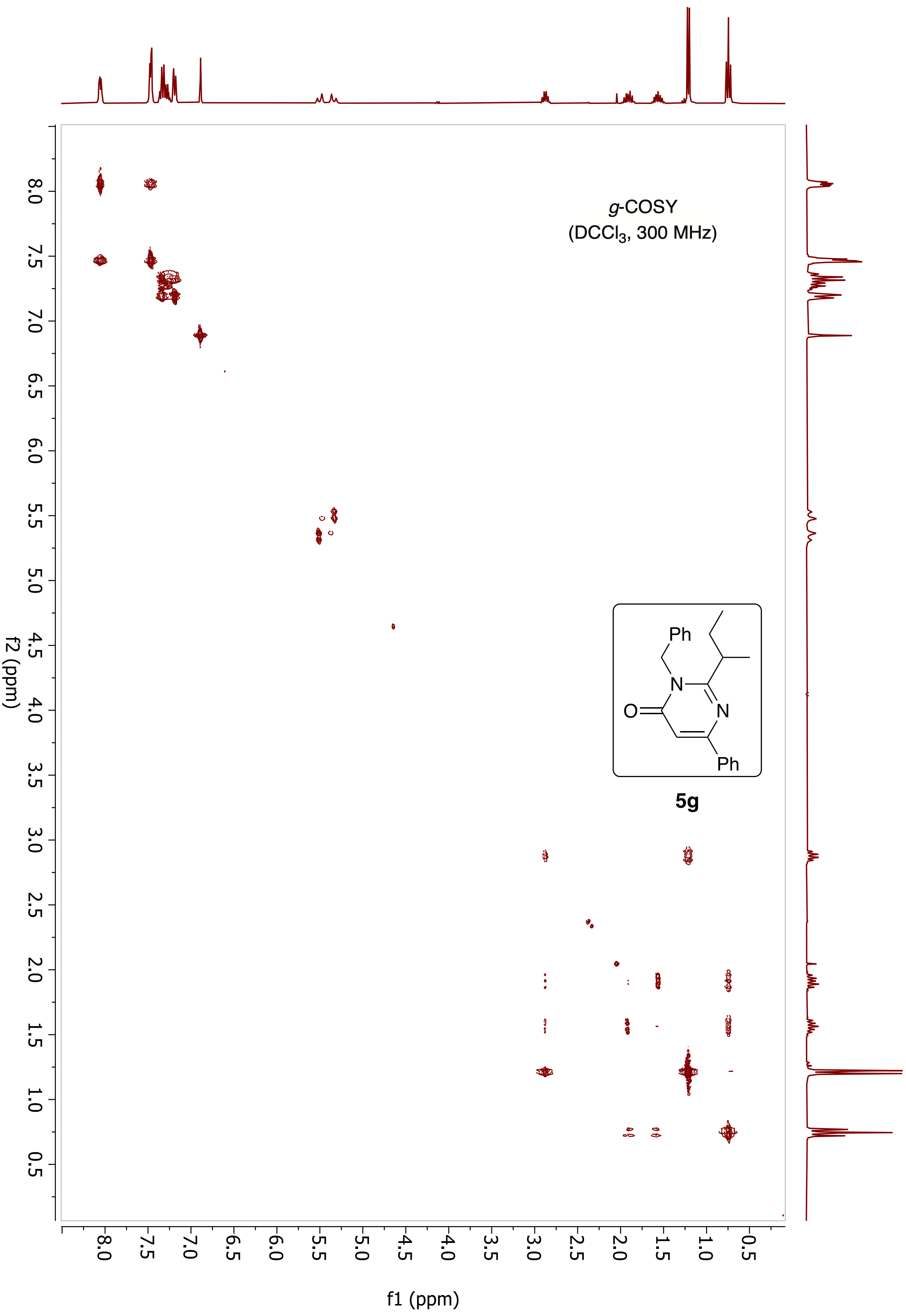
g-COSY
(DCCl3, 300 MHz)
Ph
N
O
N
Ph
5g
f2 (ppm)
f1 (ppm)
8.0
7.5
7.0
6.5
6.0
5.5
5.0
4.5
4.0
3.5
3.0
2.5
2.0
1.5
1.0
0.5

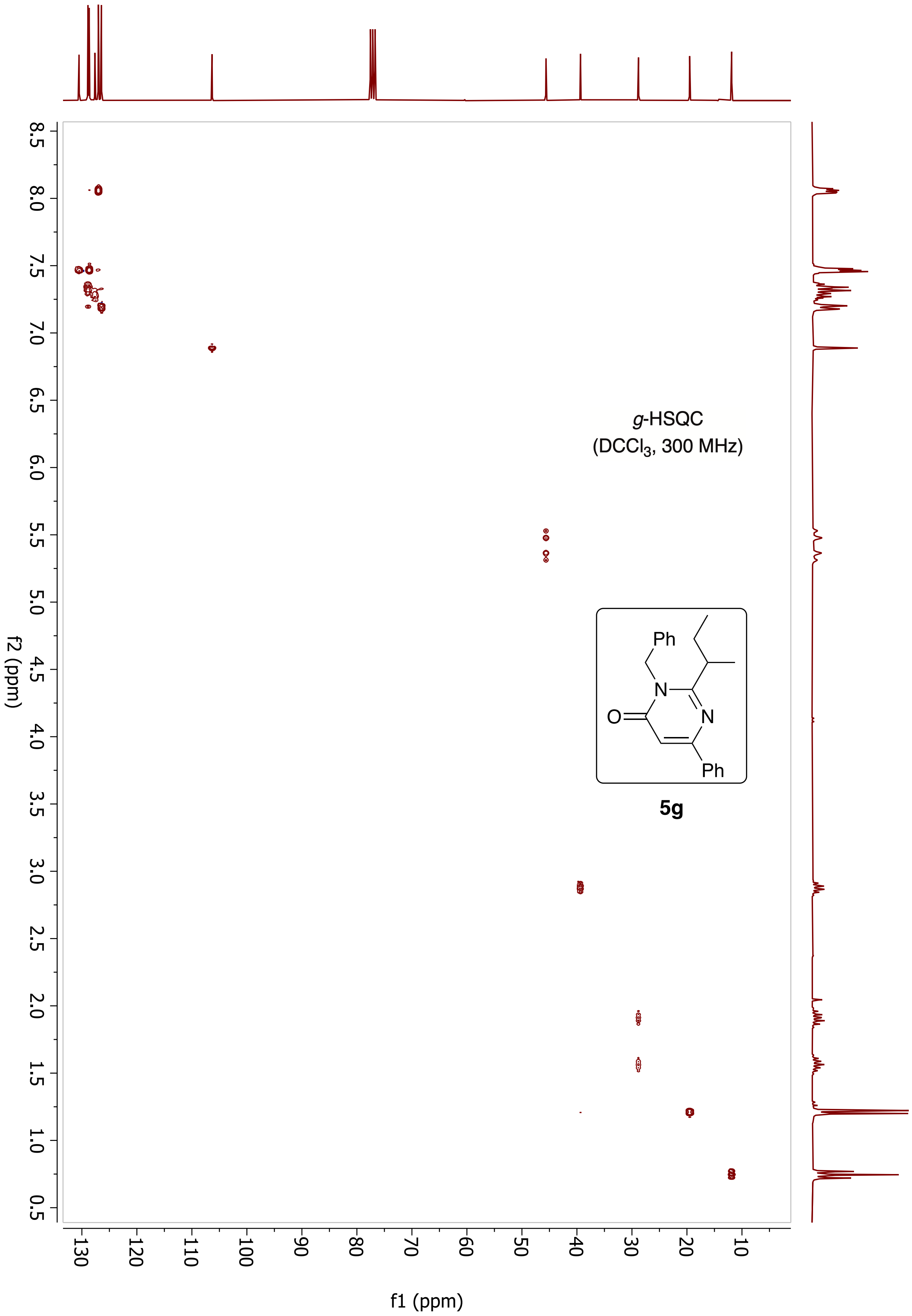
g-HSQC
(DCCl3, 300 MHz)
Ph
N
N
O
Ph
5g
f1 (ppm)
f2 (ppm)

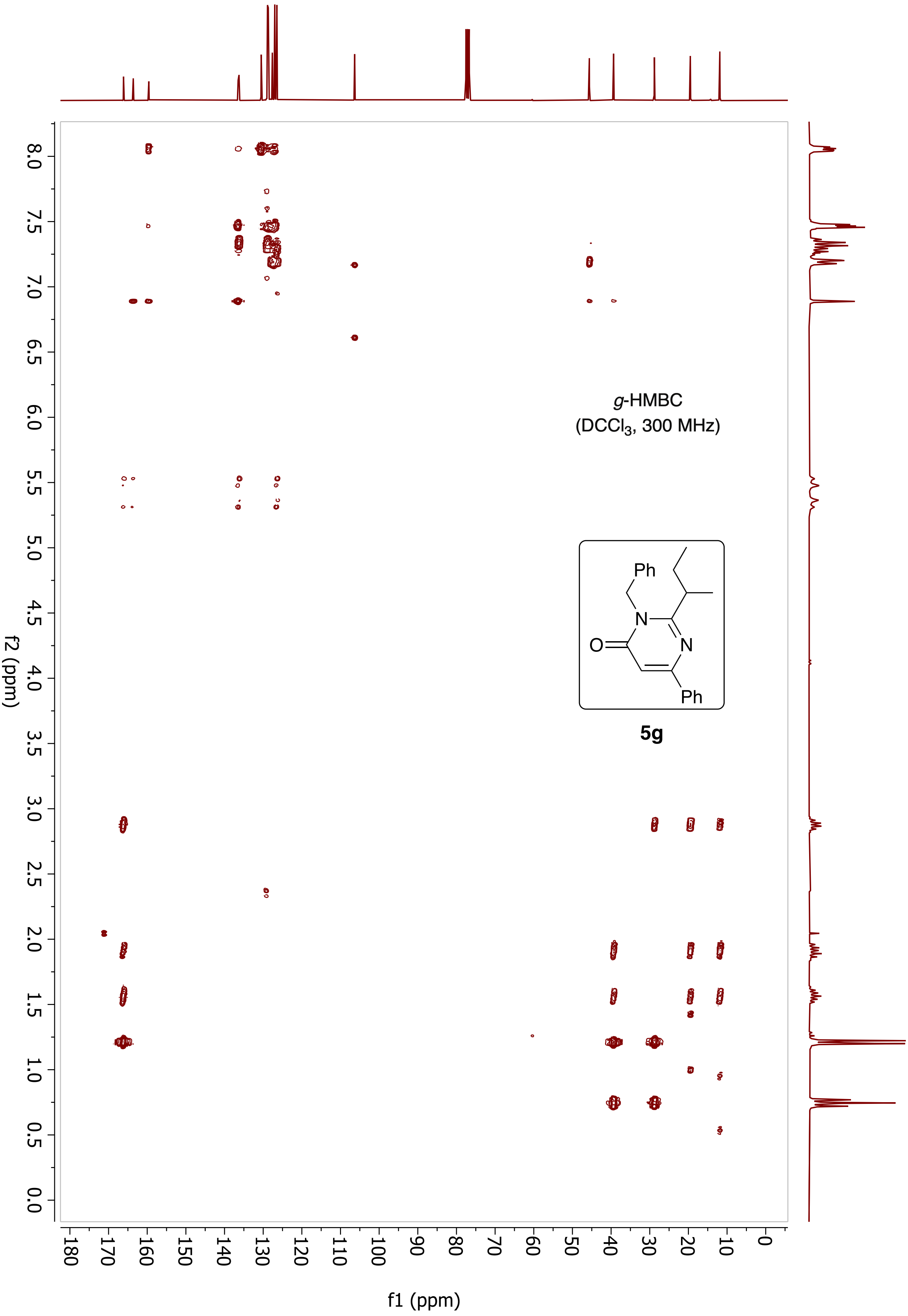

g-HMBC
(DCCl3, 300 MHz)
Ph
N
O
N
Ph
5g
f1 (ppm)
f2 (ppm)

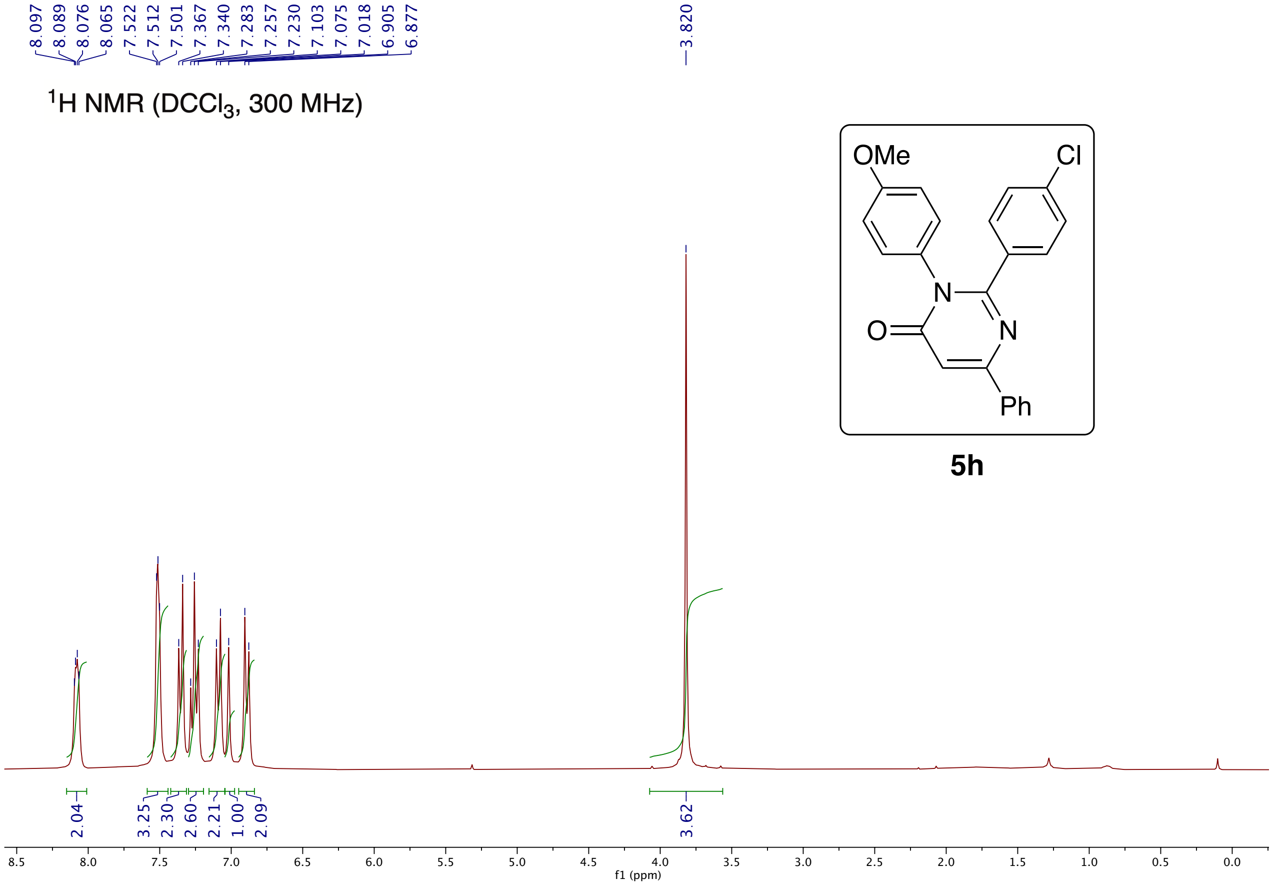

^{1}H NMR ($DCCl_3$, 300 MHz)

5h

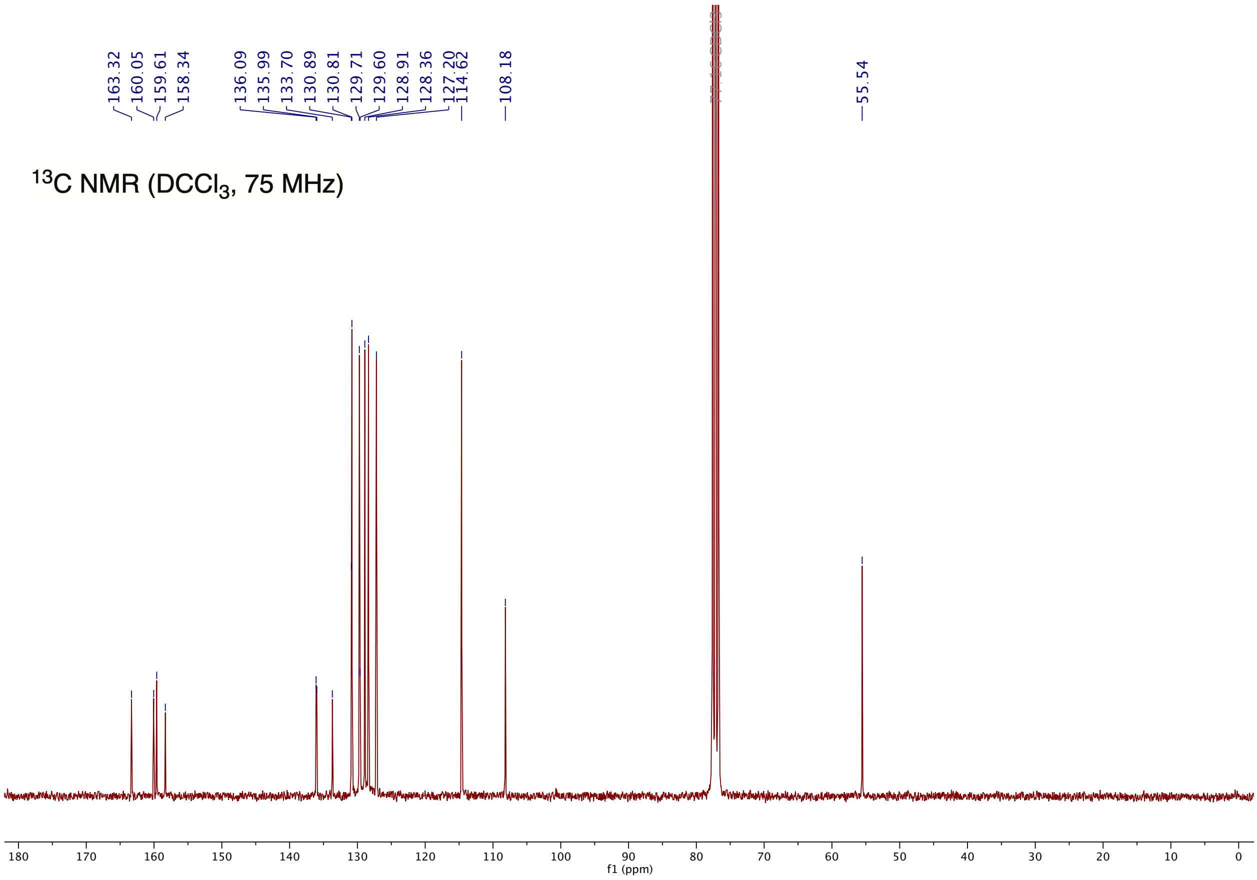

^{13}C NMR ($DCCl_3$, 75 MHz)

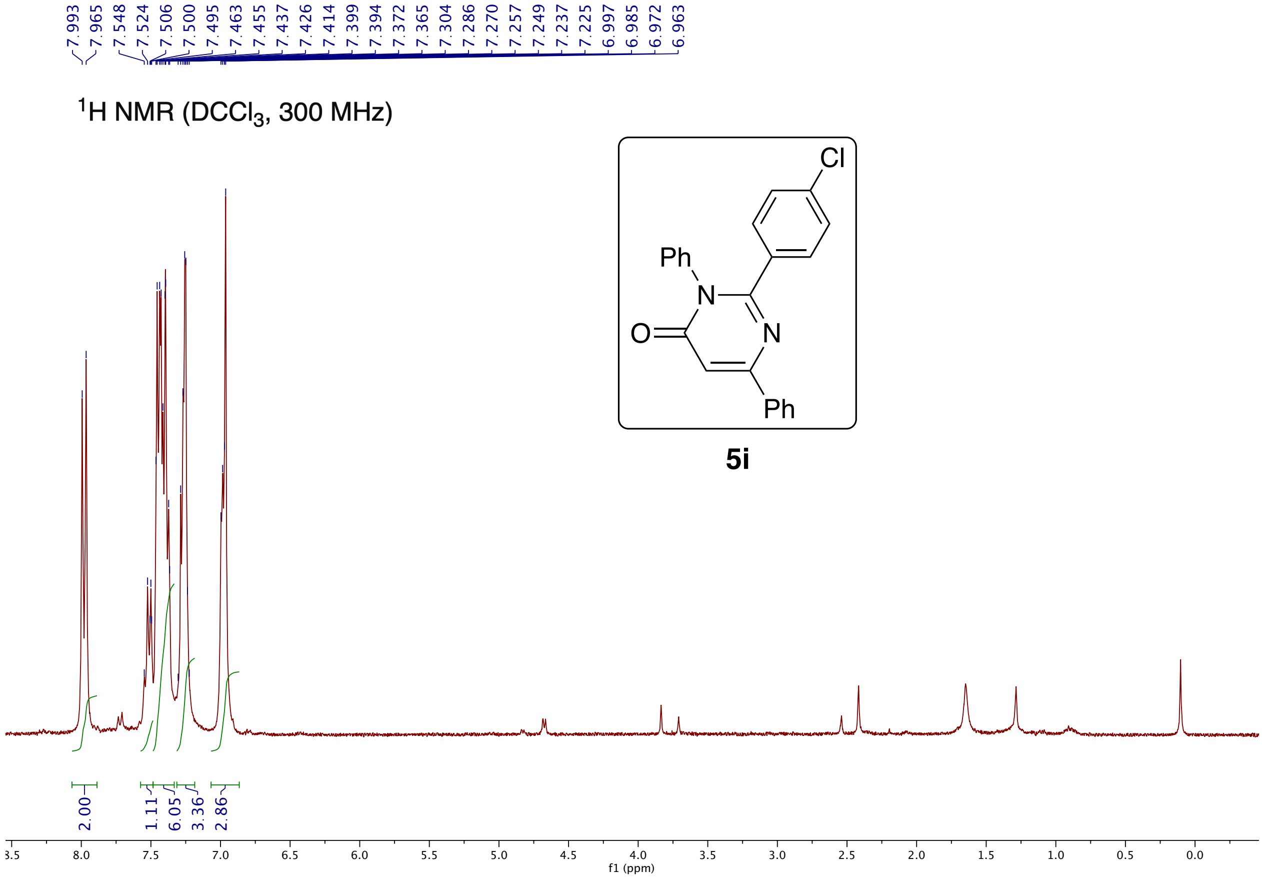
7.993
7.965
7.548
7.524
7.506
7.500
7.495
7.463
7.455
7.437
7.426
7.414
7.399
7.394
7.372
7.365
7.304
7.286
7.270
7.257
7.249
7.237
7.225
6.997
6.985
6.972
6.963
^{1}H NMR ($DCCl_3$, 300 MHz)
2.00
1.11
6.05
3.36
2.86
f1 (ppm)
Cl
Ph
N
N
O
Ph

5i

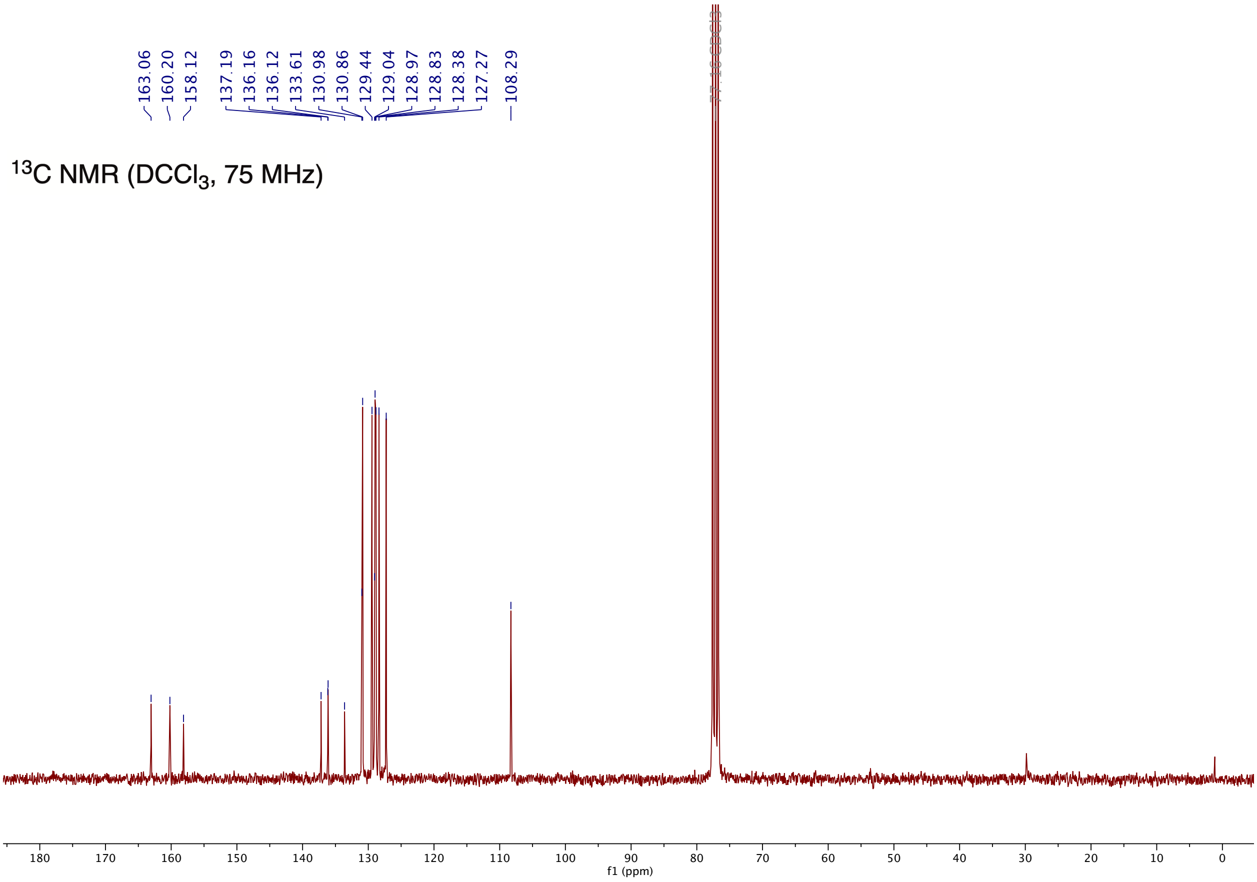
163.06
160.20
158.12
137.19
136.16
136.12
133.61
130.98
130.86
129.44
129.04
128.97
128.83
128.38
127.27
108.29
^{13}C NMR ($DCCl_3$, 75 MHz)
f1 (ppm)

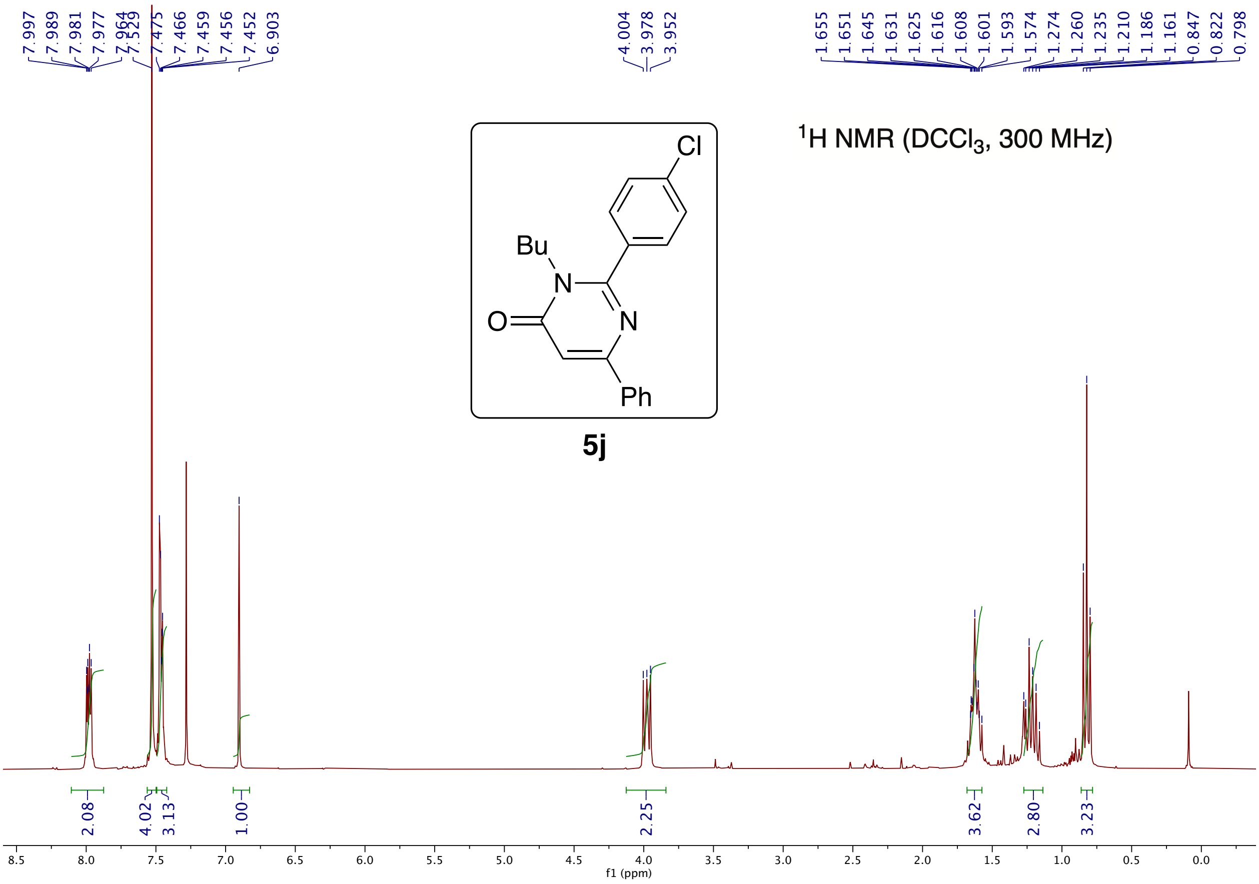

5j

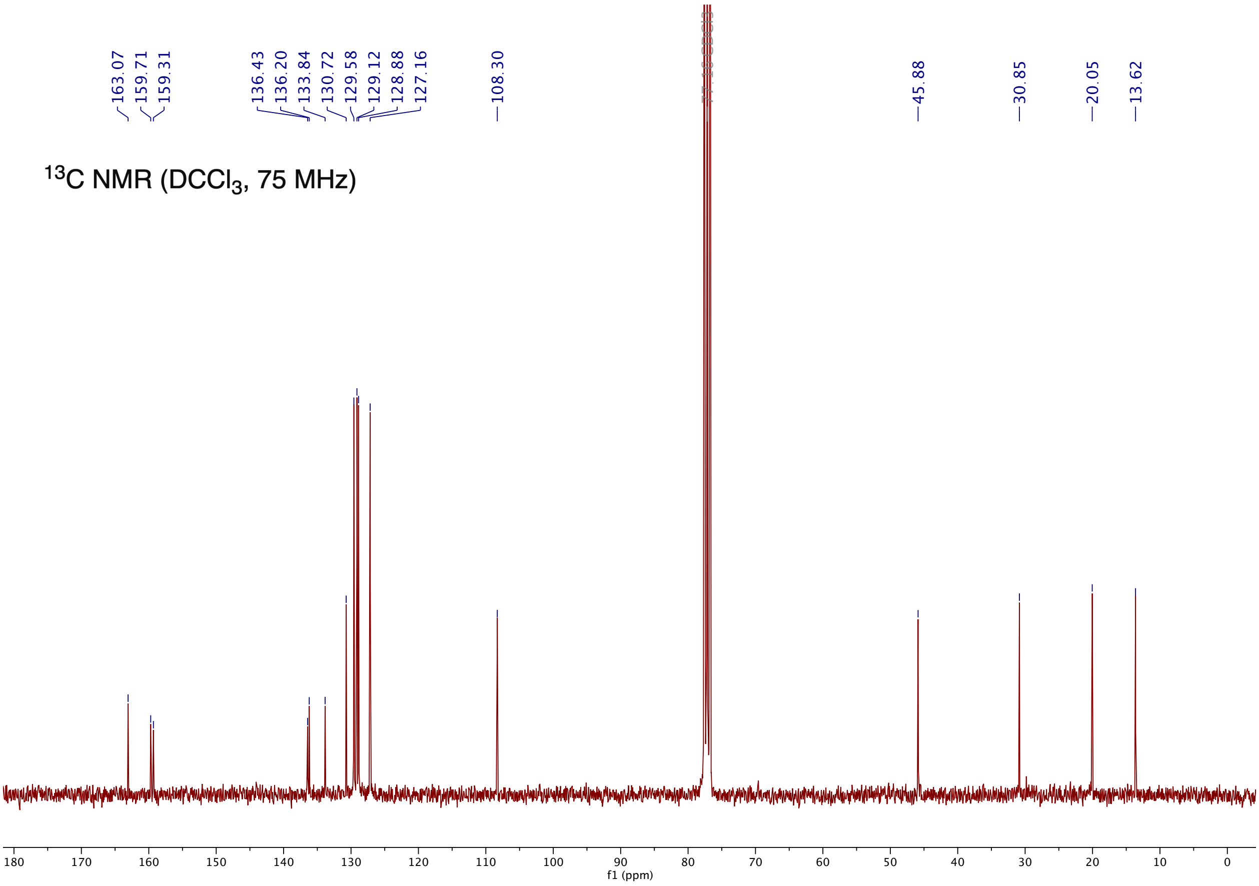

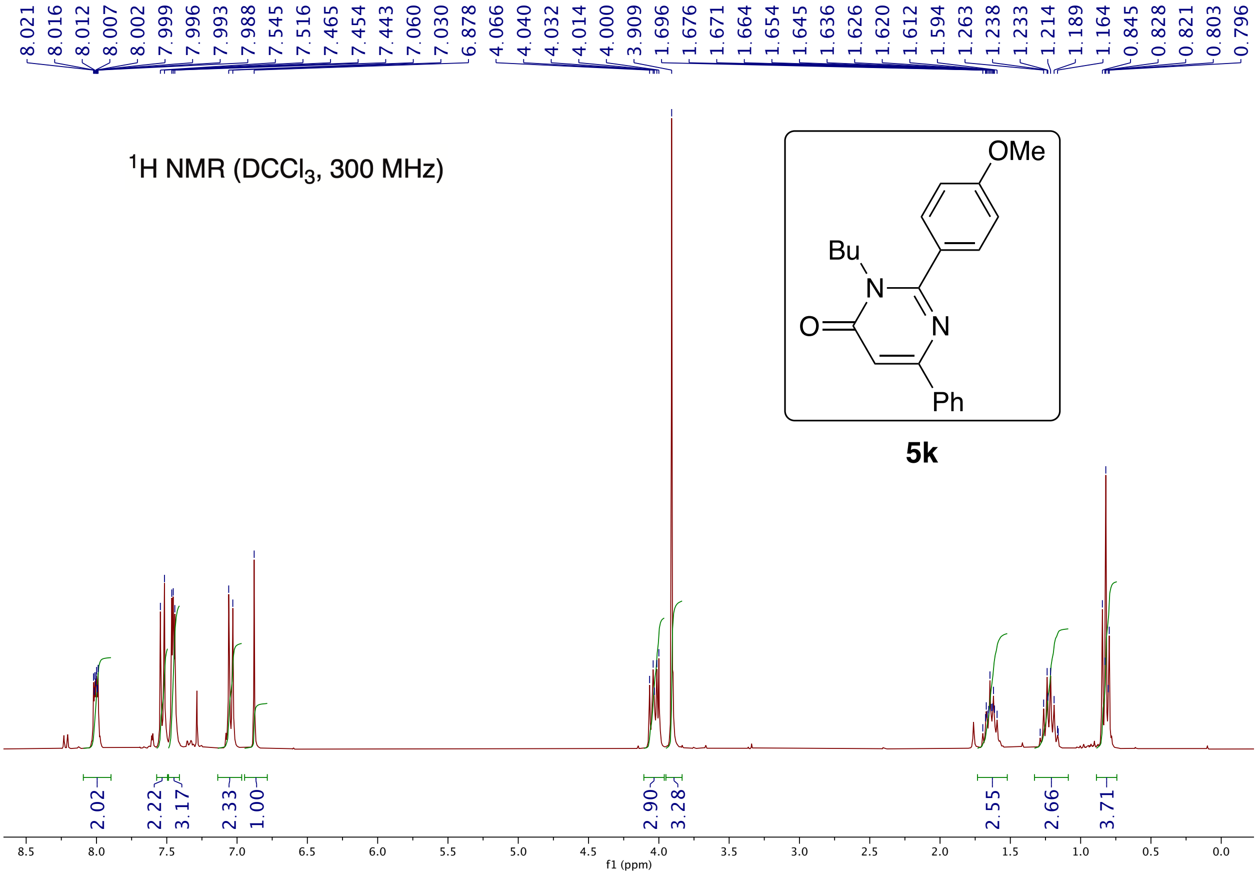
8.021 8.016 8.012 8.007 8.002 7.999 7.996 7.993 7.988 7.545 7.516 7.465 7.454 7.443 7.060 7.030 6.878 4.066 4.040 4.032 4.014 4.000 3.909 1.696 1.676 1.671 1.664 1.654 1.645 1.636 1.626 1.620 1.612 1.594 1.263 1.238 1.233 1.214 1.189 1.164 0.845 0.828 0.821 0.803 0.796
1H NMR (DCCl3, 300 MHz)
OMe
Bu
N
N
O
Ph
5k
2.02 2.22 3.17 2.33 1.00 2.90 3.28 2.55 2.66 3.71
f1 (ppm)

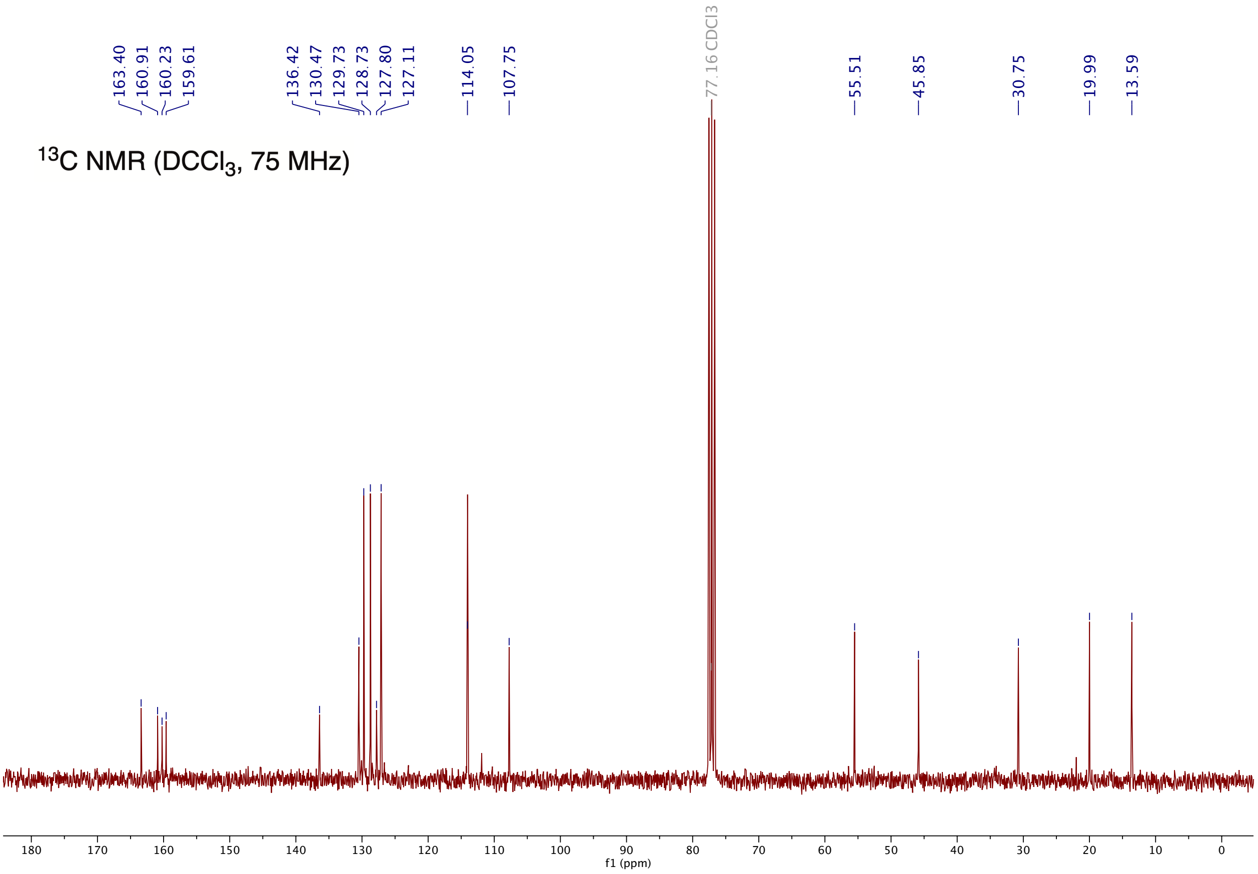
163.40 160.91 160.23 159.61 136.42 130.47 129.73 128.73 127.80 127.11 114.05 107.75 77.16 CDCl3 55.51 45.85 30.75 19.99 13.59
13C NMR (DCCl3, 75 MHz)
f1 (ppm)

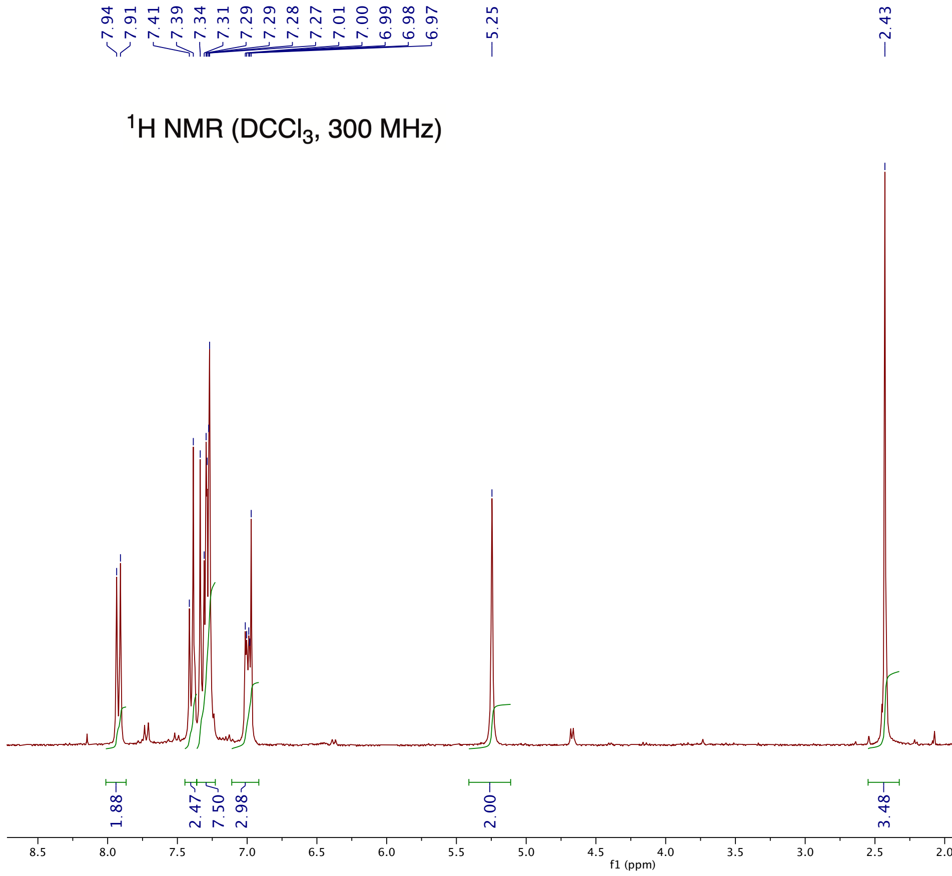
7.94
7.91
7.41
7.39
7.34
7.31
7.29
7.29
7.28
7.27
7.01
7.00
6.99
6.98
6.97
5.25
2.43
^{1}H NMR (DCCl$_3$, 300 MHz)
1.88
2.47
7.50
2.98
2.00
3.48
8.5 8.0 7.5 7.0 6.5 6.0 5.5 5.0 4.5 4.0 3.5 3.0 2.5 2.0 1.5 1.0 0.5 0.0
f1 (ppm)

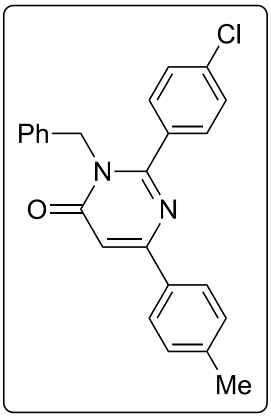
Cl
Ph
N
N
O
Me

5l

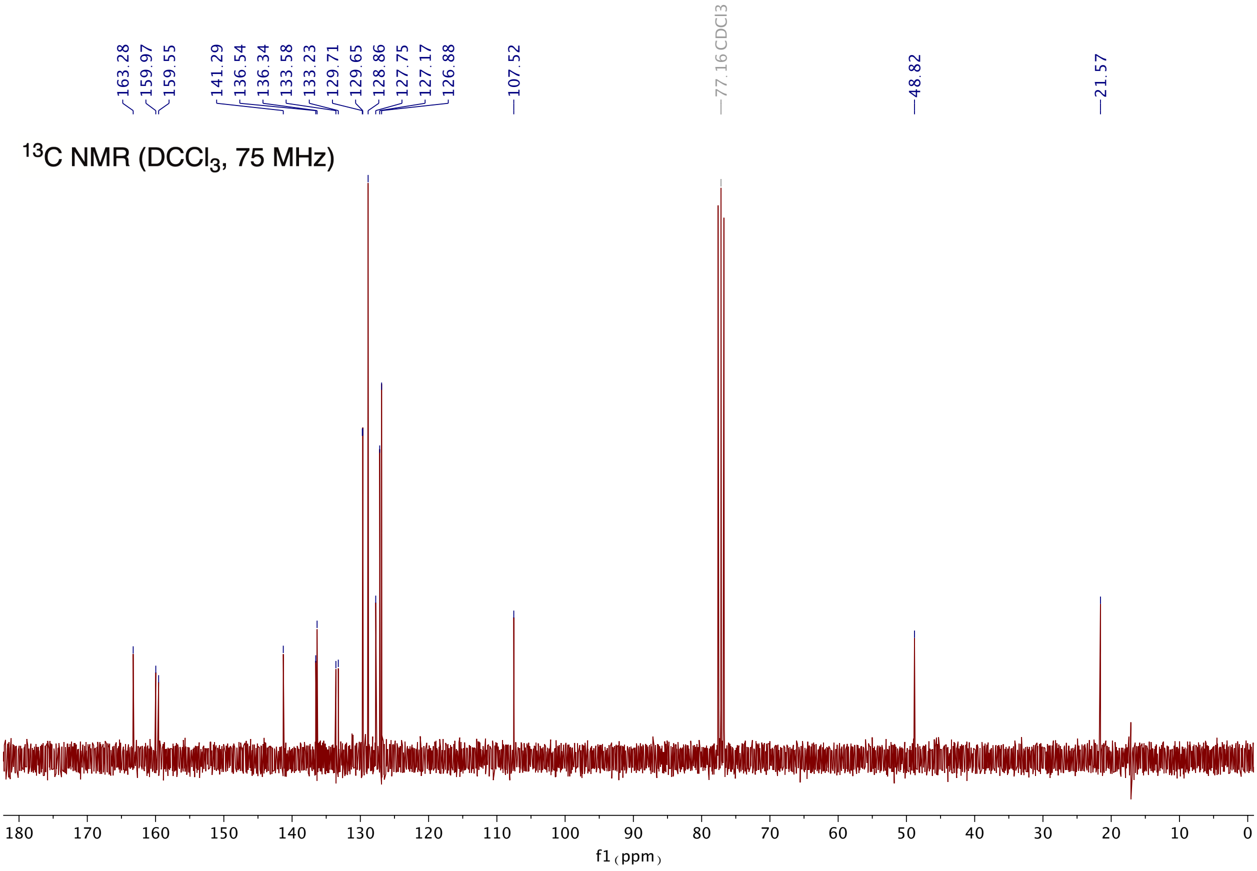
163.28
159.97
159.55
141.29
136.54
136.34
133.58
133.23
129.71
129.65
128.86
127.75
127.17
126.88
107.52
77.16 CDCl3
48.82
21.57
^{13}C NMR (DCCl$_3$, 75 MHz)
180 170 160 150 140 130 120 110 100 90 80 70 60 50 40 30 20 10 0
f1 (ppm)

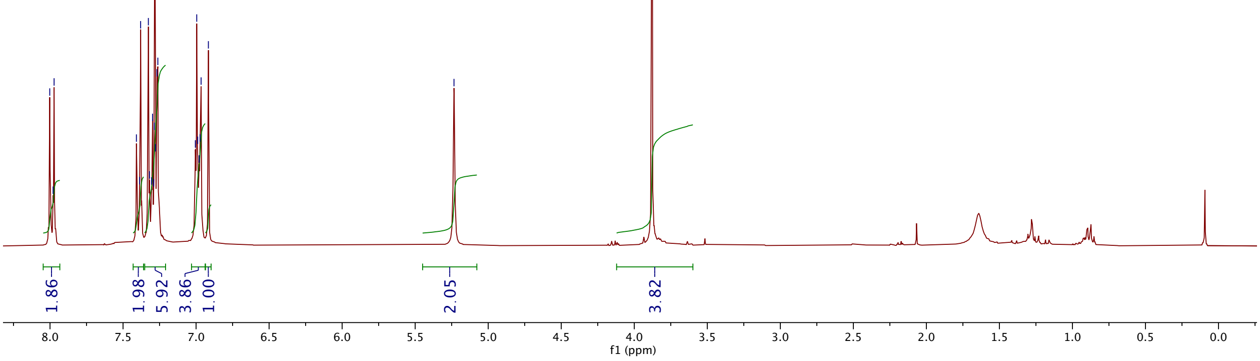
8.002
7.979
7.972
7.408
7.387
7.380
7.327
7.319
7.304
7.298
7.290
7.286
7.282
7.277
7.268
7.262
7.005
6.995
6.989
6.979
6.973
6.966
6.916
5.234
3.879
1H NMR (DCCl3, 300 MHz)
1.86
1.98
5.92
3.86
1.00
2.05
3.82
f1 (ppm)

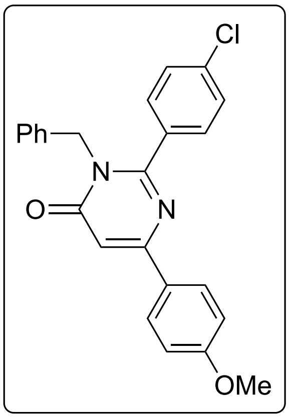
Cl
Ph
N
N
O
OMe

5m

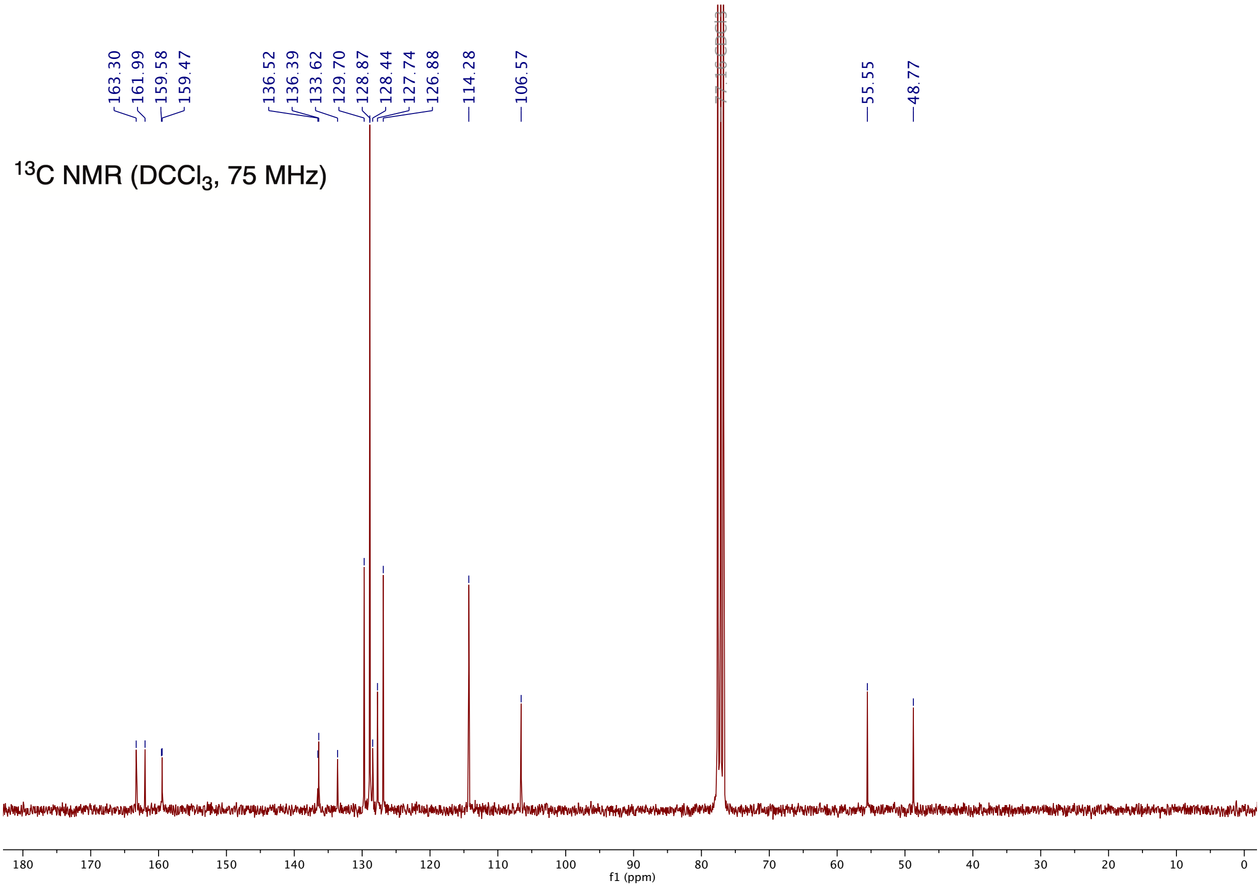
163.30
161.99
159.58
159.47
136.52
136.39
133.62
129.70
128.87
128.44
127.74
126.88
114.28
106.57
55.55
48.77
13C NMR (DCCl3, 75 MHz)
f1 (ppm)

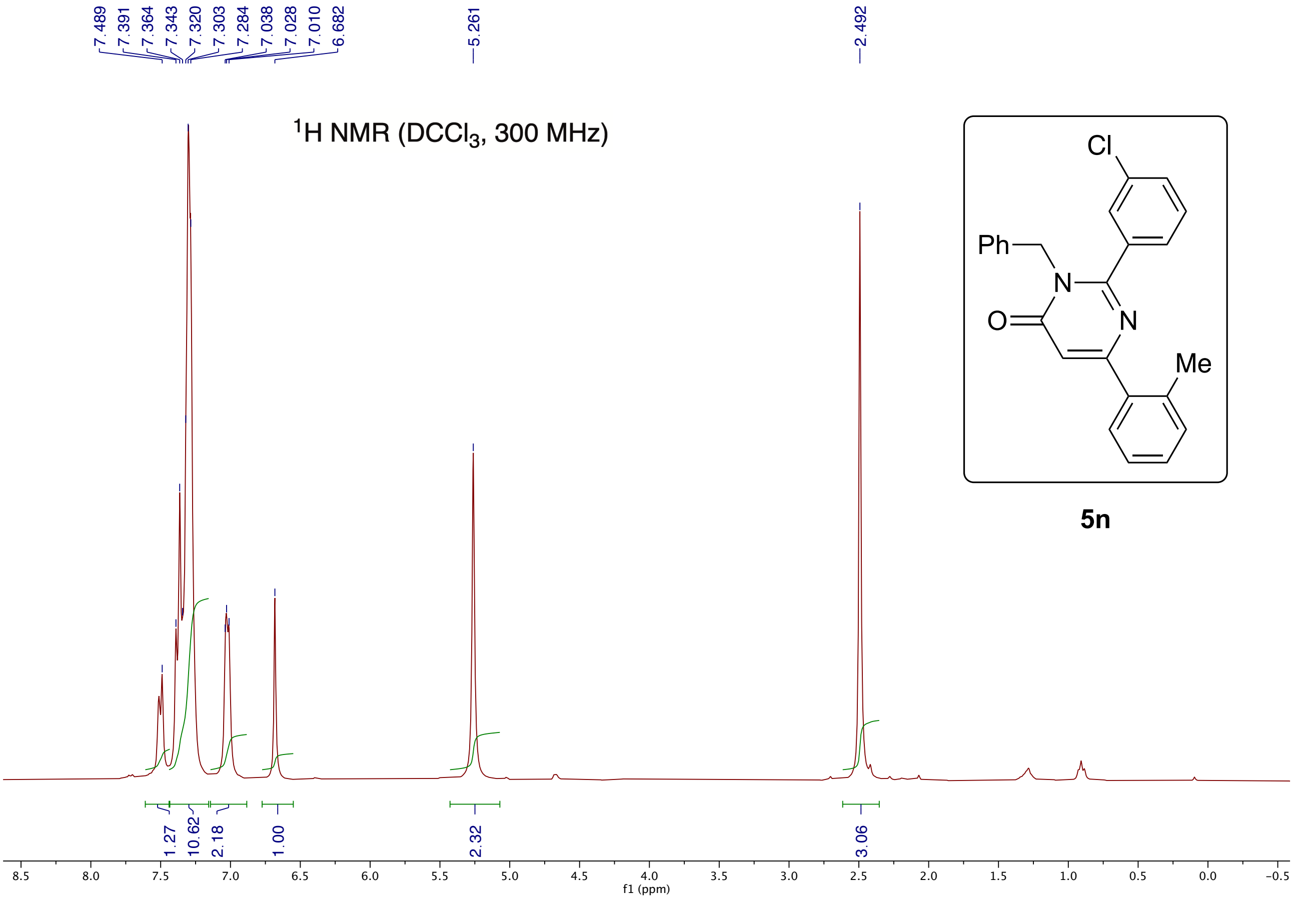
7.489
7.391
7.364
7.343
7.320
7.303
7.284
7.038
7.028
7.010
6.682
5.261
2.492
^{1}H NMR ($DCCl_3$, 300 MHz)
1.27
10.62
2.18
1.00
2.32
3.06
f1 (ppm)
Cl
Ph
N
N
O
Me

5n

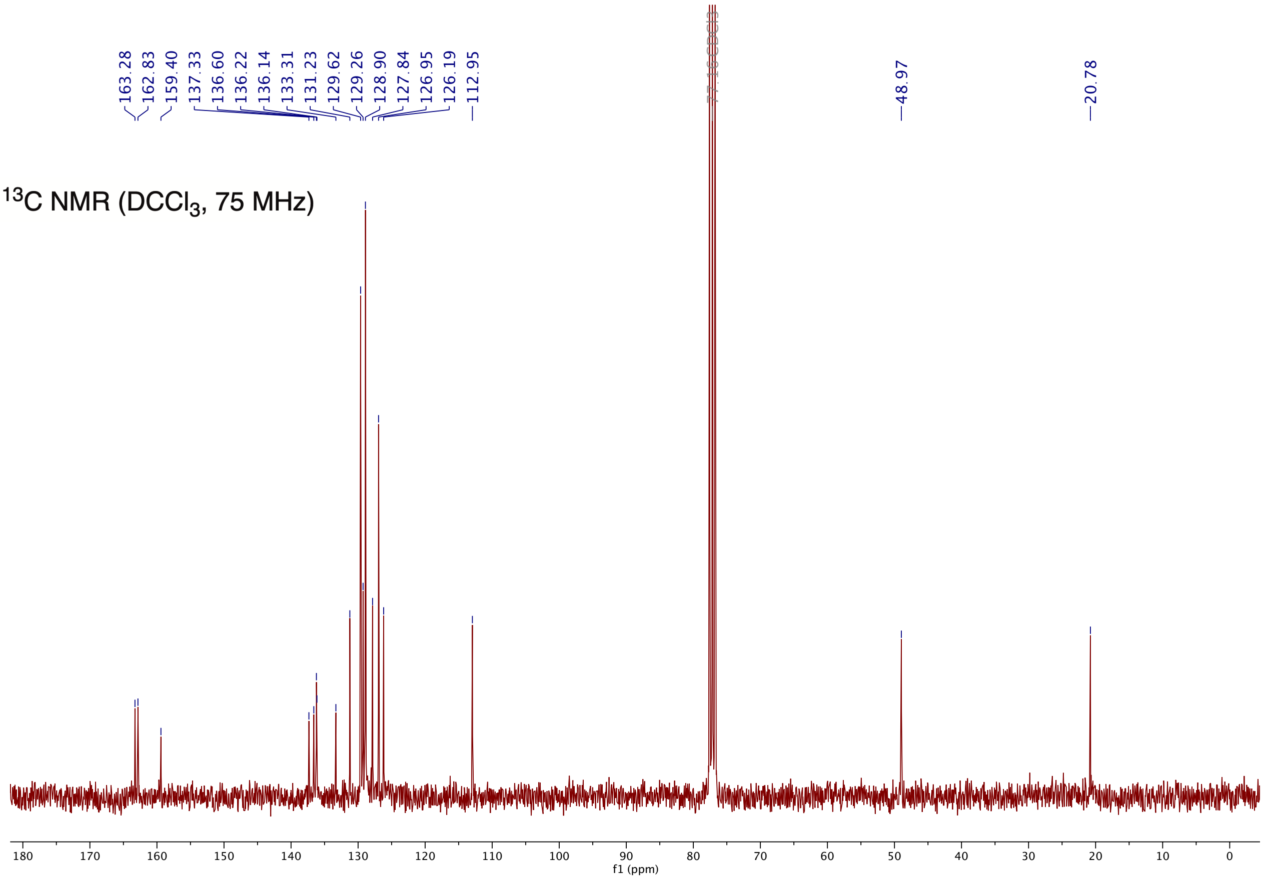
163.28
162.83
159.40
137.33
136.60
136.22
136.14
133.31
131.23
129.62
129.26
128.90
127.84
126.95
126.19
112.95
48.97
20.78
^{13}C NMR ($DCCl_3$, 75 MHz)
f1 (ppm)

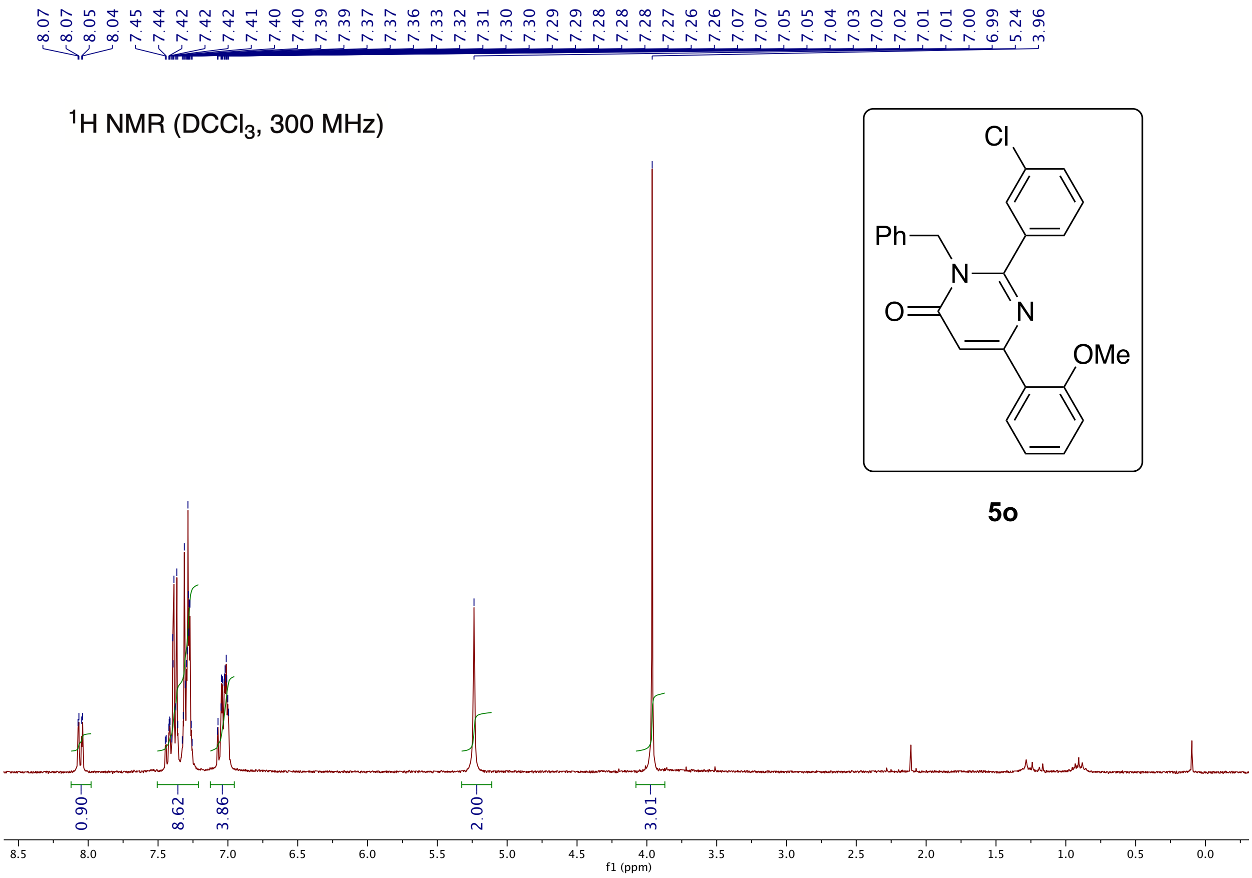
8.07 8.07 8.05 8.04 7.45 7.44 7.42 7.42 7.42 7.41 7.40 7.40 7.39 7.39 7.37 7.37 7.36 7.33 7.32 7.31 7.30 7.30 7.29 7.29 7.28 7.28 7.28 7.27 7.26 7.26 7.07 7.07 7.05 7.05 7.04 7.03 7.02 7.02 7.01 7.01 7.00 6.99 5.24 3.96
1H NMR (DCCl3, 300 MHz)
Cl
Ph
N
N
O
OMe
5o
0.90 8.62 3.86 2.00 3.01
f1 (ppm)

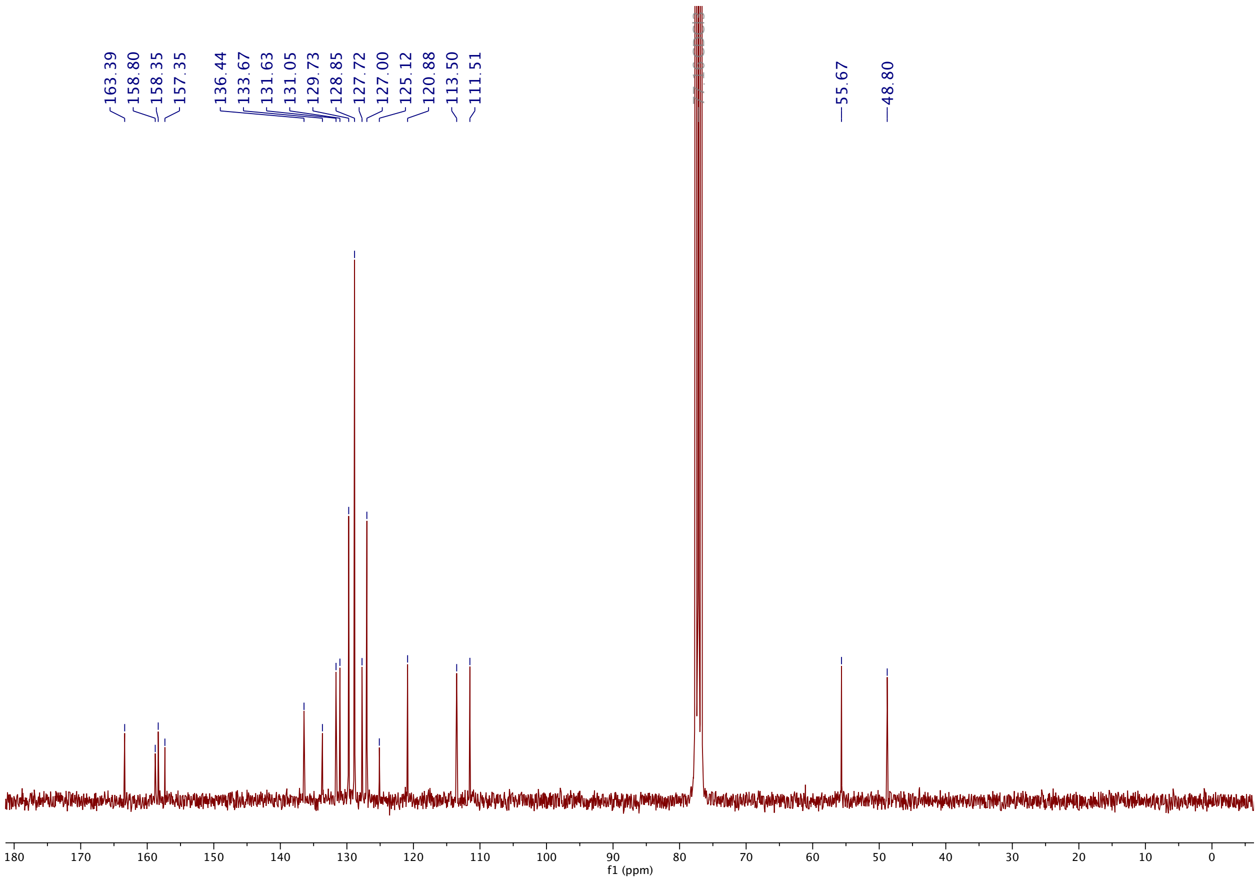
163.39 158.80 158.35 157.35 136.44 133.67 131.63 131.05 129.73 128.85 127.72 127.00 125.12 120.88 113.50 111.51 55.67 48.80
f1 (ppm)

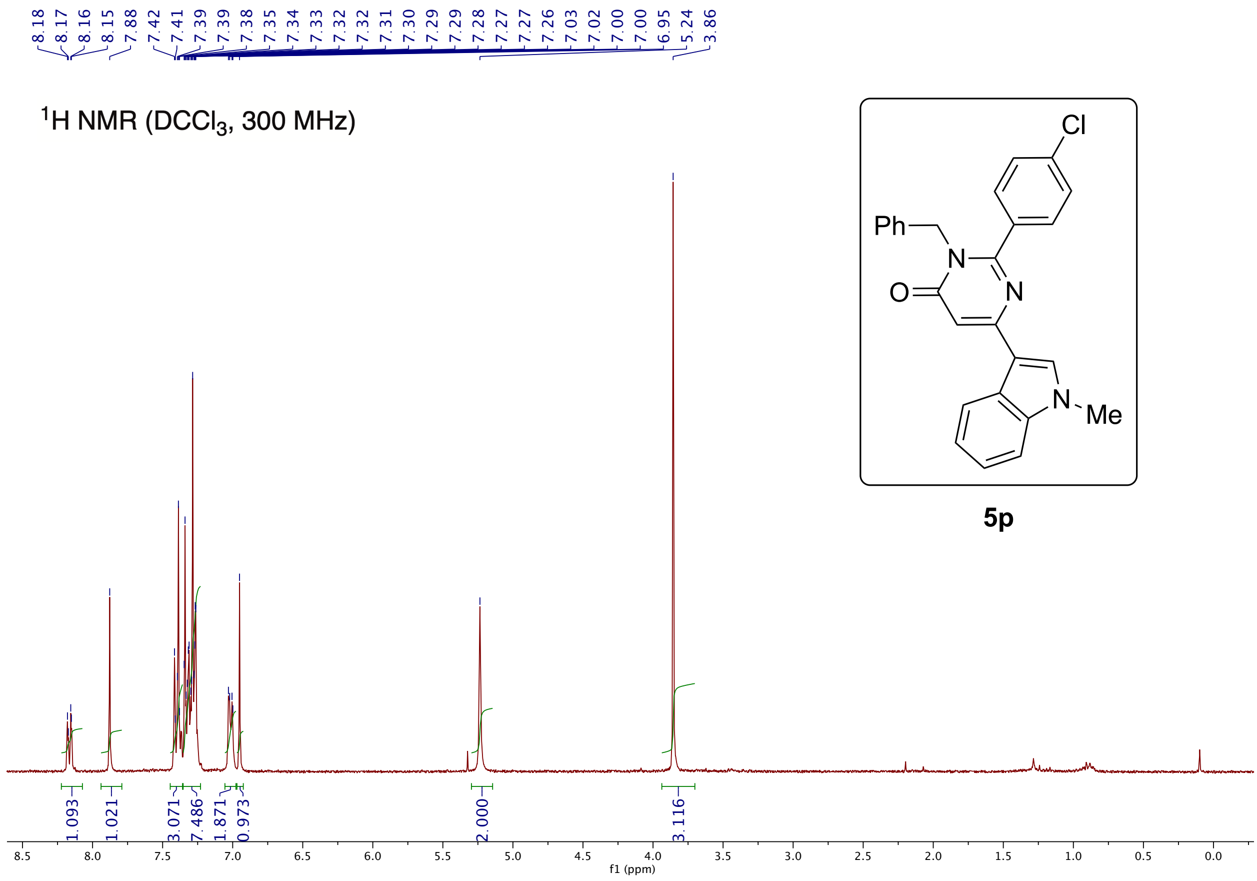

8.18
8.17
8.16
8.15
7.88
7.42
7.41
7.39
7.39
7.38
7.35
7.34
7.33
7.32
7.32
7.31
7.30
7.29
7.29
7.28
7.27
7.27
7.26
7.03
7.02
7.00
7.00
6.95
5.24
3.86
^{1}H NMR ($DCCl_3$, 300 MHz)
1.093
1.021
3.071
7.486
1.871
0.973
2.000
3.116
f1 (ppm)
Cl
Ph
N
N
O
N
Me

5p

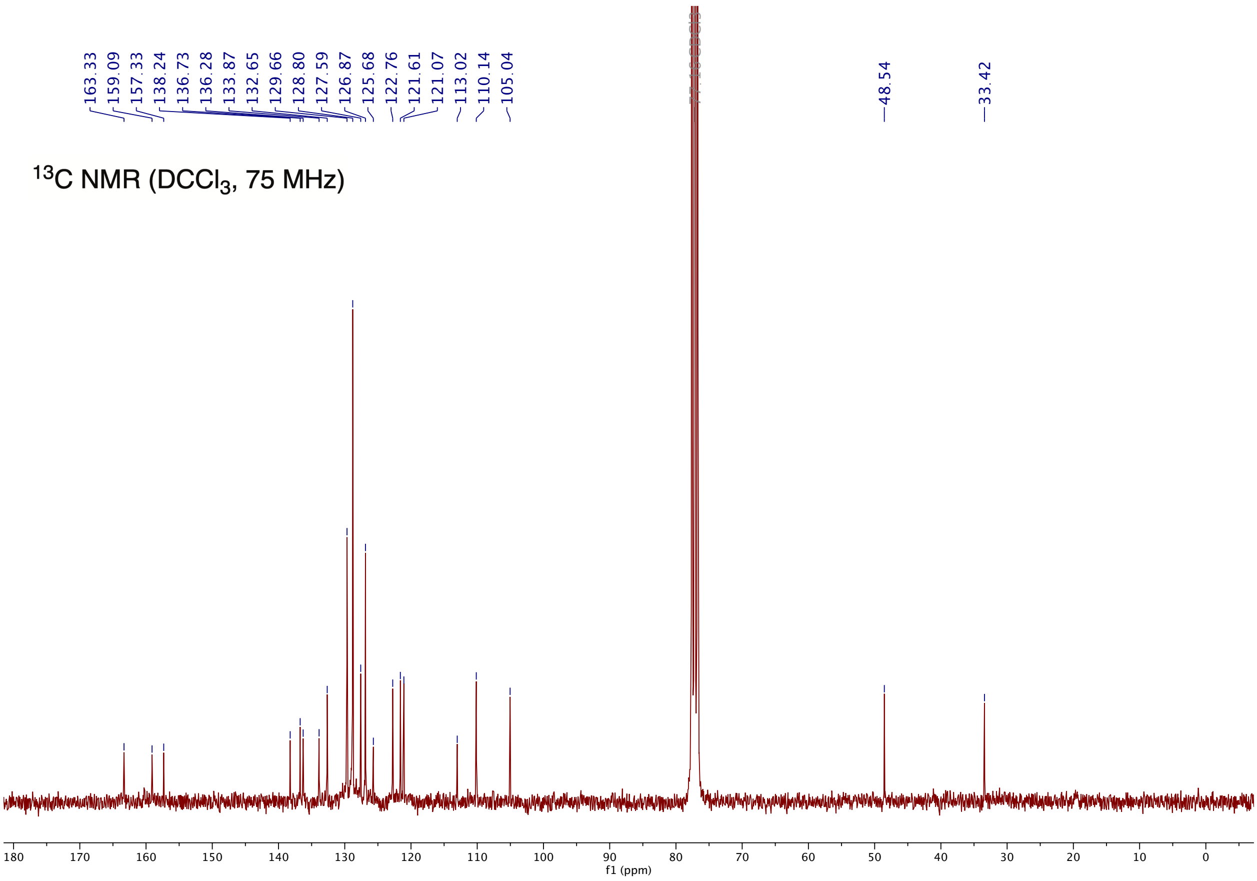

163.33
159.09
157.33
138.24
136.73
136.28
133.87
132.65
129.66
128.80
127.59
126.87
125.68
122.76
121.61
121.07
113.02
110.14
105.04
48.54
33.42
^{13}C NMR ($DCCl_3$, 75 MHz)
f1 (ppm)

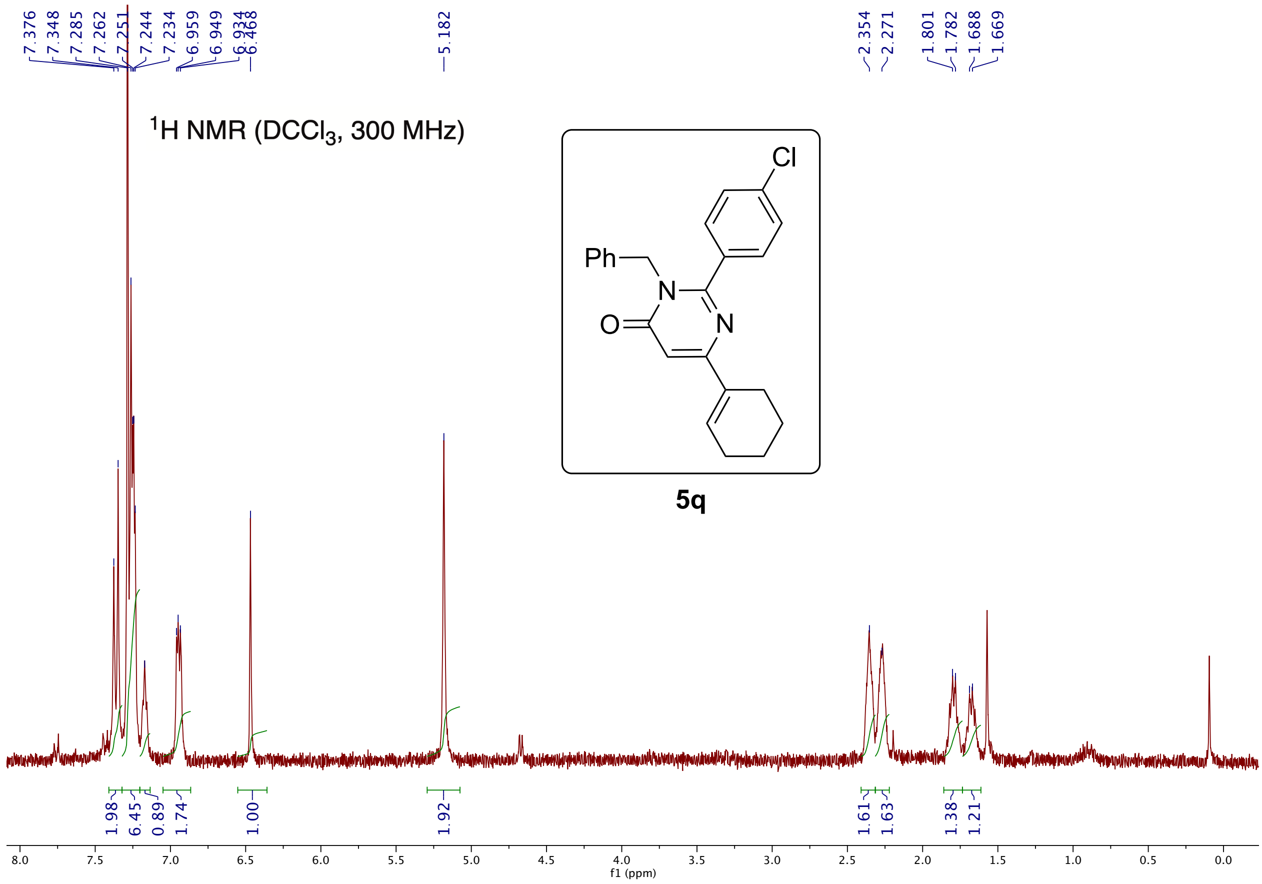
7.376
7.348
7.285
7.262
7.251
7.244
7.234
6.959
6.949
6.934
6.468
5.182
2.354
2.271
1.801
1.782
1.688
1.669
^{1}H NMR (DCCl$_3$, 300 MHz)
Cl
Ph
N
N
O
5q
1.98
6.45
0.89
1.74
1.00
1.92
1.61
1.63
1.38
1.21
f1 (ppm)

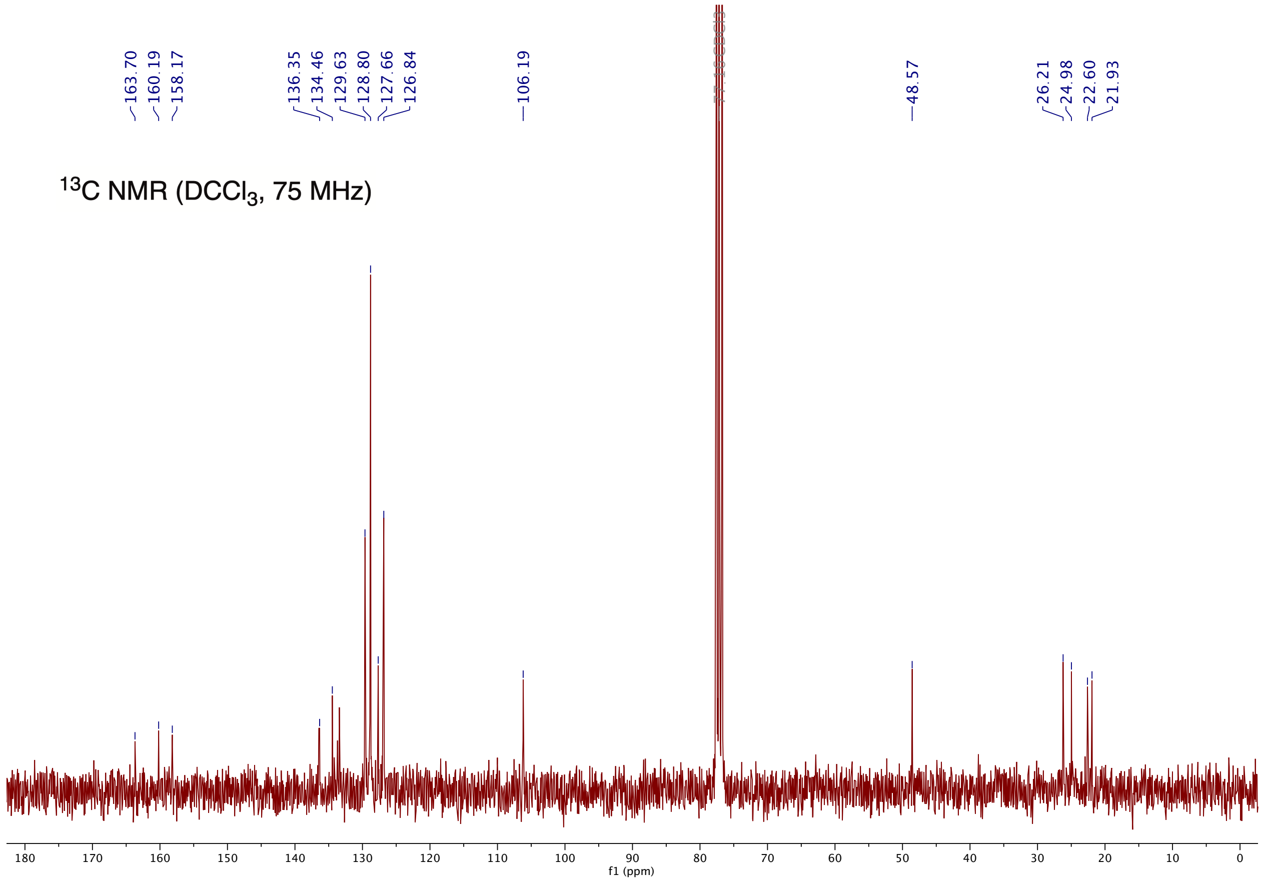
163.70
160.19
158.17
136.35
134.46
129.63
128.80
127.66
126.84
106.19
48.57
26.21
24.98
22.60
21.93
^{13}C NMR (DCCl$_3$, 75 MHz)
f1 (ppm)

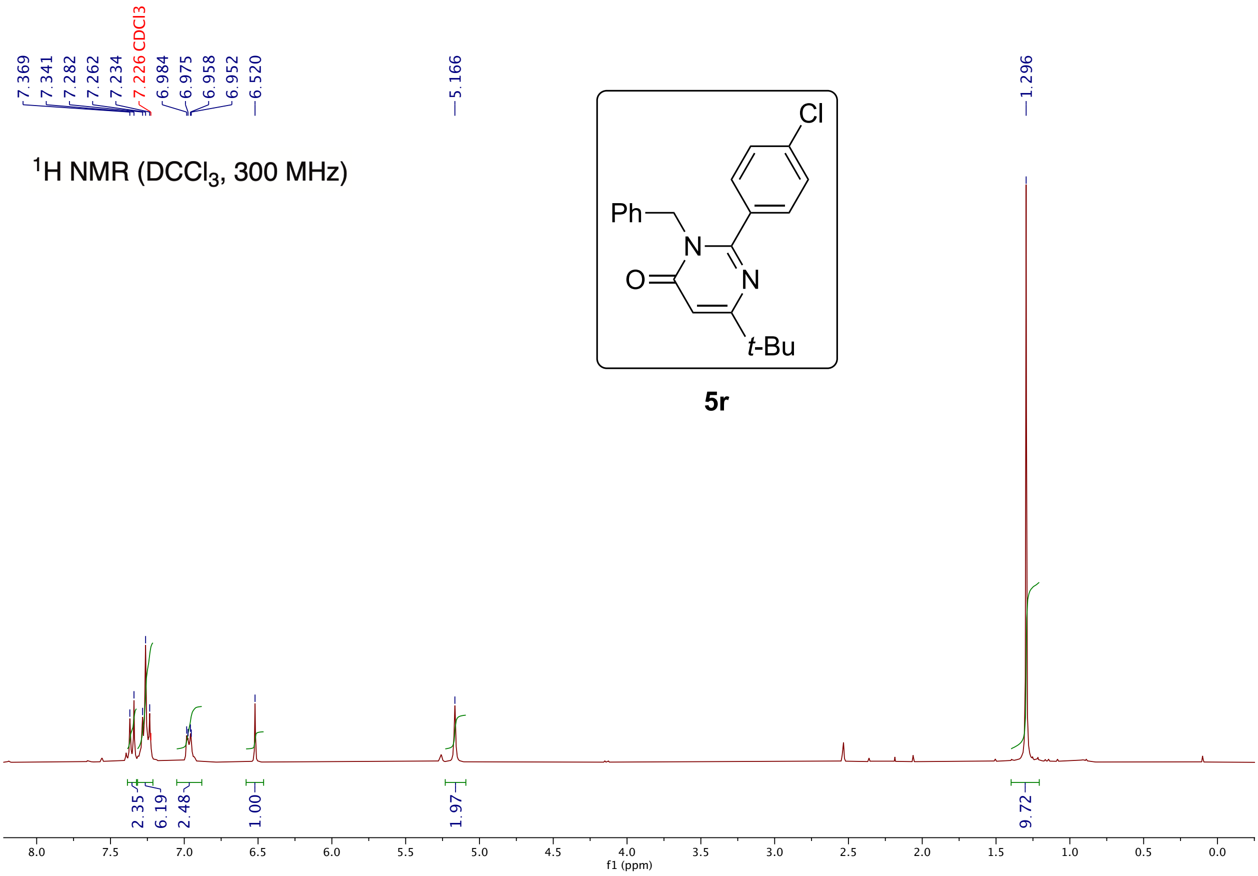
7.369
7.341
7.282
7.262
7.234
7.226 CDCl3
6.984
6.975
6.958
6.952
6.520
5.166
1.296
1H NMR (DCCl3, 300 MHz)
2.35
6.19
2.48
1.00
1.97
9.72
f1 (ppm)
Cl
Ph
N
N
O
t-Bu

5r

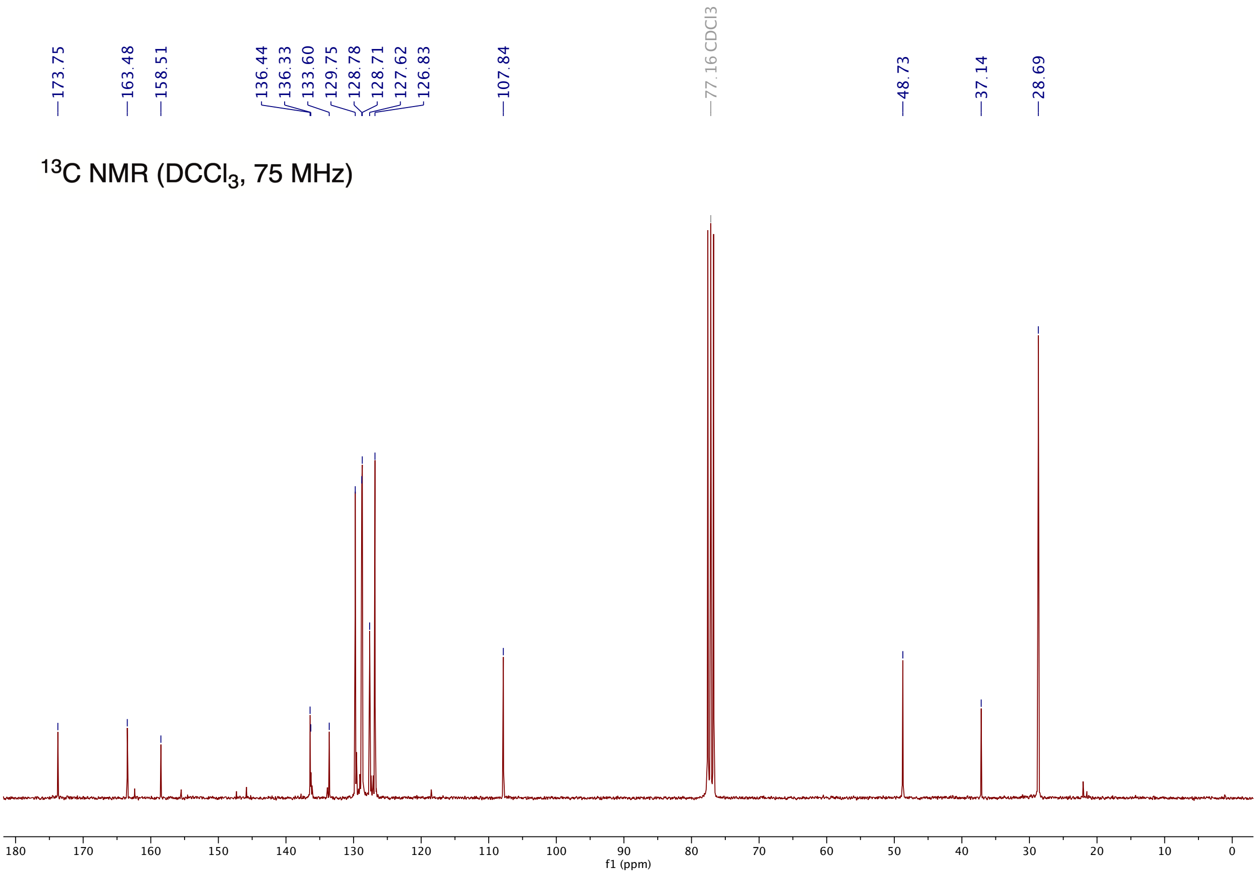
173.75
163.48
158.51
136.44
136.33
133.60
129.75
128.78
128.71
127.62
126.83
107.84
77.16 CDCl3
48.73
37.14
28.69
13C NMR (DCCl3, 75 MHz)
f1 (ppm)

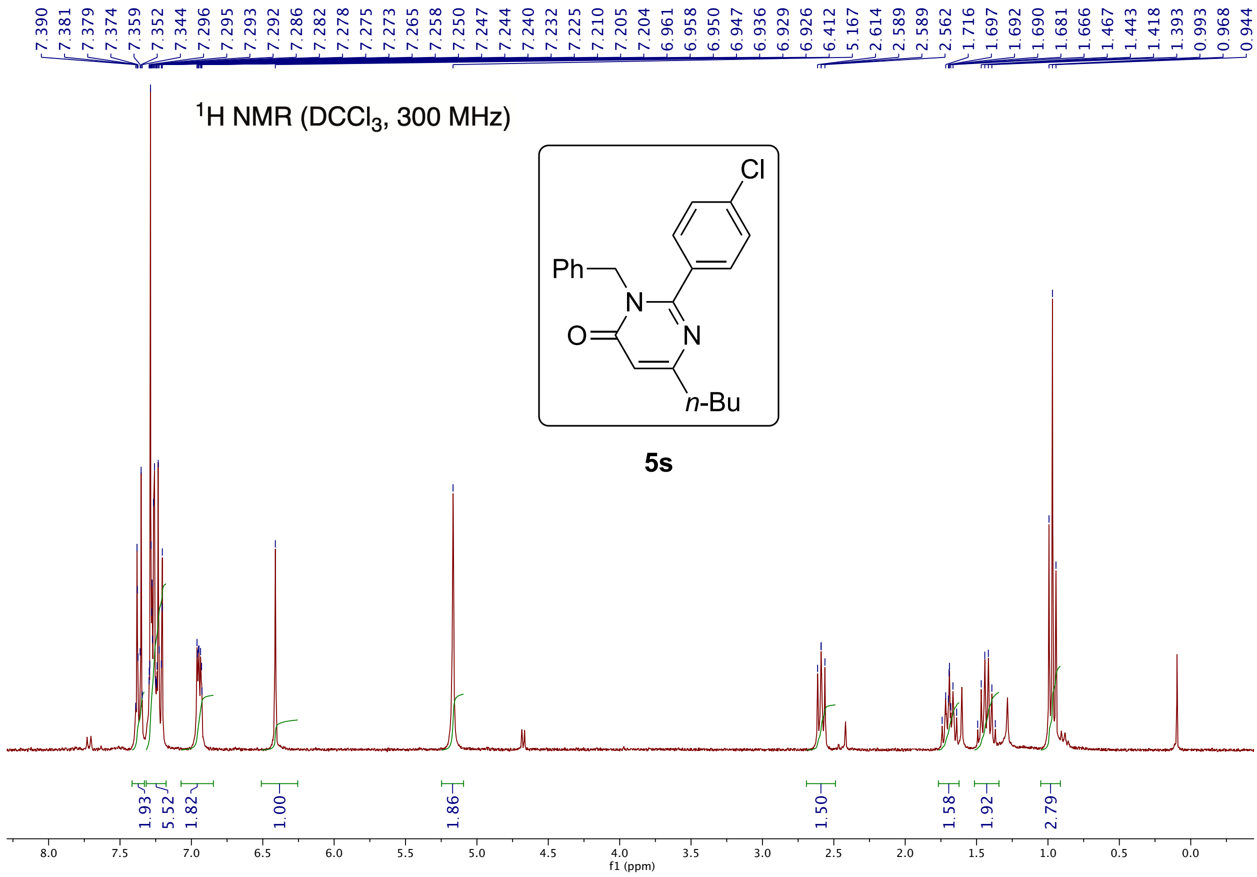
7.390 7.381 7.379 7.374 7.359 7.352 7.344 7.296 7.295 7.293 7.292 7.286 7.282 7.278 7.275 7.273 7.265 7.258 7.250 7.247 7.244 7.240 7.232 7.225 7.210 7.205 7.204 6.961 6.958 6.950 6.947 6.936 6.929 6.926 6.412 5.167 2.614 2.589 2.589 2.562 1.716 1.697 1.692 1.690 1.681 1.666 1.467 1.443 1.418 1.393 0.993 0.968 0.944
1H NMR (DCCl3, 300 MHz)
Cl
Ph
N
N
O
n-Bu
5s
1.93
5.52
1.82
1.00
1.86
1.50
1.58
1.92
2.79
f1 (ppm)

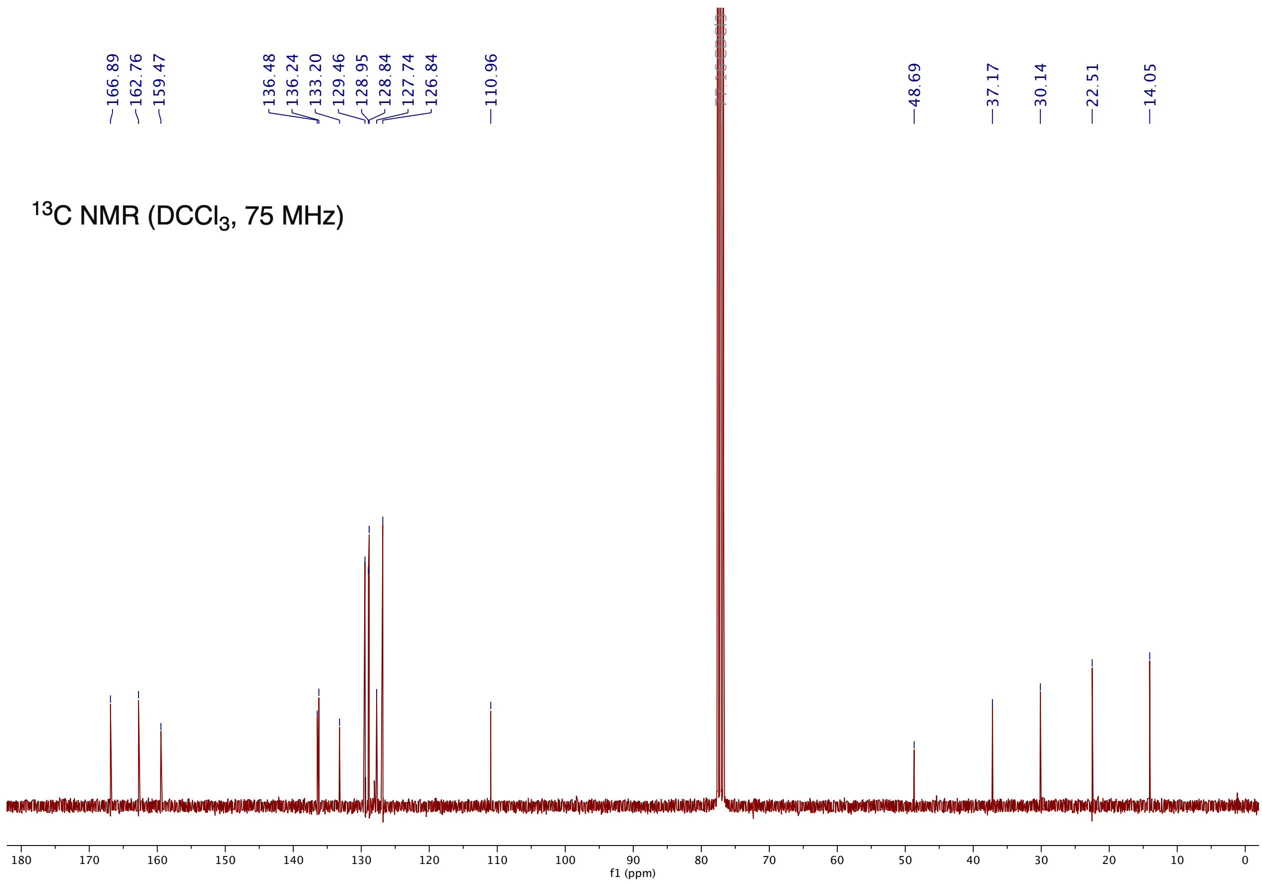
166.89 162.76 159.47
136.48 136.24 133.20 129.46 128.95 128.84 127.74 126.84
110.96
48.69
37.17
30.14
22.51
14.05
13C NMR (DCCl3, 75 MHz)
f1 (ppm)

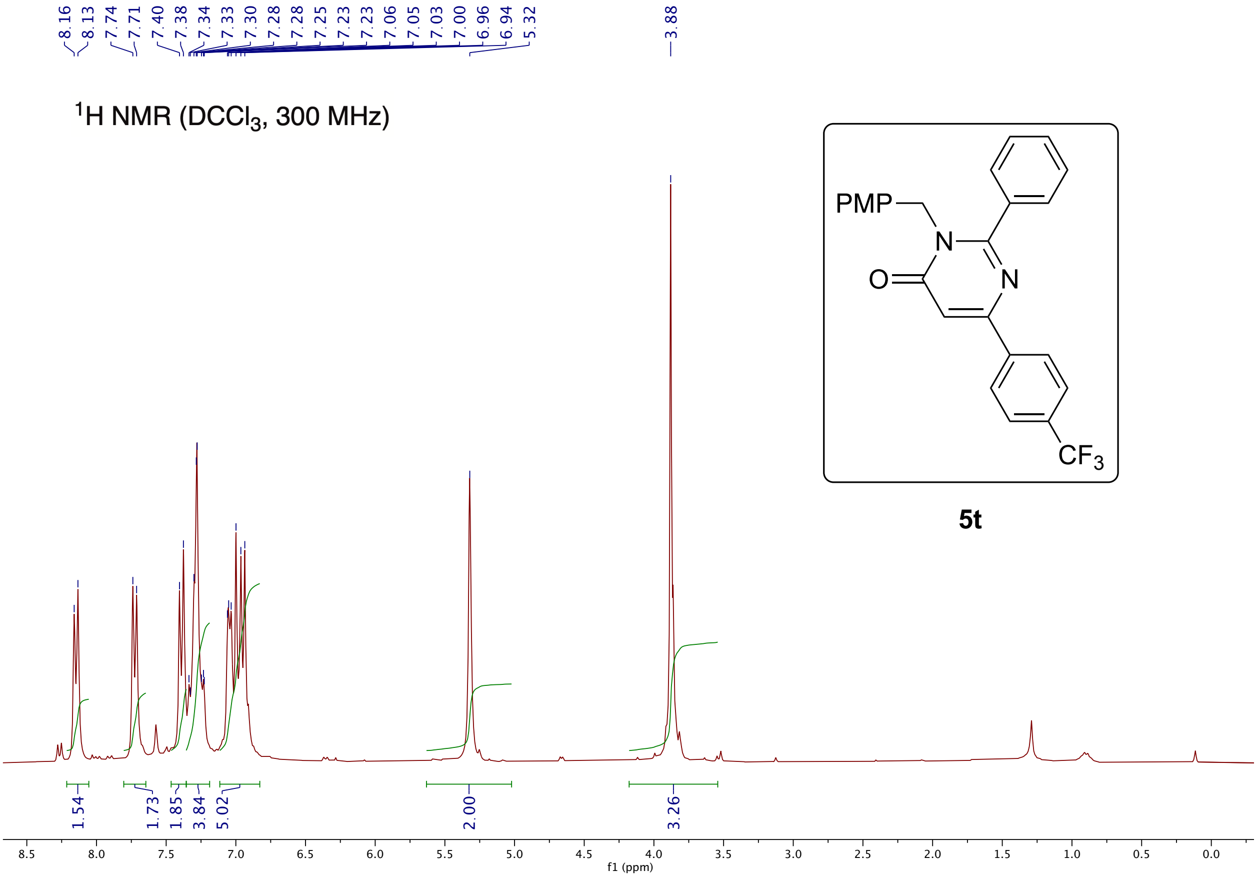
8.16
8.13
7.74
7.71
7.40
7.38
7.34
7.33
7.30
7.28
7.28
7.25
7.23
7.23
7.06
7.05
7.03
7.00
6.96
6.94
5.32
3.88
^{1}H NMR ($DCCl_3$, 300 MHz)
1.54
1.73
1.85
3.84
5.02
2.00
3.26
f1 (ppm)
PMP
N
N
O
CF_3

5t

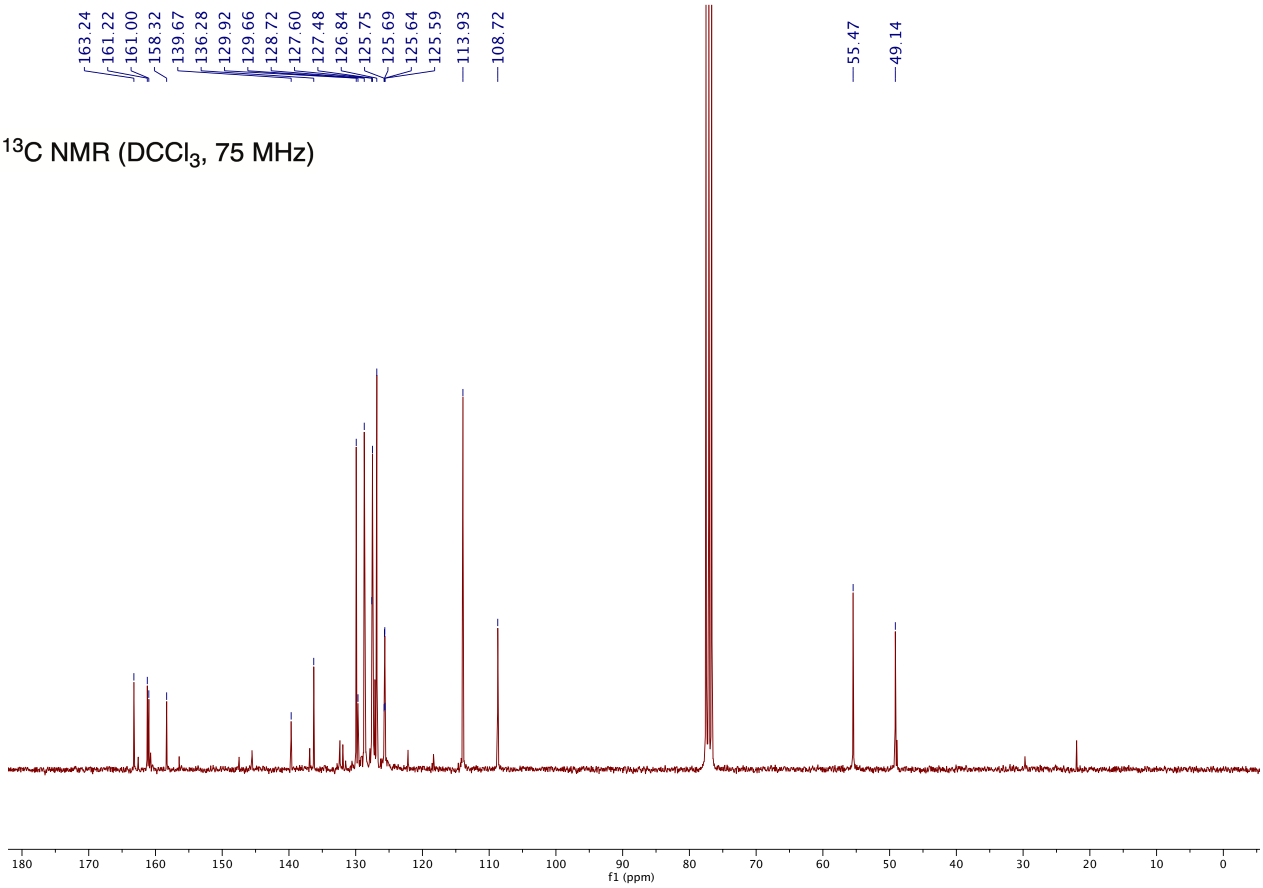
163.24
161.22
161.00
158.32
139.67
136.28
129.92
129.66
128.72
127.60
127.48
126.84
125.75
125.69
125.64
125.59
113.93
108.72
55.47
49.14
^{13}C NMR ($DCCl_3$, 75 MHz)
f1 (ppm)

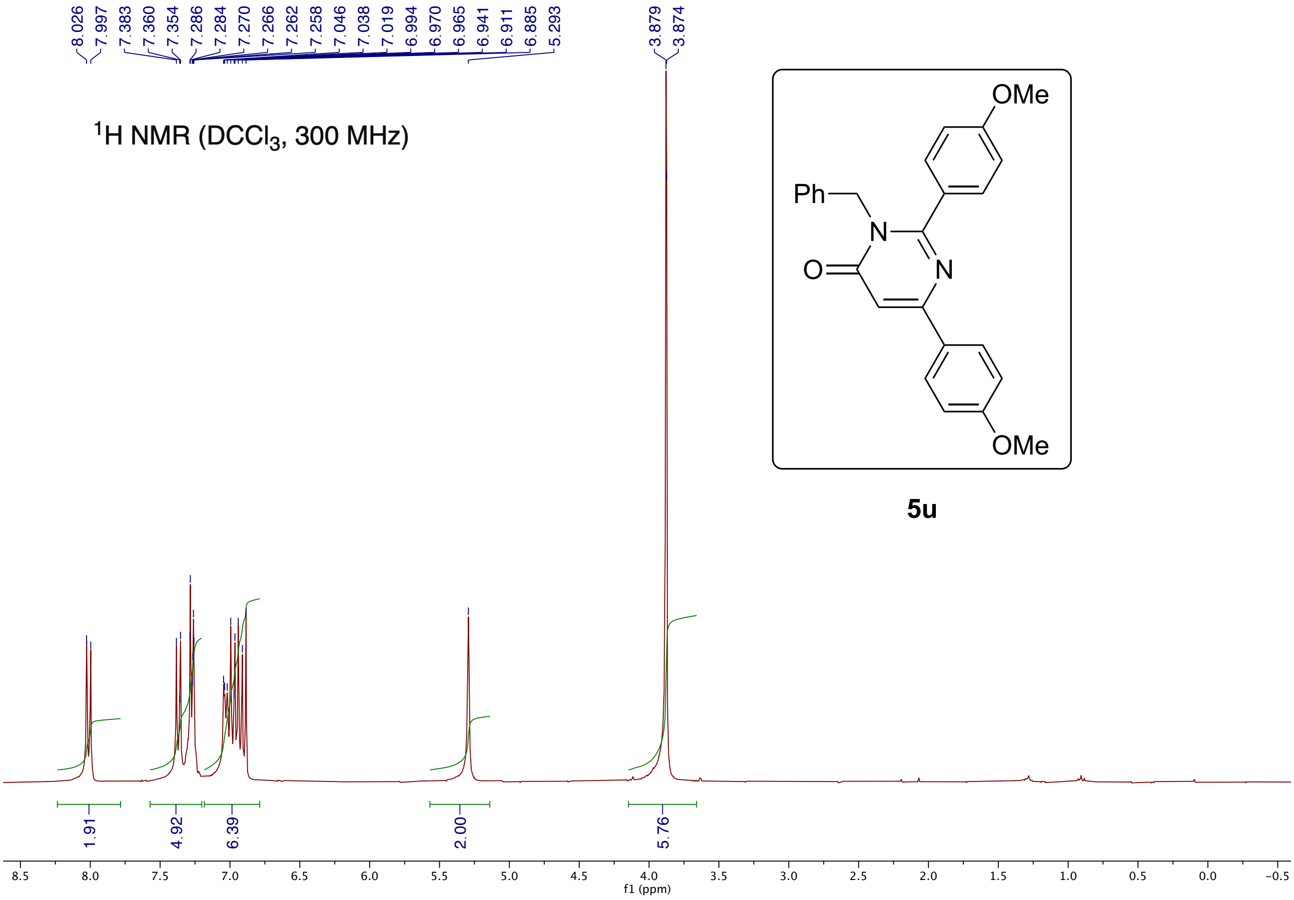

8.026
7.997
7.383
7.360
7.354
7.286
7.284
7.270
7.266
7.262
7.258
7.046
7.038
7.019
6.994
6.970
6.965
6.941
6.911
6.885
5.293
3.879
3.874
1H NMR (DCCl3, 300 MHz)
1.91
4.92
6.39
2.00
5.76
f1 (ppm)
OMe
Ph
N
O
N
OMe

5u

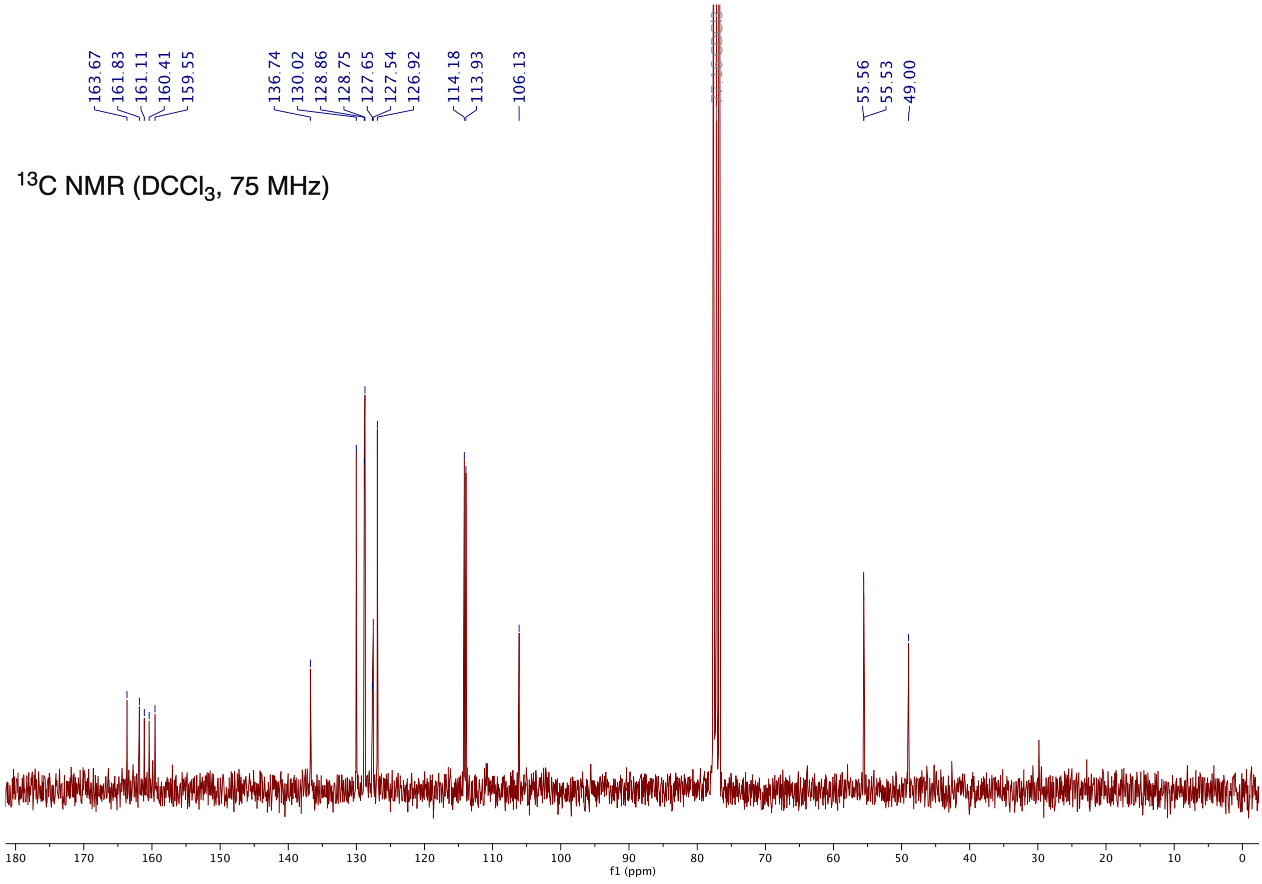

163.67
161.83
161.11
160.41
159.55
136.74
130.02
128.86
128.75
127.65
127.54
126.92
114.18
113.93
106.13
55.56
55.53
49.00
13C NMR (DCCl3, 75 MHz)
f1 (ppm)

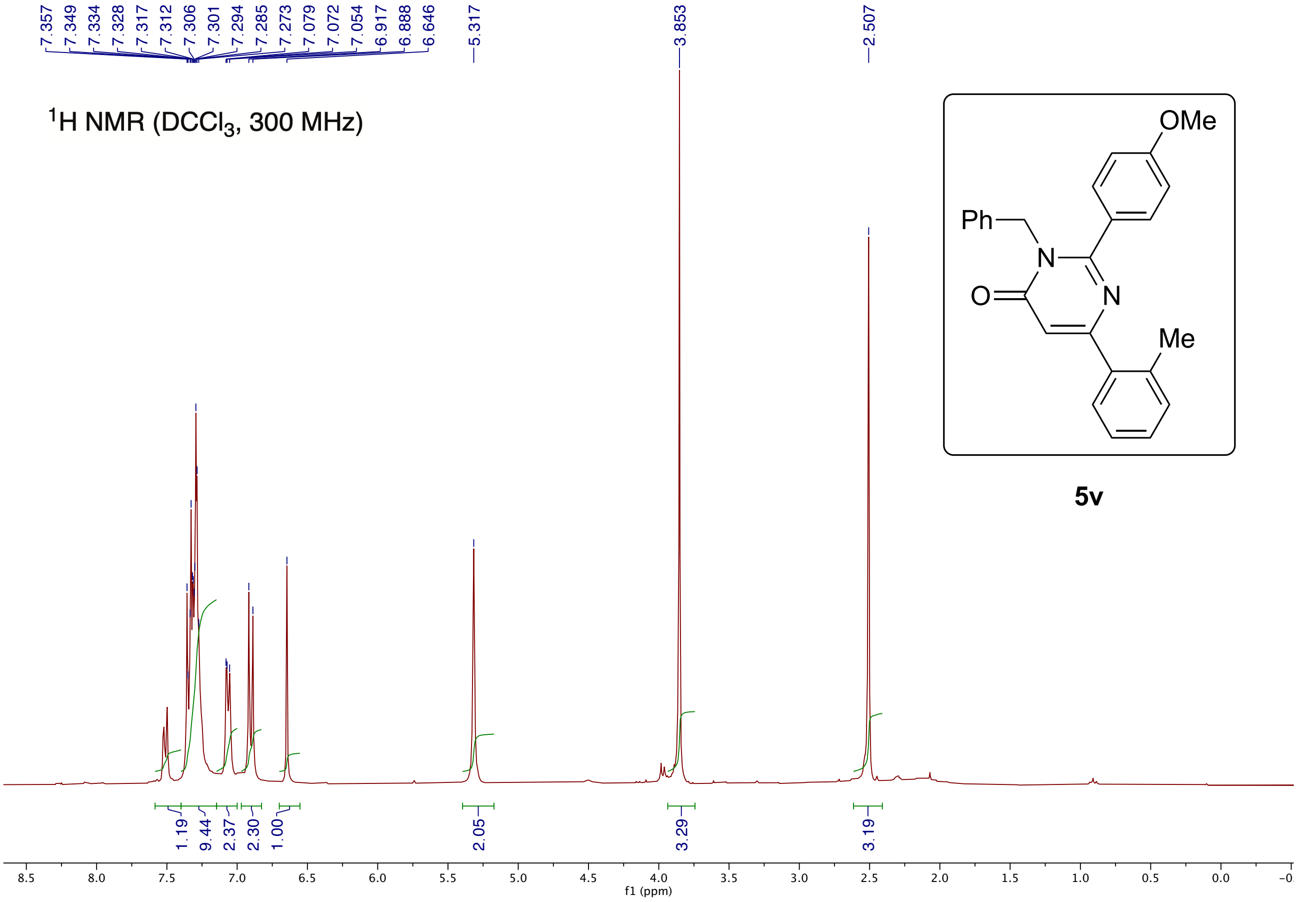
7.357
7.349
7.334
7.328
7.317
7.312
7.306
7.301
7.294
7.285
7.273
7.079
7.072
7.054
6.917
6.888
6.646
5.317
3.853
2.507
1H NMR (DCCl3, 300 MHz)
OMe
Ph
N
N
O
Me
1.19
9.44
2.37
2.30
1.00
2.05
3.29
3.19
f1 (ppm)

5v

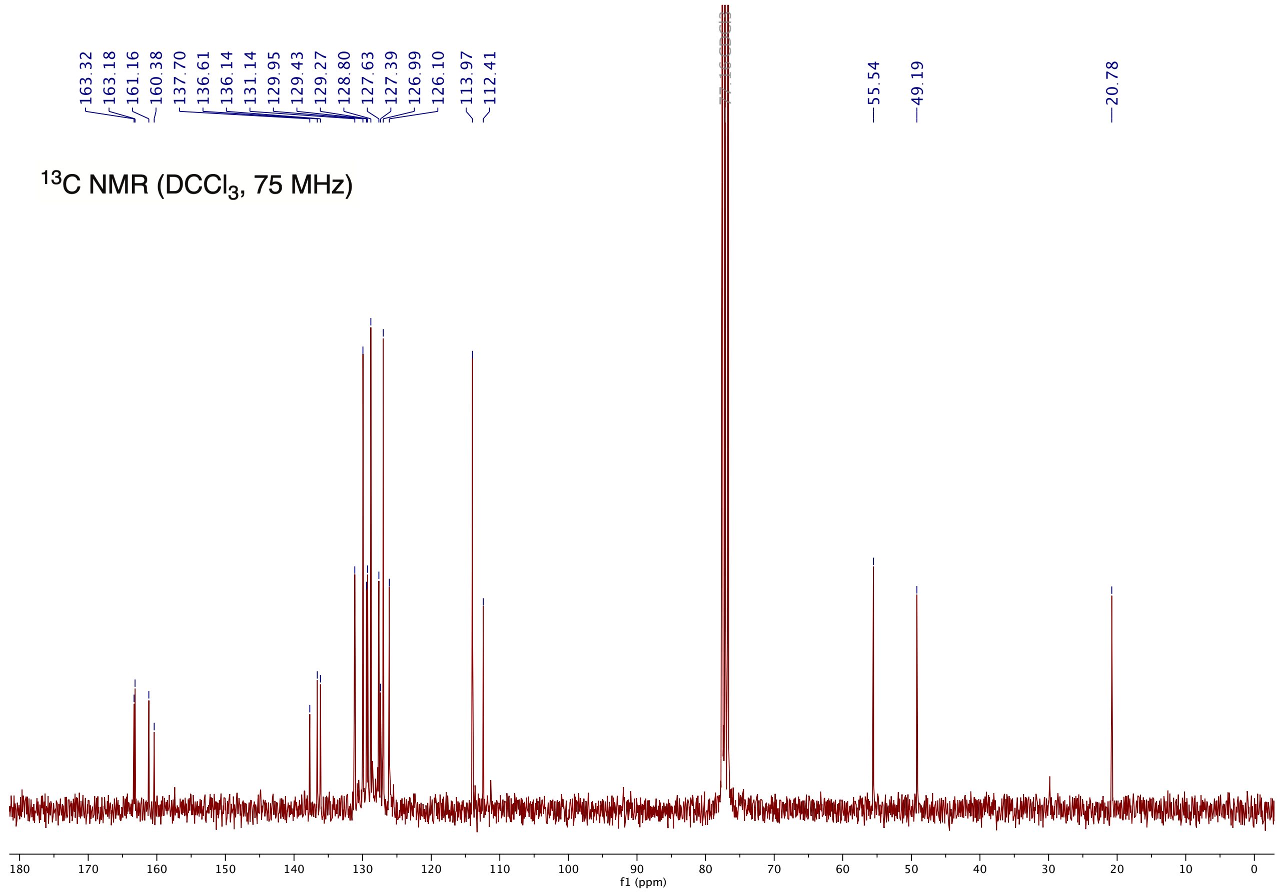
163.32
163.18
161.16
160.38
137.70
136.61
136.14
131.14
129.95
129.43
129.27
128.80
127.63
127.39
126.99
126.10
113.97
112.41
55.54
49.19
20.78
13C NMR (DCCl3, 75 MHz)
f1 (ppm)

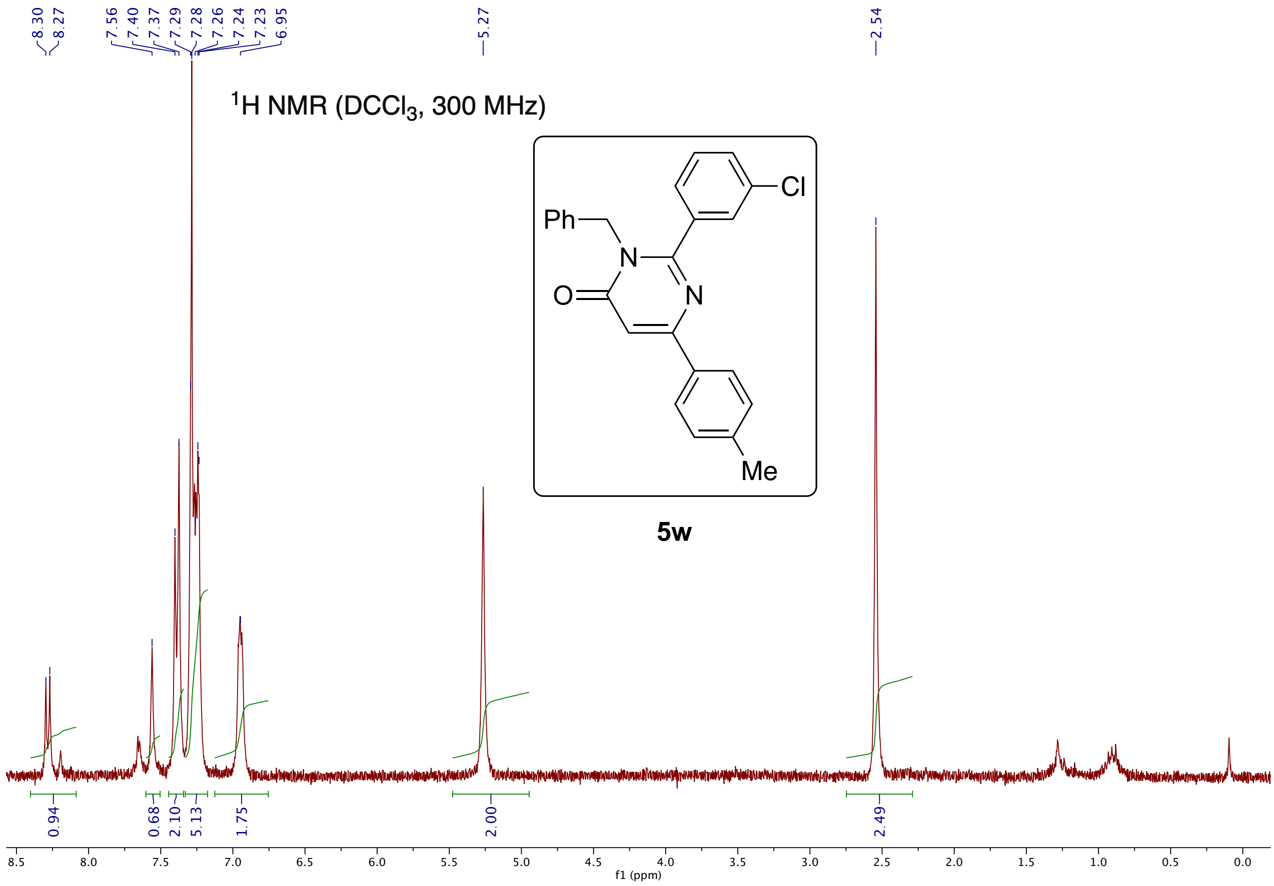
8.30
8.27
7.56
7.40
7.37
7.29
7.28
7.26
7.24
7.23
6.95
5.27
2.54
^{1}H NMR (DCCl$_3$, 300 MHz)
Ph
N
N
O
Cl
Me
0.94
0.68
2.10
5.13
1.75
2.00
2.49
f1 (ppm)

5w

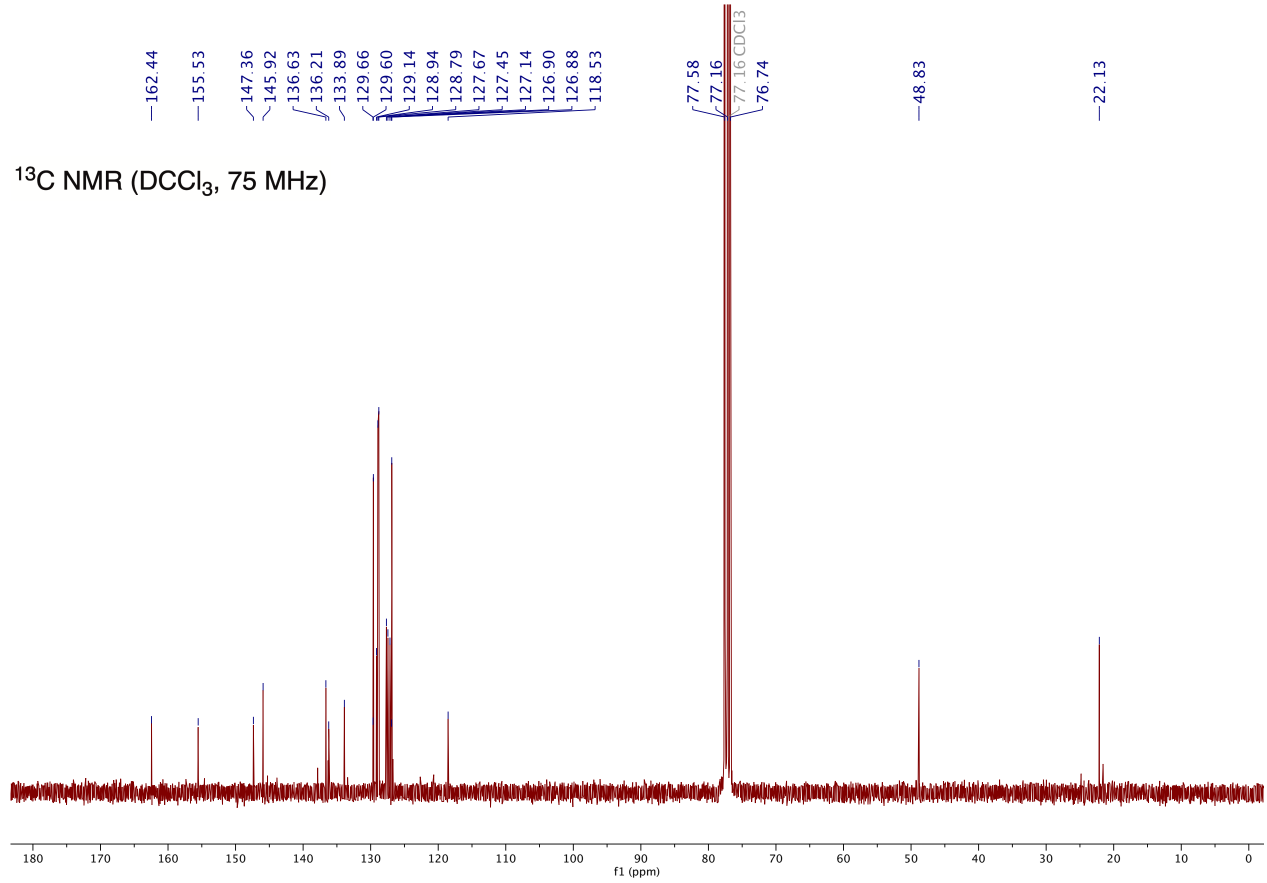
162.44
155.53
147.36
145.92
136.63
136.21
133.89
129.66
129.60
129.14
128.94
128.79
127.67
127.45
127.14
126.90
126.88
118.53
77.58
77.16
77.16 CDCl3
76.74
48.83
22.13
^{13}C NMR (DCCl$_3$, 75 MHz)
f1 (ppm)

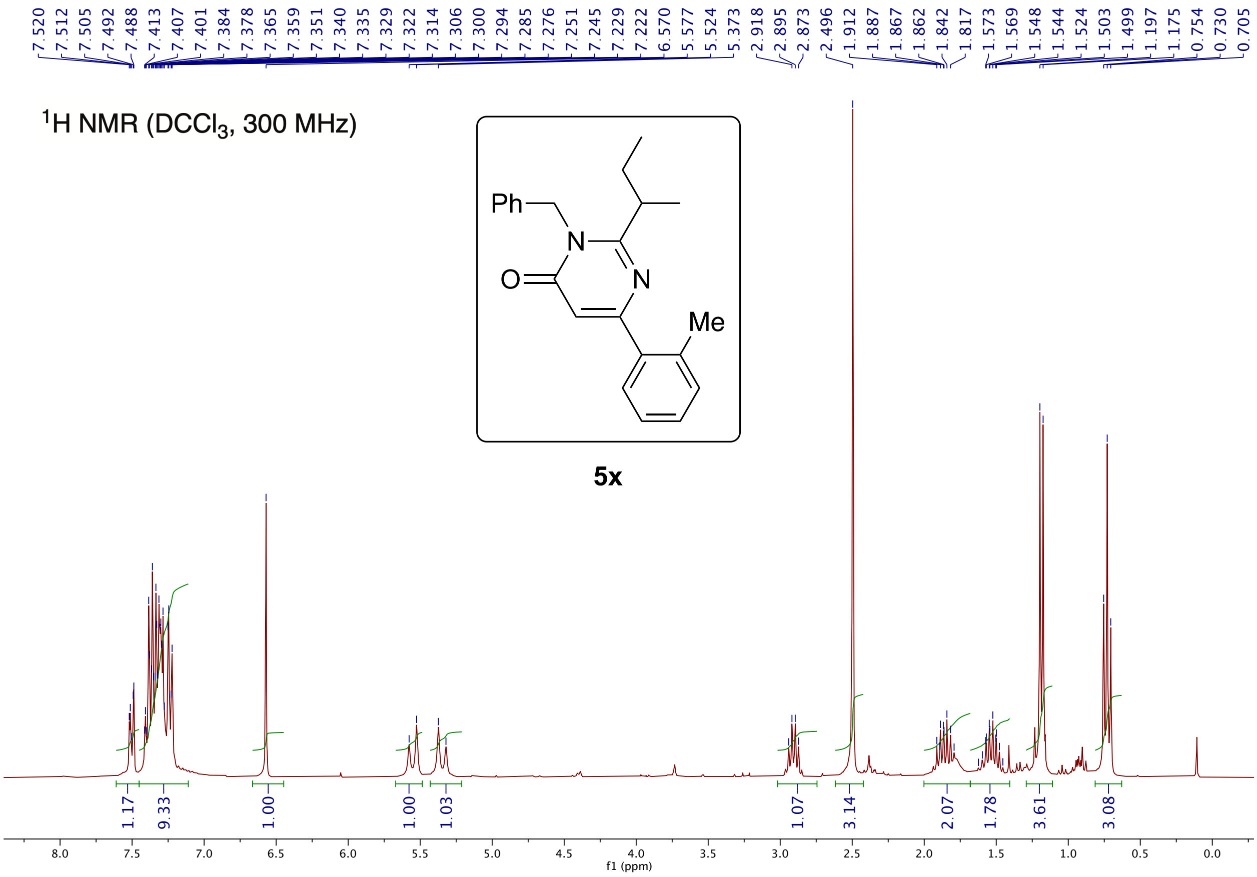

7.520 7.512 7.505 7.492 7.488 7.413 7.407 7.401 7.384 7.378 7.365 7.359 7.351 7.340 7.335 7.329 7.322 7.314 7.306 7.300 7.294 7.285 7.276 7.251 7.245 7.229 7.222 6.570 5.577 5.524 5.373 2.918 2.895 2.873 2.496 1.912 1.887 1.867 1.862 1.842 1.817 1.573 1.569 1.548 1.544 1.524 1.503 1.499 1.197 1.175 0.754 0.730 0.705
^{1}H NMR (DCCl$_3$, 300 MHz)
Ph
N
N
O
Me
5x
1.17 9.33 1.00 1.00 1.03 1.07 3.14 2.07 1.78 3.61 3.08
f1 (ppm)

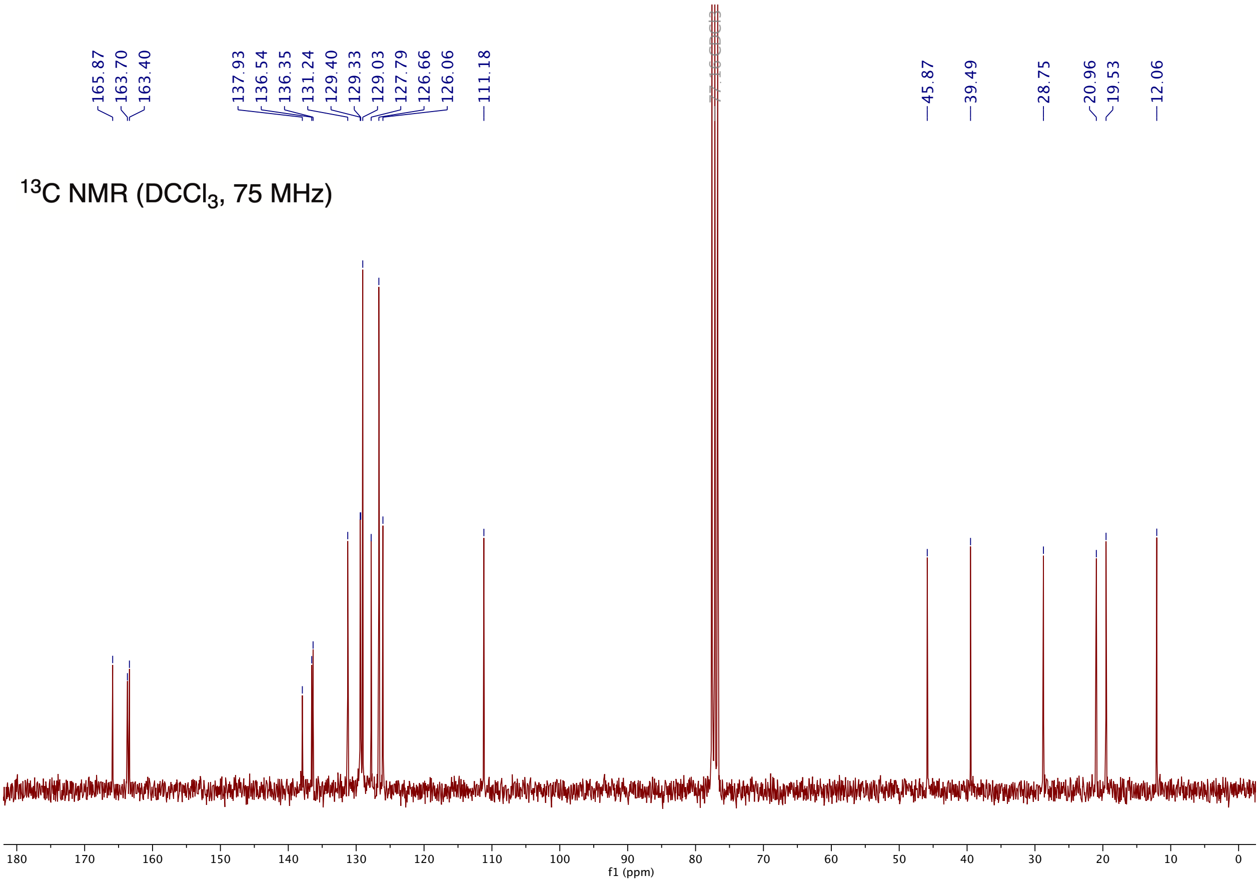

165.87 163.70 163.40 137.93 136.54 136.35 131.24 129.40 129.33 129.03 127.79 126.66 126.06 111.18 45.87 39.49 28.75 20.96 19.53 12.06
^{13}C NMR (DCCl$_3$, 75 MHz)
f1 (ppm)

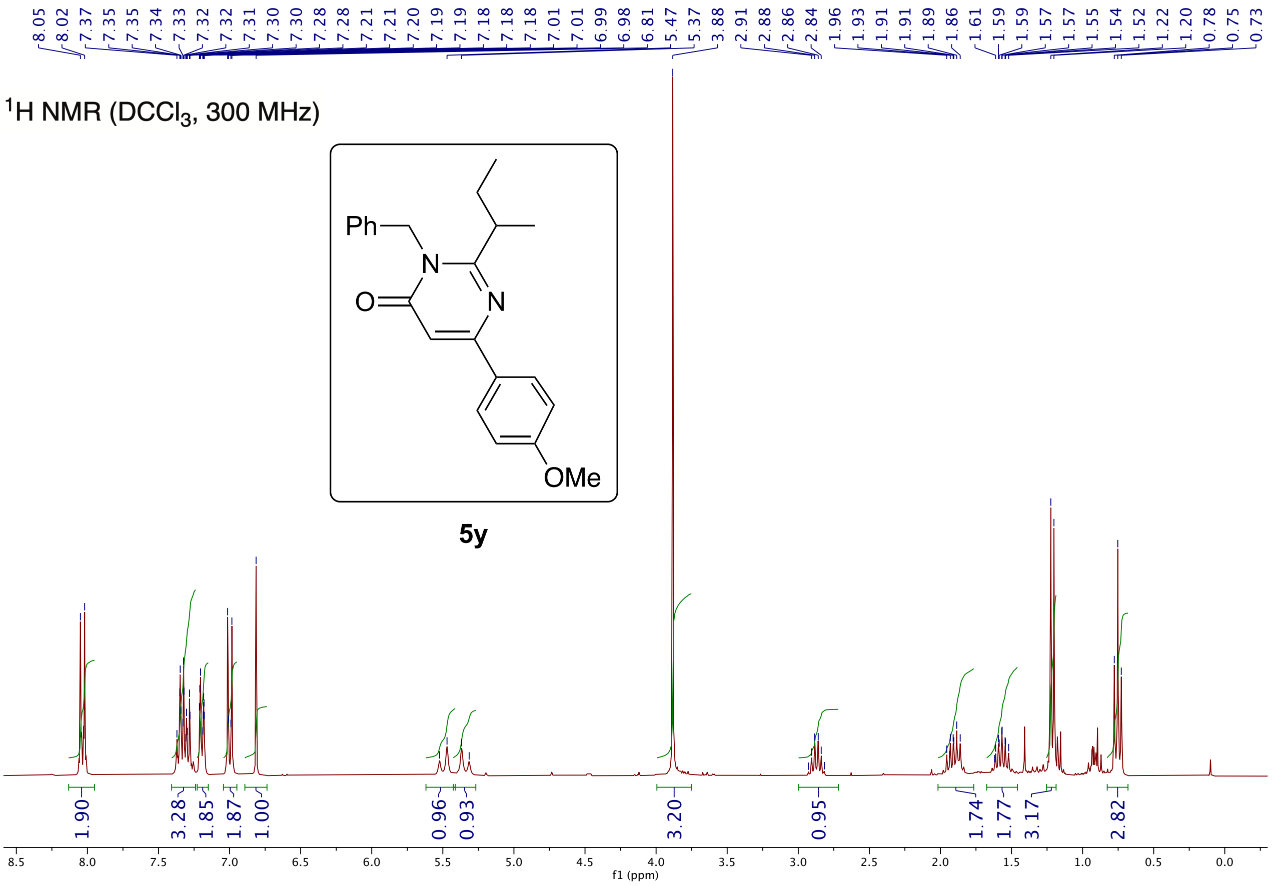
1H NMR (DCCl3, 300 MHz)
8.05 8.02 7.37 7.35 7.35 7.34 7.33 7.32 7.32 7.31 7.30 7.30 7.28 7.28 7.21 7.21 7.20 7.19 7.19 7.18 7.18 7.18 7.01 7.01 6.99 6.98 6.81 5.47 5.37 3.88 2.91 2.88 2.86 2.84 1.96 1.93 1.91 1.91 1.89 1.86 1.61 1.59 1.59 1.57 1.57 1.55 1.54 1.52 1.22 1.20 0.78 0.75 0.73
Ph
N
N
O
OMe
5y
1.90 3.28 1.85 1.87 1.00 0.96 0.93 3.20 0.95 1.74 1.77 3.17 2.82
f1 (ppm)

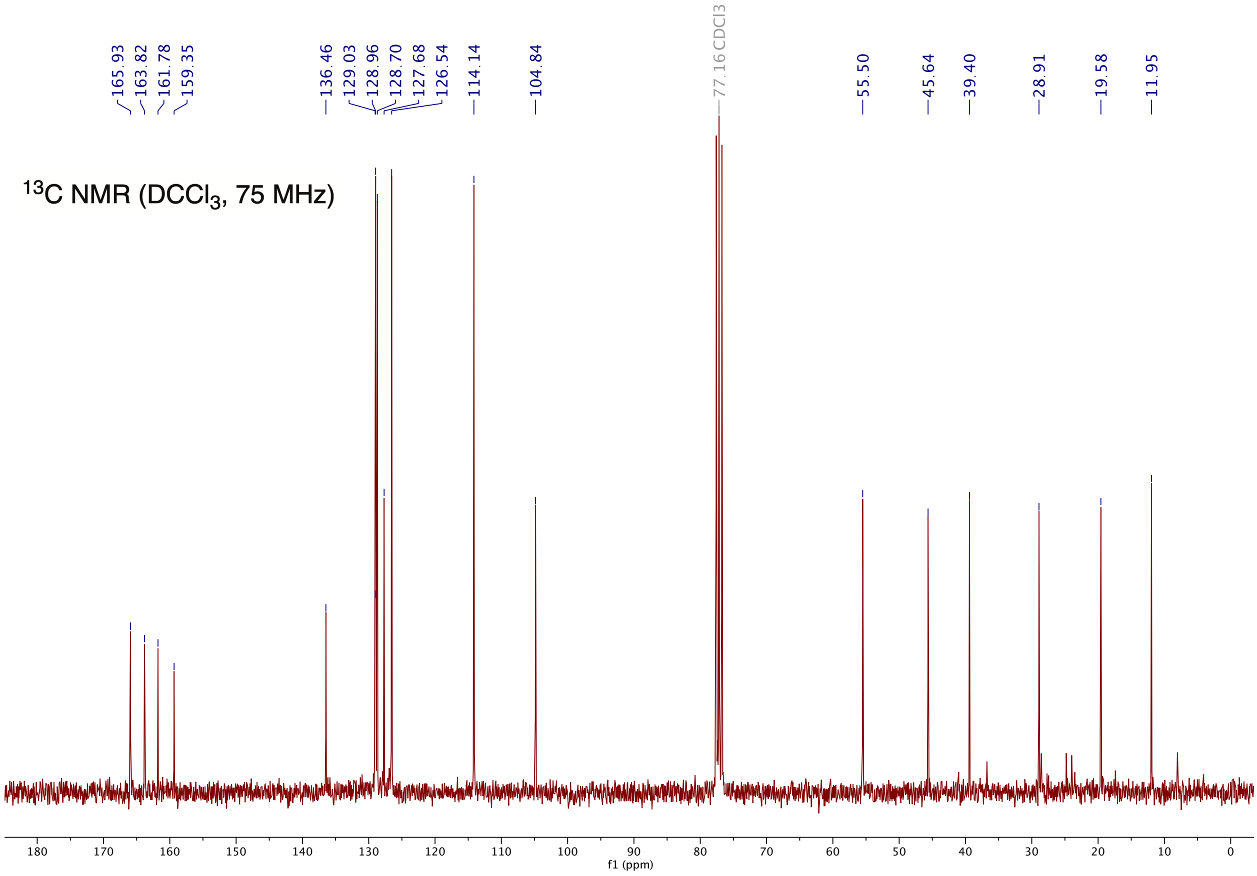
13C NMR (DCCl3, 75 MHz)
165.93 163.82 161.78 159.35 136.46 129.03 128.96 128.70 127.68 126.54 114.14 104.84 77.16 CDCl3 55.50 45.64 39.40 28.91 19.58 11.95
f1 (ppm)

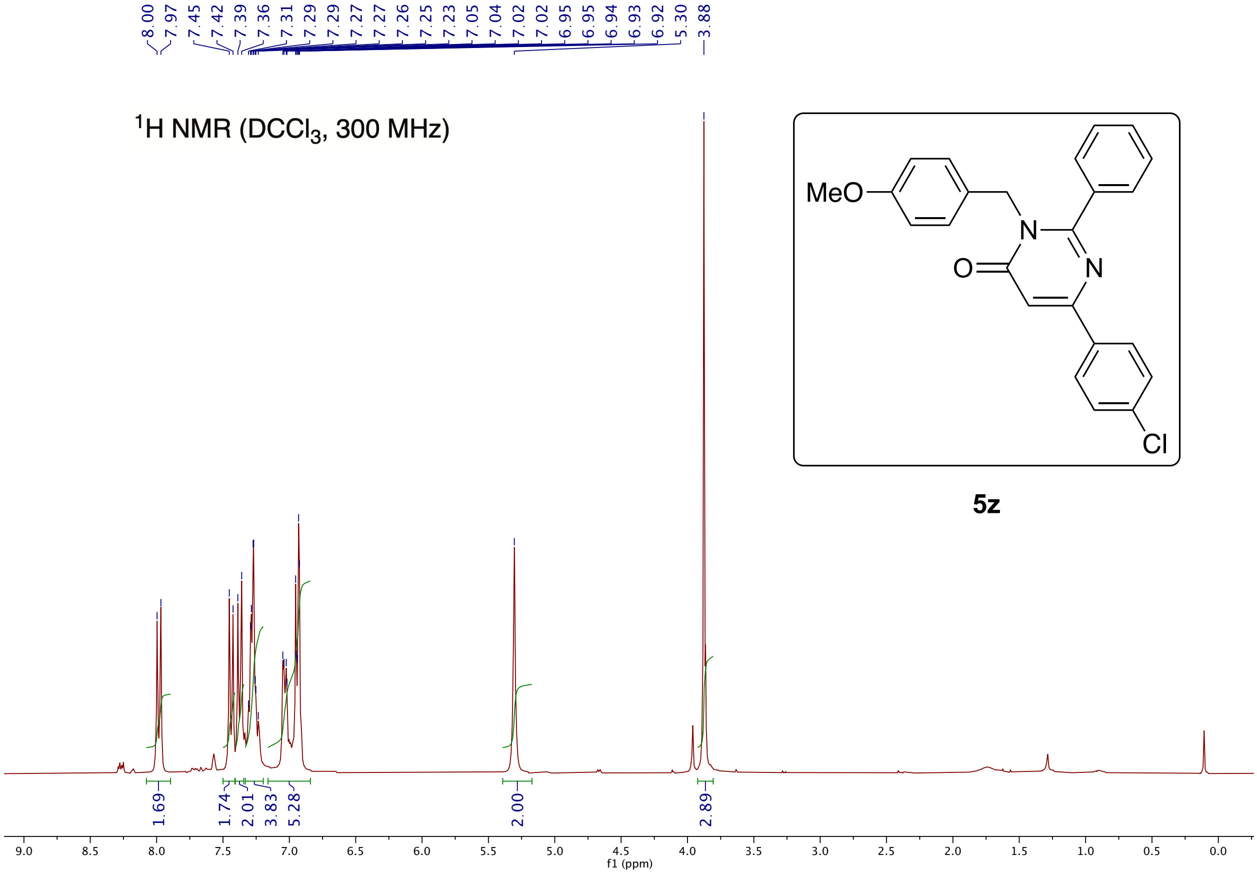
1H NMR (DCCl3, 300 MHz)
8.00 7.97 7.45 7.42 7.39 7.36 7.31 7.29 7.29 7.27 7.27 7.26 7.25 7.23 7.05 7.04 7.02 7.02 6.95 6.95 6.94 6.93 6.92 5.30 3.88
MeO
N
N
O
Cl
5z
1.69 1.74 2.01 3.83 5.28 2.00 2.89
f1 (ppm)

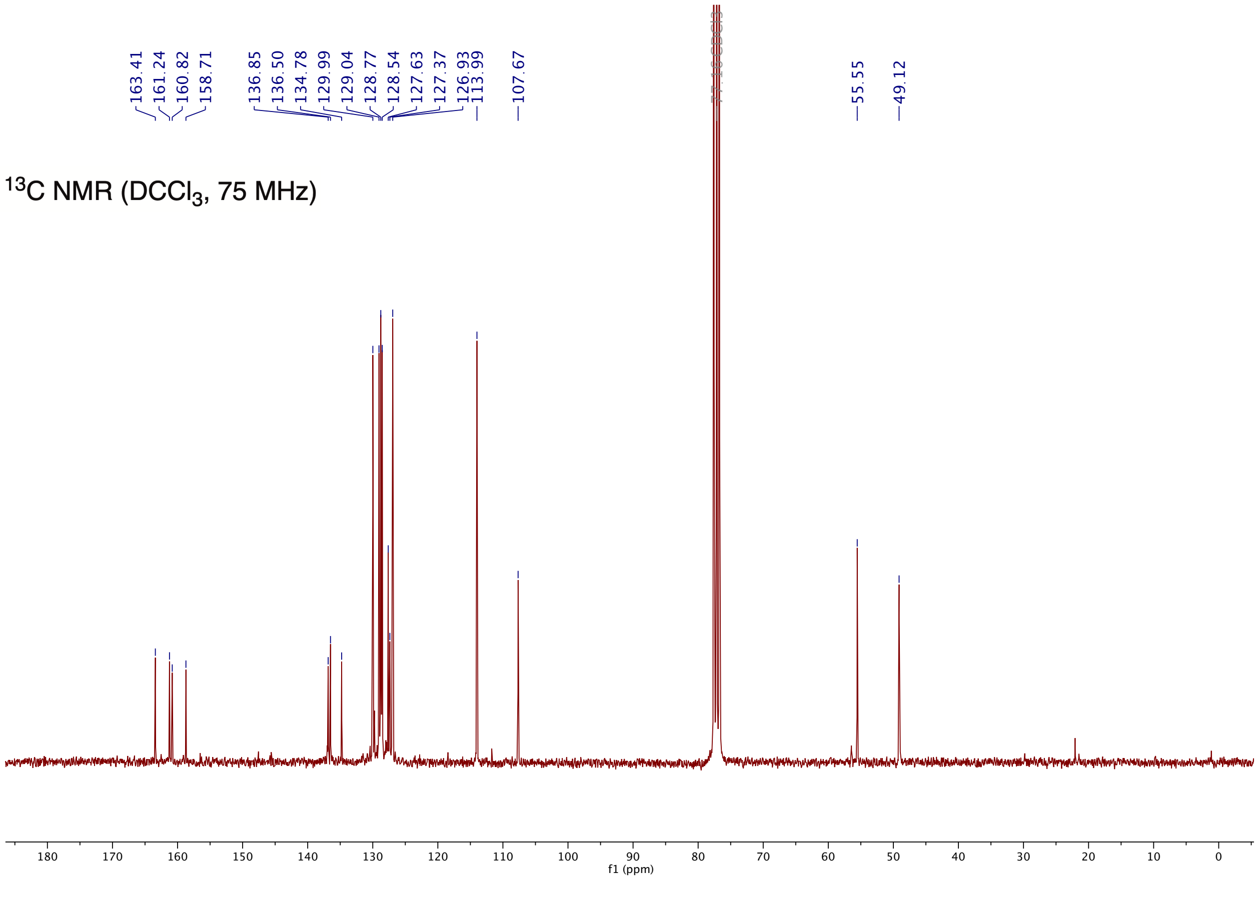
13C NMR (DCCl3, 75 MHz)
163.41 161.24 160.82 158.71 136.85 136.50 134.78 129.99 129.04 128.77 128.54 127.63 127.37 126.93 113.99 107.67 55.55 49.12
f1 (ppm)

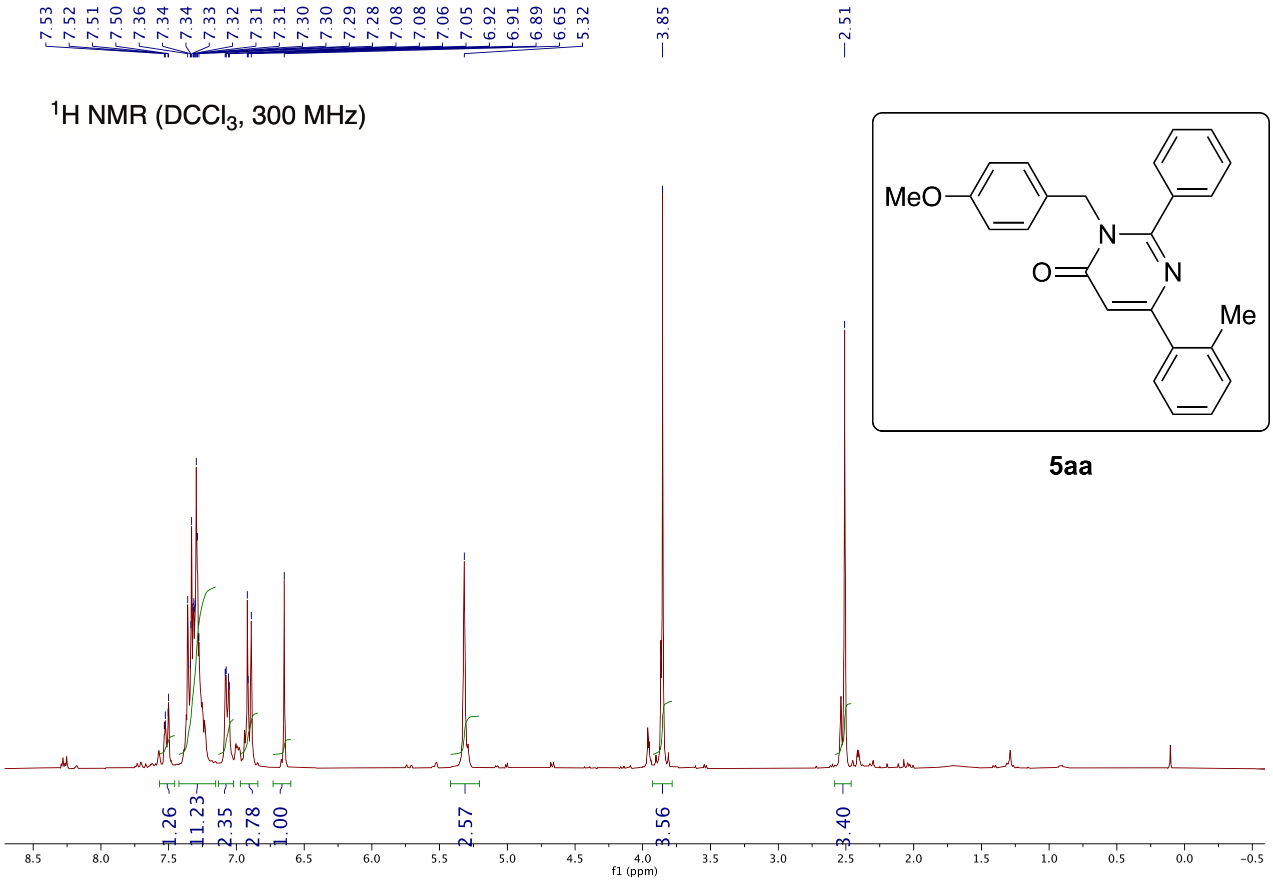
7.53
7.52
7.51
7.50
7.36
7.34
7.34
7.33
7.32
7.31
7.31
7.30
7.30
7.29
7.28
7.08
7.08
7.06
7.05
6.92
6.91
6.89
6.65
5.32
3.85
2.51
1H NMR (DCCl3, 300 MHz)
1.26
11.23
2.35
2.78
1.00
2.57
3.56
3.40
f1 (ppm)
MeO
N
N
O
Me

5aa

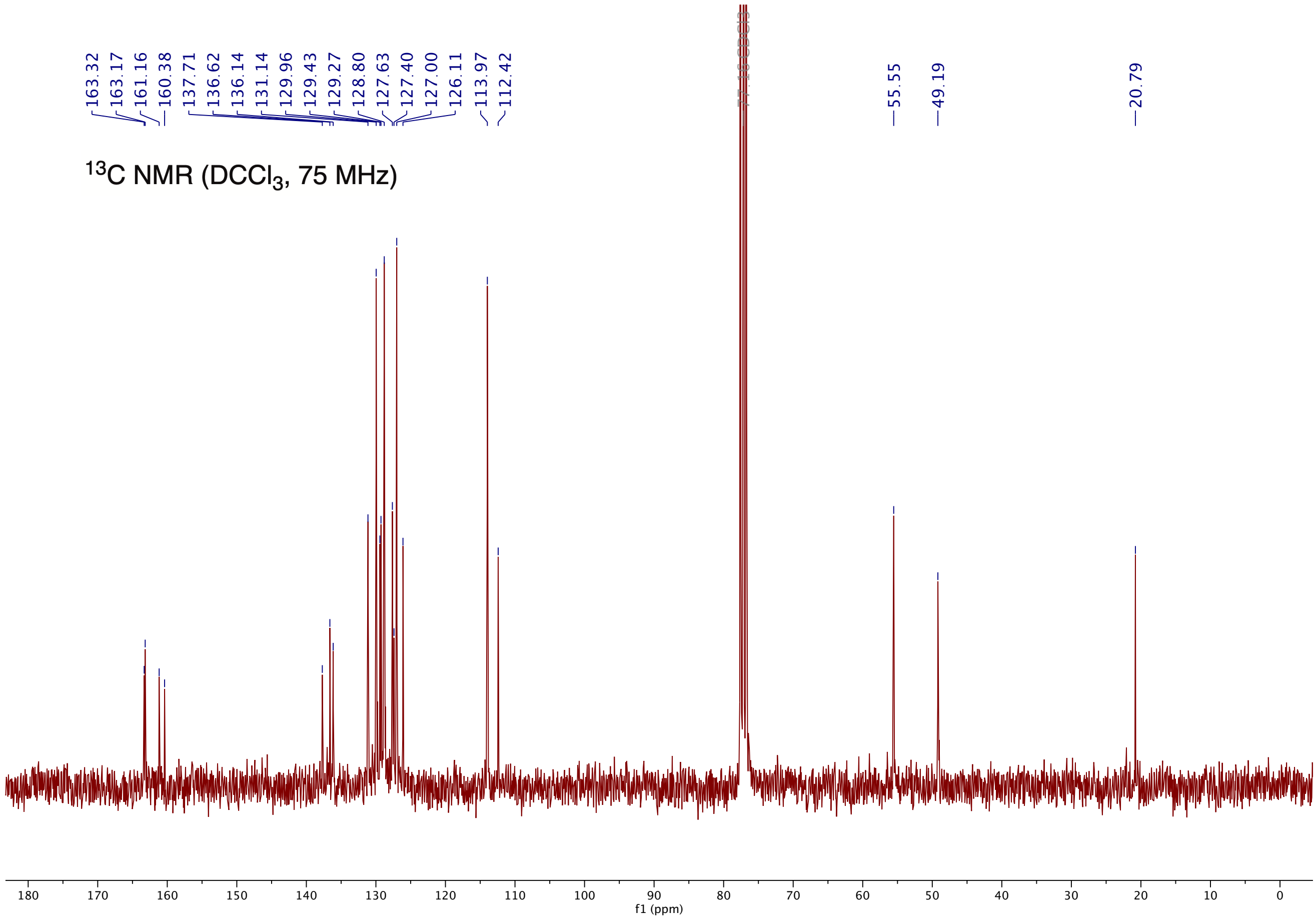
163.32
163.17
161.16
160.38
137.71
136.62
136.14
131.14
129.96
129.43
129.27
128.80
127.63
127.40
127.00
126.11
113.97
112.42
55.55
49.19
20.79
13C NMR (DCCl3, 75 MHz)
f1 (ppm)

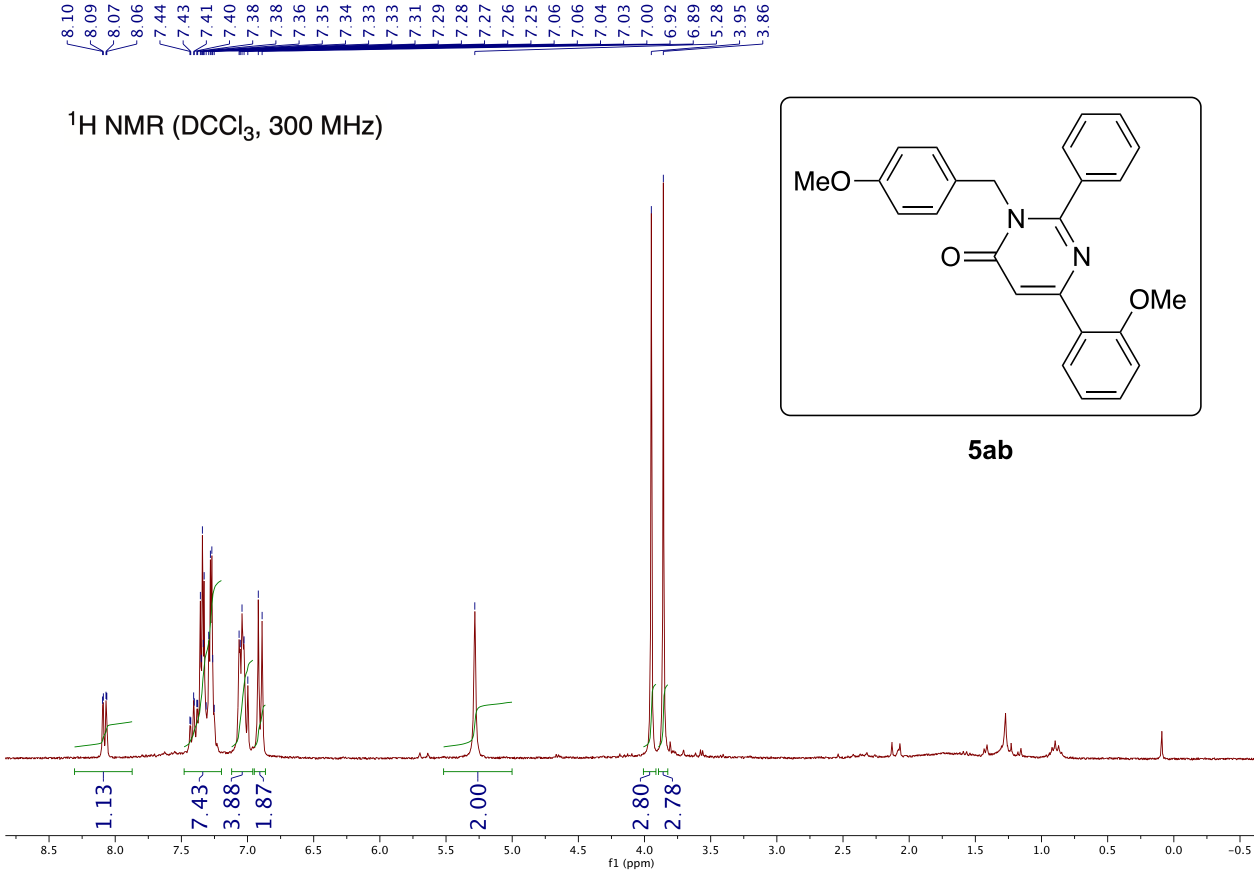
8.10 8.09 8.07 8.06 7.44 7.43 7.41 7.40 7.38 7.38 7.36 7.35 7.34 7.33 7.33 7.31 7.29 7.28 7.27 7.26 7.25 7.06 7.06 7.04 7.03 7.00 6.92 6.89 5.28 3.95 3.86
1H NMR (DCCl3, 300 MHz)
MeO
N
N
O
OMe
5ab
1.13
7.43
3.88
1.87
2.00
2.80
2.78
f1 (ppm)

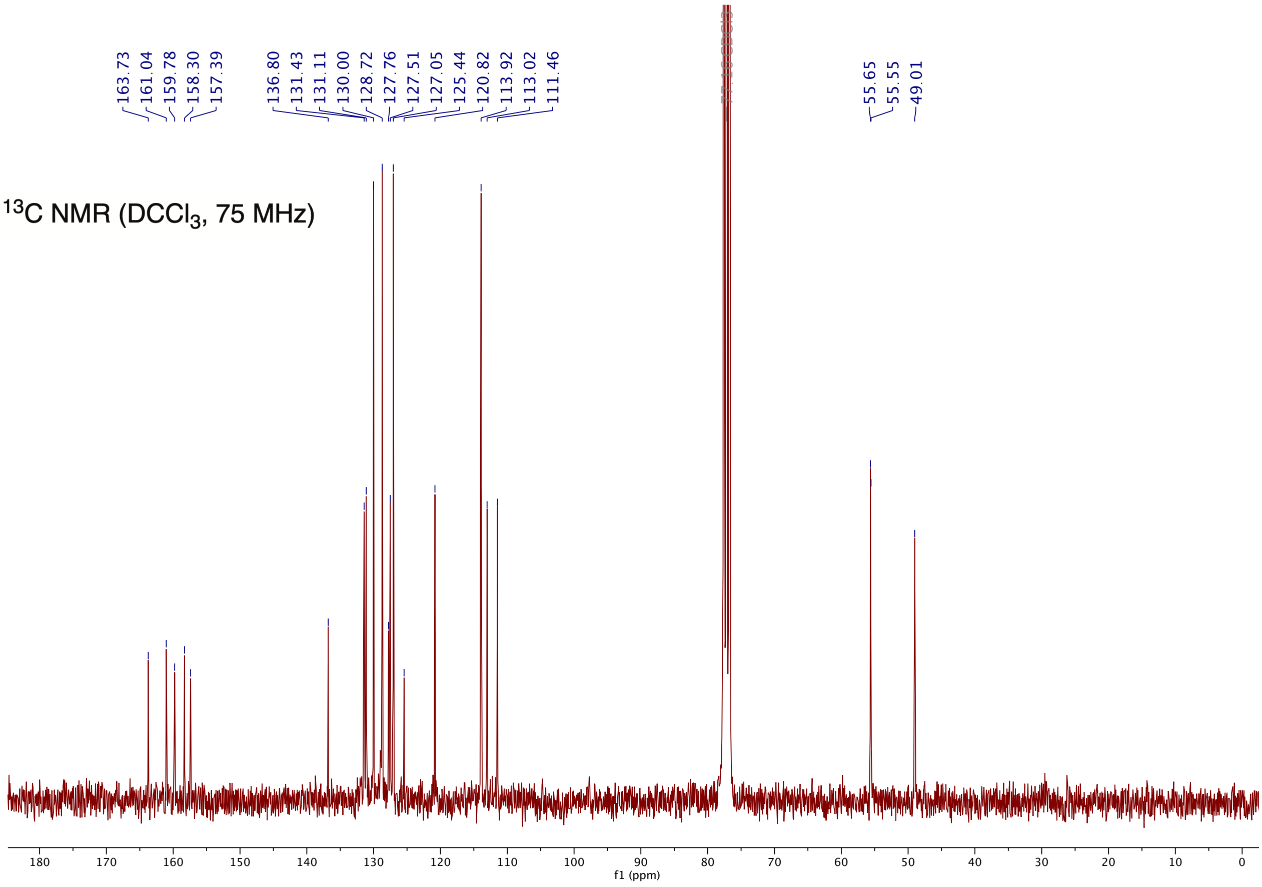
163.73 161.04 159.78 158.30 157.39
136.80 131.43 131.11 130.00 128.72 127.76 127.51 127.05 125.44 120.82 113.92 113.02 111.46
55.65 55.55 49.01
13C NMR (DCCl3, 75 MHz)
f1 (ppm)

^{1}H NMR (DCCl$_3$, 300 MHz)

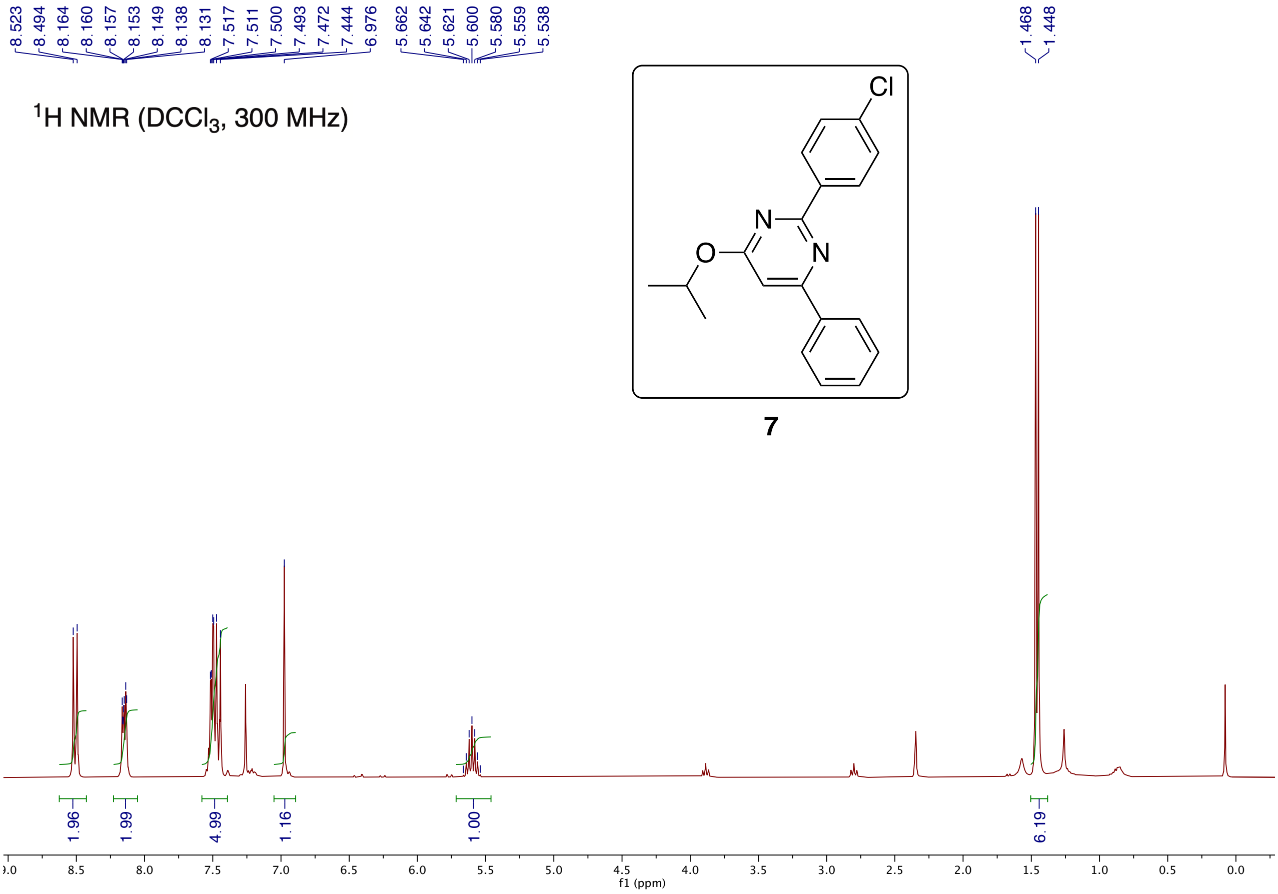

^{13}C NMR (DCCl$_3$, 75 MHz)

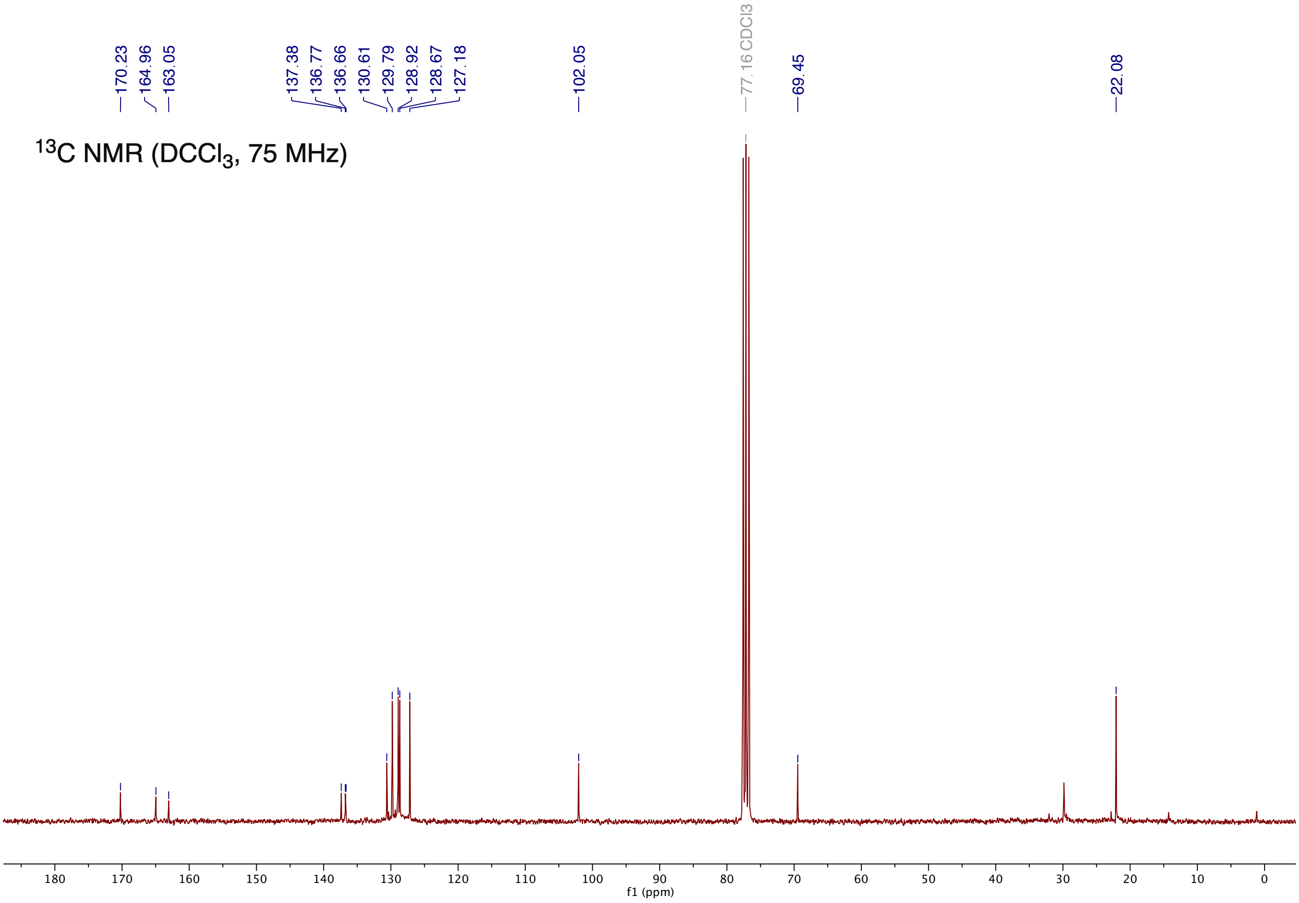

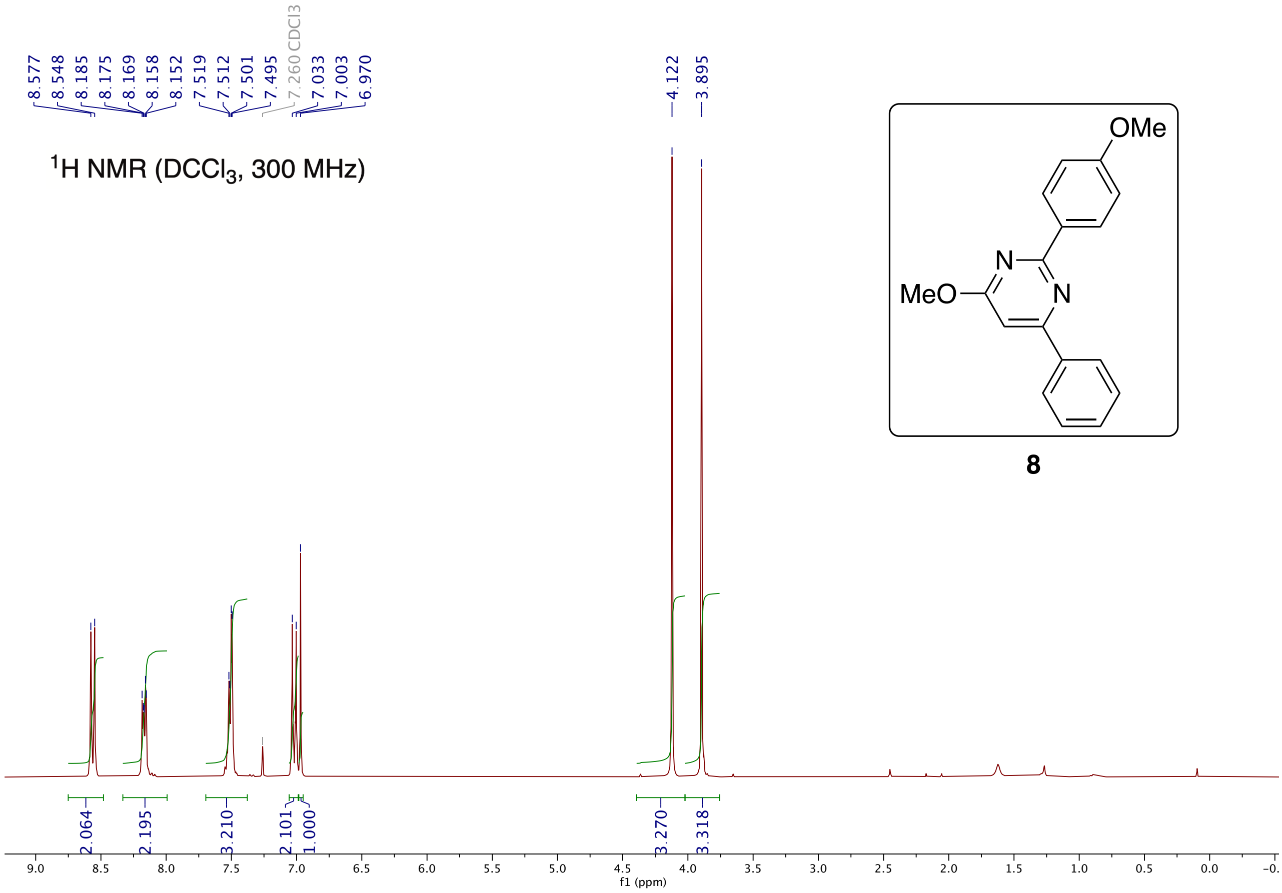

8.577
8.548
8.185
8.175
8.169
8.158
8.152
7.519
7.512
7.501
7.495
7.260 CDCl3
7.033
7.003
6.970
4.122
3.895
^{1}H NMR ($DCCl_3$, 300 MHz)
2.064
2.195
3.210
2.101
1.000
3.270
3.318
9.0
8.5
8.0
7.5
7.0
6.5
6.0
5.5
5.0
4.5
4.0
3.5
3.0
2.5
2.0
1.5
1.0
0.5
0.0
f1 (ppm)
OMe
N
N
MeO

8

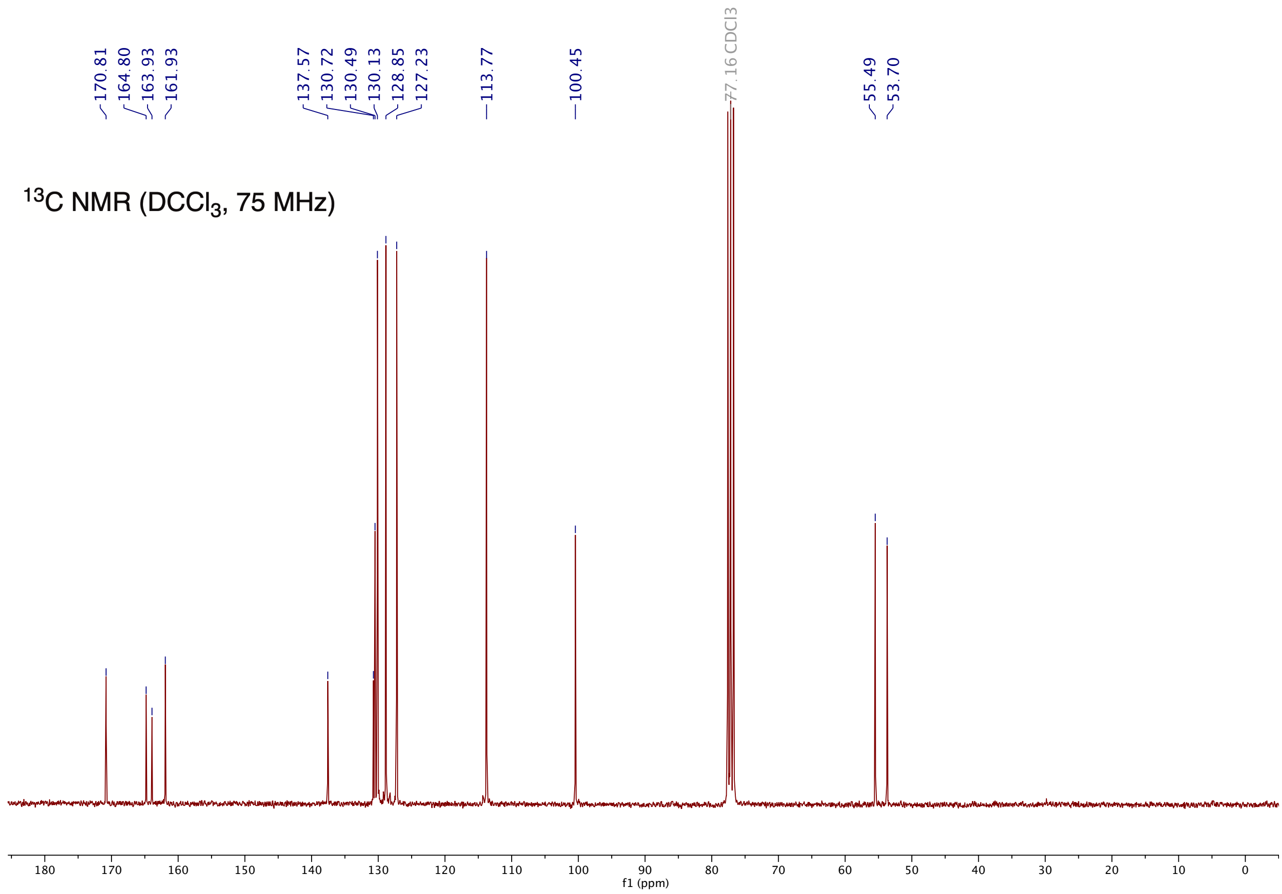

170.81
164.80
163.93
161.93
137.57
130.72
130.49
130.13
128.85
127.23
113.77
100.45
77.16 CDCl3
55.49
53.70
^{13}C NMR ($DCCl_3$, 75 MHz)
180
170
160
150
140
130
120
110
100
90
80
70
60
50
40
30
20
10
0
f1 (ppm)

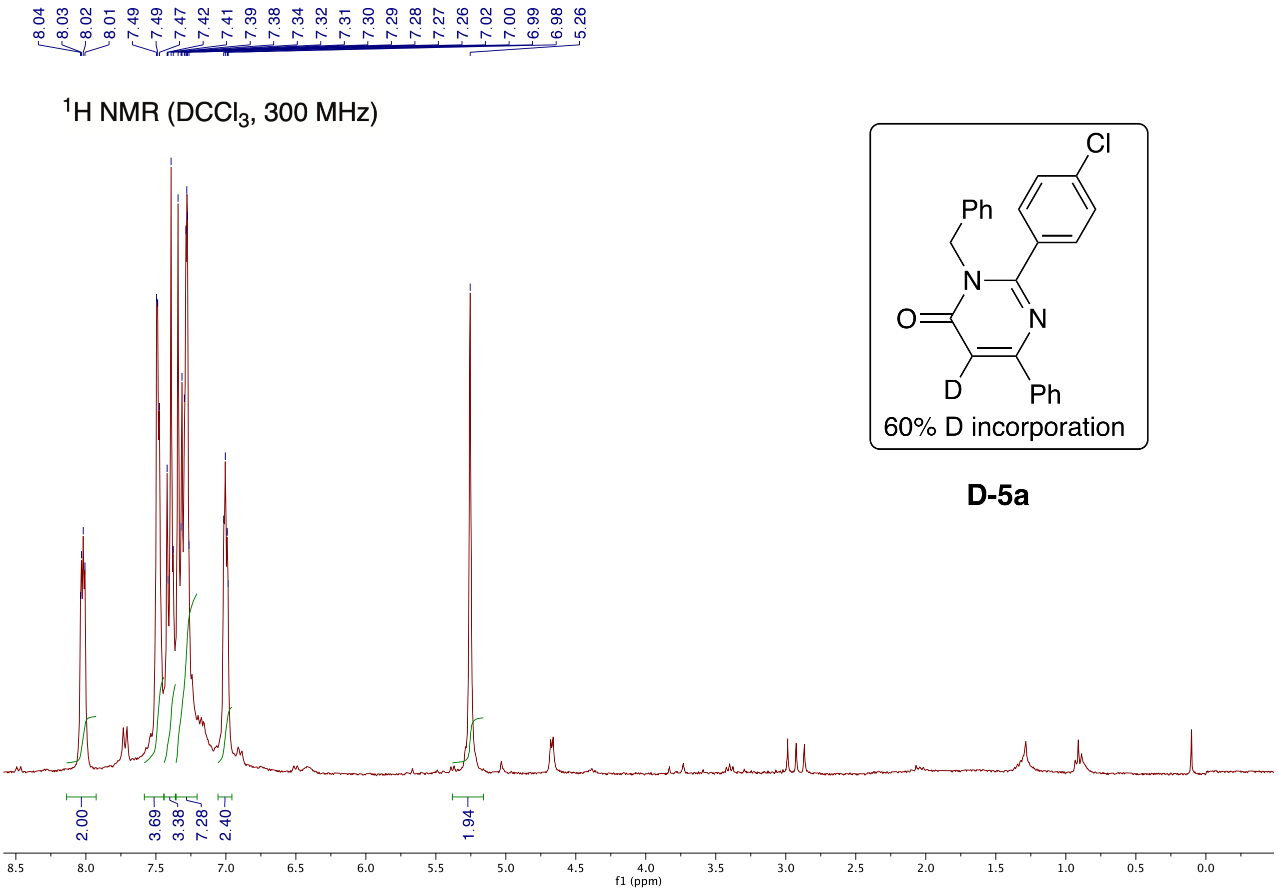
8.04 8.03 8.02 8.01 7.49 7.49 7.47 7.42 7.41 7.39 7.38 7.34 7.32 7.31 7.30 7.29 7.28 7.27 7.26 7.02 7.00 6.99 6.98 5.26
^{1}H NMR (DCCl$_3$, 300 MHz)
2.00 3.69 3.38 7.28 2.40 1.94
f1 (ppm)
Cl
Ph
N
N
O
D
Ph
60% D incorporation

D-5a

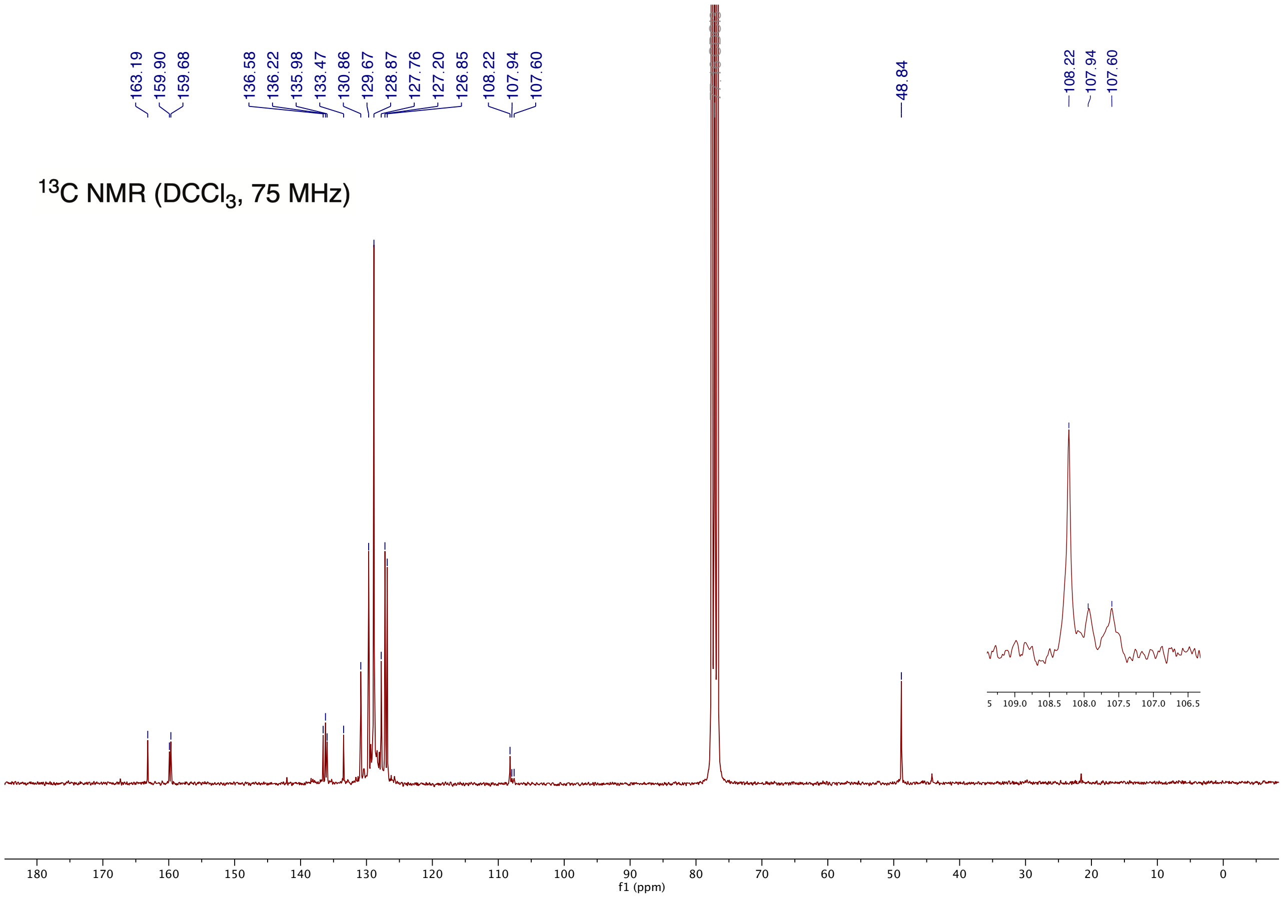
163.19 159.90 159.68 136.58 136.22 135.98 133.47 130.86 129.67 128.87 127.76 127.20 126.85 108.22 107.94 107.60 48.84
108.22 107.94 107.60
^{13}C NMR (DCCl$_3$, 75 MHz)
f1 (ppm)

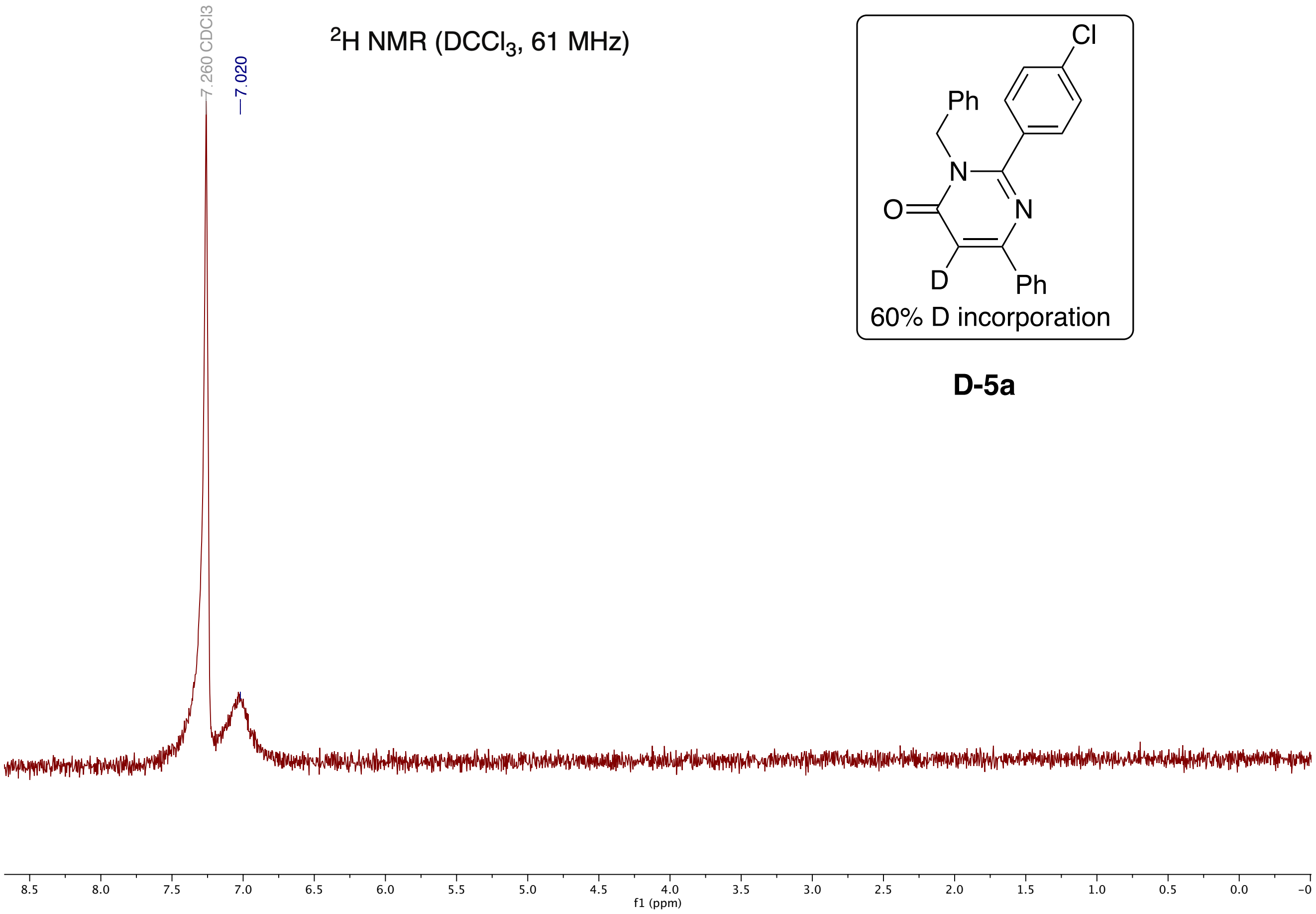

^2H NMR ($DCCl_3$, 61 MHz)
7.260 CDCl3
7.020
Cl
Ph
N
N
O
D
Ph
60% D incorporation
D-5a
8.5
8.0
7.5
7.0
6.5
6.0
5.5
5.0
4.5
4.0
3.5
3.0
2.5
2.0
1.5
1.0
0.5
0.0
f1 (ppm)

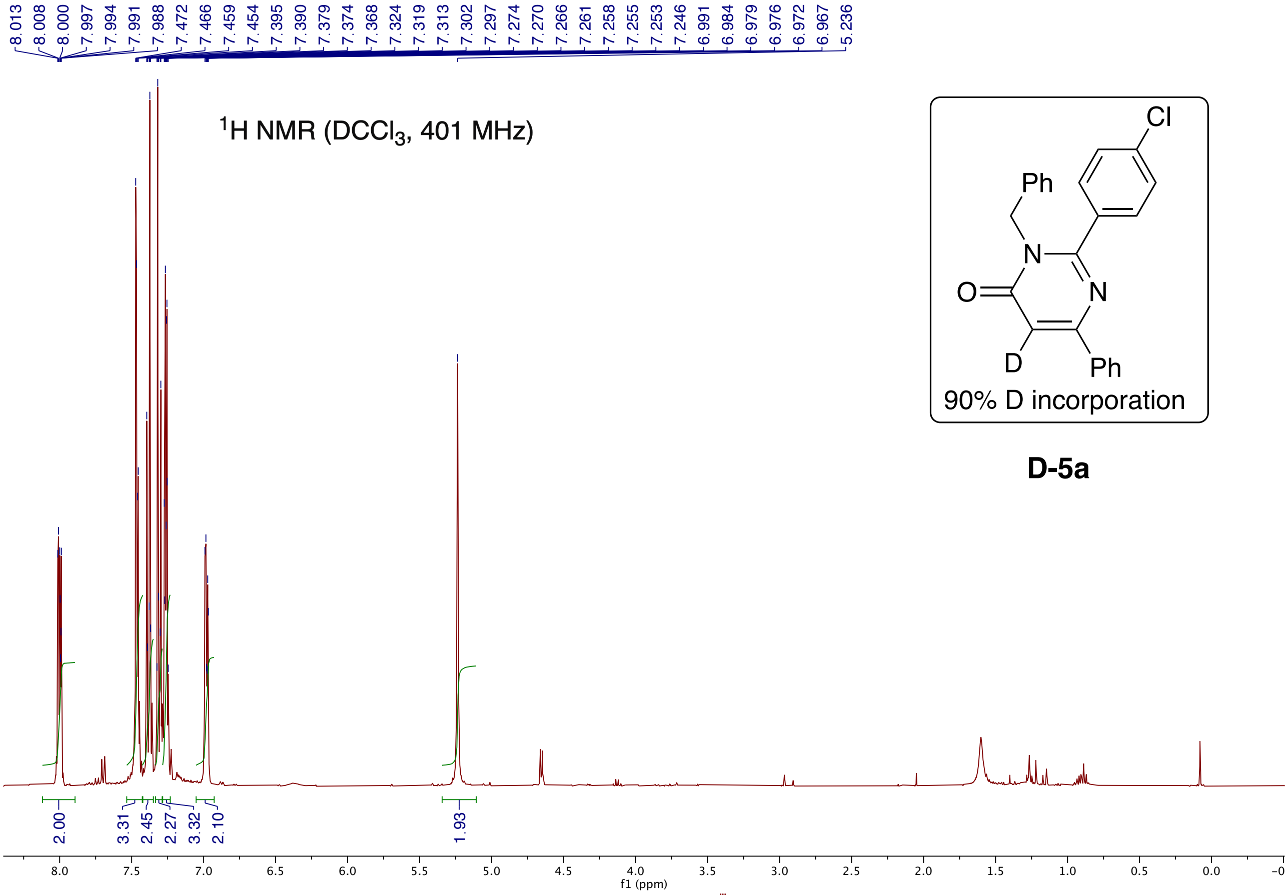
8.013
8.008
8.000
7.997
7.994
7.991
7.988
7.472
7.466
7.459
7.454
7.395
7.390
7.379
7.374
7.368
7.324
7.319
7.313
7.302
7.297
7.274
7.270
7.266
7.261
7.258
7.255
7.253
7.246
6.991
6.984
6.979
6.976
6.972
6.967
5.236
^{1}H NMR ($DCCl_3$, 401 MHz)
2.00
3.31
2.45
2.27
3.32
2.10
1.93
f1 (ppm)
Cl
Ph
N
N
O
D
Ph
90% D incorporation

D-5a

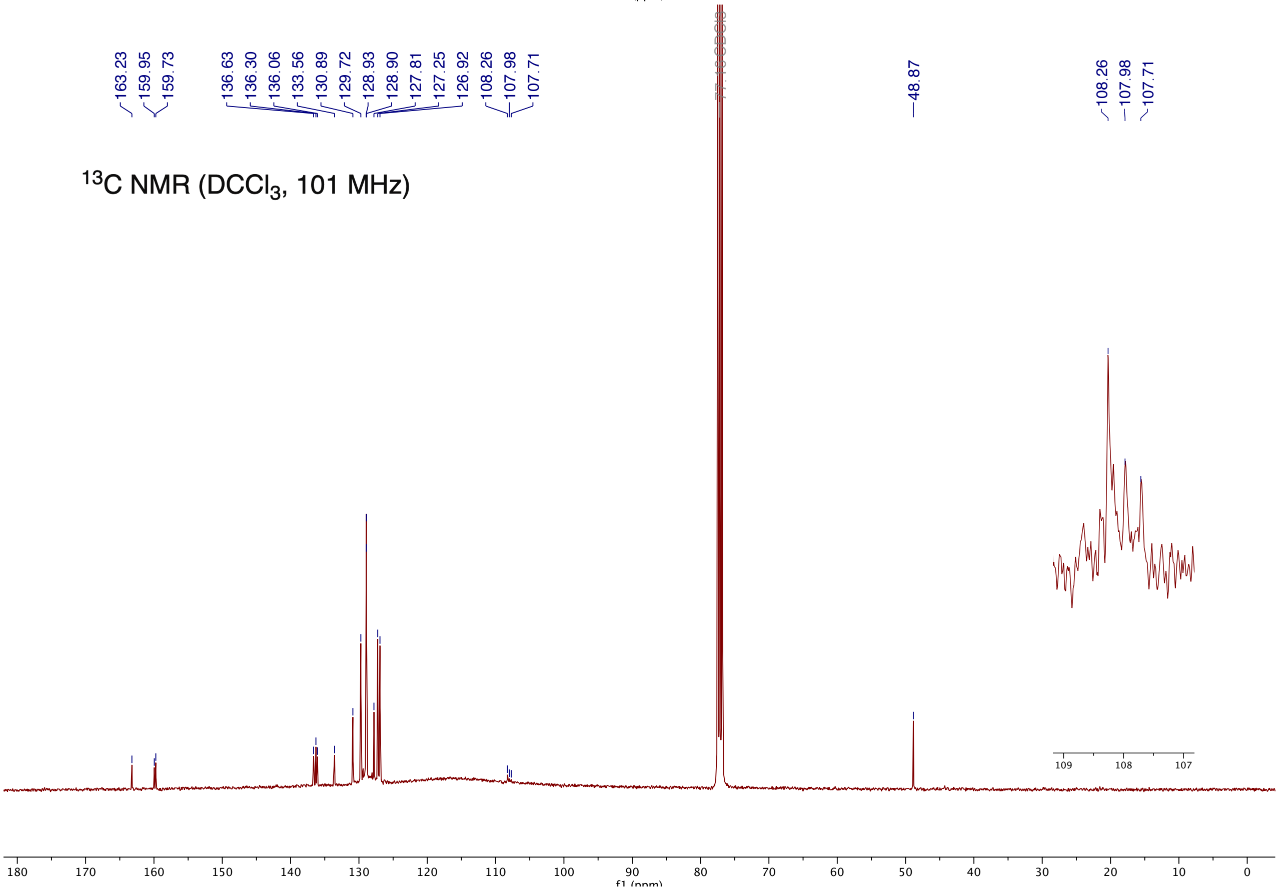
163.23
159.95
159.73
136.63
136.30
136.06
133.56
130.89
129.72
128.93
128.90
127.81
127.25
126.92
108.26
107.98
107.71
48.87
108.26
107.98
107.71
^{13}C NMR ($DCCl_3$, 101 MHz)
f1 (ppm)

^{2}H NMR ($DCCl_3$, 61 MHz)

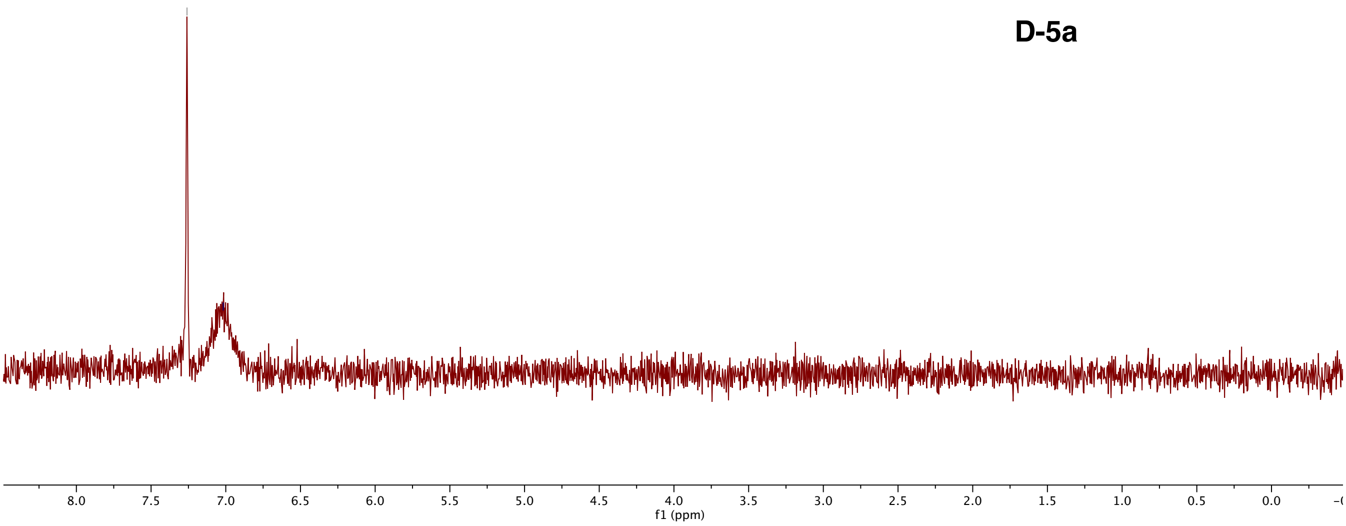

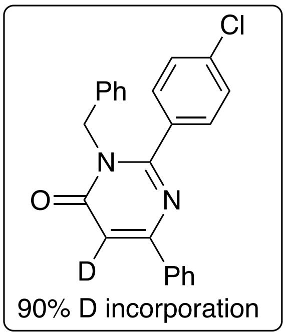

D-5a

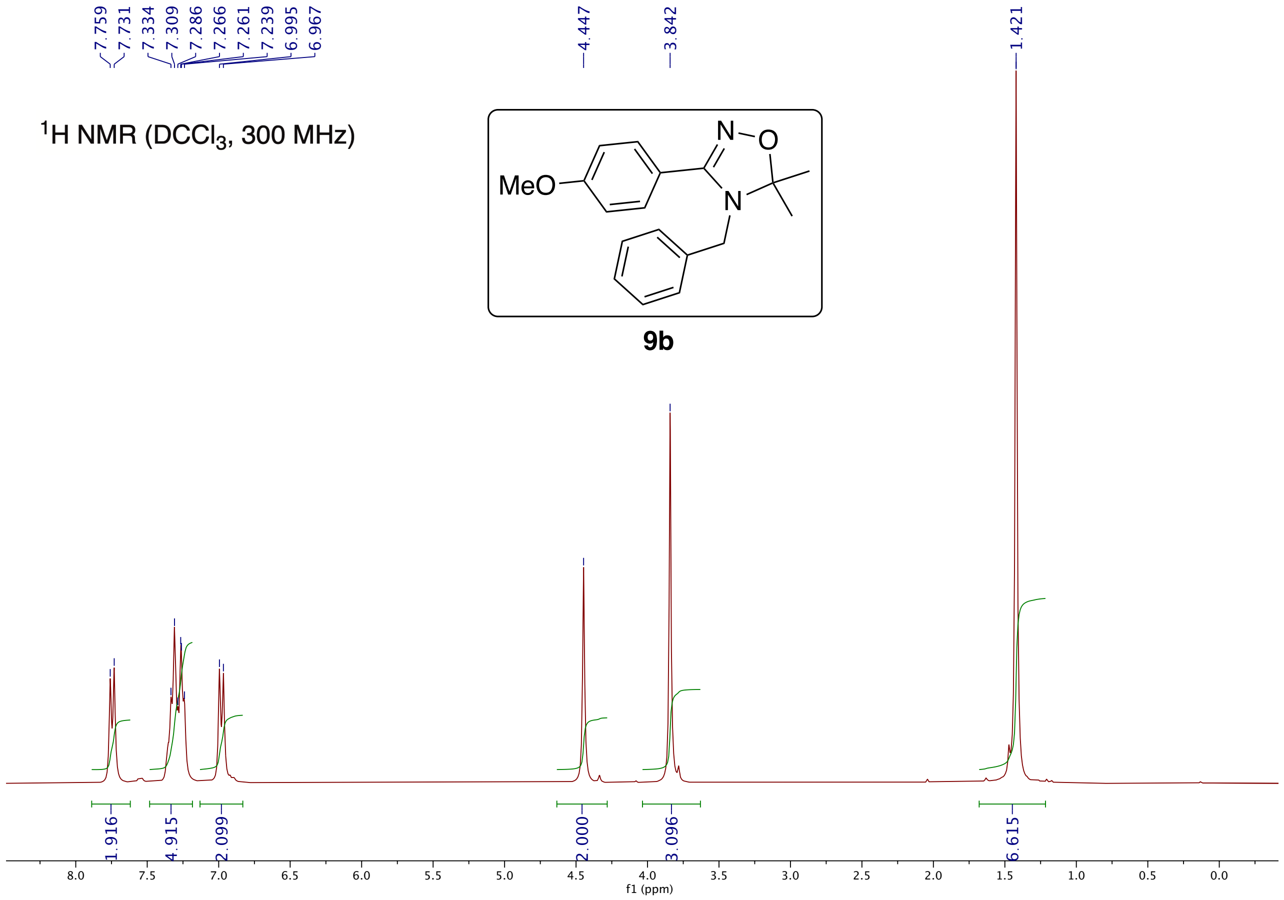
7.759
7.731
7.334
7.309
7.286
7.266
7.261
7.239
6.995
6.967
4.447
3.842
1.421
1H NMR (DCCl3, 300 MHz)
MeO
N
O
N
9b
1.916
4.915
2.099
2.000
3.096
6.615
f1 (ppm)

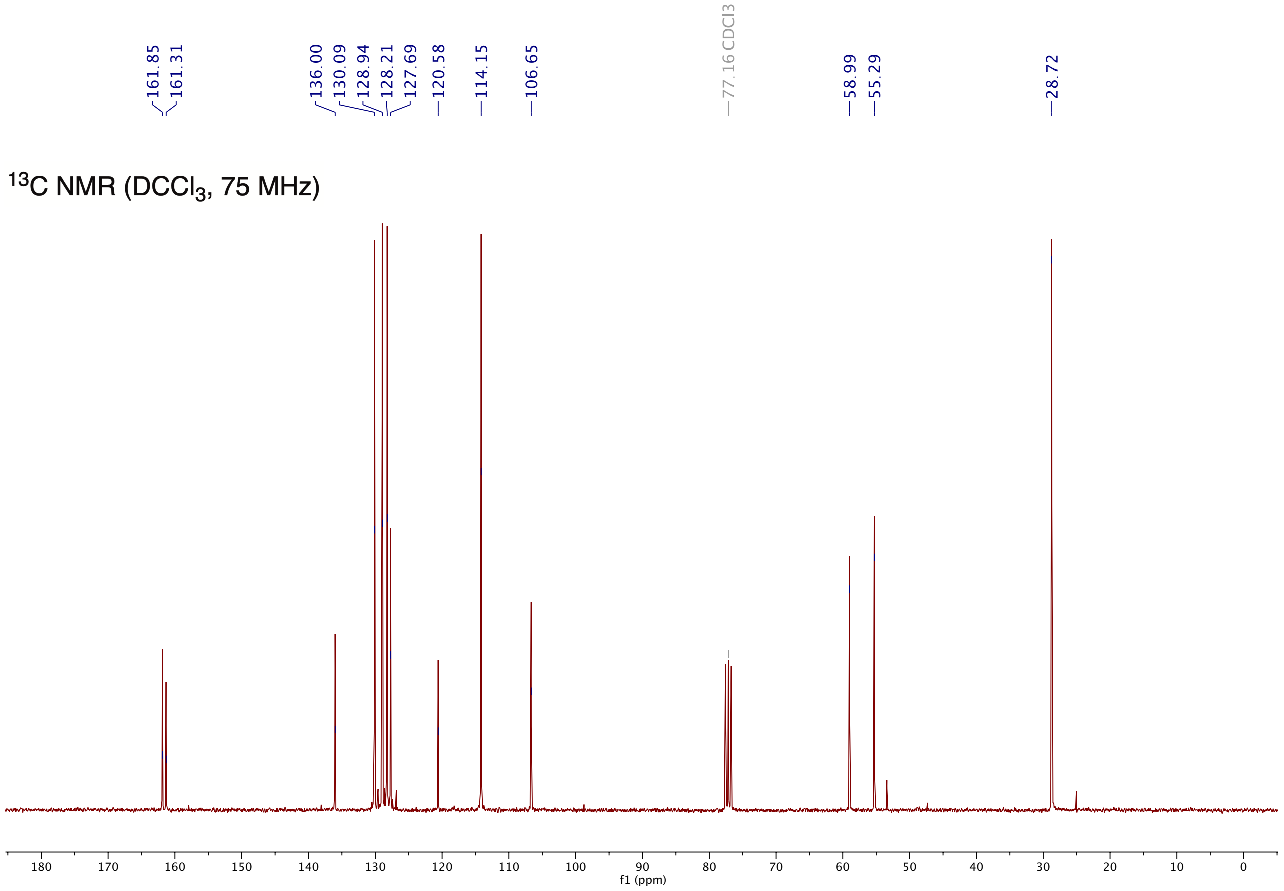
161.85
161.31
136.00
130.09
128.94
128.21
127.69
120.58
114.15
106.65
77.16 CDCl3
58.99
55.29
28.72
13C NMR (DCCl3, 75 MHz)
f1 (ppm)

^{1}H NMR ($DCCl_3$, 300 MHz)

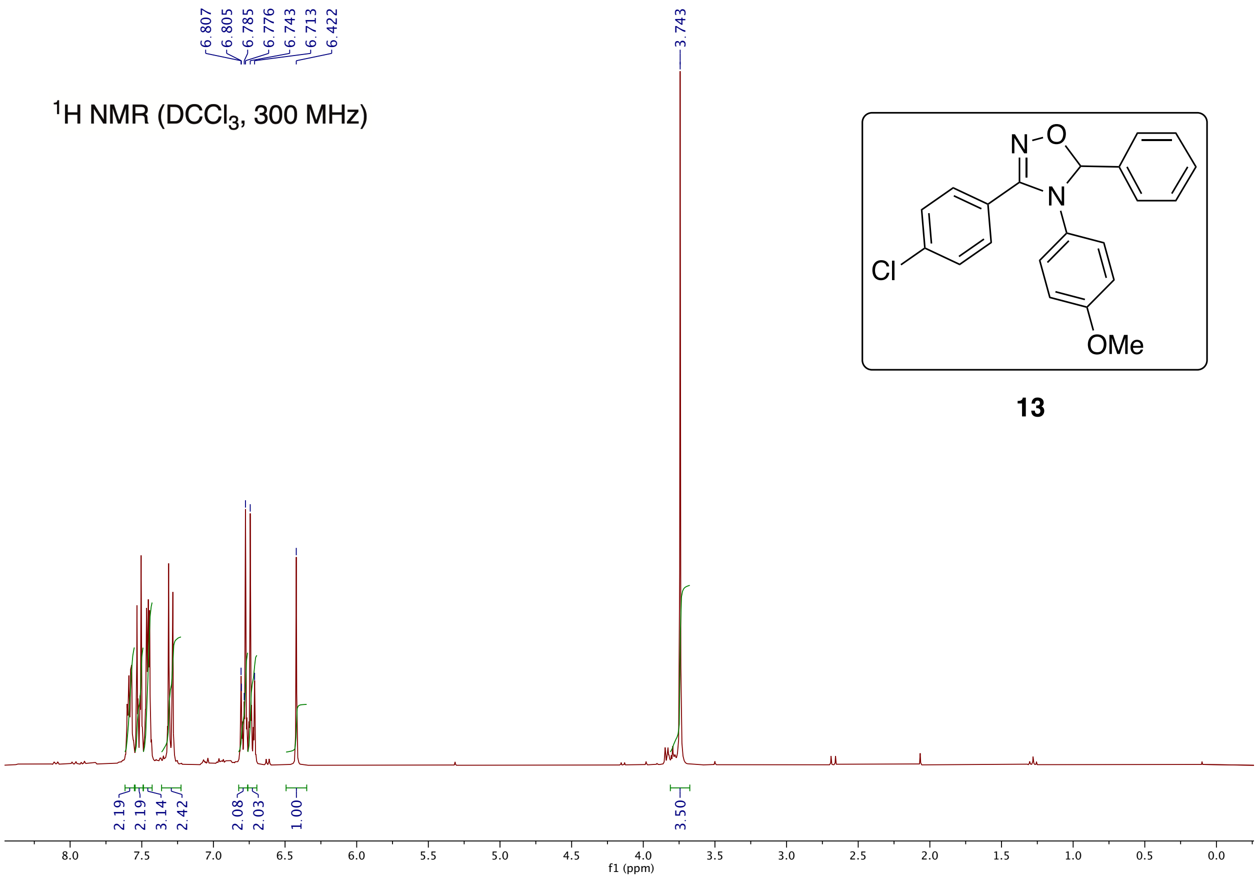

13

^{13}C NMR ($DCCl_3$, 75 MHz)

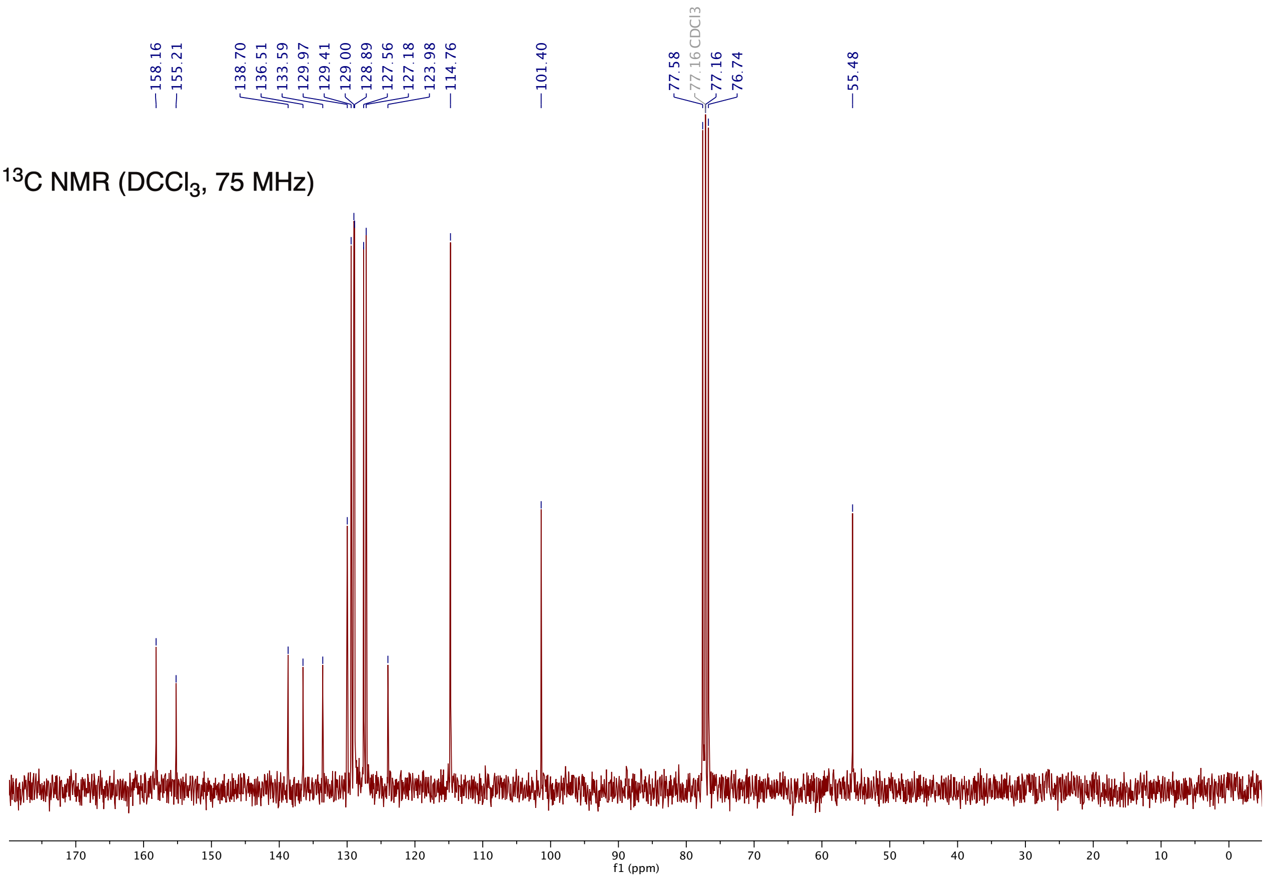

Supplement: Supplementary file 1 [file ol5c03642_si_001.pdf]
